# Supplementary material for: Silane- and peroxide-free hydrogen atom transfer hydrogenation using ascorbic acid and cobalt-photoredox dual catalysis
Source: Nat Commun. 2021 Feb 11;12:966. doi: 10.1038/s41467-020-20872-z (PMC7878493; doi:10.1038/s41467-020-20872-z)
Supplement: Supplementary file 1 — Supplementary Information [file 41467_2020_20872_MOESM1_ESM.pdf]

Supplementary Information

Silane- and peroxide-free hydrogen atom transfer hydrogenation using ascorbic acid and cobalt-photoredox dual catalysis

Table of contents

1. General 2
2. Experimental procedures 3
  2-1. Synthesis of substrates 3
  2-2. Synthesis of the cobalt complexes 18
  2-3. Ascorbic-acid-mediated hydrogenation by cobalt-photoredox dual catalysis 19
  2-4. Comparison of the hydrogenation performance for the preparation of IBCG **2ah** 37
  2-5. Detection of the alkyl radical intermediate 38
3. Additional investigations of reaction conditions 40
  3-1. Effect of other photocatalysts 40
  3-2. Effect of other solvents 40
  3-3. Effect of other acidic reducing agents 41
4.Additional mechanistic studies 42
  4-1. Stern-Volmer quenching experiment 42
  4-2. Electrochemical analysis in the presence of a protic solvent 43
  4-3. Detection of H_2 evolution 44
  4-4. Attempt of hydrogenation using H_2 gas 45
5. Computational methods 47
  5-1. General 47
  5-2. Discussion regarding the description of radical pairs 47
  5-3. Computed structures and electronic energies 48
6. NMR spectra 55
7. Supplementary references 151

1. General

Reactions were carried out under argon atmosphere unless otherwise noted. NMR spectra were recorded on JEOL JNM-ECS400 spectrometers operating at 391.78 MHz for ^{1}H NMR and 98.52 MHz for ^{13}C NMR, JEOL JNM-ECX400 spectrometers operating at 396 MHz for ^{1}H NMR and 99.55 MHz for ^{13}C NMR, and JNM-ECA500 spectrometers operating at 500.16 MHz for ^{1}H NMR and 125.77 MHz for ^{13}C NMR. Chemical shifts were reported in the scale relative to TMS (0.00 ppm for ^{1}H NMR in $CDCl_3$), $CHCl_3$ (7.26 ppm for ^{1}H NMR in $CDCl_3$), DOH (4.65 ppm for ^{1}H NMR in D_2O), $CDCl_3$ (77.00 ppm for ^{13}C NMR in $CDCl_3$), $PhCF_3$ (–63.72 ppm for ^{19}F NMR in $CDCl_3$) as an internal reference, respectively, and CH_3OH (49.50 ppm for ^{13}C NMR in D_2O) as an external reference. ESI mass spectra were measured on Thermo Scientific Exactive or Waters ACQUITY UPLC Xevo G2 QTof. Infrared (IR) spectra were recorded on a JASCO FT/IR-5300 spectrophotometer, and absorbance bands are reported in wavenumbers (inverse centimeters). Column chromatography was performed with silica gel Kanto Silica gel 60 N (40-50 mesh) or Yamazen YFLC AI-580 using Universal Column SiOH. Reverse phase column chromatography was performed with Yamazen YFLC AI-580 using Universal Column ODS. Gel permeation chromatography (GPC) was performed with YMC LC-forte/R. Gas chromatography was performed on a Shimadzu GC-2010 Plus system with a flame ionization detector using a capillary column and helium as a carrier gas. Retention times and peak ratios were determined with Shimadzu GCsolution. Liquid chromatography was performed on a Shimadzu LCMS-2020 using InertSustainswift® C18 (3.0 µm, 3.0 mm x 100 mm) column and CH_3CN/H_2O with 0.1% formic acid as an eluent. Retention times and peak ratios were determined with Shimadzu Labsolutions LCMS. Cyclic voltammetry was performed with BAS ALS/DY2325 potentiostat and BAS model2325 software and the measurements were conducted in DMF (0.1 M tetrabutylammonium perchlorate) under argon atmosphere with a standard three electrode setup (working: glassy carbon, reference: $Ag/AgNO_3$, counter: platinum). All non-aqueous reactions were carried out in a flame-dried glassware under argon atmosphere unless otherwise noted or in an argon-filled glove box. Dichloromethane (CH_2Cl_2), tetrahydrofuran (THF), diethyl ether (Et_2O), and toluene were purified by Glass Contour solvent purification system (Nikko Hansen & Co., Ltd.,) before use. All other reagents were commercially available and used as received unless otherwise noted. Irradiation was performed with ISLM-150X150-BB447 (purchased from CCS Inc.) as photon source. UV-Vis spectra were measured with SHIMADZU UV-1850. Measurement of luminescence quenching was performed with JASCO FP-8600.

2. Experimental procedures

2-1. Synthesis of substrates

General Procedure A

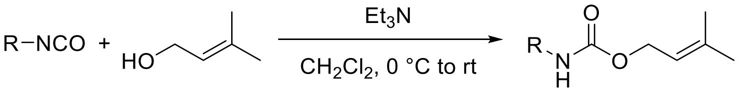

To a solution of the corresponding isocyanate (1.0 equiv) in CH_2Cl_2 (1.0 M), triethylamine (3.0 equiv) and prenol (1.0 equiv) were sequentially added at 0 °C. The mixture was allowed to warm to room temperature and was stirred overnight. The mixture was diluted with CH_2Cl_2 and washed with 1 M aq. HCl (x 3), water (x 1), and brine (x 1). The organic layer was concentrated under reduced pressure, and the obtained crude was purified by column chromatography (silica gel, hexane/EtOAc) to afford the carbamate product.

3-methylbut-2-en-1-yl phenylcarbamate (1a)

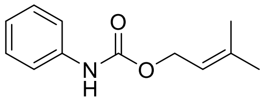

According to the general procedure A, **1a** was prepared from phenyl isocyanate (2.4 g, 20 mmol), prenol (1.7 g, 20 mmol) and Et_3N (6.1 g, 60 mmol). The crude product was purified by column chromatography (silica gel, hexane/EtOAc = 20:1 to 10:1) to provide **1a** (3.6 g, 88%) as a colorless solid. NMR spectra of the obtained product were consistent with the reported one.[1]

^{1}H NMR (400 MHz, $CDCl_3$) δ 1.75 (s, 3H), 1.78 (s, 3H), 4.67 (d, J = 7.2 Hz, 2H), 5.38-5.42 (m, 1H), 6.56 (s, 1H), 7.05 (t, J = 7.3 Hz, 1H), 7.28-7.32 (m, 2H), 7.37 (d, J = 7.8 Hz, 2H).

^{13}C NMR (125 MHz, $CDCl_3$) δ 17.95, 25.68, 61.92, 118.62, 118.66, 123.21, 128.91, 137.93, 139.19, 153.64.

3-methylbut-2-en-1-yl *p*-tolylcarbamate (1b)

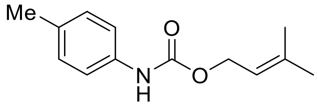

According to the general procedure A, **1b** was prepared from 4-methylphenyl isocyanate (0.67 g, 5.0 mmol), prenol (0.43 g, 5.0 mmol) and Et_3N (1.52 g, 15 mmol). The crude product was purified by column chromatography (silica gel, hexane/EtOAc = 7:1) to provide **1b** (0.94 g, 86%) as a colorless solid.

TLC: R_f = 0.46 (hexane/EtOAc = 5:1).

^{1}H NMR (500 MHz, $CDCl_3$) δ 1.75 (s, 3H), 1.78 (s, 3H), 2.30 (s, 3H), 4.66 (d, J = 7.3 Hz, 2H), 5.40 (t, J = 7.3 Hz, 1H), 6.50 (s, 1H), 7.10 (d, J = 8.4 Hz, 2H), 7.25 (d, J = 9.0 Hz, 2H).

^{13}C NMR (125 MHz, $CDCl_3$) δ 17.95, 20.65, 25.69, 61.84, 118.67, 118.77, 129.40, 132.76, 135.35, 139.06, 153.74.
HRMS (ESI): *m/z* calculated for $C_{13}H_{17}NO_2Na$ $[M+Na]^+$: 242.1152, found: 242.1156.

3-methylbut-2-en-1-yl (4-methoxyphenyl)carbamate (1c)

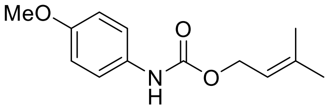

According to the general procedure A, **1c** was prepared from 4-methoxylphenyl isocyanate (0.75 g, 5.0 mmol), prenol (0.43 g, 5.0 mmol) and Et_3N (1.5 g, 15 mmol). The crude product was purified by column chromatography (silica gel, hexane/EtOAc = 10:1 to 5:1) to provide **1c** (0.93 g, 79%) as a colorless solid. NMR spectra of the obtained product were consistent with the reported one.[2]
^{1}H NMR (400 MHz, $CDCl_3$) δ 1.74 (s, 3H), 1.77 (s, 3H), 3.78 (s, 3H), 4.65 (d, J = 7.2 Hz, 2H), 5.37-5.41 (m, 1H), 6.46 (s, 1H), 6.84 (d, J = 9.2 Hz, 2H), 7.27 (d, J = 9.5 Hz, 2H).
^{13}C NMR (125 MHz, $CDCl_3$) δ 17.91, 25.66, 55.36, 61.80, 114.09, 118.79, 120.54, 131.03, 138.94, 154.01, 155.76.

3-methylbut-2-en-1-yl (4-(trifluoromethyl)phenyl)carbamate (1d)

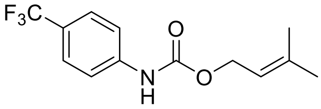

According to the general procedure A, **1d** was prepared from 4-trifluoromethylphenyl isocyanate (0.94 g, 5.0 mmol), prenol (0.43 g, 5.0 mmol) and Et_3N (1.5 g, 15 mmol). The crude product was purified by column chromatography (silica gel, hexane/EtOAc = 7:1) to provide **1d** (1.1 g, 71%) as a colorless solid.
TLC: R_f = 0.39 (hexane/EtOAc = 5:1).
^{1}H NMR (400 MHz, $CDCl_3$) δ 1.76 (s, 3H), 1.79 (s, 3H), 4.69 (d, J = 7.7 Hz, 2H), 5.40 (t, J = 7.2 Hz, 1H), 6.72 (s, 1H), 7.49 (d, J = 9.1 Hz, 2H), 7.56 (d, J = 8.6 Hz, 2H).
^{13}C NMR (125 MHz, $CDCl_3$) δ 17.97, 25.69, 62.36, 118.03, 118.29, 124.14 (q, J = 271.1 Hz), 125.06 (q, J = 32.8 Hz), 126.24 (q, J = 3.6 Hz), 139.78, 141.14, 153.35.
^{19}F NMR (471 MHz, $CDCl_3$) δ −63.0 (s, 3F).
HRMS (ESI): *m/z* calculated for $C_{13}H_{13}F_3NO_2$ $[M–H]^-$: 272.0904, found: 272.0908.

3-methylbut-2-en-1-yl (4-cyanophenyl)carbamate (1e)

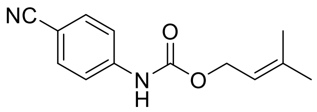

According to the general procedure A, **1e** was prepared from 4-cyanophenyl isocyanate (432 mg, 3.0 mmol), prenol (258 mg, 3.0 mmol) and Et_3N (910 mg, 3.0 mmol). The crude product was purified by column chromatography (silica gel, hexane/EtOAc = 3:1) to provide **1e** (500 mg, 72%) as a colorless solid. NMR spectra of the obtained product were consistent with the reported one.[3]

^1H NMR (400 MHz, $CDCl_3$) δ 1.76 (s, 3H), 1.79 (s, 3H), 4.69 (d, J = 7.3 Hz, 2H), 5.38 (t, J = 7.6 Hz, 1H), 6.79 (s, 1H), 7.50 (d, J = 8.9 Hz, 2H), 7.59 (d, J = 8.8 Hz, 2H).

^{13}C NMR (125 MHz, $CDCl_3$) δ 18.03, 25.75, 62.53, 106.09, 118.11, 118.20, 118.91, 133.30, 140.06, 142.19, 152.94.

3-methylbut-2-en-1-yl (4-chlorophenyl)carbamate (1f)

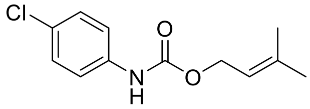

According to the general procedure A, **1f** was prepared from 4-chlorophenyl isocyanate (0.77 g, 5.0 mmol), prenol (0.43 g, 5.0 mmol) and Et_3N (1.5 g, 15 mmol). The crude product was purified by column chromatography (silica gel, hexane/EtOAc = 7:1) to provide **1f** (1.04 g, 87%) as a colorless solid.

TLC: R_f = 0.42 (hexane/EtOAc = 5:1).

^1H NMR (400 MHz, $CDCl_3$) δ 1.75 (s, 3H), 1.78 (s, 3H), 4.66 (d, J = 7.2 Hz, 2H), 5.39 (tt, J = 7.2, 1.4 Hz, 1H), 6.56 (s, 1H), 7.25-7.27 (m, 2H), 7.32 (d, J = 8.6 Hz, 2H).

^{13}C NMR (125 MHz, $CDCl_3$) δ 18.02, 25.75, 62.16, 118.49, 119.82, 128.29, 128.97, 136.58, 139.57, 153.48.

HRMS (ESI): *m/z* calculated for $C_{12}H_{14}ClNO_2Na$ $[M+Na]^+$: 262.0605, found: 262.0610.

3-methylbut-2-en-1-yl (4-bromophenyl)carbamate (1g)

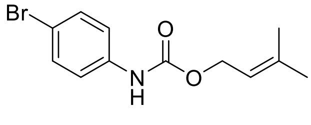

According to the general procedure A, **1g** was prepared from 4-bromophenyl isocyanate (0.99 g, 5.0 mmol), prenol (0.43 g, 5.0 mmol) and Et_3N (1.5 g, 15 mmol). The crude product was purified by column chromatography (silica gel, hexane/EtOAc = 7:1) followed by recrystallization from hexane to provide **1g** (1.02 g, 72%) as a colorless solid.

TLC: R_f = 0.41 (hexane/EtOAc = 5:1).

^{1}H NMR (400 MHz, $CDCl_3$) δ 1.75 (s, 3H), 1.78 (s, 3H), 4.66 (d, J = 7.2 Hz, 2H), 5.39 (tt, J = 7.2, 1.4 Hz, 1H), 6.57 (s, 1H), 7.27 (d, J = 8.6 Hz, 2H), 7.38-7.72 (m, 2H).

^{13}C NMR (125 MHz, $CDCl_3$) δ 18.03, 25.76, 62.18, 115.78, 118.47, 120.13, 131.92, 137.09, 139.60, 153.42.

HRMS (ESI): *m/z* calculated for $C_{12}H_{13}BrNO_2$ [M–H]$^-$: 282.0135, found: 282.0138.

3-methylbut-2-en-1-yl (4-iodophenyl)carbamate (1h)

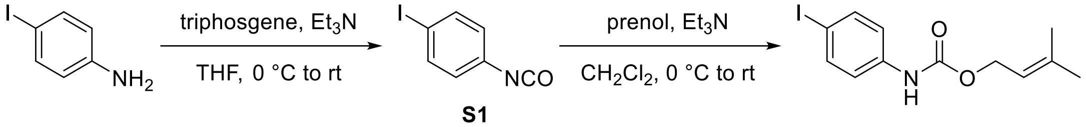

To a solution of triphosgene (1.49 g, 5.0 mmol) in THF (10 mL), a solution of 4-iodoaniline (1.10 g, 5.0 mmol) in THF (40 mL) was added dropwise at 0 °C. Then, triethylamine (1.06 g, 10.5 mmol) was added dropwise at 0 °C. The solution was warmed to room temperature and stirred for 2 hours. Then, volatiles were removed under reduced pressure to afford crude 4-iodophenyl isocyanate (**S1**) which was used for the next step without further purification.

The crude **S1** was dissolved in CH_3CN (80 mL) and triethylamine (1.06 g, 10.5 mmol) was added dropwise to the solution at 0 °C. Then, prenol (517 mg, 6.0 mmol) was added dropwise to the solution at 0 °C. The reaction mixture was warmed to room temperature and stirred for 18 hours. Volatiles were removed under reduced pressure and recrystallization from hexane/EtOAc afforded **1h** (343 mg, 21%) as a colorless solid.

TLC: R_f = 0.84 (hexane/EtOAc = 1:1).

^{1}H NMR (400 MHz, $CDCl_3$) δ 1.75 (s, 3H), 1.78 (s, 3H), 4.66 (d, J = 7.2 Hz, 2H), 5.38 (tt, J = 7.2, 1.4 Hz, 1H), 6.55 (s, 1H), 7.16 (d, J = 8.6 Hz, 2H), 7.59 (d, J = 8.6 Hz, 2H).

^{13}C NMR (125 MHz, $CDCl_3$) δ 18.03, 25.77, 62.19, 86.13, 118.47, 120.48, 137.82, 137.86, 139.64, 153.35.

HRMS (ESI): *m/z* calculated for $C_{12}H_{13}INO_2$ [M–H]$^-$: 329.9996, found: 330.0002.

3-methylbut-2-en-1-yl (4-acetylphenyl)carbamate (1i)

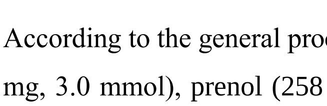

According to the general procedure A, **1i** was prepared from 1-(4-isocyanatophenyl)ethan-1-one (483 mg, 3.0 mmol), prenol (258 mg, 3.0 mmol) and Et_3N (910 mg, 9.0 mmol). The crude product was purified by column chromatography (silica gel, hexane/EtOAc = 2:1) to provide **1i** (620 mg, 84%) as a colorless solid.

TLC: R_f = 0.14 (hexane/EtOAc = 5:1).

^{1}H NMR (500 MHz, $CDCl_3$) δ 1.74 (s, 3H), 1.77 (s, 3H), 2.57 (s, 3H), 4.68 (d, J = 7.4 Hz, 2H), 5.37 (tt, J = 7.4, 1.6 Hz, 1H), 7.29 (s, 1H), 7.51 (d, J = 8.6 Hz, 2H), 7.92 (d, J = 8.9 Hz, 2H).
^{13}C NMR (125 MHz, $CDCl_3$) δ 17.96, 25.67, 26.26, 62.25, 117.54, 118.34, 129.78, 131.90, 139.57, 142.68, 153.22, 197.02.
HRMS (ESI): m/z calculated for $C_{14}H_{17}NO_3Na$ $[M+Na]^+$: 270.1101, found: 270.1106.

3-methylbut-2-en-1-yl (4-(benzyloxy)phenyl)carbamate (1j)

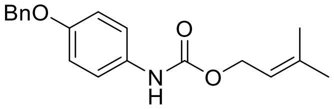

According to the general procedure A, **1j** was prepared from 1-(benzyloxy)-4-isocyanatobenzene (680 mg, 3.0 mmol), prenol (258 mg, 3.0 mmol) and Et_3N (910 mg, 9.0 mmol). The crude product was purified by column chromatography (silica gel, hexane/EtOAc = 5:1) to provide **1j** (710 mg, 76%) as a colorless solid.
TLC: R_f = 0.55 (hexane/EtOAc = 2:1).
^{1}H NMR (400 MHz, $CDCl_3$) δ 1.74 (s, 3H), 1.78 (s, 3H), 4.65 (d, J = 7.2 Hz, 2H), 5.04 (s, 2H), 5.39 (tt, J = 7.2, 1.4 Hz, 1H), 6.43 (s, 1H), 6.92 (d, J = 6.3 Hz, 2H), 7.26-7.28 (m, 2H), 7.30-7.34 (m, 1H), 7.36-7.43 (m, 4H).
^{13}C NMR (125 MHz, $CDCl_3$) δ 18.03, 25.77, 61.92, 70.32, 115.32, 118.79, 120.56, 127.43, 127.91, 128.54, 131.27, 137.02, 139.22, 153.92, 155.07.
HRMS (ESI): m/z calculated for $C_{19}H_{21}NO_3Na$ $[M+Na]^+$: 334.1414, found: 334.1419.

3-methylbut-2-en-1-yl benzylcarbamate (1k)

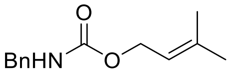

1k was prepared according to the reported procedure.[4] NMR spectra of the obtained product were consistent with the reported one.[4]
^{1}H NMR (400 MHz, $CDCl_3$) δ 1.72 (s, 3H), 1.76 (s, 3H), 4.37 (d, J = 5.4 Hz, 2H), 4.60 (d, J = 7.2 Hz, 2H), 4.95 (s, 1H), 5.36 (tt, J = 7.2, 1.4 Hz, 1H), 7.25-7.29 (m, 3H), 7.32-7.35 (m, 2H).
^{13}C NMR (125 MHz, $CDCl_3$) δ 17.93, 25.68, 44.50, 61.80, 119.07, 127.34, 127.41, 128.55, 138.54, 138.61, 156.64.

3-methylbut-2-en-1-yl 4-oxopiperidine-1-carboxylate (1l)

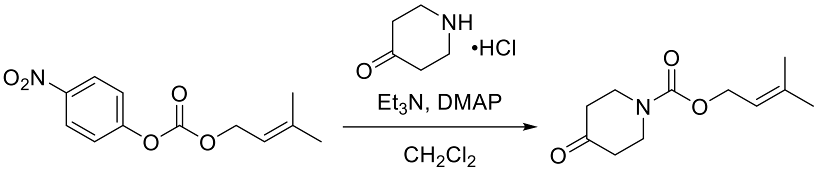

1l was prepared using the reported procedure.[4] To a stirred solution of prenyl 4-nitrophenyl carbonate (829 mg, 3.3 mmol, prepared according to the reported procedure[4]), 4-piperidinone hydrochloride (407 mg, 3.0 mmol) and DMAP (36.7 mg, 0.30 mmol) in CH_2Cl_2 at 0 °C, triethylamine (304 mg, 3.0 mmol) was added. The reaction mixture was warmed to room temperature and stirred for 6.5 hours. The mixture was then diluted with diethyl ether and washed with 1 M aq. HCl (x 1), sat. aq. Na_2CO_3 (x 2), water (x 2) and brine (x 1). The organic layer was dried over Na_2SO_4 and concentrated under reduced pressure. The crude product was purified by column chromatography (silica gel, hexane/EtOAc = 3:1 to 1:1) to provide **1l** (433 mg, 68%) as a colorless oil.

TLC: R_f = 0.40 (hexane/EtOAc = 1:1).

^{1}H NMR (500 MHz, $CDCl_3$) δ 1.66 (s, 3H), 1.70 (s, 3H), 2.39 (t, J = 5.5 Hz, 4H), 3.71 (t, J = 5.5 Hz, 4H), 4.56 (d, J = 7.5 Hz), 5.30-5.31 (m, 1H).

^{13}C NMR (125 MHz, $CDCl_3$) δ 17.89, 25.60, 40.92, 42.93, 62.57, 118.93, 138.49, 155.23, 207.20.

HRMS (APCI): m/z calculated for $C_{11}H_{18}NO_3$ $[M+H]^+$: 212.1281, found: 212.1280.

4-methyl-*N*-(3-methylbut-2-en-1-yl)benzenesulfonamide (1m)

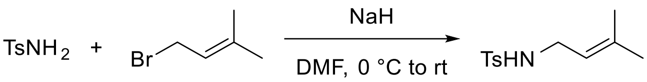

A solution of *p*-toluenesulfoneamide (856 mg, 5.0 mmol) in DMF (15 mL) was added to NaH (60% w/w in mineral oil, 300 mg, 7.5 mmol) at 0 °C. Then, prenyl bromide (749 mg, 5.0 mmol) was added at 0 °C. The mixture was stirred overnight and was quenched with water. The organic material was extracted with Et_2O (x 3) and the combined organic layer was concentrated under reduced pressure. Purification by column chromatography (silica gel, hexane/EtOAc = 10:1) afforded 4-methyl-*N*-(3-methylbut-2-en-1-yl)benzenesulfonamide (**1m**) (261 mg, 22%) as a colorless solid. NMR spectra of the obtained product were consistent with the reported one.[5]

^{1}H NMR (400 MHz, $CDCl_3$) δ 1.54 (s, 3H), 1.64 (s, 3H), 2.43 (s, 3H), 3.54 (t, J = 6.5 Hz, 2H), 4.14 (m, 1H), 5.06 (t, J = 7.2 Hz, 1H), 7.31 (d, J = 7.9 Hz, 2H), 7.75 (d, J = 8.3 Hz, 2H).

^{13}C NMR (125 MHz, $CDCl_3$) δ 17.68, 21.43, 25.46, 40.96, 118.85, 127.12, 129.54, 136.99, 137.36, 143.22.

benzyl (3-methylbut-2-en-1-yl) carbonate (1n)

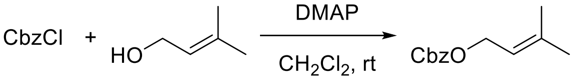

To a solution of prenol (431 mg, 5.0 mmol) and DMAP (3.05 g, 25 mmol) in CH_2Cl_2 (38 mL), benzyl chloroformate (4.26 g, 25 mmol) was added. The mixture was stirred at room temperature for 3 hours. Then, sat. aq. $NaHCO_3$ was added, and organic material was extracted with CH_2Cl_2 (x 3). The combined organic layer was washed with 1M aq. HCl (x 1), sat. aq. $NaHCO_3$ (x 1) and brine (x 1). The organic layer was dried over Na_2SO_4 and concentrated under reduced pressure. Purification by column chromatography (silica gel, hexane/CH_2Cl_2 = 2:1) afforded **1n** (588 mg, 53%) as a colorless oil.

TLC: R_f = 0.28 (hexane/CH_2Cl_2 = 3:2).

^{1}H NMR (500 MHz, $CDCl_3$) δ 1.71 (s, 3H), 1.75 (s, 3H), 4.64 (d, J = 7.4 Hz, 2H), 5.15 (s, 2H), 5.38-5.40 (m, 1H), 7.32-7.36 (m, 5H).

^{13}C NMR (125 MHz, $CDCl_3$) δ 17.98, 25.67, 64.69, 69.35, 117.96, 128.18, 128.35, 128.47, 135.33, 139.97, 155.16.

HRMS (ESI): *m/z* calculated for $C_{13}H_{16}O_3Na$ $[M+Na]^+$: 243.0992, found: 243.0994.

3-methylbut-2-en-1-yl (2-chloroethyl)carbamate (1o)

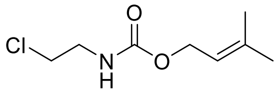

According to the general procedure A, **1o** was prepared from 2-chloroethyl isocyanate (523 mg, 5.0 mmol), prenol (431 mg, 5.0 mmol) and Et_3N (1.52 g, 15 mmol). The crude product was purified by column chromatography (silica gel, hexane/EtOAc = 7:1 to 2:1) to provide **1o** (813 mg, 85%) as a colorless solid.

TLC: R_f = 0.38 (hexane/EtOAc = 3:1).

^{1}H NMR (500 MHz, $CDCl_3$) δ 1.72 (s, 3H), 1.76 (s, 3H), 3.52 (q, J = 5.5 Hz, 2H), 3.61 (t, J = 5.4 Hz, 2H), 4.58 (d, J = 6.9 Hz, 2H), 5.10 (s, 1H), 5.33-5.36 (m, 1H).

^{13}C NMR (125 MHz, $CDCl_3$) δ 17.81, 25.57, 42.64, 43.84, 61.77, 118.81, 138.63, 156.41.

HRMS (ESI): *m/z* calculated for $C_8H_{15}ClNO_2$ $[M+Na]^+$: 192.0786, found: 192.0790.

3-methylbut-3-en-1-yl phenylcarbamate (1p)

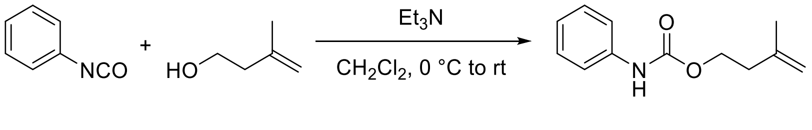

To a solution of the phenyl isocyanate (596 mg, 5.0 mmol) in CH_2Cl_2 (5 mL), triethylamine (1.5 g, 15 mmol) and 3-methyl-3-butene-1-ol (431 mg, 5.0 mmol) were sequentially added at 0 °C. The mixture was allowed to warm to room temperature and was stirred for 24 hours. The mixture was diluted with CH_2Cl_2 and washed with 1M aq. HCl (x 3), water (x 1), and brine (x 1). The organic layer was dried over Na_2SO_4 and was concentrated under reduced pressure. The obtained crude was purified by

column chromatography (silica gel, hexane/EtOAc = 7:1) to afford 3-methylbut-3-en-1-yl phenylcarbamate (**1p**) (728 mg, 71%) as a colorless solid.

TLC: R_f = 0.38 (hexane/EtOAc = 5:1).

^{1}H NMR (400 MHz, $CDCl_3$) δ 1.79 (s, 3H), 2.40 (t, J = 6.7 Hz, 2H), 4.29 (t, J = 6.7 Hz, 2H), 4.78-4.79 (m, 1H), 4.83-4.84 (m, 1H), 6.58 (s, 1H), 7.06 (t, J = 7.2 Hz, 1H), 7.30 (t, J = 7.9 Hz, 2H), 7.36-7.39 (m, 2H).

^{13}C NMR (100 MHz, $CDCl_3$) δ 22.37, 36.88, 63.22, 112.25, 118.56, 123.28, 128.92, 137.85, 141.56, 153.55.

HRMS (ESI): *m/z* calculated for $C_{12}H_{15}NO_2Na$ $[M+Na]^+$: 228.0995, found: 228.0998.

benzyl (*E*)-2-methylbut-2-enoate (1q)

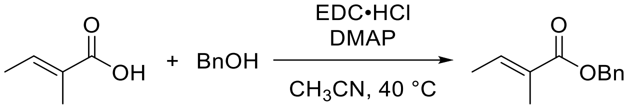

To a solution of tiglic acid (501 mg, 5.0 mmol), EDC•HCl (1.15 g, 6.0 mmol) and DMAP (1.47 g, 12 mmol) in CH_3CN (63 mL), benzyl alcohol (649 mg, 6.0 mmol) was added. The solution was warmed to 40 °C and stirred for 2.5 hours. Volatiles were removed under reduced pressure, and the residue was dissolved in Et_2O. The solution was washed with 3 M aq. HCl (x 3), sat. aq. $NaHCO_3$ (x 2) and brine (x 1). The organic layer was dried over Na_2SO_4 and concentrated under reduced pressure. Purification by column chromatography (silica gel, hexane/CH_2Cl_2 = 1:2) afforded **1q** (777 mg, 82%) as a colorless oil. NMR spectra of the obtained product were consistent with the reported one.[6]

^{1}H NMR (500 MHz, $CDCl_3$) δ 1.78 (ddd, J = 7.0, 1.5, 1.0 Hz, 3H), 1.86 (t, J = 1.4 Hz, 3H), 5.17 (s, 2H), 6.89-6.93 (m, 1H), 7.28-7.38 (m, 5H).

^{13}C NMR (125 MHz, $CDCl_3$) δ 12.00, 14.28, 66.06, 127.90, 127.94, 128.42, 128.44, 136.38, 137.52, 167.78.

1-(*tert*-butyl)-4-methylenecyclohexane (1r)

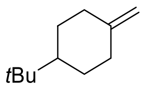

1r was prepared according to the reported procedure.[7] NMR spectra of the obtained product were consistent with the reported one.[7]

^{1}H NMR (500 MHz, $CDCl_3$) δ 0.86 (s, 9H), 1.01-1.09 (m, 2H), 1.12-1.18 (m, 1H), 1.84-1.88 (m, 2H), 1.99 (tt, J = 12.7, 1.9 Hz, 2H), 2.31-2.35 (m, 2H), 4.58 (t, J = 1.8 Hz, 2H).

^{13}C NMR (125 MHz, $CDCl_3$) δ 27.63, 28.96, 32.42, 35.30, 47.88, 106.06, 150.27.

***tert*-butyl((3-methylbut-2-en-1-yl)oxy)diphenylsilane (1t)**

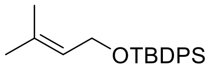

1t was prepared according to the reported procedure.[8] NMR spectra of the obtained product were consistent with the reported one.[8]

^{1}H NMR (400 MHz, $CDCl_3$) δ 1.04 (s, 9H), 1.45 (s, 3H), 1.69 (d, J = 0.9 Hz, 3H), 4.19 (d, J = 6.3 Hz, 2H), 5.35-5.40 (m, 1H), 7.35-7.44 (m, 6H), 7.67-7.71 (m, 4H).

^{13}C NMR (100 MHz, $CDCl_3$) δ 17.92, 19.16, 25.70, 26.83, 61.11, 124.17, 127.56, 129.47, 133.80, 134.04, 135.60.

(*S*)-(((3,7-dimethyloct-6-en-1-yl)oxy)methyl)benzene (1u)

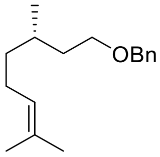

1u was prepared according to the reported procedure.[9] NMR spectra of the obtained product were consistent with the reported one.[9]

^{1}H NMR (400 MHz, $CDCl_3$) δ 0.89 (d, J = 6.3 Hz, 3H), 1.11-1.20 (m, 1H), 1.29-1.47 (m, 2H), 1.52-1.72 (m, 8H), 1.90-2.06 (m, 2H), 3.46-3.55 (m, 2H), 4.50 (s, 2H), 5.07-5.11 (m, 1H), 7.24-7.38 (m, 5H).

^{13}C NMR (125 MHz, $CDCl_3$) δ 17.61, 19.54, 25.45, 25.70, 29.57, 36.71, 37.20, 68.73, 72.89, 124.82, 127.44, 127.59, 128.32, 131.12, 138.69.

Synthesis of 5-methylhex-4-enoic acid (S4)

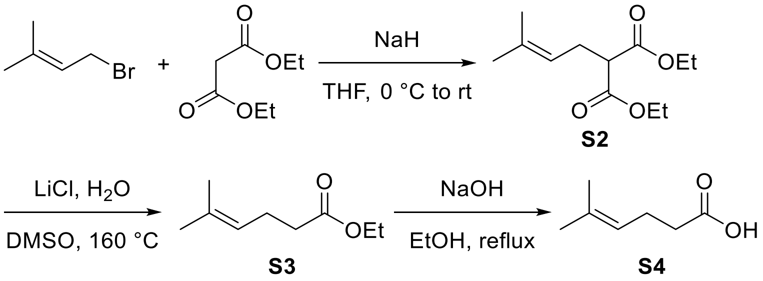

To a stirred suspension of NaH (60% w/w in mineral oil, 0.90 g, 22.5 mmol) in THF (27.5 mL), diethylmalonate (3.60 g, 22.5 mmol) and 1-bromo-3-methyl-2-butene (2.23 g, 15.0 mmol) were sequentially added at 0 °C. The reaction mixture was warmed to room temperature and was stirred overnight. The reaction was quenched with saturated aq. NH_4Cl and diluted with water. Organic material was extracted with EtOAc (x 3) and the combined organic layer was washed with brine (x 1). The organic layer was concentrated under reduced pressure and roughly purified by column

chromatography (silica gel, hexane/EtOAc = 10:1) to afford crude diethyl 2-(3-methylbut-2-en-1-yl)malonate (**S2**) (2.63 g) as a colorless oil.

The crude **S2** (2.63 g) and LiCl (1.48 g, 34.8 mmol) were dissolved in DMSO (18 mL) and water (0.2 mL). The solution was warmed to 160 °C and was stirred for 3 hours. The solution was then cooled to room temperature and crushed ice and brine was added. The organic material was extracted with Et_2O (x 3) and the combined organic layer was washed with water (x 1). The organic layer was dried over Na_2SO_4 and concentrated to afford crude ethyl 5-methylhex-4-enoate (**S3**) (2.88 g) as a colorless oil.

The crude **S3** (1.78 g) and NaOH (1.46 g, 36.5 mmol) were dissolved in EtOH (4 mL) and water (12 mL) and the solution was warmed to reflux and was stirred for 21 hours. The reaction mixture was cooled to room temperature and washed with Et_2O. Then, the reaction mixture was acidified to pH = 1 by adding aq. HCl. Then, organic material was extracted with Et_2O, dried over Na_2SO_4 and concentrated under reduced pressure to afford 5-methylhex-4-enoic acid (**S4**) (1.09 g, 8.6 mmol, ca. 93% for 3 steps) as a colorless oil. NMR spectra of the obtained product were consistent with the reported one.[10]

^{1}H NMR (400 MHz, $CDCl_3$) δ 1.62 (s, 3H), 1.69 (s, 3H), 2.32 (t, J = 6.8 Hz, 2H), 2.36-2.40 (m, 2H), 5.10 (tt, J = 6.9, 1.4 Hz, 1H), 11.35 (s, 1H).

^{13}C NMR (100 MHz, $CDCl_3$) δ 17.62, 23.29, 25.64, 34.27, 122.03, 133.39, 180.12.

5-methyl-*N*-phenylhex-4-enamide (1v)

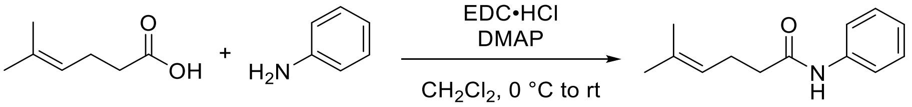

S4 1v

To a solution of EDC•HCl (498 mg, 2.6 mmol) and DMAP (342 mg, 2.8 mmol) in CH_2Cl_2 (5 mL), **S4** (256 mg, 2.0 mmol) and aniline (224 mg, 2.4 mmol) were added at 0 °C. The mixture was warmed to room temperature and was stirred for 24 hours. Then, 1 M aq. HCl was added to the mixture, and organic material was extracted with CH_2Cl_2 (x 2). The organic layer was dried over Na_2SO_4 and was concentrated under reduced pressure. Recrystallization with EtOAc and hexane afforded 5-methyl-*N*-phenylhex-4-enamide (**1v**) (220 mg, 54%) as a colorless solid. NMR spectra of the obtained product were consistent with the reported one.[11]

^{1}H NMR (400 MHz, $CDCl_3$) δ 1.66 (s, 3H), 1.72 (s, 3H), 2.38-2.43 (m, 4H), 5.16-5.20 (m, 1H), 7.10 (t, J = 7.5 Hz, 1H), 7.18 (s, 1H), 7.32 (t, J = 7.9 Hz, 2H), 7.49 (d, J = 7.7 Hz, 2H).

^{13}C NMR (125 MHz, $CDCl_3$) δ 17.77, 24.16, 25.72, 37.74, 119.69, 122.57, 124.14, 128.98, 133.85, 137.96, 170.95.

benzyl (5-methylhex-4-enoyl)-L-glutaminate (1w)

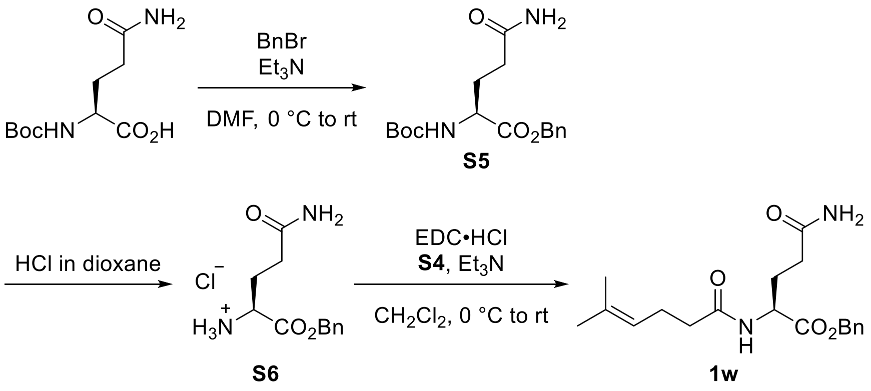

To a solution of *N*-(*tert*-butoxycarbonyl)-L-glutamine (1.23 g, 5.0 mmol) in DMF (13 mL), benzyl bromide (940 mg, 5.5 mmol) and triethylamine (557 mg, 5.5 mmol) were added at 0 °C. The solution was warmed to room temperature and was stirred overnight. Additional benzyl bromide (855 mg, 5.0 mmol) and triethylamine (506 mg, 5.0 mmol) were added at 0 °C. The solution was warmed to room temperature and was stirred overnight. Volatiles were removed under reduced pressure, and the residue was dissolved in EtOAc. The solution was washed with 10% aq. $KHSO_4$ (x 3), sat. aq. $NaHCO_3$ (x 3), and brine (x 1). The organic layer was dried over Na_2SO_4 and concentrated under reduced pressure to afford crude benzyl (*tert*-butoxycarbonyl)-L-glutaminate (**S5**) (1.48 g) as a colorless oil.

The crude **S5** (1.48 g) was dissolved in 4 M HCl-dioxane solution (17 mL) and was stirred for 2 hours at room temperature. The solution was concentrated to afford crude L-glutamine benzyl ester hydrochloride (**S6**) (1.26 g) as a colorless solid.

To a solution of **S4** (291 mg, 2.3 mmol) in CH_2Cl_2 (6 mL), triethylamine (683 mg, 6.8 mmol), **S6** (614 mg, 2.3 mmol) and EDC•HCl (431 mg, 2.3 mmol) were added at 0 °C. The mixture was warmed to room temperature and stirred overnight. The mixture was diluted with water and extracted with CH_2Cl_2 (x 3). The combined organic layer was washed with 1 M aq. HCl (x 1), sat. aq. $NaHCO_3$ (x 1) and brine (x 1). The organic layer was dried over Na_2SO_4 and concentrated under reduced pressure. Recrystallization (hexane/EtOAc) afforded **1w** (150 mg, 19%) as a colorless solid.

TLC: R_f = 0.38 (CH_2Cl_2/MeOH = 10:1).

^{1}H NMR (400 MHz, $CDCl_3$) δ 1.61 (s, 3H), 1.67 (s, 3H), 1.89-1.96 (m, 1H), 2.20-2.32 (m, 7H), 4.60-4.66 (m, 1H), 5.08 (s, 1H), 5.16 (d, J = 12.4 Hz, 1H), 5.20 (d, J = 12.4 Hz, 1H), 6.48-6.50 (m, 2H), 7.33-7.39 (m, 5H).

^{13}C NMR (100 MHz, $CDCl_3$) δ 17.73, 24.05, 25.68, 28.74, 31.62, 36.49, 51.71, 67.48, 122.30, 128.42, 128.62, 128.67, 133.53, 135.01, 171.73, 173.44, 174.70.

HRMS (ESI): *m/z* calculated for $C_{19}H_{26}N_2O_4Na$ $[M+Na]^+$: 369.1785, found: 369.1790.

benzyl (5-methylhex-4-enoyl)-L-serinate (1x)

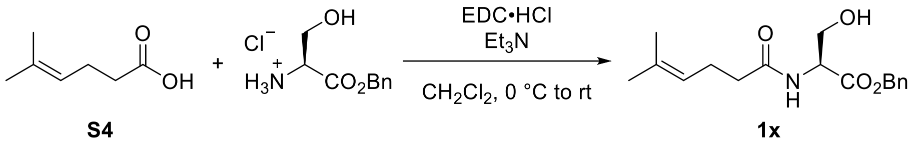

To a solution of **S4** (256 mg, 2.0 mmol) in CH_2Cl_2 (6 mL), triethylamine (607 mg, 6.0 mmol), L-serine benzyl ester hydrochloride (463 mg, 2.0 mmol) and EDC•HCl (383 mg, 2.0 mmol) were added at 0 °C. The mixture was warmed to room temperature and stirred for 18 hours. The mixture was diluted with water and extracted with CH_2Cl_2 (x 3). The combined organic layer was washed with 1M aq. HCl (x 3), sat. aq. $NaHCO_3$ (x 3) and brine (x 1). The organic layer was dried over Na_2SO_4 and concentrated under reduced pressure. Purification by column chromatography (silica gel, hexane/EtOAc = 2:3) followed by recrystallization (hexane/EtOAc) afforded **1x** (367 mg, 60%) as a colorless solid.

TLC: R_f = 0.35 (CH_2Cl_2/EtOAc = 2:1).

^{1}H NMR (400 MHz, $CDCl_3$) δ 1.61 (s, 3H), 1.68 (s, 3H), 2.32-2.28 (m, 4H), 2.69 (s, 1H), 3.93 (ddd, *J* = 28.5, 11.2, 3.4 Hz, 2H), 4.72-4.69 (m, 1H), 5.10-5.08 (m, 1H), 5.21 (s, 2H), 6.57 (d, *J* = 7.2 Hz, 1H), 7.39-7.31 (m, 5H).

^{13}C NMR (100 MHz, $CDCl_3$) δ 17.68, 24.06, 25.64, 36.37, 54.80, 63.47, 67.46, 122.34, 128.11, 128.49, 128.62, 133.48, 135.05, 170.37, 173.40.

HRMS (ESI): *m/z* calculated for $C_{17}H_{23}NO_4Na$ $[M+Na]^+$: 328.1519, found: 328.1524.

benzyl (5-methylhex-4-enoyl)-L-methioninate (1y)

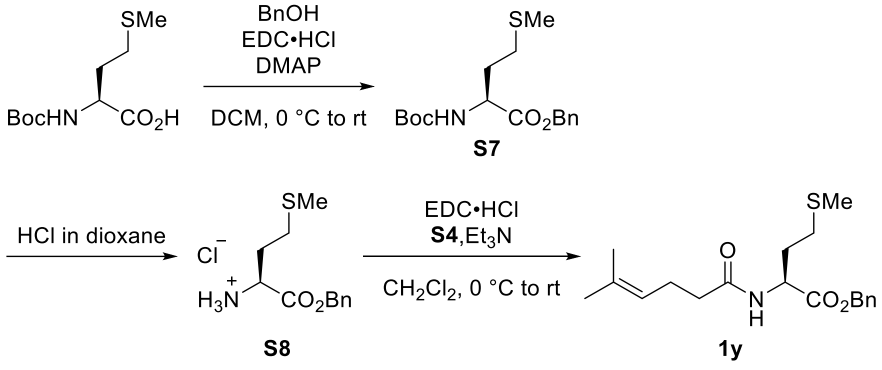

To a solution of *N*-(*tert*-butoxycarbonyl)-L-methionine (2.49 g, 10 mmol), benzyl alcohol (1.22 g, 11 mmol) and DMAP (610 mg, 5.0 mmol) in CH_2Cl_2 (36 mL), EDC•HCl (2.08 g, 11 mmol) was added at 0 °C. The solution was stirred for 2 hours. The solution was then warmed to room temperature and was stirred overnight. Volatiles were removed under reduced pressure, and the residue was dissolved in EtOAc. The solution was washed with sat. aq. $NaHCO_3$ (x 2), H_2O (x 2) and brine (x 1). The organic

layer was dried over Na_2SO_4 and concentrated under reduced pressure to afford crude benzyl (*tert*-butoxycarbonyl)-L-methioninate (**S7**) (3.31 g) as a colorless oil.

The crude **S7** (2.72 g) was dissolved in 4 M HCl-dioxane solution (29 mL) and was stirred for 2 hours at room temperature. The solution was concentrated to afford crude L-methionine benzyl ester hydrochloride (**S8**) (2.07 g) as a colorless solid.

To a solution of **S8** (385 mg, 3.0 mmol) in CH_2Cl_2 (9 mL), triethylamine (911 mg, 9.0 mmol), **S8** (986 mg, 3.0 mmol) and EDC•HCl (575 mg, 3.0 mmol) were added at 0 °C. The mixture was warmed to room temperature and stirred overnight. The mixture was diluted with water and extracted with CH_2Cl_2 (x 3). The combined organic layer was washed with 1 M aq. HCl (x 1), sat. aq. $NaHCO_3$ (x 1) and brine (x 1). The organic layer was dried over Na_2SO_4 and concentrated under reduced pressure. Purification by column chromatography (silica gel, hexane/EtOAc = 4:1 to 3:1) afforded **1y** (707 mg, 67%) as a colorless solid.
TLC: R_f = 0.22 (hexane/EtOAc = 3:1).
^{1}H NMR (400 MHz, $CDCl_3$) δ 1.62 (s, 3H), 1.68 (s, 3H), 1.96-2.02 (m, 1H), 2.03 (s, 3H), 2.14-2.47 (m, 7H), 4.76 (td, J = 7.4, 5.5 Hz, 1H), 5.07-5.09 (m, 1H), 5.16 (d, J = 12.4 Hz, 1H), 5.21 (d, J = 12.4 Hz, 2H), 6.16 (d, J = 7.6 Hz, 1H), 7.34-7.39 (m, 5H).
^{13}C NMR (125 MHz, $CDCl_3$) δ 15.43, 17.72, 24.10, 25.67, 29.78, 31.81, 36.55, 51.52, 67.33, 122.50, 128.35, 128.56, 128.65, 133.42, 135.15, 171.90, 172.49.
HRMS (ESI): *m/z* calculated for $C_{19}H_{27}NO_3SNa$ $[M+Na]^+$: 372.1604, found: 372.1610.

benzyl (5-methylhex-4-enoyl)glycylglycinate (1z)

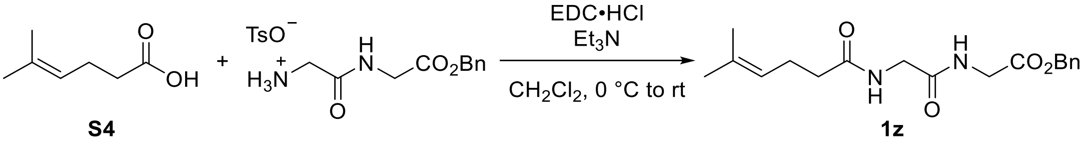

S4 1z

To a solution of **S4** (320 mg, 2.5 mmol) in CH_2Cl_2 (7 mL), triethylamine (759 mg, 7.5 mmol), glycylglycine benzyl ester *p*-toluenesulfonate (986 mg, 2.5 mmol) and EDC•HCl (480 mg, 2.5 mmol) were added at 0 °C. The mixture was warmed to room temperature and stirred for 16 hours. The mixture was diluted with water and extracted with CH_2Cl_2 (x 3). The combined organic layer was washed with 1 M aq. HCl (x 3), sat. aq. $NaHCO_3$ (x 3) and brine (x 1). The organic layer was dried over Na_2SO_4 and concentrated under reduced pressure. Purification by column chromatography (silica gel, CH_2Cl_2/MeOH = 15:1) followed by recrystallization (hexane/EtOAc) afforded **1z** (390 mg, 48%) as a colorless solid.
TLC: R_f = 0.43 (CH_2Cl_2/MeOH = 10:1).

^{1}H NMR (500 MHz, $CDCl_3$) δ 1.61 (s, 3H), 1.68 (s, 3H), 2.24-2.27 (m, 2H), 2.30-2.34 (m, 2H), 3.99 (d, J = 5.2 Hz, 2H), 4.07 (d, J = 5.3 Hz, 2H), 5.09 (t, J = 6.9 Hz, 1H), 5.17 (s, 2H), 6.43-6.44 (m, 1H), 6.86-6.88 (m, 1H), 7.32-7.38 (m, 5H).

^{13}C NMR (125 MHz, $CDCl_3$) δ 17.69, 24.06, 25.66, 36.26, 41.30, 43.16, 67.25, 122.39, 128.35, 128.56, 128.64, 133.43, 135.07, 169.33, 169.41, 173.53.

HRMS (ESI): *m/z* calculated for $C_{18}H_{24}N_2O_4Na$ $[M+Na]^+$: 355.1628, found: 355.1629.

((2*R*,3*S*,4*R*,5*S*)-3,4-dihydroxy-5-(2-methylallyl)tetrahydrofuran-2-yl)methyl acetate (1af)

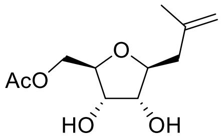

1af was prepared according to the reported procedure.[12] NMR spectra of the obtained product were consistent with the reported one.[12]

^{1}H NMR (500 MHz, $CDCl_3$) δ 1.79 (s, 3H), 2.10 (s, 3H), 2.28 (dd, J = 14.3, 6.3 Hz, 1H), 2.32 (dd, J = 14.0, 7.2 Hz, 1H), 3.13 (s, 1H), 3.37 (s, 1H), 3.83 (d, J = 4.0 Hz, 1H), 3.93-3.98 (m, 3H), 4.12 (dd, J = 11.7, 4.9 Hz, 1H), 4.32 (dd, J = 11.7, 3.2 Hz, 1H), 4.81 (s, 1H), 4.85 (s, 1H).

^{13}C NMR (125 MHz, $CDCl_3$) δ 20.84, 22.91, 41.75, 64.48, 71.80, 74.40, 80.75, 81.91, 112.86, 142.02, 171.30.

(2*R*,3*S*,4*R*,5*S*,6*S*)-2-(acetoxymethyl)-6-(2-methylallyl)tetrahydro-2*H*-pyran-3,4,5-triyl triacetate (1ag)

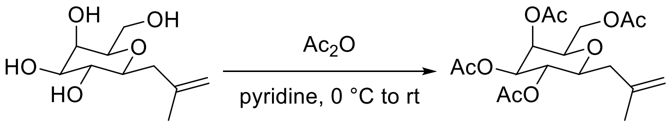

To a solution of **1ah** (prepared according to the reported procedure,[13] 437 mg, 2.0 mmol) in pyridine (10 mL), acetic anhydride (4.08 g, 40 mmol) was added at 0 °C. The solution was warmed to room temperature and stirred for 21 hours. Volatiles were removed under reduced pressure, and the residue was dissolved in CH_2Cl_2. The solution was washed with water (x 1), and brine (x 1). The organic layer was dried over Na_2SO_4 and concentrated under reduced pressure. Purification by column chromatography (silica gel, hexane/EtOAc = 3:1 to 3:2) afforded **1ag** (742 mg, 96%) as a colorless solid.

NMR spectra of the obtained product were consistent with the reported one.[14]

^{1}H NMR (500 MHz, $CDCl_3$) δ 1.76 (s, 3H), 1.98 (s, 3H), 2.03 (s, 3H), 2.04 (s, 3H), 2.16 (s, 3H), 2.21-2.31 (m, 2H), 3.58 (ddd, J = 9.7, 8.0, 4.0 Hz, 1H), 3.86 (td, J = 6.9, 1.1 Hz, 1H), 4.05 (dd, J = 11.5,

6.3 Hz, 1H), 4.14 (dd, J = 11.2, 7.2 Hz, 1H), 4.74 (s, 1H), 4.80 (s, 1H), 5.03 (dd, J = 9.7, 3.4 Hz, 1H), 5.11 (t, J = 10.0 Hz, 1H), 5.42 (dd, J = 3.4, 1.1 Hz, 1H).

^{13}C NMR (125 MHz, $CDCl_3$) δ 20.57, 20.61, 20.67, 20.76, 22.75, 39.76, 61.61, 67.69, 69.47, 72.20, 74.08, 77.03, 112.63, 141.37, 169.74, 170.18, 170.27, 170.38.

(2*R*,3*R*,4*R*,5*R*,6*S*)-2-(hydroxymethyl)-6-(2-methylallyl)tetrahydro-2*H*-pyran-3,4,5-triol (1ah)

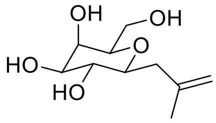

1ah was prepared according to the reported procedure.[13] NMR spectra of the obtained product were consistent with the reported one.[13]

^{1}H NMR (500 MHz, D_2O) δ 1.74 (s, 3H), 2.17 (dd, J = 15.5, 9.2 Hz, 1H), 2.57 (d, J = 14.9 Hz, 1H), 3.41-3.43 (m, 2H), 3.57-3.60 (m, 2H), 3.61-3.71 (m, 2H), 3.92 (d, J = 3.4 Hz, 1H), 4.83 (d, J = 12.0 Hz, 2H).

^{13}C NMR (100 MHz, D_2O) δ 22.36, 39.98, 61.95, 69.79, 71.76, 74.71, 78.40, 79.27, 112.64, 144.66.

2-2. Synthesis of the cobalt complexes

3a was purchased from Sigma-Aldrich and was used as received. **3b** was prepared according to the reported procedure.[15]

Representative procedure: Synthesis of 3f

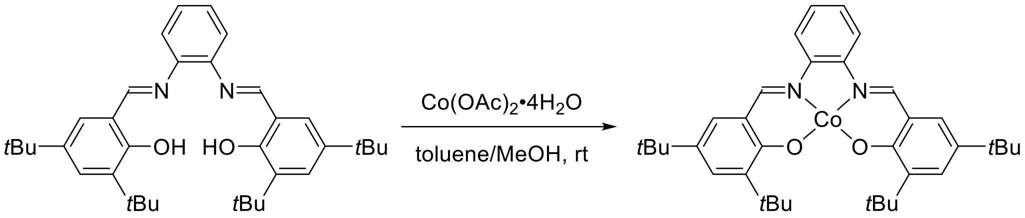

6,6'-((1*E*,1'*E*)-(1,2-phenylenebis(azaneylylidene))bis(methaneylylidene))bis(2,4-di-*tert*-butylphenol) (prepared according to the reported procedure,[16] 541 mg, 1.0 mmol) was dissolved in toluene (10 mL). Then, a solution of $Co(OAc)_2•4H_2O$ (249 mg, 1.0 mmol) in MeOH (20 mL) was added. The mixture was stirred for 2 hours and the precipitate was collected by filtration and washed with cold MeOH. Removal of the residual solvent *in vacuo* afforded **3f** (511 mg, 86%) as a dark red solid. IR spectra of the obtained product were consistent with the reported one.[17]
IR (KBr) 2956, 2865, 1573, 1524, 1466, 1423, 1360, 1258 cm^{-1}.
HRMS (ESI): *m/z* calculated for $C_{36}H_{46}O_2N_2Co$ $[M]^+$: 597.2886, found: 597.2890.

Complexes **3c**, **3d** and **3e** were prepared according to the representative procedure.

2-3. Ascorbic-acid-mediated hydrogenation by cobalt-photoredox dual catalysis

General procedure B

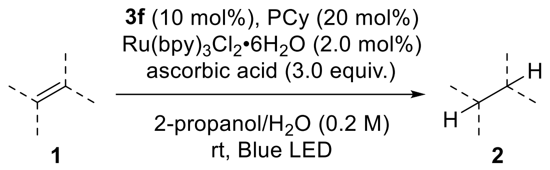

In an argon filled glove box, a flame dried reaction vial was charged with an alkene **1** (0.20 mmol), ascorbic acid (106 mg, 0.60 mmol), **3f** (12.0 mg, 20 μmol), tricyclohexylphosphine (11.2 mg, 40 μmol) and $Ru(bpy)_3Cl_2•6H_2O$ (3.0 mg, 4.0 μmol). The vial was capped and removed from the glove box. A mixed solvent (2-propanol/H_2O = 3:1, 1 mL) was added to the vial via syringe, and the syringe hole was carefully sealed with a vinyl tape. The reaction vial was placed in front of the light source (ca. 3 cm from two blue LED panels) in a cold room (4 °C) so that the temperature of the reaction mixture was kept approximately at 25 °C. After stirring for indicated time, the mixture was cooled in an ice bath and sat. aq. $NaHCO_3$ was added. Organic material was extracted with EtOAc (x 3) and the combined organic layer was washed with brine. The organic layer was concentrated under reduced pressure and purified by column chromatography (silica gel) to afford the hydrogenated product **2**.

isopentyl phenylcarbamate (2a from 1a)

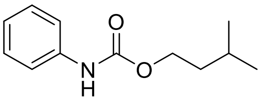

According to the general procedure B (18 h), **2a** was prepared from **1a** (41 mg, 0.20 mmol). The crude was purified by column chromatography (silica gel, hexane/CH_2Cl_2 = 2:1 to 1:2), and **2a** was isolated as a colorless solid (38 mg, 92%).

TLC: R_f = 0.30 (hexane/CH_2Cl_2 = 1:1).

^{1}H NMR (500 MHz, $CDCl_3$) δ 0.94 (d, J = 6.9 Hz, 6H), 1.56 (dt, J = 6.9, 6.9 Hz, 2H), 1.68-1.76 (m, 1H), 4.19 (t, J = 6.9 Hz, 2H), 6.70 (s, 1H), 7.04 (t, J = 7.2 Hz, 1H), 7.25-7.30 (m, 2H), 7.38 (d, J = 7.4 Hz, 2H).

^{13}C NMR (125 MHz, $CDCl_3$) δ 22.43, 24.93, 37.59, 63.87, 118.57, 123.26, 128.97, 137.96, 153.71.

HRMS (ESI): *m/z* calculated for $C_{12}H_{17}O_2NNa$ $[M+Na]^+$: 230.1152, found: 230.1151.

isopentyl *p*-tolylcarbamate (2b)

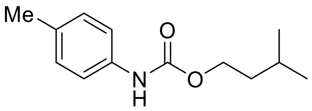

According to the general procedure B (18 h), **2b** was prepared from **1b** (44 mg, 0.20 mmol). The crude was purified by column chromatography (silica gel, hexane/CH_2Cl_2 = 1:1 to 2:1), and **2b** was isolated as a colorless solid (41 mg, 92%).

TLC: R_f = 0.33 (hexane/CH_2Cl_2 = 1:2).

^{1}H NMR (400 MHz, $CDCl_3$) δ 0.94 (d, J = 6.8 Hz, 6H), 1.55 (dt, J = 6.8, 6.8 Hz, 2H), 1.67-1.77 (m, 1H), 2.30 (s, 3H), 4.18 (t, J = 6.8 Hz, 2H), 6.58 (s, 1H), 7.09 (d, J = 8.0 Hz, 2H), 7.26 (d, J = 6.8 Hz, 2H).

^{13}C NMR (125 MHz, $CDCl_3$) δ 20.68, 22.44, 24.94, 37.62, 63.76, 118.69, 129.45, 132.80, 135.37, 153.82.

HRMS (ESI): *m/z* calculated for $C_{13}H_{19}NO_2Na$ $[M+Na]^+$: 244.1308, found: 244.1307.

isopentyl (4-methoxyphenyl)carbamate (2c)

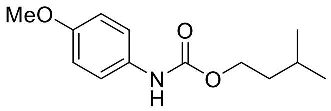

According to the general procedure B (18 h), **2c** was prepared from **1c** (47 mg, 0.20 mmol). The crude was purified by column chromatography (silica gel, hexane/CH_2Cl_2 = 1:1 to 1:9), and **2c** was isolated as a colorless solid (43 mg, 91%).

TLC: R_f = 0.40 (hexane/CH_2Cl_2 = 1:9).

^{1}H NMR (400 MHz, $CDCl_3$) δ 0.93 (d, J = 6.3 Hz, 6H), 1.55 (dt, J = 7.2, 7.2 Hz, 2H), 1.66-1.76 (m, 1H), 3.77 (s, 3H), 4.17 (t, J = 6.8 Hz, 2H), 6.61 (s, 1H), 6.82-6.86 (m, 2H), 7.28 (d, J = 7.7 Hz, 2H).

^{13}C NMR (100 MHz, $CDCl_3$) δ 22.43, 24.91, 37.59, 55.41, 63.72, 114.13, 120.48, 131.02, 154.06, 155.78.

HRMS (ESI): *m/z* calculated for $C_{13}H_{19}NO_3Na$ $[M+Na]^+$: 260.1257, found: 260.1262.

isopentyl (4-(trifluoromethyl)phenyl)carbamate (2d)

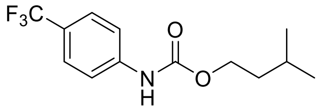

According to the general procedure B (18 h), **2d** was prepared from **1d** (55 mg, 0.20 mmol). The crude was purified by column chromatography (silica gel, hexane/CH_2Cl_2 = 1:2 to 2:1), and **2d** was isolated as a colorless solid (48 mg, 86%).

TLC: R_f = 0.30 (hexane/CH_2Cl_2 = 1:1).

^{1}H NMR (400 MHz, $CDCl_3$) δ 0.94 (d, J = 6.3 Hz, 6H), 1.57 (dt, J = 6.8, 6.8 Hz, 2H), 1.67-1.77 (m, 1H), 4.22 (t, J = 6.8 Hz, 2H), 6.89 (s, 1H), 7.49-7.56 (m, 4H).

^{13}C NMR (100 MHz, $CDCl_3$) δ 22.40, 24.93, 37.50, 64.30, 117.99, 124.14 (q, J = 271.5 Hz), 125.07 (q, J = 32.6 Hz), 126.28 (q, J = 3.8 Hz), 141.14, 153.39.
^{19}F NMR (471 MHz, $CDCl_3$) δ −63.00 (s, 3F).
HRMS (ESI): *m/z* calculated for $C_{13}H_{15}F_3NO_2$ $[M–H]^-$: 274.1060, found: 274.1060.

isopentyl (4-cyanophenyl)carbamate (2e)

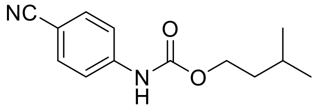

According to the general procedure B (18 h), **2e** was prepared from **1e** (46 mg, 0.20 mmol). The crude was purified by column chromatography (silica gel, hexane/CH_2Cl_2 = 1:3 to 0:1), and **2e** was isolated as a colorless solid (41 mg, 87%).
TLC: R_f = 0.36 (CH_2Cl_2).
^1H NMR (400 MHz, $CDCl_3$) δ 0.94 (d, J = 6.8 Hz, 6H), 1.56 (dt, J = 6.8, 6.8 Hz, 2H), 1.67-1.77 (m, 1H), 4.22 (t, J = 6.8 Hz, 2H), 7.15 (s, 1H), 7.53-7.60 (m, 4H).
^{13}C NMR (100 MHz, $CDCl_3$) δ 22.35, 24.86, 37.40, 64.41, 105.88, 118.19, 118.93, 133.24, 142.32, 153.10.
HRMS (ESI): *m/z* calculated for $C_{13}H_{16}N_2O_2Na$ $[M+Na]^+$: 255.1104, found: 255.1106.

isopentyl (4-chlorophenyl)carbamate (2f)

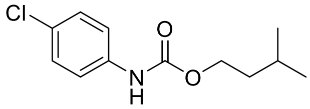

According to the modified general procedure B (18 h; 2-propanol/DMF/H_2O = 3:3:2, 1 mL as a solvent and at 40 °C), **2f** was prepared from **1f** (48 mg, 0.20 mmol). The crude was purified by column chromatography (silica gel, hexane/CH_2Cl_2 = 1:3), and **2f** was isolated as a colorless solid (37 mg, 76%).
TLC: R_f = 0.26 (hexane/CH_2Cl_2 = 1:3).
^1H NMR (500 MHz, $CDCl_3$) δ 0.94 (d, J = 6.9 Hz, 6H), 1.53-1.57 (m, 2H), 1.67-1.75 (m, 1H), 4.19 (t, J = 6.6 Hz, 2H), 6.73 (s, 1H), 7.24-7.26 (m, 2H), 7.33 (d, J = 8.0 Hz, 2H).
^{13}C NMR (125 MHz, $CDCl_3$) δ 22.42, 24.92, 37.54, 64.06, 119.76, 128.24, 128.96, 136.57, 153.56.
HRMS (ESI): *m/z* calculated for $C_{12}H_{16}ClNO_2Na$ $[M+Na]^+$: 264.0762, found: 264.0763.

isopentyl (4-bromophenyl)carbamate (2g)

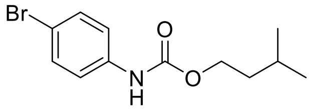

According to the general procedure B (40 h), **2g** was prepared from **1g** (49 mg, 0.20 mmol). The crude was purified by column chromatography (silica gel, hexane/CH_2Cl_2 = 1:2 to 1:3), and **2g** was isolated as a colorless solid (45 mg, 78%).

TLC: R_f = 0.33 (hexane/CH_2Cl_2 = 1:3).

^{1}H NMR (400 MHz, $CDCl_3$) δ 0.94 (d, J = 6.8 Hz, 6H), 1.55 (dt, J = 7.2, 7.2 Hz, 2H), 1.66-1.77 (m, 1H), 4.19 (t, J = 7.0 Hz, 2H), 6.69 (s, 1H), 7.26-7.29 (m, 2H), 7.38-7.42 (m, 2H).

^{13}C NMR (125 MHz, $CDCl_3$) δ 22.43, 24.92, 37.53, 64.09, 115.75, 120.09, 131.90, 137.07, 153.50.

HRMS (ESI): *m/z* calculated for $C_{12}H_{15}BrNO_2$ [M–H]$^-$: 284.0292, found: 284.0295.

isopentyl (4-iodophenyl)carbamate (2h)

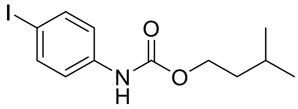

According to the general procedure B (40 h), **2h** was prepared from **1h** (66 mg, 0.20 mmol). The crude was purified by column chromatography (silica gel, toluene), and **2h** was isolated as a colorless solid (47 mg, 71%).

TLC: R_f = 0.30 (toluene).

^{1}H NMR (400 MHz, $CDCl_3$) δ 0.93 (d, J = 6.3 Hz, 6H), 1.52-1.58 (m, 2H), 1.66-1.74 (m, 1H), 4.18 (t, J = 7.0 Hz, 2H), 6.70 (s, 1H), 7.17 (d, J = 8.6 Hz, 2H), 7.56-7.60 (m, 2H)..

^{13}C NMR (125 MHz, $CDCl_3$) δ 22.43, 24.92, 37.52, 64.09, 86.11, 120.45, 137.80, 137.84, 153.44.

HRMS (ESI): *m/z* calculated for $C_{12}H_{15}INO_2$ [M–H]$^-$: 332.0153, found: 332.0158.

isopentyl (4-acetylphenyl)carbamate (2i)

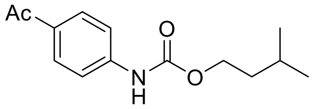

According to the modified general procedure B (18 h; at 40 °C), **2i** was prepared from **1i** (49 mg, 0.20 mmol). The crude was purified by column chromatography (silica gel, hexane/Et_2O = 1:1 to 3:2), and **2i** was isolated as a colorless solid (33 mg, 67%).

TLC: R_f = 0.38 (hexane/Et_2O = 2:3).

^{1}H NMR (500 MHz, $CDCl_3$) δ 0.94 (d, J = 6.3 Hz, 6H), 1.56 (dt, J = 7.4, 7.4 Hz, 2H), 1.68-1.76 (m, 1H), 2.58 (s, 3H), 4.22 (t, J = 6.9 Hz, 2H), 7.22 (s, 1H), 7.52 (d, J = 8.6 Hz, 2H), 7.92-7.94 (m, 2H).

^{13}C NMR (125 MHz, $CDCl_3$) δ 22.38, 24.86, 26.33, 37.45, 64.20, 117.51, 129.81, 131.90, 142.64, 153.27, 197.04.
HRMS (ESI): *m/z* calculated for $C_{14}H_{19}NO_3Na$ $[M+Na]^+$: 272.1257, found: 272.1262.

isopentyl (4-(benzyloxy)phenyl)carbamate (2j)

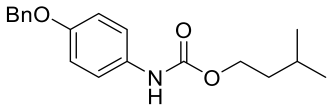

According to the modified general procedure B (40 h; 2-propanol/DMF/H_2O = 3:3:2, 1 mL as a solvent and at 40 °C), **2j** was prepared from **1j** (62 mg, 0.20 mmol). The crude was purified by column chromatography (silica gel, hexane/EtOAc = 4:1), and **2j** was isolated as a colorless solid (49 mg, 78%).
TLC: R_f = 0.29 (hexane/EtOAc = 4:1).
^1H NMR (400 MHz, $CDCl_3$) δ 0.93 (d, *J* = 6.7 Hz, 6H), 1.52-1.56 (m, 2H), 1.66-1.76 (m, 1H), 4.17 (t, *J* = 7.0 Hz, 2H), 5.01 (s, 2H), 6.59 (s, 1H), 6.88-6.92 (m, 2H), 7.23-7.32 (m, 3H), 7.34-7.42 (m, 4H).
^{13}C NMR (100 MHz, $CDCl_3$) δ 22.44, 24.90, 37.59, 63.73, 70.22, 115.20, 120.43, 127.40, 127.87, 128.49, 131.26, 136.93, 154.02, 154.95.
HRMS (ESI): *m/z* calculated for $C_{19}H_{23}NO_3Na$ $[M+Na]^+$: 336.1570, found: 336.1571.

isopentyl benzylcarbamate (2k)

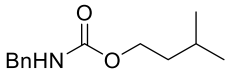

According to the general procedure B (18 h), **2k** was prepared from **1k** (44 mg, 0.20 mmol). The crude was purified by column chromatography (silica gel, hexane/Et_2O = 3:1 to 5:2), and **2k** was isolated as a pale yellow oil (39 mg, 87%).
TLC: R_f = 0.28 (hexane/Et_2O = 2:1).
^1H NMR (400 MHz, $CDCl_3$) δ 0.91 (d, *J* = 6.7 Hz, 6H), 1.50 (dt, *J* = 6.9, 6.9 Hz, 2H), 1.65-1.70 (m, 1H), 4.11 (t, *J* = 6.7 Hz, 2H), 4.35 (d, *J* = 5.4 Hz, 2H), 5.06 (s, 1H), 7.24-7.34 (m, 5H).
^{13}C NMR (100 MHz, $CDCl_3$) δ 22.44, 24.92, 37.68, 44.95, 63.65, 127.36, 127.44, 128.56, 138.58, 156.75.
HRMS (APCI): *m/z* calculated for $C_{13}H_{20}NO_2$ $[M+H]^+$: 222.1489, found: 222.1488.

isopentyl 4-oxopiperidine-1-carboxylate (2l)

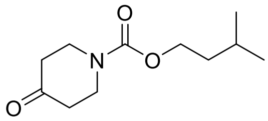

According to the general procedure B (18 h), **2l** was prepared from **1l** (42 mg, 0.20 mmol). The crude was purified by column chromatography (silica gel, hexane/EtOAc = 3:1 to 2:1), and **2l** was isolated as a pale yellow oil (38 mg, 88%).

TLC: R_f = 0.19 (hexane/EtOAc = 3:1).

^{1}H NMR (500 MHz, $CDCl_3$) δ 0.94 (d, J = 6.9 Hz, 6H), 1.56 (dt, J = 6.7, 6.7 Hz, 2H), 1.67-1.75 (m, 1H), 2.46 (t, J = 6.3 Hz, 4H), 3.77 (t, J = 6.0 Hz, 4H), 4.17 (t, J = 6.9 Hz, 2H).

^{13}C NMR (125 MHz, $CDCl_3$) δ 22.46, 25.11, 37.65, 41.07, 43.01, 64.57, 155.38, 207.34.

HRMS (APCI): m/z calculated for $C_{11}H_{20}NO_3$ $[M+H]^+$: 214.1438, found: 214.1437.

***N*-isopentyl-4-methylbenzenesulfonamide (2m)**

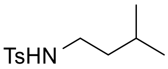

According to the general procedure B (18 h), **2m** was prepared from **1m** (48 mg, 0.20 mmol). The crude was purified by column chromatography (silica gel, CH_2Cl_2), and **2m** was isolated as a colorless solid (39 mg, 80%).

TLC: R_f = 0.24 (CH_2Cl_2).

NMR spectra of the obtained product were consistent with the reported one.[18]

^{1}H NMR (400 MHz, $CDCl_3$) δ 0.82 (d, J = 6.7 Hz, 6H), 1.31-1.36 (m, 2H), 1.52-1.63 (m, 1H), 2.43 (s, 3H), 2.90-2.96 (m, 2H), 4.78 (t, J = 6.1 Hz, 1H), 7.31 (d, J = 8.1 Hz, 2H), 7.75-7.78 (m, 2H).

^{13}C NMR (100 MHz, $CDCl_3$) δ 21.46, 22.17, 25.32, 38.21, 41.40, 127.04, 129.62, 136.81, 143.25.

benzyl isopentyl carbonate (2n)

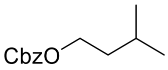

According to the general procedure B (48 h), **2n** was prepared from **1n** (44 mg, 0.20 mmol). The crude was purified by column chromatography (silica gel, hexane/CH_2Cl_2 = 2:1 to 1:1), and **2n** was isolated as a pale yellow oil (38 mg, 85%).

TLC: R_f = 0.34 (hexane/CH_2Cl_2 = 2:1).

NMR spectra of the obtained product were consistent with the reported one.[19]

^{1}H NMR (400 MHz, $CDCl_3$) δ 0.92 (d, J = 6.7 Hz, 6H), 1.56 (dt, J = 6.9, 6.9 Hz, 2H), 1.66-1.76 (m, 1H), 4.18 (t, J = 7.0 Hz, 2H), 5.15 (s, 2H), 7.30-7.39 (m, 5H).

^{13}C NMR (100 MHz, $CDCl_3$) δ 22.35, 24.71, 37.23, 66.76, 69.40, 128.28, 128.44, 128.52, 135.28, 155.21.

isopentyl (2-chloroethyl)carbamate (2o)

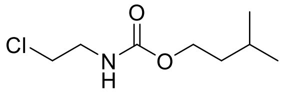

According to the general procedure B (18 h), **2o** was prepared from **1o** (38 mg, 0.20 mmol). The crude was purified by column chromatography (silica gel, hexane/EtOAc = 8:1 to 5:1), and **2o** was isolated as a pale yellow oil (34 mg, 87%).

TLC: R_f = 0.30 (hexane/EtOAc = 5:1).

^{1}H NMR (400 MHz, $CDCl_3$) δ 0.92 (d, J = 6.7 Hz, 6H), 1.51 (q, J = 6.9 Hz, 2H), 1.64-1.74 (m, 1H), 3.52 (q, J = 5.7 Hz, 2H), 3.62 (t, J = 5.4 Hz, 2H), 4.10 (t, J = 7.0 Hz, 2H), 5.13 (s, 1H).

^{13}C NMR (100 MHz, $CDCl_3$) δ 22.42, 24.88, 37.60, 42.68, 44.11, 63.76, 156.56.

HRMS (ESI): *m/z* calculated for $C_8H_{17}ClNO_2$ $[M+H]^+$: 194.0943, found: 194.0949.

isopentyl phenylcarbamate (2p from 1p)

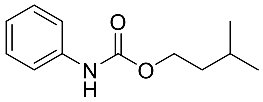

According to the modified general procedure B (18 h; at 40 °C), **2p** was prepared from **1p** (41 mg, 0.20 mmol). The crude was purified by column chromatography (silica gel, hexane/CH_2Cl_2 = 2:1 to CH_2Cl_2), and **2p** was isolated as a colorless solid (36 mg, 86%).

TLC: R_f = 0.30 (hexane/CH_2Cl_2 = 1:1).

^{1}H NMR (500 MHz, $CDCl_3$) δ 0.94 (d, J = 6.9 Hz, 6H), 1.56 (dt, J = 6.9, 6.9 Hz, 2H), 1.68-1.77 (m, 1H), 4.19 (t, J = 6.9 Hz, 2H), 6.70 (s, 1H), 7.05 (t, J = 7.4 Hz, 1H), 7.25-7.31 (m, 2H), 7.38 (d, J = 7.4 Hz, 2H).

^{13}C NMR (125 MHz, $CDCl_3$) δ 22.43, 24.92, 37.58, 63.85, 118.54, 123.25, 128.97, 137.95, 153.72.

benzyl 2-methylbutanoate (2q)

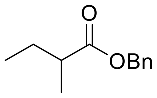

According to the general procedure B with stirring 18 h, **2q** was prepared from **1q** (38 mg, 0.20 mmol). The crude was purified by column chromatography (silica gel, pentane/CH_2Cl_2 = 2:1 to 1:1) followed by gel permeation chromatography (eluent: $CHCl_3$) to afford **2q** as a colorless oil (24 mg, 64%).

TLC: R_f = 0.38 (pentane/CH_2Cl_2 = 1:1).

NMR spectra of the obtained product were consistent with the reported one.[20]

^{1}H NMR (400 MHz, $CDCl_3$) δ 0.90 (t, J = 7.5 Hz, 3H), 1.16 (d, J = 6.8 Hz, 3H), 1.43-1.54 (m, 1H), 1.65-1.76 (m, 1H), 2.38-2.47 (m, 1H), 5.12 (s, 2H), 7.29-7.38 (m, 5H).

^{13}C NMR (125 MHz, $CDCl_3$) δ 11.57, 16.54, 26.75, 41.05, 65.92, 128.01, 128.05, 128.49, 136.27, 176.52.

***trans*-1-(*tert*-butyl)-4-methylcyclohexane (2r)**

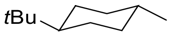

In an argon filled glove box, a flame dried reaction vial was charged with an alkene **1r** (30 mg, 0.20 mmol), ascorbic acid (106 mg, 0.60 mmol), **3f** (12.0 mg, 20 μmol), tricyclohexylphosphine (11.2 mg, 40 μmol) and $Ru(bpy)_3Cl_2$•$6H_2O$ (3.0 mg, 4.0 μmol). The vial was capped and removed from the glove box. A mixed solvent (2-propanol/H_2O = 3:1, 1 mL) was added to the vial via syringe, and the syringe hole was carefully sealed with a vinyl tape. The reaction vial was placed in front of the light source (ca. 3 cm from two blue LED panels) in a cold room (4 °C) so that the temperature of the reaction mixture was kept approximately at 25 °C. After stirring for 40 h, the mixture was cooled in an ice bath and sat. aq. $NaHCO_3$ was added. Organic material was extracted with Et_2O (x 3) and the combined organic layer was washed with brine. The organic layer was analyzed by GCMS using dodecane as an internal standard. The yield of **2r** was determined to be 45% and the ratio of *trans*/*cis* isomers was determined to be 75:25.

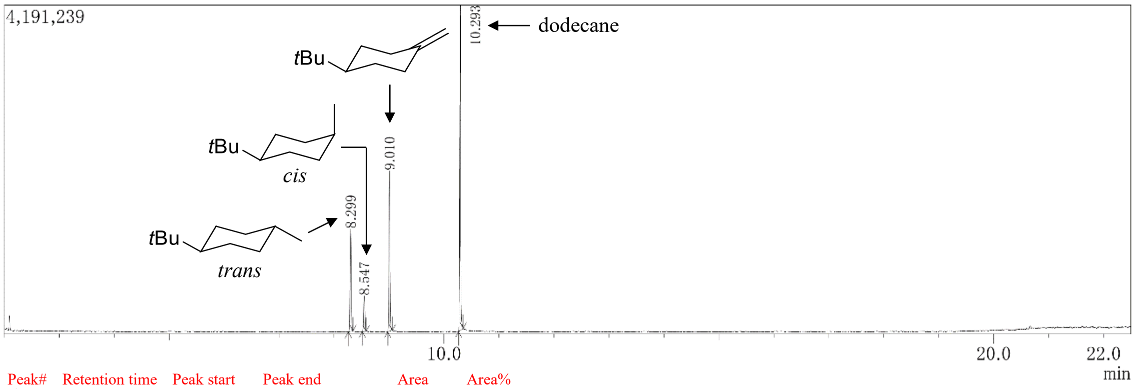

Peak# ピーク#	Retention time 保持時間	Peak start ピーク開始	Peak end ピーク終了	Area 面積	Area% 面積%	
1	8.299	8.260	8.345	1841719	18.81	*trans*
2	8.547	8.515	8.585	618952	6.32	*cis*
3	9.010	8.975	9.060	2519359	25.73	
4	10.293	10.265	10.350	4812879	49.15	

The major diastereomer obtained in the cobalt-photoredox catalyzed hydrogenation of **1r** was determined to be *trans* by comparing the GCMS chart with the chart of a *cis*/*trans* mixture of **2r** obtained by the hydrogenation of **1r** using Wilkinson's catalyst. The *cis*/*trans* mixture of **2r** was prepared as reported in the literature[21] and shown in the scheme below.

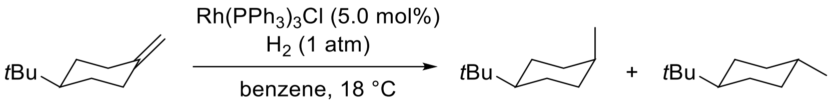

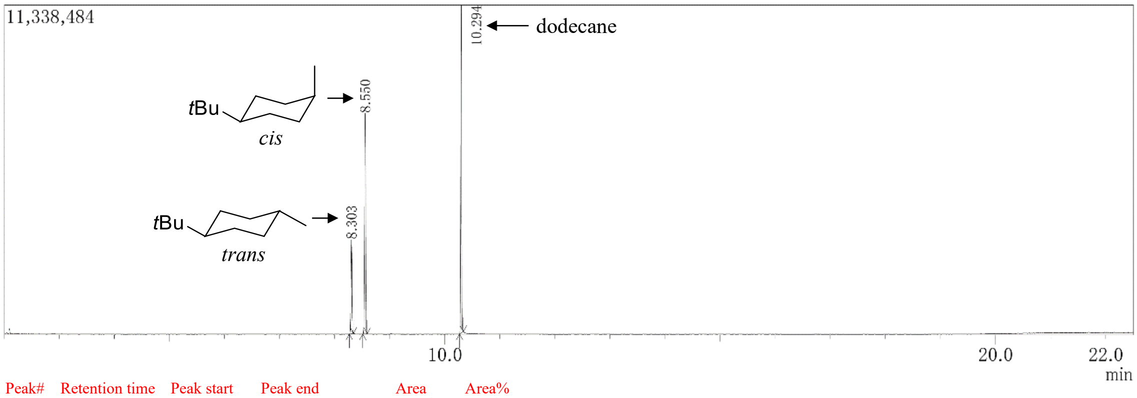

Peak# ピーク#	Retention time 保持時間	Peak start ピーク開始	Peak end ピーク終了	Area 面積	Area% 面積%	
1	8.303	8.265	8.340	4565924	17.01	*trans*
2	8.550	8.510	8.590	9784768	36.45	*cis*
3	10.294	10.265	10.335	12492882	46.54	

2-methylheptane (2s)

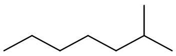

In an argon filled glove box, a flame dried reaction vial was charged with **1s** (22 mg, 0.20 mmol), ascorbic acid (106 mg, 0.60 mmol), **3f** (12.0 mg, 20 µmol), tricyclohexylphosphine (11.2 mg, 40 µmol) and $Ru(bpy)_3Cl_2$•$6H_2O$ (3.0 mg, 4.0 µmol). The vial was capped and removed from the glove box. A mixed solvent (2-propanol/H_2O = 3:1, 1 mL) was added to the vial via syringe, and the syringe hole was carefully sealed with a vinyl tape. The reaction vial was placed in front of the light source (ca. 3 cm from two blue LED panels) in a cold room (4 °C) so that the temperature of the reaction mixture was kept approximately at 25 °C. After stirring for 9 h (longer reaction time led to decrease in the recovery of **1s** and the yield of **2s** possibly due to competitive volatilization of **1s** and **2s** from the reaction vessel), the mixture was cooled in an ice bath and sat. aq. $NaHCO_3$ was added. Organic material was extracted with Et_2O (x 3) and the combined organic layer was washed with brine. The organic layer was analyzed by GCMS using nonane as an internal standard and the yield of **2s** was determined to be 65%.

***tert*-butyl(isopentyloxy)diphenylsilane (2t)**

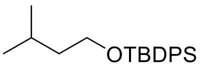

According to the general procedure B (40 h), **2t** was prepared from **1t** (65 mg, 0.20 mmol). The crude was purified by column chromatography (silica gel, hexane/CH_2Cl_2 = 20:1), and **2t** was isolated as a colorless oil (51 mg, 79%).
NMR spectra of the obtained product were consistent with the reported one.[22]
TLC: R_f = 0.33 (hexane/CH_2Cl_2 = 20:1).

^{1}H NMR (400 MHz, $CDCl_3$) δ 0.85 (d, J = 6.8 Hz, 6H), 1.05 (s, 9H), 1.46 (q, J = 6.8 Hz, 2H), 1.70-1.80 (m, 1H), 3.69 (t, J = 6.6 Hz, 2H), 7.35-7.43 (m, 6H), 7.66-7.69 (m, 4H).
^{13}C NMR (100 MHz, $CDCl_3$) δ 19.21, 22.65, 24.65, 26.87, 41.55, 62.35, 127.56, 129.47, 134.18, 135.57.

(*S*)-(((3,7-dimethyloctyl)oxy)methyl)benzene (2u)

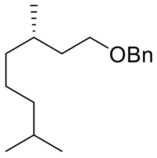

In an argon filled glove box, a flame dried reaction vial was charged with **1u** (49 mg, 0.20 mmol), ascorbic acid (106 mg, 0.60 mmol), **3f** (12.0 mg, 20 µmol), tricyclohexylphosphine (11.2 mg, 40 µmol) and $Ru(bpy)_3Cl_2$•$6H_2O$ (3.0 mg, 4.0 µmol). The vial was capped and removed from the glove box. A mixed solvent (2-propanol/H_2O = 3:1, 1 mL) was added to the vial via syringe, and the syringe hole was carefully sealed with a vinyl tape. The reaction vial was placed in front of the light source (ca. 3 cm from two blue LED panels) in a cold room (4 °C) and the temperature of the reaction mixture was kept approximately at 25 °C. After stirring for 18 h, the mixture was cooled in an ice bath and sat. aq. $NaHCO_3$ was added. Organic material was extracted with EtOAc (x 3) and the combined organic layer was washed with brine. The combined organic layer was concentrated under reduced pressure and the residue was dissolved in EtOAc and transferred into a reaction vial. After evaporation of the solvent 2-propanol was added to the vial. The 2-propanol was removed under reduced pressure and thus the crude product was subjected to the same reaction setup as described above. After stirring for 18 h, the mixture was cooled in an ice bath and sat. aq. $NaHCO_3$ was added. Organic material was extracted with EtOAc (x 3) and the combined organic layer was washed with brine. The organic layer was concentrated under reduced pressure. The crude product was purified by column chromatography (silica gel, hexane/toluene = 1:1) to afford **2u** as a pale yellow oil (42 mg, 84% for 2 cycles).
NMR spectra of the obtained product were consistent with the reported one.[23]
TLC: R_f = 0.41 (hexane/toluene = 1:1).
^{1}H NMR (500 MHz, $CDCl_3$) δ 0.86 (d, J = 6.3 Hz, 6H), 0.87 (d, J = 6.9 Hz, 3H), 1.06-1.17 (m, 3H), 1.19-1.35 (m, 3H), 1.38-1.45 (m, 1H), 1.48-1.60 (m, 2H), 1.63-1.69 (m, 1H), 3.46-3.54 (m, 2H), 4.50 (s, 2H), 7.25-7.29 (m, 1H), 7.31-7.36 (m, 4H).
^{13}C NMR (125 MHz, $CDCl_3$) δ 19.67, 22.58, 22.69, 24.65, 27.94, 29.86, 36.79, 37.33, 39.26, 68.76, 72.87, 127.42, 127.58, 128.31, 138.70.

5-methyl-*N*-phenylhexanamide (2v)

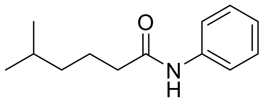

According to the general procedure B (18 h), **2v** was prepared from **1v** (41 mg, 0.20 mmol). The crude was purified by column chromatography (silica gel, hexane/EtOAc = 4:1), and an inseparable mixture of **2v** and **1v** was obtained as a colorless solid (37 mg in total: 35 mg (0.168 mmol, 84%) of **2v** and 2 mg (0.010 mmol) of **1v** judging from ^{1}H NMR analysis).

TLC: R_f = 0.24 (hexane/EtOAc = 4:1).

^{1}H NMR (400 MHz, $CDCl_3$) δ 0.88 (d, J = 6.3 Hz, 6H), 1.21-1.29 (m, 2H), 1.51-1.61 (m, 1H), 1.68-1.76 (m, 2H), 2.33 (t, J = 7.6 Hz, 2H), 7.09 (t, J = 7.4 Hz, 1H), 7.30 (t, J = 8.1 Hz, 2H), 7.49 (s, 1H), 7.52 (d, J = 8.1 Hz, 2H). The signals at δ 2.39, 5.16 are derived from the alkene **1v**.

^{13}C NMR (100 MHz, $CDCl_3$) δ 22.46, 23.50, 27.83, 37.96, 38.42, 119.80, 124.10, 128.90, 137.96, 171 58. The signals at δ 25.70, 122.52 are derived from the alkene **1v**.

HRMS (ESI): *m/z* calculated for $C_{13}H_{19}NONa$ $[M+Na]^+$: 228.1359, found: 228.1362.

benzyl (5-methylhexanoyl)-L-glutaminate (2w)

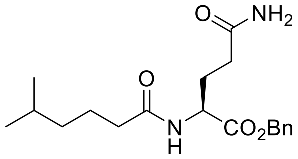

In an argon filled glove box, a flame dried reaction vial was charged with **1w** (69 mg, 0.20 mmol), ascorbic acid (106 mg, 0.60 mmol), **3f** (12.0 mg, 20 µmol), tricyclohexylphosphine (11.2 mg, 40 µmol) and $Ru(bpy)_3Cl_2 \cdot 6H_2O$ (3.0 mg, 4.0 µmol). The vial was capped and removed from the glove box. A mixed solvent (2-propanol/H_2O = 3:1, 1 mL) was added to the vial via syringe, and the syringe hole was carefully sealed with a vinyl tape. The reaction vial was placed in front of the light source (ca. 3 cm from two blue LED panels) in a cold room (4 °C) so that the temperature of the reaction mixture was kept approximately at 25 °C. After stirring for 48 h, the mixture was cooled in an ice bath and sat. aq. $NaHCO_3$ was added. Organic material was extracted with EtOAc (x 3) and the combined organic layer was washed with brine. After evaporation of the solvent, the crude product was dissolved in 2-propanol and was transferred into a reaction vial. The 2-propanol was removed under reduced pressure and thus the crude product was subjected to the same reaction setup as described above. After stirring for 48 h, the mixture was cooled in an ice bath and sat. aq. $NaHCO_3$ was added. Organic material was extracted with EtOAc (x 3) and the combined organic layer was washed with brine. The organic layer was concentrated under reduced pressure and the residue was suspended in EtOAc and filtered. The filtrate was concentrated and purified by column chromatography (silica gel, hexane/acetone = 2:1 to 2:3) to afford **2w** as a colorless solid (58 mg, 84% for 2 cycles).

TLC: R_f = 0.29 (hexane/acetone = 1:1).

^{1}H NMR (500 MHz, $CDCl_3$) δ 0.87 (d, J = 6.3 Hz, 6H), 1.17-1.21 (m, 2H), 1.50-1.58 (m, 1H), 1.59-1.65 (m, 2H), 1.93-2.01 (m, 1H), 2.16-2.24 (m, 3H), 2.26-2.34 (m, 2H), 4.63 (m, 1H), 5.16 (d, J = 12.0 Hz, 1H), 5.19 (d, J = 12.0 Hz, 1H), 5.60 (s, 1H), 6.29 (s, 1H), 6.58 (d, J = 7.4 Hz, 1H), 7.32-7.38 (m, 5H).

^{13}C NMR (125 MHz, $CDCl_3$) δ 22.46, 23.36, 27.74, 28.30, 31.76, 36.68, 38.40, 51.84, 67.38, 128.35, 128.55, 128.64, 135.10, 171.83, 173.79, 174.65.

HRMS (ESI): m/z calculated for $C_{19}H_{28}N_2O_4Na$ $[M+Na]^+$: 371.1941, found: 371.1948.

benzyl (5-methylhexanoyl)-L-serinate (2x)

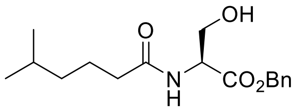

According to the general procedure B (18 h), **2x** was prepared from **1x** (61 mg, 0.20 mmol). The crude was purified by column chromatography (silica gel, CH_2Cl_2/EtOAc = 2:1 to 1:1) and an inseparable mixture of **2x** and **1x** was obtained as a colorless solid (58 mg in total: 54 mg (0.177 mmol, 89%) of **2x** and 4 mg (0.012 mmol) of **1x** judging from ^{1}H NMR analysis).

TLC: R_f = 0.35 (CH_2Cl_2/EtOAc = 2/1).

^{1}H NMR (400 MHz, $CDCl_3$) δ 0.86 (d, J = 6.7 Hz, 6H), 1.17-1.21 (m, 2H), 1.48-1.67 (m, 3H), 2.22 (t, J = 7.6 Hz, 2H), 3.02 (s, 1H), 3.88 (dd, J = 11.2, 3.2 Hz, 1H), 3.98 (dd, J = 11.2, 4.0 Hz, 1H), 4.69-4.72 (m, 1H), 5.19 (s, 2H), 6.62 (d, J = 7.6 Hz, 1H), 7.27-7.38 (m, 5H).

^{13}C NMR (100 MHz, $CDCl_3$) δ 22.38, 23.32, 27.68, 36.56, 38.31, 54.61, 63.11, 67.32, 128.00, 128.37, 128.52, 135.05, 170.43, 174.15. The signals at δ 17.61, 24.01, 25.58, 36.29, 122.29, 133.32 are derived from the alkene **1x**.

HRMS (ESI): m/z calculated for $C_{17}H_{25}NO_4Na$ $[M+Na]^+$: 330.1676, found: 330.1683.

benzyl (5-methylhexanoyl)-L-methioninate (2y)

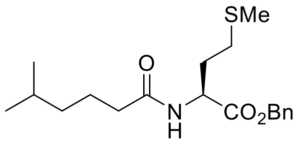

In an argon filled glove box, a flame dried reaction vial was charged with **1y** (70 mg, 0.20 mmol), ascorbic acid (106 mg, 0.60 mmol), **3f** (12.0 mg, 20 µmol), tricyclohexylphosphine (11.2 mg, 40 µmol) and $Ru(bpy)_3Cl_2 \cdot 6H_2O$ (3.0 mg, 4.0 µmol). The vial was capped and removed from the glove box. A mixed solvent (2-propanol/H_2O = 3:1, 1 mL) was added to the vial via syringe, and the syringe hole was carefully sealed with a vinyl tape. The reaction vial was placed in front of the light source (ca. 3 cm from two blue LED panels) in a cold room (4 °C) and the temperature of the reaction mixture was

kept approximately at 25 °C. After stirring for 48 h, the mixture was cooled in an ice bath and sat. aq. $NaHCO_3$ was added. Organic material was extracted with EtOAc (x 3) and the combined organic layer was washed with brine. The combined organic layer was concentrated under reduced pressure and the residue was filtered through a short pad of silica gel using EtOAc. After evaporation of the solvent, the crude was dissolved in 2-propanol and transferred into a reaction vial. The 2-propanol was removed under reduced pressure and thus the crude product was subjected to the same reaction setup as described above. After stirring for 48 h, the mixture was cooled in an ice bath and sat. aq. $NaHCO_3$ was added. Organic material was extracted with EtOAc (x 3) and the combined organic layer was washed with brine. The organic layer was concentrated under reduced pressure and the residue was filtered through a short pad of silica gel using EtOAc. After removal of the solvent, the crude product was purified by column chromatography (silica gel, hexane/EtOAc = 3:1 to 5:2) to afford **2y** as a pale yellow solid (60 mg, 85% for 2 cycles).

TLC: $R_f = 0.25$ (hexane/EtOAc = 3:1).

^{1}H NMR (500 MHz, $CDCl_3$) δ 0.87 (d, J = 6.3 Hz, 6H), 1.17-1.22 (m, 2H), 1.50-1.58 (m, 1H), 1.60-1.66 (m, 2H), 1.95-2.02 (m, 1H), 2.04 (s, 3H), 2.12-2.22 (m, 3H), 2.42-2.52 (m, 2H), 4.77 (td, J = 7.6, 5.2 Hz, 1H), 5.15 (d, J = 12.6 Hz, 1H), 5.20 (d, J = 12.0 Hz, 1H), 6.30 (d, J = 8.0 Hz, 1H), 7.31-7.38 (m, 5H).

^{13}C NMR (125 MHz, $CDCl_3$) δ 15.30, 22.39, 23.34, 27.69, 29.75, 31.66, 36.65, 38.34, 51.42, 67.20, 128.23, 128.44, 128.54, 135.08, 171.89, 172.98.

HRMS (ESI): m/z calculated for $C_{19}H_{29}NO_3SNa$ $[M+Na]^+$: 374.1760, found: 374.1762.

benzyl (5-methylhexanoyl)glycylglycinate (2z)

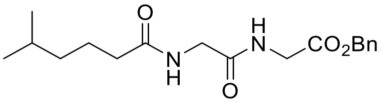

According to the general procedure B (48 h), **2z** was prepared from **1z** (66 mg, 0.2 mmol). The crude was purified by reverse phase column chromatography (ODS, H_2O/MeCN = 56:44 to 31:69), and **2z** was isolated as a colorless solid (53 mg, 79%).

TLC: $R_f = 0.40$ (CH_2Cl_2/MeOH = 10:1).

^{1}H NMR (500 MHz, $CDCl_3$) δ 0.87 (d, J = 6.5 Hz, 6H), 1.16-1.21 (m, 2H), 1.49-1.67 (m, 3H), 2.21 (t, J = 7.5 Hz, 2H), 3.99 (d, J = 5.0 Hz, 2H), 4.05 (d, J = 5.5 Hz, 2H), 5.15 (s, 2H), 6.83 (s, 1H), 7.27-7.36 (m, 5H).

^{13}C NMR (125 MHz, $CDCl_3$) δ 22.39, 23.34, 27.72, 36.36, 38.38, 41.21, 43.00, 67.06, 128.21, 128.42, 128.54, 135.09, 169.45, 169.75, 174.05.

HRMS (ESI): m/z calculated for $C_{18}H_{26}N_2O_4Na$ $[M+Na]^+$: 357.1785, found: 357.1791.

(1*R*,2*S*,5*R*)-2-isopropyl-5-methylcyclohexan-1-ol (2aa)

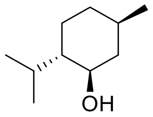

According to the general procedure B (18 h), **2aa** was prepared from **1aa** (32 mg, 0.20 mmol). The crude was purified by column chromatography (silica gel, pentane/Et_2O = 4:1 to 3:1), and **2aa** was isolated as a colorless solid (25 mg, 78%).

TLC: R_f = 0.30 (pentane/Et_2O = 3:1).

NMR spectra of the obtained product were consistent with the reported one.[24]

^{1}H NMR (400 MHz, $CDCl_3$) δ 0.81 (d, *J* = 7.2 Hz, 3H), 0.83-0.89 (m, 1H), 0.91 (d, *J* = 7.6 Hz, 3H), 0.93 (d, *J* = 5.2 Hz, 3H), 0.95-1.02 (m, 2H), 1.08-1.15 (m, 1H), 1.36-1.48 (m, 1H), 1.52 (s, 1H), 1.58-1.69 (m, 2H), 1.94-1.99 (m, 1H), 2.13-2.21 (m, 1H), 3.42 (td, *J* = 10.3, 4.0 Hz, 1H).

^{13}C NMR (100 MHz, $CDCl_3$) δ 16.03, 20.99, 22.19, 23.07, 25.76, 31.60, 34.49, 44.99, 50.10, 71.51.

3,7-dimethyloctan-3-ol (2ab)

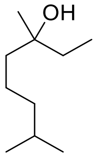

According to the general procedure B (18 h), **2ab** was prepared from **1ab** (31 mg, 0.20 mmol). The crude was purified by column chromatography (silica gel, hexane/Et_2O = 2:1 to 1:1), and **2ab** was isolated as a brown oil (29 mg, 93%).

TLC: R_f = 0.39 (hexane/Et_2O = 1:1).

NMR spectra of the obtained product were consistent with the reported one.[25]

^{1}H NMR (400 MHz, $CDCl_3$) δ 0.87-0.89 (m, 9H), 1.14 (s, 3H), 1.16-1.44 (m, 6H), 1.49 (q, *J* = 7.6 Hz, 2H), 1.55 (sept, *J* = 6.8 Hz, 1H).

^{13}C NMR (100 MHz, $CDCl_3$) δ 8.21, 21.59, 22.61, 26.39, 27.94, 34.20, 39.54, 41.53, 72.94.

(*S*)-3,7-dimethyloctan-1-ol (2ac)

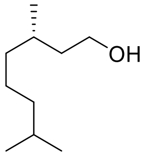

According to the general procedure B (40 h), **2ac** was prepared from **1ac** (31 mg, 0.20 mmol). The crude was purified by column chromatography (silica gel, pentane/Et_2O = 3:1 to 2:1) and an

inseparable mixture of **2ac** and **1ac** was obtained as a yellow oil (28 mg in total: 26 mg (0.160 mmol, 80%) of **2ac** and 2 mg (0.015 mmol) of **1ac** judging from ^{1}H NMR analysis).

TLC: R_f = 0.30 (pentane/Et_2O = 2:1).

NMR spectra of the obtained product were consistent with the reported one.[26]

^{1}H NMR (400 MHz, $CDCl_3$) δ 0.87 (d, J = 6.8 Hz, 6H), 0.91 (d, J = 5.2 Hz, 3H), 1.11-1.37 (m, 7H), 1.51-1.62 (m, 3H), 3.64-3.75 (m, 2H). The signal at δ 5.10 is derived from the alkene **1y**.

^{13}C NMR (100 MHz, $CDCl_3$) δ 19.60, 22.56, 22.65, 24.64, 27.93, 29.48, 37.34, 39.22, 39.99, 61.21.

methyl 6-(4,6-dimethoxy-7-methyl-3-oxo-1,3-dihydroisobenzofuran-5-yl)-4-methylhexanoate (2ad')

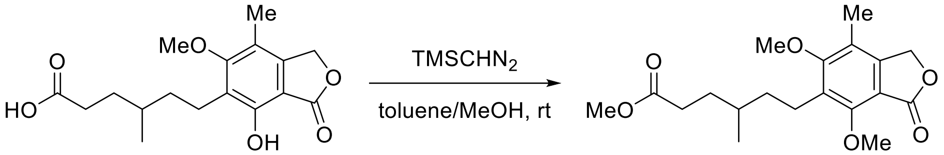

2ad **2ad'**

In an argon filled glove box, a flame dried reaction vial was charged with **1ad** (64 mg, 0.20 mmol), ascorbic acid (106 mg, 0.60 mmol), **3f** (12.0 mg, 20 µmol), tricyclohexylphosphine (11.2 mg, 40 µmol) and Ru(bpy)$_3$Cl$_2$•6H_2O (3.0 mg, 4.0 µmol). The vial was capped and removed from the glove box. A mixed solvent (2-propanol/H_2O = 3:1, 1 mL) was added to the vial via syringe, and the syringe hole was carefully sealed with a vinyl tape. The reaction vial was placed in front of the light source (ca. 3 cm from two blue LED panels) in a cold room (4 °C) and the temperature of the reaction mixture was kept approximately at 25 °C. After stirring for 18 h, the mixture was cooled in an ice bath and 1 M aq. HCl was added. Organic material was extracted with EtOAc (x 3) and the combined organic layer was washed with brine. The organic layer was concentrated under reduced pressure. The residue was dissolved in 2-propanol and transferred into a reaction vial. The 2-propanol was removed under reduced pressure and thus the crude product was subjected to the same reaction setup as described above. After stirring for 18 h, the mixture was cooled in an ice bath and 1 M aq. HCl was added. Organic material was extracted with EtOAc (x 3) and the combined organic layer was washed with brine. The organic layer was concentrated under reduced pressure. Then, the crude **2ad** was dissolved in toluene/MeOH = 3:1 (2 mL) and $TMSCHN_2$ (2.0 M in Et_2O, 0.35 mL, 0.70 mmol) was added to the solution. After stirred for 22 hours, $TMSCHN_2$ (2.0 M in Et_2O, 0.10 mL, 0.2 mmol) was added. After stirred for 14 hours a few drops of AcOH were added, and volatiles were removed under reduced pressure. The crude was purified by column chromatography (silica gel, hexane/EtOAc = 3:1 to 2:1) and **2ad'** was isolated as a colorless solid (60 mg, 86% for 2 cycles).

TLC: R_f = 0.40 (hexane/EtOAc = 2:1).

^{1}H NMR (500 MHz, $CDCl_3$) δ 1.00 (d, J = 6.3 Hz, 3H), 1.35-1.40 (m, 1H), 1.48-1.58 (m, 3H), 1.74-1.81 (m, 1H), 2.19 (s, 3H), 2.31-2.41 (m, 2H), 2.62-2.71 (m, 2H), 3.68 (s, 3H), 3.81 (s, 3H), 4.07 (s, 3H), 5.14 (s, 2H).

^{13}C NMR (125 MHz, $CDCl_3$) δ 11.44, 19.08, 21.69, 31.42, 31.60, 32.65, 37.21, 51.36, 60.99, 62.63, 68.23, 112.34, 119.84, 130.14, 146.41, 156.55, 162.72, 168.88, 174.36.

HRMS (ESI): *m/z* calculated for $C_{19}H_{26}O_6Na$ $[M+Na]^+$: 372.1622, found: 372.1625.

***N*-(3,4-dimethoxybenzyl)-8-methylnonanamide (2ae')**

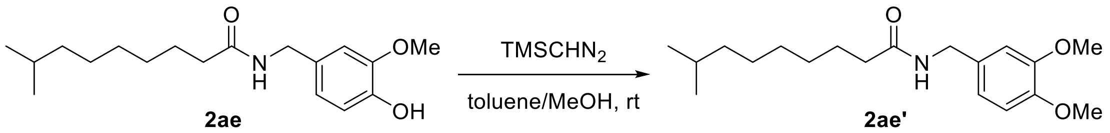

In an argon filled glove box, a flame dried reaction vial was charged with **1ae** (61 mg, 0.20 mmol), ascorbic acid (106 mg, 0.60 mmol), **3f** (12.0 mg, 20 µmol), tricyclohexylphosphine (11.2 mg, 40 µmol) and $Ru(bpy)_3Cl_2•6H_2O$ (3.0 mg, 4.0 µmol). The vial was capped and removed from the glove box. A mixed solvent (2-propanol/H_2O = 3:1, 1 mL) was added to the vial via syringe, and the syringe hole was carefully sealed with a vinyl tape. The reaction vial was placed in front of the light source (ca. 3 cm from two blue LED panels) in a cold room (4 °C) so that the temperature of the reaction mixture was kept approximately at 25 °C. After stirring for 18 h, the mixture was cooled in an ice bath and 1 M aq. HCl was added. Organic material was extracted with EtOAc (x 3) and the combined organic layer was washed with brine. The organic layer was concentrated under reduced pressure. The residue was dissolved in 2-propanol and transferred into a reaction vial. The 2-propanol was removed under reduced pressure and thus the crude product was subjected to the same reaction setup as described above. After stirring for 18 h, the mixture was cooled in an ice bath and 1 M aq. HCl was added. Organic material was extracted with EtOAc (x 3) and the combined organic layer was washed with brine. The organic layer was concentrated under reduced pressure. Then, the crude **2ae** was dissolved in toluene/MeOH = 3:1 (2 mL) and $TMSCHN_2$ (2.0 M in Et_2O, 0.30 mL, 0.60 mmol) was added to the solution. After stirred for 22 hours a few drops of AcOH were added, and volatiles were removed under reduced pressure. The crude was purified by column chromatography (silica gel, hexane/EtOAc = 1:1 to 2:3) and **2ae'** was isolated as a colorless solid (61 mg, 95% for 2 cycles).

TLC: R_f = 0.24 (hexane/EtOAc = 1:1).

^{1}H NMR (400 MHz, $CDCl_3$) δ 0.82 (d, J = 6.5 Hz, 6H), 1.09-1.13 (m, 2H), 1.20-1.29 (m, 6H), 1.41-1.51 (m, 1H), 1.57-1.64 (m, 2H), 2.16 (t, J = 7.6 Hz, 2H), 3.81 (s, 6H), 4.31 (d, J = 5.8 Hz, 2H), 6.03 (s, 1H), 6.74-6.48 (m, 3H).

^{13}C NMR (100 MHz, $CDCl_3$) δ 22.49, 25.70, 27.12, 27.79, 29.24, 29.50, 36.64, 38.80, 43.16, 55.67, 55.75, 110.95, 110.97, 119.88, 131.01, 148.19, 148.91, 172.92.

HRMS (ESI): *m/z* calculated for $C_{19}H_{31}NO_3Na$ $[M+Na]^+$: 344.2196, found: 344.2199.

((2*R*,3*S*,4*R*,5*S*)-3,4-dihydroxy-5-isobutyltetrahydrofuran-2-yl)methyl acetate (2af)

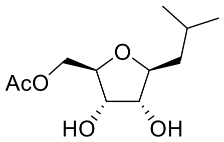

According to the general procedure B (18 h), **2af** was prepared from **1af** (46 mg, 0.20 mmol). The crude was purified by column chromatography (silica gel, CH_2Cl_2/EtOAc = 1:1 to 1:2), and **2af** was isolated as a colorless oil (42 mg, 90%).

TLC: R_f = 0.45 (CH_2Cl_2/EtOAc = 1:2).

^{1}H NMR (500 MHz, $CDCl_3$) δ 0.94 (t, J = 6.3 Hz, 6H), 1.38-1.49 (m, 2H), 1.75-1.84 (m, 1H), 2.10 (s, 3H), 3.38-3.53 (m, 2H), 3.74 (t, J = 5.2 Hz, 1H), 3.83 (dt, J = 8.0, 5.2 Hz, 1H), 3.91-3.94 (m, 2H), 4.11 (dd, J = 11.7, 4.9 Hz, 1H), 4.32 (dd, J = 12.0, 2.9 Hz, 1H).

^{13}C NMR (125 MHz, $CDCl_3$) δ 20.86, 22.16, 23.22, 24.92, 42.89, 64.60, 71.87, 75.27, 80.63, 82.10, 171.38.

HRMS (ESI): *m/z* calculated for $C_{11}H_{20}O_5Na$ $[M+Na]^+$: 255.1203, found: 255.1200.

(2*R*,3*S*,4*R*,5*S*,6*S*)-2-(acetoxymethyl)-6-isobutyltetrahydro-2*H*-pyran-3,4,5-triyl triacetate (2ag)

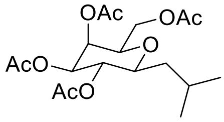

According to the general procedure B (18 h), **2ag** was prepared from **1ag** (77 mg, 0.20 mmol). The crude was purified by column chromatography (silica gel, hexane/EtOAc = 2:1), and **2ag** was isolated as a colorless solid (71 mg, 92%).

NMR spectra of the obtained product were consistent with the reported one.[14]

TLC: R_f = 0.35 (hexane/EtOAc = 2:1).

^{1}H NMR (500 MHz, $CDCl_3$) δ 0.88 (d, J = 6.9 Hz, 3H), 0.92 (d, J = 6.9 Hz, 3H), 1.21 (ddd, J = 14.2, 9.6, 2.4 Hz, 1H), 1.51-1.57 (m, 1H), 1.80-1.87 (m, 1H), 1.98 (s, 3H), 2.04 (s, 3H), 2.05 (s, 3H), 2.15 (s, 3H), 3.44 (td, J = 9.7, 2.1 Hz, 1H), 3.83-3.86 (m, 1H), 4.05 (dd, J = 11.5, 6.9 Hz, 1H), 4.15 (dd, J = 10.9, 6.9 Hz, 1H), 5.00-5.08 (m, 2H), 5.41 (dd, J = 2.9, 1.1 Hz, 1H).

^{13}C NMR (125 MHz, $CDCl_3$) δ 20.51, 20.53, 20.60, 20.70, 21.33, 23.37, 24.15, 40.08, 61.56, 67.68, 69.70, 72.17, 74.02, 76.56, 169.75, 170.09, 170.22, 170.29.

(2*R*,3*R*,4*R*,5*R*,6*S*)-2-(hydroxymethyl)-6-isobutyltetrahydro-2*H*-pyran-3,4,5-triol

isobutyl-β-*C*-galactoside (2ah)

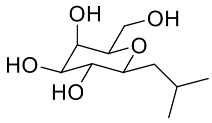

In an argon filled glove box, a flame dried reaction vial was charged with **1ah** (44 mg, 0.20 mmol), ascorbic acid (106 mg, 0.60 mmol), **3f** (12.0 mg, 20 µmol), tricyclohexylphosphine (11.2 mg, 40 µmol) and $Ru(bpy)_3Cl_2$•$6H_2O$ (3.0 mg, 4.0 µmol). The vial was capped and removed from the glove box. A mixed solvent (2-propanol/H_2O = 3:1, 1 mL) was added to the vial via syringe, and the syringe hole was carefully sealed with a vinyl tape. The reaction vial was placed in front of the light source (ca. 3 cm from two blue LED panels) in a cold room (4 °C) so that the temperature of the reaction mixture was kept approximately at 25 °C. After stirring for 18 h, the reaction mixture was washed with toluene (x 3), and aqueous layer was azeotroped with MeCN. The residue was dissolved in MeOH and filtered through a short pad of silica gel using MeOH. The methanol solution was concentrated under reduced pressure to afford the crude product, which was purified by reverse phase column chromatography (ODS, H_2O/MeCN = 1:9) to afford **2ah** as a colorless amorphous solid (39 mg, 89%).

TLC: R_f = 0.50 (Reverse phase TLC, H_2O/MeCN = 1:20).

NMR spectra of the obtained product were consistent with the reported one.[14]

^{1}H NMR (400 MHz, D_2O) δ 0.86 (d, J = 6.3 Hz, 3H), 0.89 (d, J = 6.7 Hz, 3H), 1.34-1.41 (m, 1H), 1.52-1.55 (m, 1H), 1.76-1.86 (m, 1H), 3.23-3.28 (m, 1H), 3.33-3.38 (m, 1H), 3.54-3.58 (m, 2H), 3.64-3.70 (m, 2H), 3.91 (d, J = 3.6 Hz, 1H).

^{13}C NMR (100 MHz, D_2O) δ 21.51, 23.75, 24.57, 40.91, 61.97, 69.81, 72.12, 74.78, 78.85 ,79.16.

2-4. Comparison of the hydrogenation performance for the preparation of IBCG **2ah**

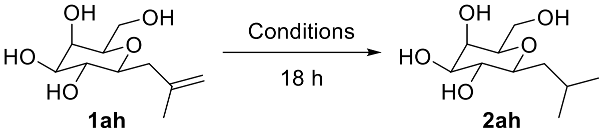

Experimental procedures

[Shenvi's conditions][21]

In an argon filled glove box, a flame dried reaction vial was charged with **1ah** (44 mg, 0.20 mmol), phenylsilane (22 mg, 0.20 mmol) and 2-propanol (0.4 mL). The vial was capped and removed from the glove box. *tert*-Butyl hydroperoxide (5.5 M in decane, 55 μL, 0.30 mmol) was added to the vial via pipette and the resulting mixture was degassed by bubbling argon through the solution for 10 minutes. Tris(2,2,6,6-tetramethyl-3,5-heptanedionato)manganese(III) (12.0 mg, 20 μmol) was added to the vial. The reaction mixture was further degassed by bubbling argon for 30 seconds, and then the syringe hole was carefully sealed with a vinyl tape. The reaction mixture was stirred at 22 °C. After 18 h, the reaction mixture was concentrated under reduced pressure. The residue was dissolved in MeOH and filtered through a short pad of silica gel using MeOH. The filtrate was concentrated under reduced pressure and analyzed by ^{1}H NMR in D_2O using dimethyl sulfone as an internal standard. Thus, yield of **2ah** was determined to be 35%.

[Herzon's conditions][27]

In an argon filled glove box, a flame dried test tube was charged with **1ah** (44 mg, 0.20 mmol), $Co(acac)_2$ (13 mg, 50 μmol), tricyclohexylphosphine (14 mg, 50 μmol) and 2,6-di-*tert*-butyl-4-methylpyridine (21 mg, 0.10 mmol). The test tube was removed from the glove box and 1-propanol (670 μL), freshly distilled 1,4-cyclohexadiene (93 μL, 1.0 mmol), *tert*-butyl hydroperoxide (5.5 M in decane, 9.1 μL, 50 μmol) and triethylsilane (160 μL, 1.0 mmol) were sequentially added via syringe. The resulting reaction mixture was stirred at 50 °C for 18 h. After the reaction mixture was cooled to room temperature, volatiles were removed under reduced pressure. The residue was dissolved in MeOH and filtered through a short pad of silica gel using MeOH. The filtrate was concentrated under reduced pressure and analyzed by ^{1}H NMR in D_2O using dimethylformamide as an internal standard. Thus, yield of **2ah** was determined to be 5%.

[Pd/C-Catalyzed hydrogenation][13]

A flame-dried test tube was charged with **1ah** (44 mg, 0.20 mmol), palladium on carbon (10%, 3.4 mg, 3.2 μmol), ethanol (0.36 mL) and H_2O (3.2 μL). The mixture was subjected to H_2 atmosphere (balloon) and was stirred at 25 °C for 18 hours. Then, the mixture was filtered through celite using methanol and concentrated under reduced pressure. Yield of **2ah** was determined to be 96% by ^{1}H NMR analysis of the crude mixture in D_2O using dimethylsulfone as an internal standard.

2-5. Detection of the alkyl radical intermediate

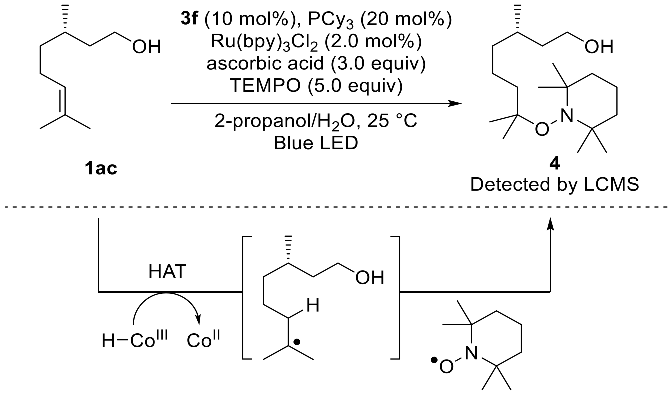

In an argon filled glove box, a flame dried reaction vial was charged with **1ac** (31 mg, 0.20 mmol), ascorbic acid (106 mg, 0.60 mmol), **3f** (12.0 mg, 20 μmol), tricyclohexylphosphine (11.2 mg, 40 μmol) and $Ru(bpy)_3Cl_2$•$6H_2O$ (3.0 mg, 4.0 μmol). The vial was capped and removed from the glove box. A solution of TEMPO (156 mg, 1.0 mmol) in mixed solvent (2-propanol/H_2O = 3:1, 1 mL) was added to the vial via syringe, and the syringe hole was carefully sealed with a vinyl tape. The reaction vial was placed in front of the light source (ca. 3 cm from two blue LED panels) in a cold room (4 °C) so that the temperature of the reaction mixture was kept approximately at 25 °C. After stirring for 18 h, the mixture was cooled in an ice bath and sat. aq. $NaHCO_3$ was added. Organic material was extracted with EtOAc (x 3) and the combined organic layer was washed with brine. The organic layer was concentrated under reduced pressure. The *m/z* of **4** ($[M+H]^+$: 314.30) was observed by LCMS analysis of the crude mixture. The identity of **4** was further confirmed by comparing the retention time in LCMS to that of **4** independently prepared by Boger's method.[28] The crude was also analyzed by ^1H NMR in $CDCl_3$ using 1,1,2,2-tetrachloroethane as an internal standard and the yield of **4** was determined to be 9% along with 88% recovery of **1ac**.

(*S*)-3,7-dimethyl-7-((2,2,6,6-tetramethylpiperidin-1-yl)oxy)octan-1-ol (4)

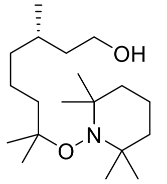

4 was independently synthesized according to the reported procedure.[28] NMR spectra of the obtained product were consistent with the reported one.[29]

^1H NMR (500 MHz, $CDCl_3$) δ 0.92 (3H, d, J = 6.9 Hz), 1.07 (6H, s), 1.10 (6H, s), 1.12-1.68 (16H, m), 1.24 (6H, s), 3.65-3.74 (2H, m).

^{13}C NMR (125 MHz, $CDCl_3$) δ 17.11, 19.61, 20.59, 20.60, 21.39, 26.85, 26.87, 29.47, 34.74, 37.88, 39.96, 40.82, 43.75, 59.06, 59.08, 61.23, 78.59.

LCMS chart of the reaction mixture

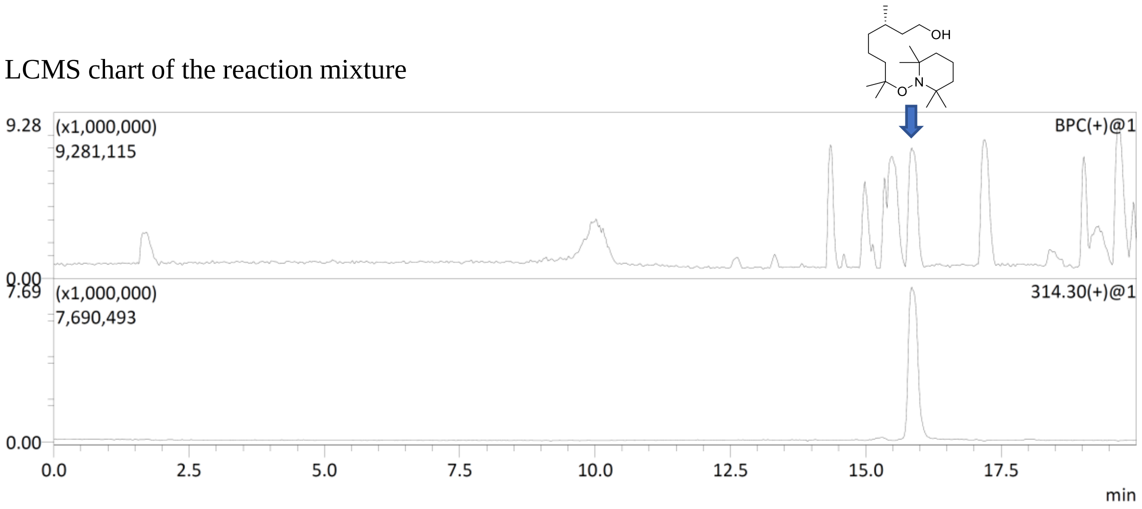

LCMS chart of independently synthesized **4**

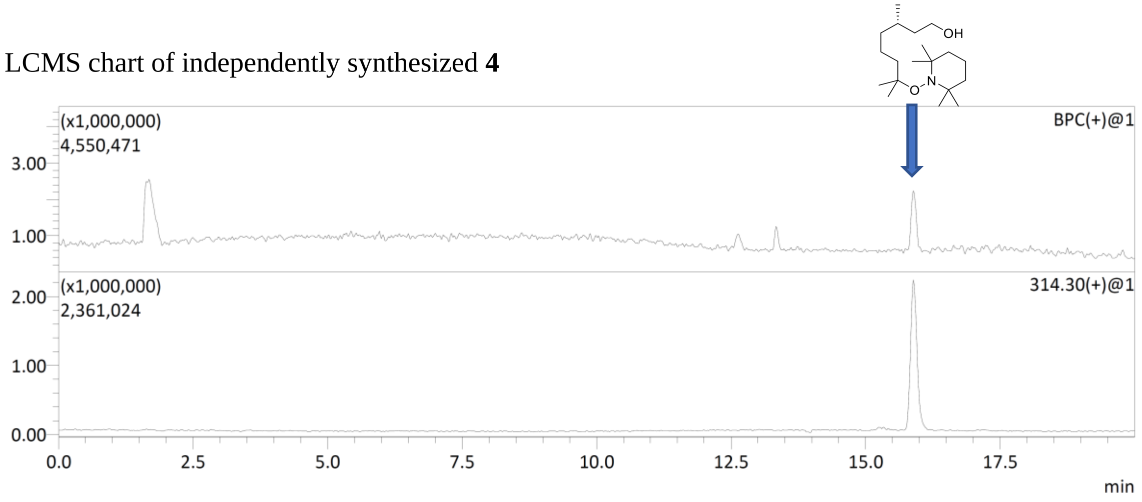

3. Additional investigations of reaction conditions

3-1. Effect of other photocatalysts

Hydrogenation of **1a** was attempted using other photocatalysts instead of $Ru(bpy)_3Cl_2$ in order to assess the relationship between the reactivity of hydrogenation and reducing power of the photocatalyst. The results are summarized in Supplementary Table 1. Compared to $Ru(bpy)_3Cl_2$, less reducing rhodamine 6G did not promote the hydrogenation (entry 2). Comparably reducing photocatalysts promoted the hydrogenation in varying degree (entries 3,4). More reducing photocatalyst realized the hydrogenation in even higher yield (entry 5). These results indicate that feasibility of photocatalytic reduction of **3f** ($E_{1/2}$ = −1.33 V vs. SCE for Co^{II}/Co^{I} redox couple) partly determines the overall efficiency of the hydrogenation.

Supplementary Table 1. Effect of other photocatalysts in the hydrogenation of **1a**

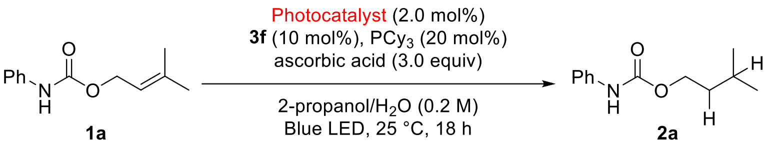

Entry	Photocatalyst	NMR Yield of **2a** (%)	E_{red} (PC/PC•−) [V vs. SCE]
1	$Ru(bpy)_3Cl_2$	90	–1.33
2	rhodamine 6G	0	–1.14
3	4CzIPN	58	–1.21
4	$[Ir(dF(CF_3)ppy)_2(dtbbpy)]PF_6$	38	–1.37
5	$[Ir(ppy)_2(dtbbpy)]PF_6$	>99	–1.51

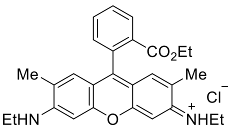

rhodamine 6G

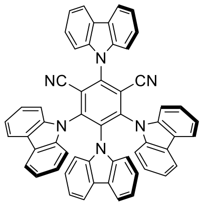

4CzIPN

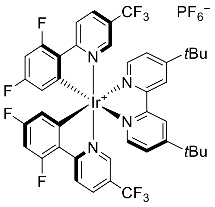

$[Ir(dF(CF_3)ppy)_2(dtbbpy)]PF_6$

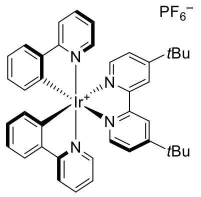

$[Ir(ppy)_2(dtbbpy)]PF_6$

3-2. Effect of other solvents

Results of the additional solvent screening is summarized in Supplementary Table 2. In general, the hydrogenation proceeded in good yield in aqueous solvent system even in the absence of 2-propanol, suggesting that 2-propanol is unlikely to be the stoichiometric reductant in this hydrogenation (entries 2-5). In contrast, reactions in the absence of water suffered from low reactivity (entries 6-8). The hydrogenation did not proceed efficiently in 2-propanol or CH_3CN partly due to poor solubility of ascorbic acid in these solvents (entry 6,7). While ascorbic acid is sufficiently soluble in DMF, the hydrogenation in DMF was also sluggish (entry 8). It is tentatively assumed that the electrolytic dissociation of ascorbic acid might be beneficial for the catalytic turnover and its dissociation is more

enhanced in aqueous solvent compared to that in DMF. However, further studies are necessary to clarify the role of aqueous solvent in this reaction system.

Supplementary Table 2. Effect of other solvents in the hydrogenation of **1a**

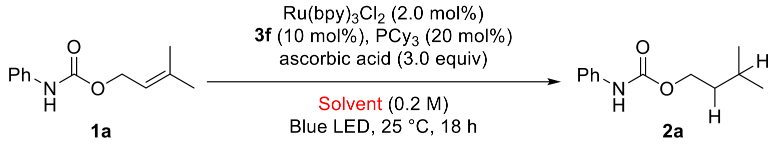

Entry	Solvent	NMR Yield of **2a** (%)
1	2-propanol/H_2O (3:1)	90
2	2-methyl-2-propanol/H_2O (3:1)	83
3	CH_3CN/H_2O (3:1)	85
4	DMF/H_2O (3:1)	70
5	acetone/H_2O (3:1)	79
6	2-propanol	0
7	CH_3CN	4
8	DMF	4

3-3. Effect of other acidic reducing agents

Considering the proposed mechanism in Figure 2, other electron rich organic acid might substitute ascorbic acid under the current conditions. To test this hypothesis, the cobalt and photoredox-catalyzed hydrogenation was attempted using phenol derivatives instead of ascorbic acid as described in Supplementary Table 3. While BHT did not promote the hydrogenation (entry 2), trace amount of **2a** was observed when gallic acid was employed as a reductant (entry 3). While the result in entry 3 indicates that other electron rich organic acid could be used instead of ascorbic acid, the observed reactivity was much lower compared to the hydrogenation using ascorbic acid (entry 1).

Supplementary Table 3. Effect of other acidic reducing agents in the hydrogenation of **1a**

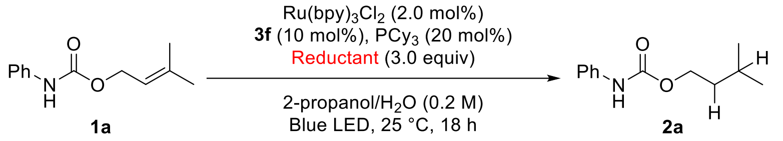

Entry	Reductant	NMR Yield of **2a** (%)
1	ascorbic acid	90
2	BHT	0
3	gallic acid	<5

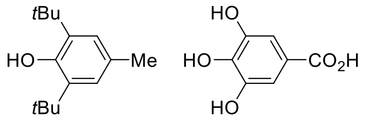

BHT gallic acid

4.Additional mechanistic studies

4-1. Stern-Volmer quenching experiment

In order to get insight into the photochemical process of the reaction, Stern-Volmer luminescence quenching analysis was conducted.

[Analysis using ascorbic acid]

Considering limited solubility of ascorbic acid in 2-propanol, the quenching study was conducted using aqueous solvent. To a solution of 50 μM of $Ru(bpy)_3Cl_2$ in mixed solvent (2-propanol/H_2O = 3:1) was added ascorbic acid under air so that the concentration of ascorbic acid was 0, 5, 10 or 15 mM. The four samples were transferred to a quartz cuvette and degassed by sparging argon for 20 minutes. The luminescence of each sample was measured at 613 nm using excitation light at 450 nm.

[Analysis using **1a** or tricyclohexylphosphine (PCy_3)]

To a solution of 50 μM of $Ru(bpy)_3Cl_2$ in 2-propanol (degassed by sparging argon for 20 minutes) was added **1a** or PCy_3 in an argon-filled glove box so that the concentration of **1a** or PCy_3 was 0, 5, 10 or 15 mM. The 8 samples were transferred to a quartz cuvette and removed from the glove box. The luminescence of each sample was measured at 613 nm using excitation light at 450 nm.

The I_0/I values (I_0: luminescence of the photocatalyst in the absence of the quencher, I: luminescence of the photocatalyst in the presence of the quencher) thus obtained was plotted as a function of a concentration of the quencher and the result is summarized in Supplementary Figure 1. These results verify that ascorbic acid quenches the excited $Ru(bpy)_3Cl_2$ while the substrate **1a** or PCy_3 does not react with the excited photocatalyst.

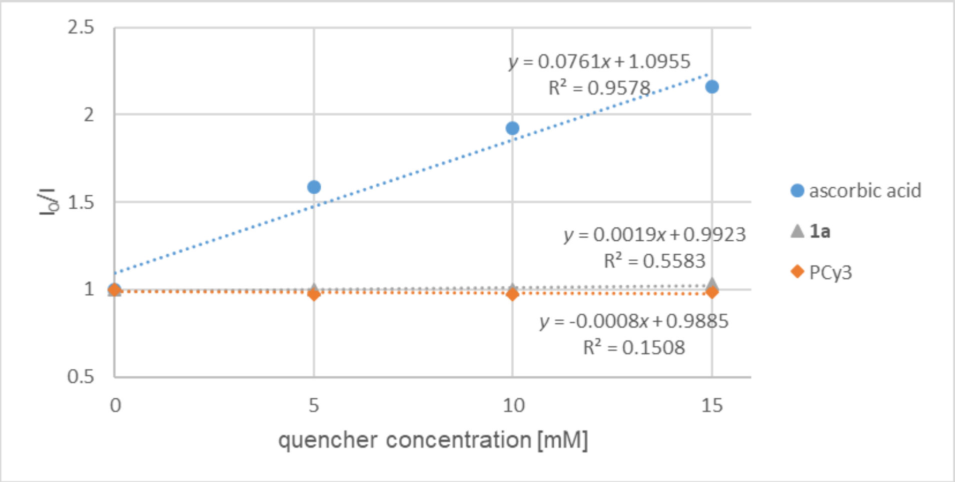

Supplementary Figure 1. Results of the Stern-Volmer luminescence quenching analysis. Quenching of the excited state of the photocatalyst was observed in the presence of ascorbic acid while quenching was not observed in the presence of **1a** or PCy_3.

[Difficulty in analyzing luminescence quenching with **3f**]

Luminescence quenching analysis using **3f** was not able to be conducted because **3f** has a strong absorption in visible light region (Supplementary Figure 2). The absorption at 450 nm (competes with the absorption of $Ru(bpy)_3Cl_2$) and 613 nm (decreases the observed luminescence derived from $Ru(bpy)_3Cl_2$) posed a challenge in quantitative analysis of the luminescence derived from the excited photocatalyst.

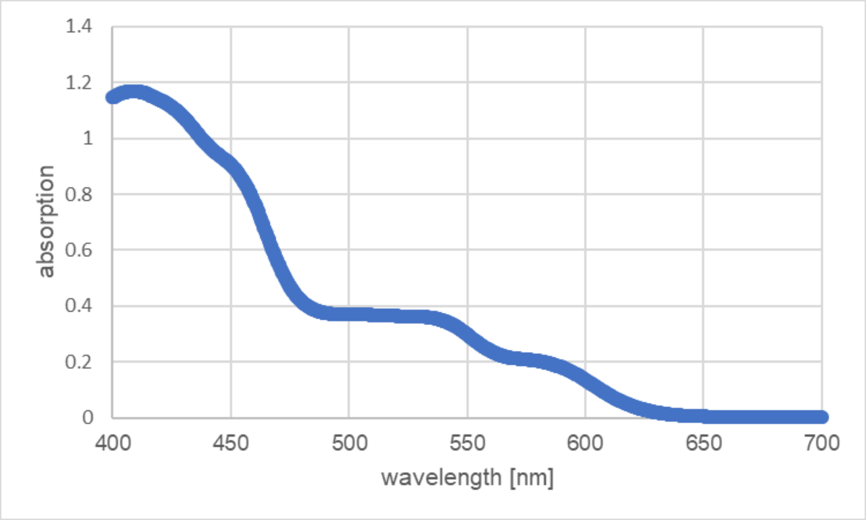

Supplementary Figure 2. Absorption of 3f (50 µM in 2-propanol) in visible light region. The absorbance at 450 nm and 613 nm disturbs Stern-Volmer quenching analysis using $Ru(bpy)_3Cl_2$.

4-2. Electrochemical analysis in the presence of a protic solvent

Our attempt to systematically analyze cyclic voltammograms of **3f** in the reaction solvent (2-propanol/H_2O = 3:1) was not successful due to interference of the background current presumably derived from the solvent. Instead, in order to assess the effect of a protic solvent in the electrochemistry of **3f**, cyclic voltammograms of **3f** were studied in the presence of a small amount of the protic solvent. As a result, addition of a small amount of 2-propanol and H_2O to a DMF solution of **3f** did not make an obvious change in its cyclic voltammogram (Supplementary Figure 3). This observation contrasts with the result obtained in Figure 3, in which ascorbic acid made a clear change in the voltammogram under the similar concentration presumably by facile protonation of the electrochemically generated Co^I. However, actual proton source that protonates Co^I in the reaction system cannot be clearly determined only by these electrochemical studies considering the large difference in stoichiometry of ascorbic acid and the protic solvents.

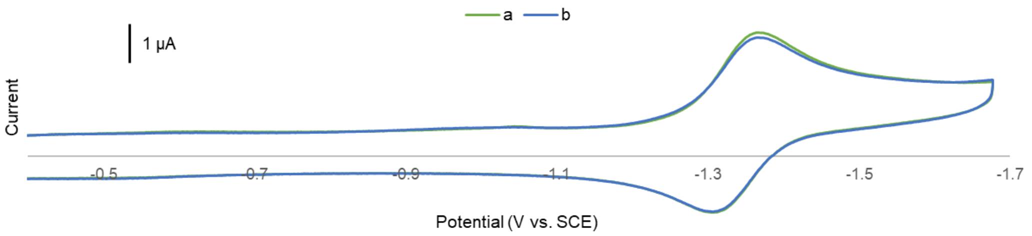

Supplementary Figure 3. Effect of a protic solvent for cyclic voltammogram of 3f. Recorded at 100 mV/s in a DMF solution of 3f (0.50 mM) and Bu_4NClO_4 (0.1 M). **a** In the absence of a protic solvent. **b** In the presence of 2-propanol (1.0 mM) and H_2O (0.33 mM). The potential was corrected using Fc/Fc^+ as an internal standard; SCE = saturated calomel electrode; Fc = ferrocene.

4-3. Detection of H_2 evolution

The possibility of hydrogen gas evolution from the reaction system was assessed by the parallel reaction (Supplementary Figure 4). The cobalt and photoredox-catalyzed reaction in the absense of alkene was conducted in one side of COware (two-chamber reaction vessel: http://www.sigmaaldrich.com/catalog/product/aldrich/744077?lang=ja&region=JP accessed on October 31st, 2020) while the other side of the vessel was charged with an alkene and Wilkinson's catalyst (see also Supplementary Figure 5 for the photograph of the reaction setup). The two reactions are connected through overhead space and only gaseous components can be transferred between the two sides. After 18 hours of irradiation, hydrogenation of cycloheptene proceeded in 5% yield. This result suggested that hydrogen gas is certainly released from ascorbic acid by the dual cobalt and photoredox catalysis while the efficiency of the evolution of H_2 is not very high.

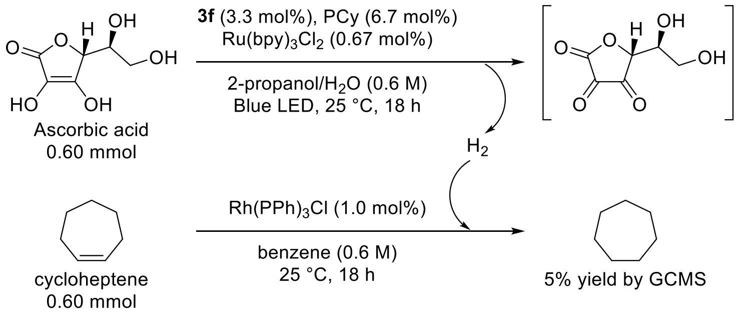

Supplementary Figure 4. Detection of H_2 gas by the parallel reactions using COware. The hydrogenation of cycloheptene supports the generation of molecular hydrogen from ascorbic acid by cobalt/photoredox catalysis.

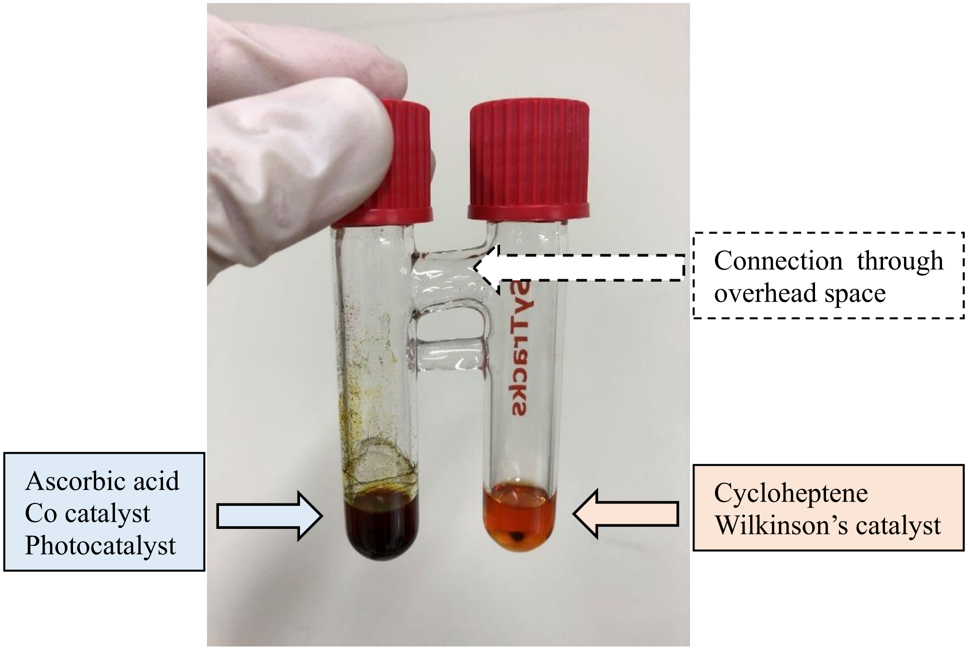

Supplementary Figure 5. The setup of the reaction using COware. Hydrogen gas is transferred from left side to right side through the overhead space.

[Procedures of the detection of H_2 evolution]

In an argon filled glove box, one side of COware was charged with ascorbic acid (106 mg, 0.60 mmol), **3f** (12.0 mg, 20 μmol), tricyclohexylphosphine (11.2 mg, 40 μmol) and Ru(bpy)$_3$Cl$_2$•6H$_2$O (3.0 mg, 4.0 μmol). The other side of the COware was charged with tris(triphenylphosphine)rhodium chloride (5.6 mg, 6.0 μmol), cycloheptene (58 mg, 0.60 mmol) and benzene (1.0 mL). The apparatus was capped and removed from the glove box. A mixed solvent (2-propanol/H_2O = 3:1, 1 mL) was added to the cobalt-photoredox side via syringe, and the syringe hole was carefully sealed with a vinyl tape. These two reactions share the same atmosphere so that only gaseous components can be transferred between sides. The benzene solution side was wrapped with aluminum foil, and the apparatus was placed in front of the light source (ca. 3 cm from two blue LED panels) in a cold room (4 °C) and the temperature of the reaction mixture was kept approximately at 25 °C. After stirring for 18 h, nonane was added to the reaction mixture of cycloheptene as an internal standard. The reaction mixture of rhodium-catalyzed hydrogenation was passed through a short pad of silica gel with Et_2O. The solution was subjected to GCMS analysis and the yield of cycloheptane was determined to be 5%.

4-4. Attempt of hydrogenation using H_2 gas

In order to assess whether hydrogen gas works as a reductant in the cobalt-photoredox catalyzed hydrogenation, hydrogenation of **1a** was attempted using the dual cobalt and photoredox system under hydrogen gas atmosphere in the absence of ascorbic acid (Supplementary Figure 6). As a result,

hydrogenation of **1a** did not proceed. This result indicates that hydrogen gas does not play a major role as a stoichiometric reductant in the current reaction conditions.

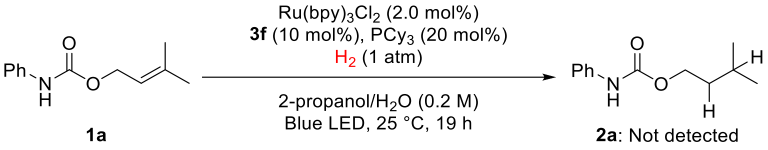

Supplementary Figure 6. Attempt of hydrogenation under H_2 atmosphere. No hydrogenated product **2a** was observed, which suggests that hydrogen gas is not likely to be an effective reductant in the cobalt/photoredox catalytic system.

[Procedures of attempt of hydrogenation under H_2 atmosphere]

In an argon filled glove box, a flame dried test tube was charged with **1a** (41 mg, 0.20 mmol), **3f** (12.0 mg, 20 μmol), tricyclohexylphosphine (11.2 mg, 40 μmol) and $Ru(bpy)_3Cl_2•6H_2O$ (3.0 mg, 4.0 μmol). The test tube was removed from the glove box and was charged with H_2 gas (balloon). A mixed solvent (2-propanol/H_2O = 3:1, 1 mL) was added to the tube via syringe and the tube was placed in front of the light source (ca. 3 cm from two blue LED panels) in a cold room (4 °C) so that the temperature of the reaction mixture was kept approximately at 25 °C. After stirring for 19 h, organic material was extracted with EtOAc (x 3) and the combined organic layer was washed with brine. The crude reaction mixture was analyzed by ^{1}H NMR using 1,1,2,2-tetrachloroethane as an internal standard.

5. Computational methods

5-1. General

In this study, the artificial force induced reaction (AFIR) method[30] implemented in the global reaction route mapping (GRRM17) program[31,32] was employed to locate transition states (TSs) of the hydrogen atom transfer step. Initially, the reactions between the cobalt hydride and isobutene were systematically explored using the single-component mode of the AFIR method (SC-AFIR). The SC-AFIR search was done with the GFN2-xTB method[33] implemented in the Orca program[34] and the collision energy parameter γ of the AFIR method was set to 200 kJ/mol. Thus, the approximate TS structure related to hydrogen atom transfer was obtained and optimized to actual TS employing UωB97X-D density functional with inclusion of the solvent effects of 2-propanol by the PCM method, and basis functions LANL2DZ(f) (LANL2DZ with added *f* polarization function α = 2.780) for Co and 6-31G(d,p) for the others. The intrinsic reaction coordinates (IRC) analysis was then conducted for the TS. At obtained stationary structures, Gibbs free energy values were estimated through the harmonic vibrational analysis. Finally, single point energies were estimated using UωB97X-D density functional with inclusion of the solvent effects of 2-propanol by the PCM method, and basis functions SDD for Co and 6-311G(d,p) for the others. All the electronic structure calculations in the TS optimization, IRC calculation, harmonic vibrational analysis, and single point energy calculation were done by Gaussian 16 program.[35] The computed structures were visualized by Avogadro 1.2.0.[36,37]

5-2. Discussion regarding the description of radical pairs

From the theoretical standpoint, ab initio multireference theory taking account of both statical and dynamical electron correlations like CASPT2 would provide more precise description of reactions involving the generation of radical pairs compared to DFT. However, such methods are not readily applicable to realistic systems containing transition metals because of their high computational cost. Thus, DFT with broken-symmetry formalism, which has provided reasonable energy profiles in the relevant theoretical investigations,[38,39] was employed in this study. In practice, all the unrestricted DFT calculations were performed in singlet state using stable=opt option in order to guarantee stability of wavefunctions throughout the calculation. The change of biradical character during the reaction might be reflected in the computed $\langle S^2 \rangle$ values (SM: $\langle S^2 \rangle = 0.00$, TS: $\langle S^2 \rangle = 0.33$, PD: $\langle S^2 \rangle = 1.01$). However, these $\langle S^2 \rangle$ values do not strictly correspond to actual spin states of these species and precise interpretation of the change of spin states during the hydrogen atom transfer process is beyond the scope of this study.

5-3. Computed structures and electronic energies

Described below are Cartesian coordinates and electronic energies of TS and optimized local minima found as terminal points of IRC paths obtained by calculations using UωB97X-D, LANL2DZ(f) for Co and 6-31G(d,p) for the others, and PCM (2-propanol).

TS (-1963.13937590)

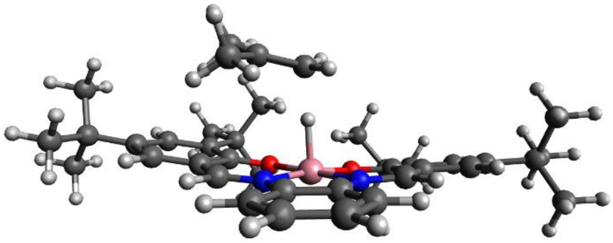

C	-1.686819926468	1.339538921546	-1.832287397843
H	-1.720455035277	2.077705998649	-1.034109661170
C	-0.950631243220	1.589674575529	-2.958953840378
C	-1.061160118102	0.719088873531	-4.174638949440
H	-0.068521511527	0.395985306923	-4.510712670633
H	-1.674145231049	-0.167352001304	-3.991077615872
H	-1.503927435009	1.280226564843	-5.007422160552
C	0.053320298775	2.700565295766	-3.021759777990
H	-0.167719986147	3.366717073795	-3.865165170133
H	0.065091092695	3.292046977829	-2.104114629024
H	1.062816706575	2.303201038790	-3.188434489901
H	-2.525970871663	0.648655503111	-1.880713564892
C	-2.591636428853	-1.792126091773	0.790325842549
H	-3.494319621661	-2.391823965721	0.674875275423
N	-1.690340971320	-1.832758750473	-0.149007291882
C	-1.845181345716	-2.643159076815	-1.300493879209
C	-0.808720129515	-2.512063904783	-2.241476241736
C	-2.899525727971	-3.525363255688	-1.548833353425
H	-3.703611382998	-3.651945578937	-0.833374210768
C	-0.842225317477	-3.263963272363	-3.418774187280
C	-2.924717267555	-4.264874443086	-2.723785451281
H	-0.051110882064	-3.184975118262	-4.155146294783
H	-3.746212245637	-4.948652852336	-2.908227351381
C	-1.897243644939	-4.134669323526	-3.657140180999
H	-1.914610810270	-4.716058179496	-4.572658463319
C	-2.513649129353	-1.040370247365	1.997592310427
C	-3.612949322407	-1.137425126965	2.883907981256
C	-1.363520307584	-0.265808353881	2.345467479907
H	-4.465851200714	-1.733383407173	2.565300983543
C	-3.620851883606	-0.512689170771	4.108036492862
C	-1.342256621817	0.349470900635	3.646395391023
C	-2.455445710364	0.212613950168	4.453795963376
H	-2.434211368766	0.691890067028	5.423354960903
C	1.155048607028	-1.252390614271	-2.690327280339
H	1.218702698019	-1.757112872684	-3.654293685543
N	0.190386725514	-1.578118276142	-1.878672924944

C	2.135780424252	-0.246864070282	-2.468592227975
C	2.215858234537	0.497386855445	-1.252305359982
C	3.013475875537	0.029894020269	-3.550039975417
H	2.899067480603	-0.564961839743	-4.451475021048
C	3.215815827472	1.535437950515	-1.166435437760
C	3.959739862575	1.018003338745	-3.476751737383
C	4.027521417471	1.745788416308	-2.258703780924
H	4.770032587873	2.533331313445	-2.196648842740
O	-0.358859058261	-0.117654987496	1.546173091511
O	1.435554258769	0.278755866130	-0.249131670270
C	-0.099620958440	1.123685512369	4.114004626652
C	-4.837245244059	-0.626391078749	5.038634004713
C	3.297627416709	2.428205777372	0.081265277583
C	4.907837416536	1.380218547231	-4.623274664330
C	-5.086018913105	-2.107409920299	5.382866094208
H	-4.216307617903	-2.539295219730	5.888565608370
H	-5.951962732957	-2.205122310999	6.047065259410
H	-5.284713025430	-2.702160581294	4.486087415786
C	-6.079261557169	-0.053141149853	4.329620889472
H	-6.300508472101	-0.594446880221	3.404697203675
H	-6.958601604093	-0.126988836225	4.979153201010
H	-5.928726725818	1.001179112693	4.075753543396
C	-4.640209119888	0.142863285892	6.352958193439
H	-4.483119392252	1.212419830221	6.178546006981
H	-5.534280103283	0.036462929869	6.975348738952
H	-3.790320620065	-0.241674917013	6.926452987106
C	1.114781366401	0.173174153554	4.144951464611
H	2.011625631481	0.720745589310	4.456803179683
H	0.947587275627	-0.640032562164	4.860106833452
H	1.297323191429	-0.255795015612	3.158800199511
C	0.186343704573	2.297819264304	3.158696720095
H	0.385364165042	1.933426004727	2.152133678982
H	-0.665347066282	2.987034780793	3.128521824774
H	1.063326350046	2.857662979125	3.504372645265
C	-0.272782238565	1.701848521267	5.526887718548
H	0.642013410405	2.232778846340	5.808986455197
H	-1.099342268305	2.418807382416	5.579199997916
H	-0.444238491907	0.920449469732	6.275112648502
C	3.476043998912	1.584105780863	1.359151540789
H	3.572447691959	2.245697476351	2.227730789288
H	2.622521054167	0.926424288598	1.516797318231
H	4.386834389279	0.977776291163	1.294091311757
C	4.476568498223	3.411727363738	0.013434796512
H	4.493730472994	4.011442078221	0.928985680687
H	5.437962247987	2.891949635721	-0.062452895913
H	4.390684678043	4.105875751299	-0.829200492973
C	2.000961522693	3.257616821751	0.171757654297
H	1.123132248677	2.609112420079	0.168468915953
H	1.991269701971	3.849506680867	1.093908467405
H	1.932177787704	3.946249306901	-0.677506292351
C	6.367955131684	1.213641650958	-4.160530237216
H	6.600008947507	1.857130611735	-3.306379738395
H	6.564982538012	0.177723177126	-3.866174875152
H	7.056144863183	1.476342609385	-4.971410251559

C	4.687455164720	0.488377517289	-5.852310391178
H	4.864085528527	-0.567501673463	-5.622137262951
H	3.671656437293	0.590289683043	-6.248427538164
H	5.383680050312	0.776942421237	-6.645971886163
C	4.666408587625	2.844244339243	-5.040185211343
H	3.632885031436	2.985094691982	-5.373409649301
H	4.851737636397	3.537807100347	-4.214228771149
H	5.333586289001	3.121123083304	-5.863850931485
Co	-0.135516757865	-0.753753212129	-0.207261770439
H	-0.878976840762	0.333006805737	-0.970223859694

SM = Optimized local minimum of the backward IRC from TS (-1963.15448942)

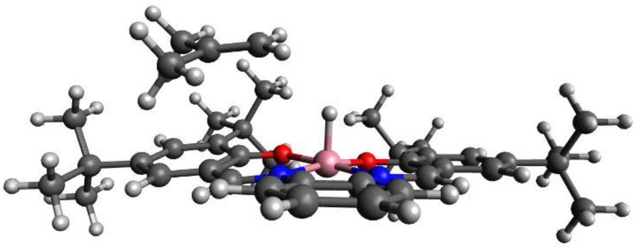

C	-1.220197436691	1.658330506056	-3.104292112738
H	-1.296597388524	2.388642096485	-2.303192295954
C	-0.257045463925	1.731003792706	-4.024476431594
C	-0.142305272707	0.727274745328	-5.140550833073
H	0.864921591223	0.294817674568	-5.165998093385
H	-0.868505572155	-0.083907307001	-5.034467453839
H	-0.301338648586	1.208095564719	-6.113391961307
C	0.782778355361	2.818576851588	-4.019253481052
H	0.776216578437	3.369914920634	-4.967626849095
H	0.622406722426	3.528245005473	-3.203730118045
H	1.783590890165	2.385914877254	-3.906031364225
H	-1.951756358635	0.854417958403	-3.109470180779
C	-2.584562421612	-1.925490232705	0.974984580996
H	-3.419432714695	-2.622685671469	0.915851949391
N	-1.800549778716	-1.809002020906	-0.059233161431
C	-2.038950812656	-2.512768498639	-1.266222145433
C	-1.081850062878	-2.272050475212	-2.264896430552
C	-3.117568609993	-3.359858445142	-1.527949463178
H	-3.873226324962	-3.556156098464	-0.776413305351
C	-1.225818680226	-2.858321527083	-3.524357483465
C	-3.239986349643	-3.957088718925	-2.775913661761
H	-0.519037328288	-2.653076305535	-4.320157985457
H	-4.081289624489	-4.612104192819	-2.974252018942
C	-2.299118184082	-3.703583459437	-3.773262849625
H	-2.407406779144	-4.156561037976	-4.752721210518
C	-2.463885778421	-1.221036425766	2.204654766557
C	-3.454411355065	-1.466324691280	3.185594287899
C	-1.389148705094	-0.318667592116	2.464864335405
H	-4.252949317452	-2.160888683460	2.933824622841
C	-3.426384879102	-0.853823523731	4.415400680079
C	-1.335752927614	0.306936230463	3.756975125559
C	-2.342925006151	0.023100829255	4.660323723966

H	-2.298977637850	0.505213263526	5.627555458336
C	0.999805583833	-1.184850514299	-2.620593824926
H	1.109434440337	-1.777144476801	-3.528692946170
N	-0.050546031299	-1.387561771903	-1.876245210731
C	2.022742338031	-0.231417498197	-2.382800466029
C	2.008398518797	0.626934350579	-1.245434584468
C	3.036384929848	-0.116843705629	-3.369601025774
H	3.000351469638	-0.807128182616	-4.207192375467
C	3.014408882612	1.657002296646	-1.168073898207
C	4.014104946704	0.839457434431	-3.287754231029
C	3.960988738071	1.711322755910	-2.167261696064
H	4.720366652785	2.482654801291	-2.106820527889
O	-0.465915531359	-0.068712908883	1.590633759701
O	1.149913449592	0.501540017892	-0.287612442606
C	-0.170449537714	1.241703983843	4.117172343009
C	-4.524193361555	-1.129393762389	5.453269865401
C	2.993396613818	2.670270652232	-0.014591204639
C	5.106974316329	1.033150485156	-4.342122546919
C	-4.553170655360	-2.633998601344	5.783381835575
H	-3.592198316364	-2.959660064143	6.194541148049
H	-5.332330091211	-2.846683333390	6.523622286041
H	-4.762940163211	-3.238461607942	4.895699167408
C	-5.889322646594	-0.707443757010	4.876933505406
H	-6.129918607846	-1.261573868324	3.964553763177
H	-6.686079193124	-0.898073117039	5.604401078382
H	-5.894898856927	0.360247576344	4.634862825330
C	-4.297675575192	-0.357327730542	6.761118320040
H	-4.298481482569	0.725718955574	6.599867567066
H	-5.104173840704	-0.587606635533	7.464248073261
H	-3.352329022092	-0.634711425034	7.239121437439
C	1.146449991950	0.439631428298	4.089258099590
H	1.994100640776	1.099012799438	4.307508101783
H	1.127167562728	-0.352689972372	4.845813693354
H	1.306359432194	-0.013274011900	3.110061182427
C	-0.094993193814	2.414695277667	3.119631371514
H	0.076664958899	2.054428872974	2.106129318774
H	-1.024252705899	2.995297955819	3.140058813127
H	0.728757850989	3.083868711871	3.393421450539
C	-0.320131380698	1.839513608192	5.524716422252
H	0.533392717519	2.494749571618	5.725424102697
H	-1.229639030502	2.442595546964	5.619729209689
H	-0.332196922997	1.068877688096	6.302919531688
C	3.141181258426	1.944812687633	1.337108731762
H	3.142388056967	2.675196525477	2.154387869732
H	2.318018188995	1.250103398680	1.497891833560
H	4.087250431094	1.392816865775	1.374691591614
C	4.134677487855	3.692875299972	-0.121639992069
H	4.062719094487	4.395630139052	0.714470065564
H	5.119547424100	3.216251412993	-0.068355246347
H	4.080851603408	4.275672901268	-1.047483764722
C	1.663331596993	3.449921791915	-0.053105880795
H	0.811082805640	2.771025705036	-0.005143571680
H	1.606890442851	4.139012129674	0.797116945414
H	1.593716920922	4.039121361385	-0.974169269264

C	6.492686081430	0.886897455483	-3.685169517487
H	6.646638768054	1.624530862816	-2.891654218770
H	6.610565038251	-0.109715195588	-3.247621925501
H	7.283169717898	1.029832094875	-4.429914958406
C	4.997953447926	0.003329790617	-5.474322386077
H	5.099477894396	-1.020848813058	-5.100420156258
H	4.042558843381	0.085110608167	-6.003446294194
H	5.796429877275	0.172324709864	-6.203411188586
C	4.975262338437	2.440373569841	-4.956732082592
H	3.996302663825	2.563595903184	-5.431814121278
H	5.085078845832	3.225591863429	-4.202522769522
H	5.748176413127	2.597726951756	-5.716929336901
Co	-0.346831876594	-0.613509778757	-0.186578066167
H	-1.212831983051	0.354738145441	-0.727030187303

PD = Optimized local minimum of the forward IRC from TS (-1963.16925165)

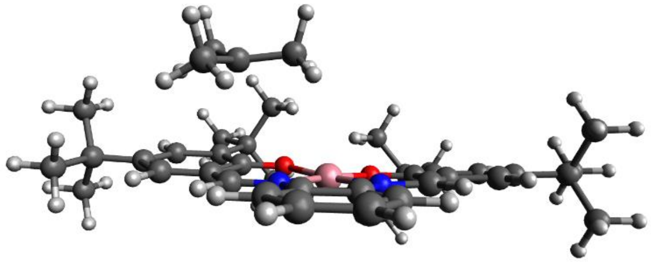

C	-1.629988186360	1.468773776136	-2.576554186259
H	-2.460832569425	2.134585524326	-2.868367838598
C	-0.517221776286	1.481568083406	-3.572774199156
C	-0.630898624914	0.647101901684	-4.807263155276
H	0.358026896387	0.421802470947	-5.226141388907
H	-1.146469093045	-0.300254164942	-4.609156520749
H	-1.200212077320	1.158420388718	-5.602593927710
C	0.463696438969	2.607672196406	-3.559269308967
H	0.110952248287	3.469807580171	-4.151665265666
H	0.643507741889	2.966829203762	-2.541056381859
H	1.428085397099	2.300167525382	-3.981815546096
H	-2.056180963425	0.464275196347	-2.462661081401
C	-2.620121981322	-1.813740334821	0.882772180226
H	-3.513510216170	-2.429706794914	0.782257609803
N	-1.767056920505	-1.789116272032	-0.102132380528
C	-1.962372085573	-2.540145702066	-1.289454888560
C	-0.967660033318	-2.348774157888	-2.265585243880
C	-3.023900113058	-3.411586948866	-1.542300884193
H	-3.794815389187	-3.584618864308	-0.800611406597
C	-1.062034200727	-3.016372965091	-3.489429243247
C	-3.103485917340	-4.075391863004	-2.759592315490
H	-0.316501048324	-2.873359586343	-4.262829466534
H	-3.932146271014	-4.749142644300	-2.948400801942
C	-2.125899594982	-3.875312231749	-3.732740107082
H	-2.190688693745	-4.389474554937	-4.685520069818
C	-2.505764556459	-1.109897143447	2.115887133724
C	-3.552520688181	-1.291047112210	3.052031799107
C	-1.387868339069	-0.274771910817	2.428324220706

H	-4.382344831799	-1.932241842377	2.761872279693
C	-3.538850204250	-0.690652217200	4.287955480973
C	-1.350912621377	0.334653502117	3.730707286784
C	-2.411622027805	0.111165813208	4.587970809318
H	-2.377643852682	0.580762442108	5.561827656913
C	1.056301296299	-1.165458788106	-2.675017354932
H	1.123935916680	-1.681384530834	-3.632220525799
N	0.055353382412	-1.445511878106	-1.888255029137
C	2.081738050573	-0.212270401178	-2.430826771453
C	2.112573658702	0.598396502308	-1.257153911114
C	3.034666510978	-0.027347210909	-3.466186943995
H	2.954902933383	-0.668258485533	-4.339290704063
C	3.131256892673	1.615339150768	-1.168210255642
C	4.008619858626	0.933599835937	-3.386775050180
C	4.020102614274	1.733956846687	-2.213831404627
H	4.784177571216	2.500341897226	-2.147809303315
O	-0.425588274364	-0.057294909221	1.592274872431
O	1.268288379522	0.459505857836	-0.289582018356
C	-0.144052373694	1.190771676048	4.148037233950
C	-4.693610778910	-0.906775591509	5.277135019800
C	3.178034035610	2.555623726848	0.046364081991
C	5.039646044028	1.196625865730	-4.487855320385
C	-4.815088403051	-2.407517203932	5.604191597588
H	-3.890646010126	-2.782045461527	6.055650797967
H	-5.635235481805	-2.579592219439	6.310063906432
H	-5.016835842222	-3.000684599255	4.707013434212
C	-6.009671033369	-0.415271086628	4.644655299731
H	-6.240377361024	-0.956755631298	3.722185494698
H	-6.845337756753	-0.563920428162	5.337502966201
H	-5.949887302476	0.651129630923	4.404091465747
C	-4.483578915091	-0.146231878485	6.594484686914
H	-4.414919860165	0.934760164651	6.433803866685
H	-5.333116651177	-0.328771426881	7.260026023918
H	-3.578656284333	-0.476039127587	7.115433254231
C	1.126464576974	0.316472469808	4.144423495222
H	1.998968186640	0.920254006267	4.419018043803
H	1.033617761020	-0.497353046165	4.872215779111
H	1.300831724102	-0.111924650138	3.156656319433
C	0.036747877483	2.375614195217	3.178485361587
H	0.229878956002	2.022985515034	2.166132414856
H	-0.858406292153	3.007961704795	3.172752726345
H	0.884808090712	2.992407546831	3.498004297946
C	-0.305393125938	1.769176207429	5.562450385621
H	0.579472433611	2.365563802462	5.806477193651
H	-1.178332152261	2.426384790637	5.640063662911
H	-0.393137184474	0.985422542885	6.322524944178
C	3.325923589529	1.747667135456	1.351322358471
H	3.391097930682	2.431454651944	2.205544310030
H	2.471689921334	1.088590547620	1.501253824727
H	4.242062487578	1.146511157890	1.328663389922
C	4.359117557248	3.535662151831	-0.026432713989
H	4.345129627460	4.177734832677	0.860009329301
H	5.322763357453	3.014918858701	-0.043678682016
H	4.301935409769	4.187780694169	-0.904510378067

C	1.882088076545	3.390318115469	0.090963714721
H	1.002391990784	2.744722632461	0.103464375855
H	1.866060722831	4.014289370658	0.991818534200
H	1.823844071804	4.051764456700	-0.780644376825
C	6.460936157681	1.014063672091	-3.921989117715
H	6.660298992782	1.701471448608	-3.094176784861
H	6.603039475653	-0.007347994192	-3.554352225901
H	7.207876242180	1.205227004736	-4.700248258290
C	4.866436739164	0.239489802585	-5.674571009054
H	4.991495711225	-0.805586651629	-5.372518280320
H	3.881279484491	0.348830026673	-6.140235851487
H	5.620402195503	0.458157893065	-6.437193761332
C	4.875512225545	2.639135836472	-5.005068734300
H	3.871736617915	2.789730358156	-5.416145114552
H	5.028267592431	3.375855178106	-4.210438956583
H	5.604876062949	2.845948590152	-5.795985676386
Co	-0.206865139441	-0.704952689683	-0.161200736062
H	-1.284735458881	1.810096372595	-1.592687720661

^{1}H NMR spectrum of **1b**

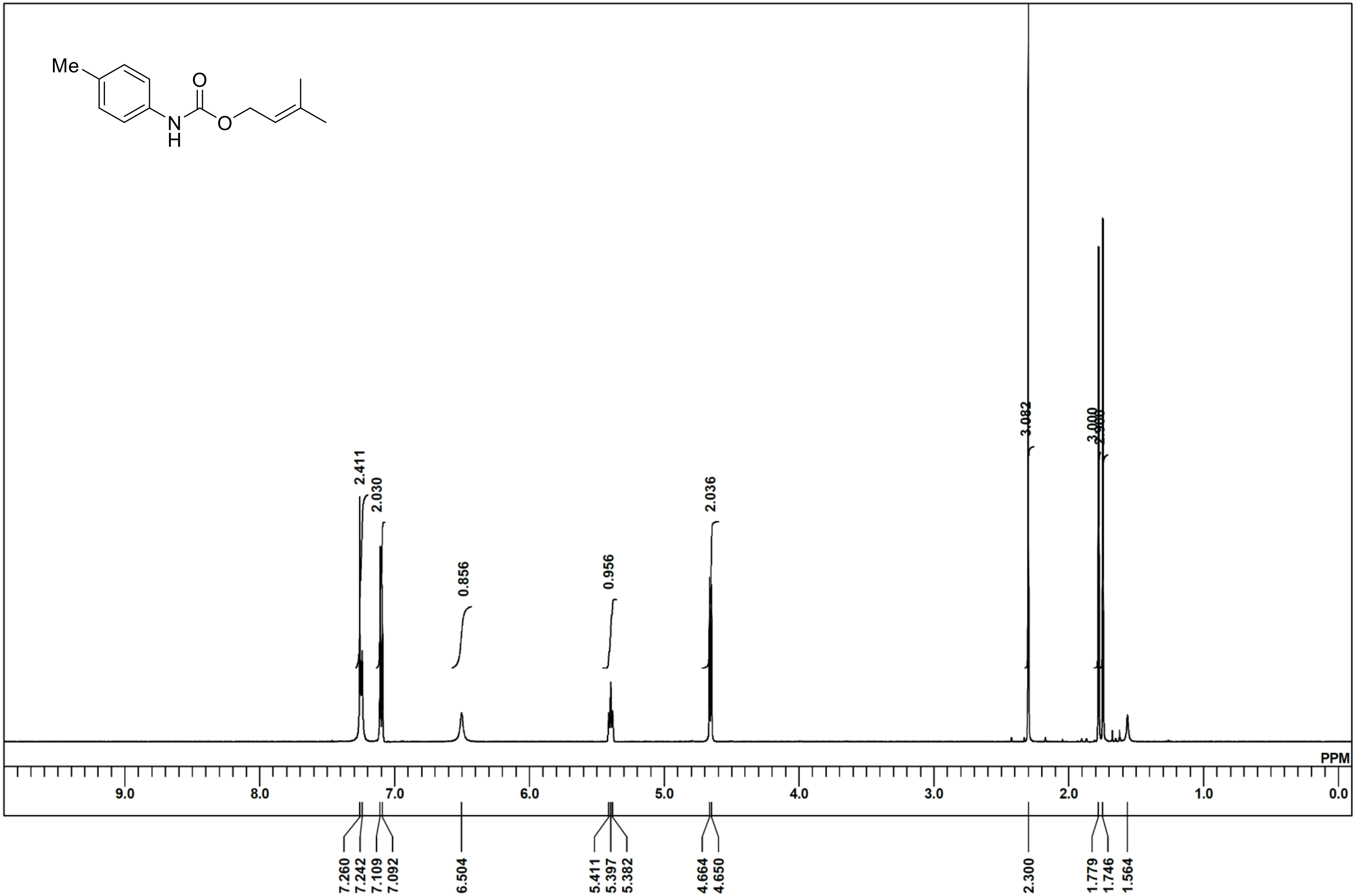

13C NMR spectrum of **1b**

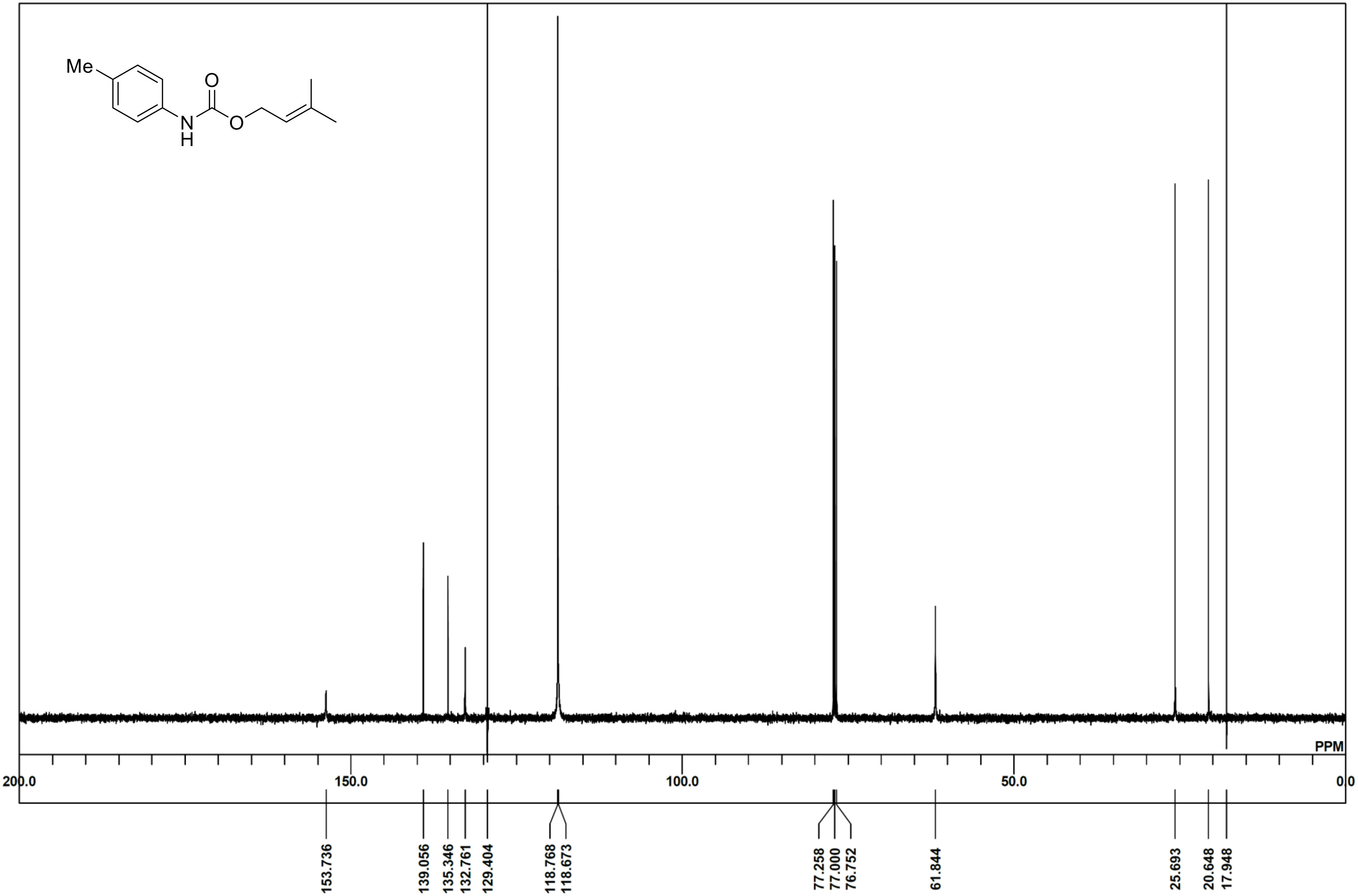

^{1}H NMR spectrum of **1d**

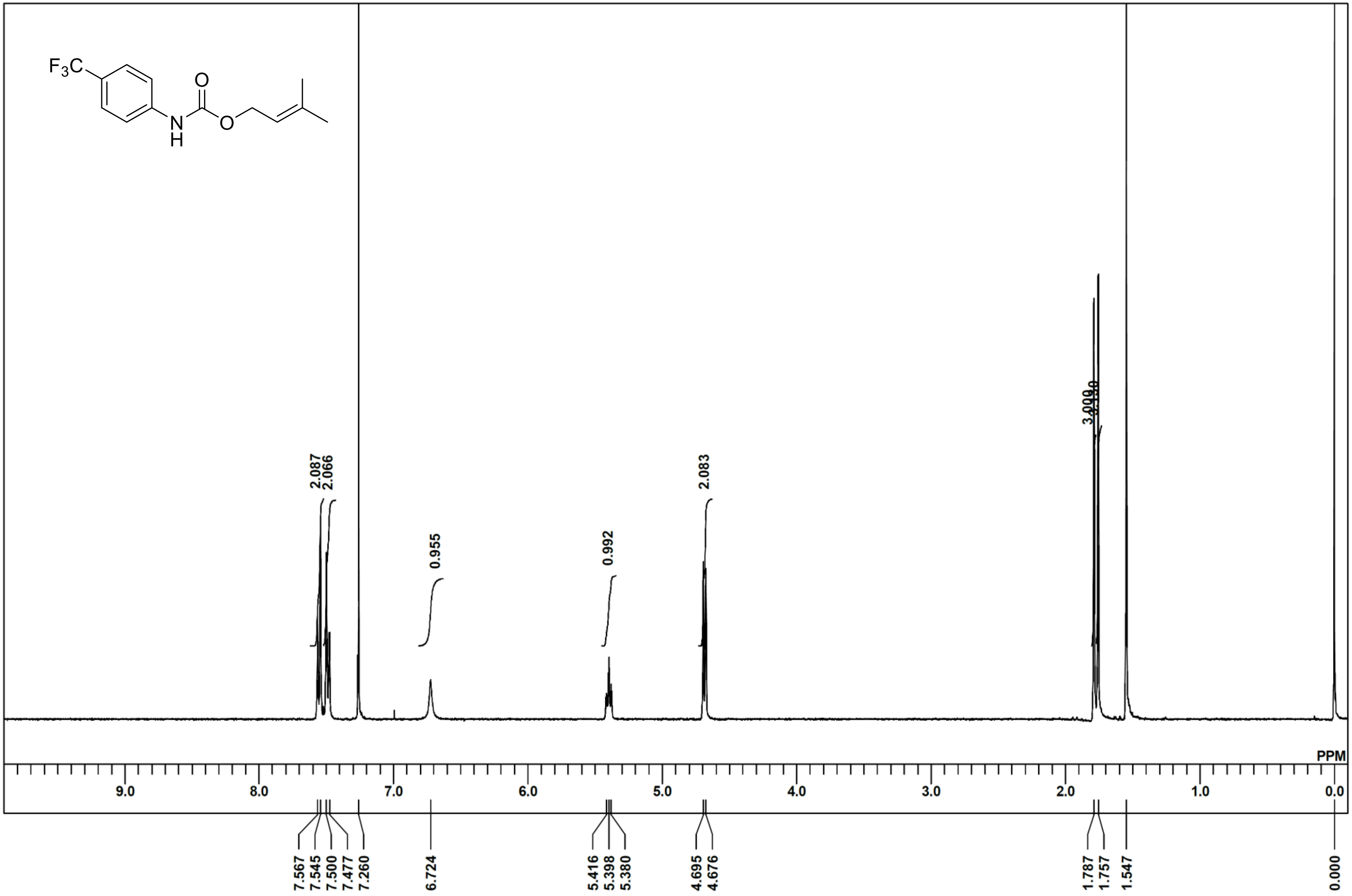

^{13}C NMR spectrum of **1d**

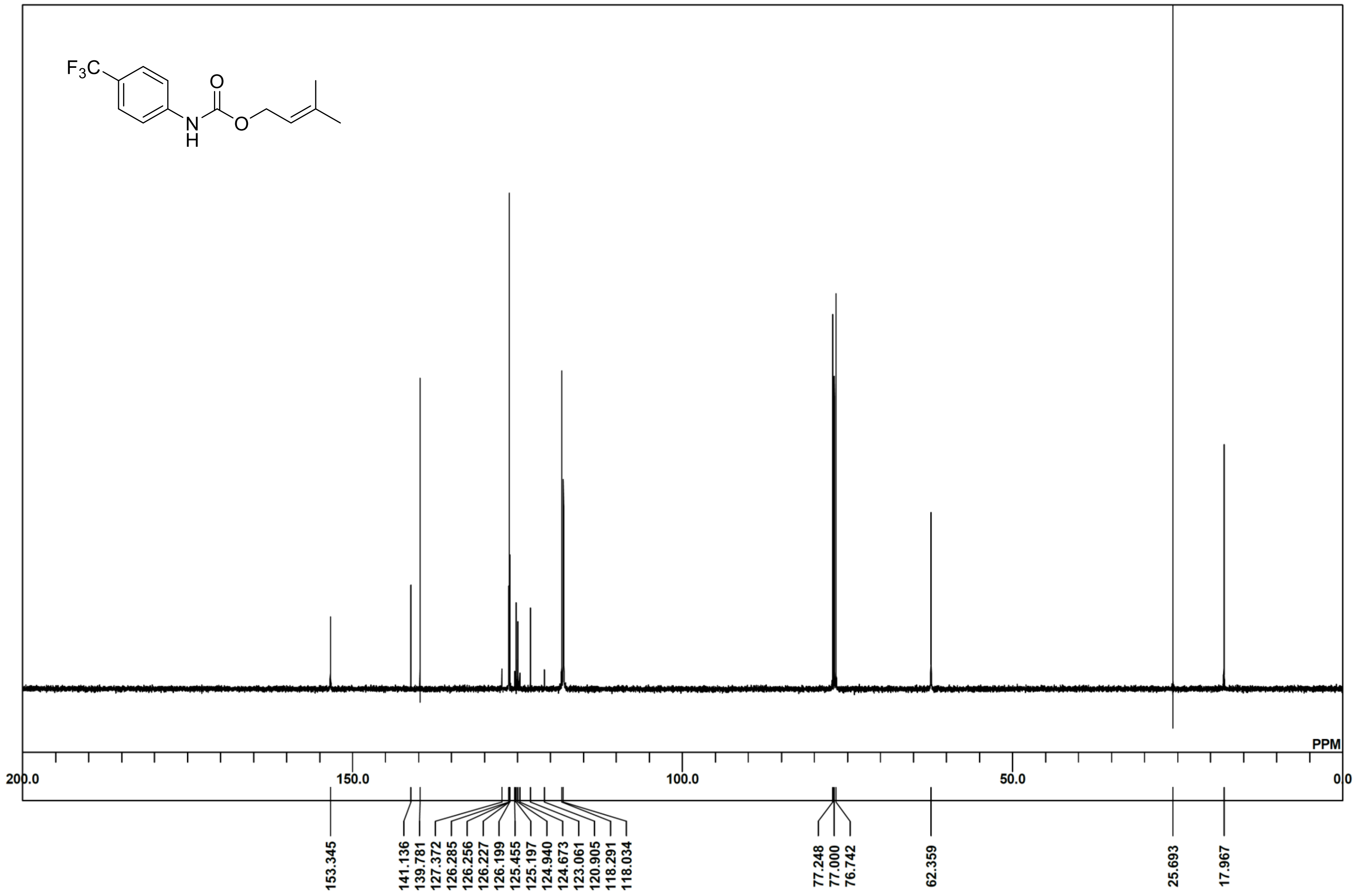

^{19}F NMR spectrum of **1d**

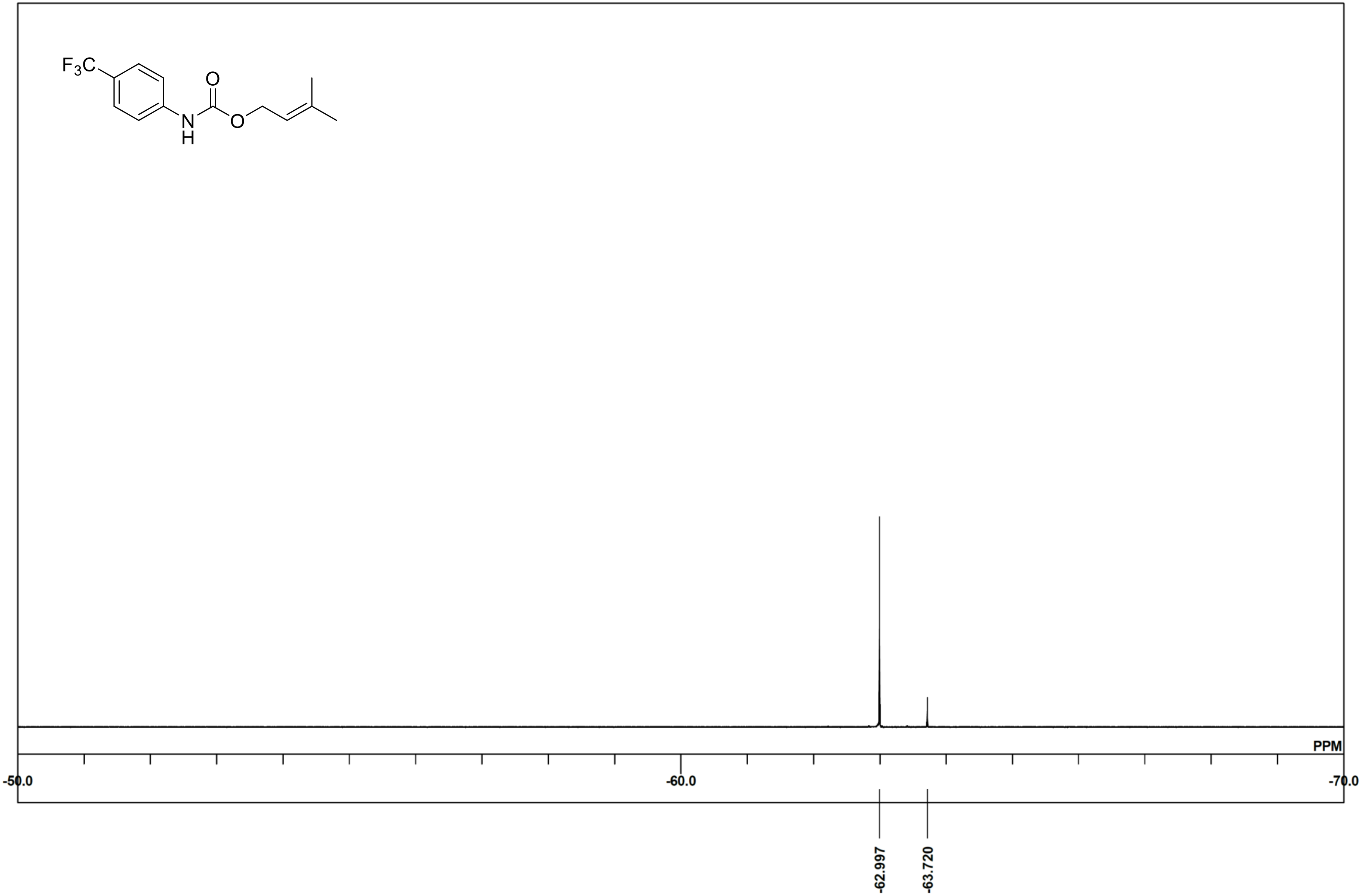

^{1}H NMR spectrum of **1f**

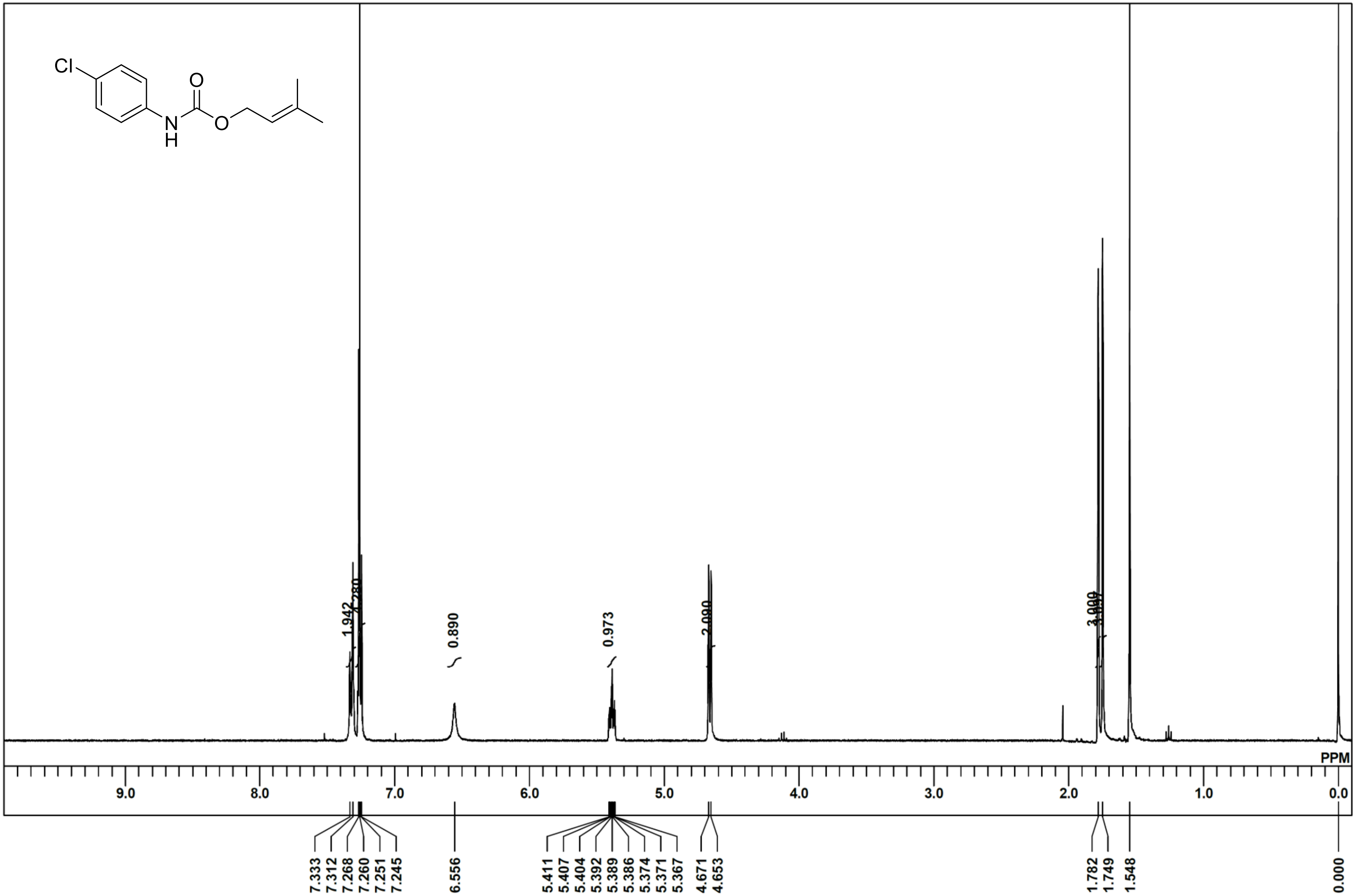

^{13}C NMR spectrum of **1f**

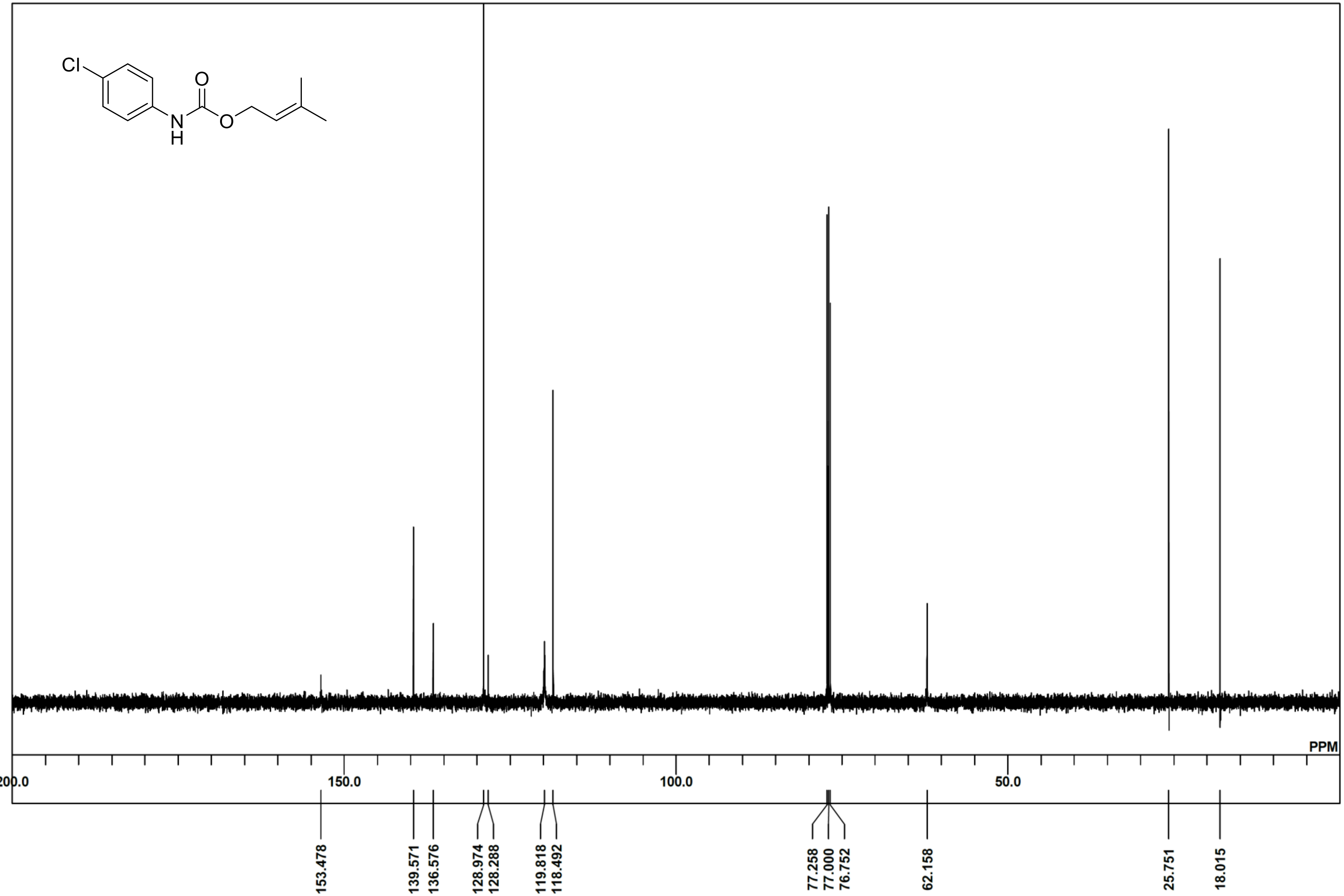

¹H NMR spectrum of **1g**

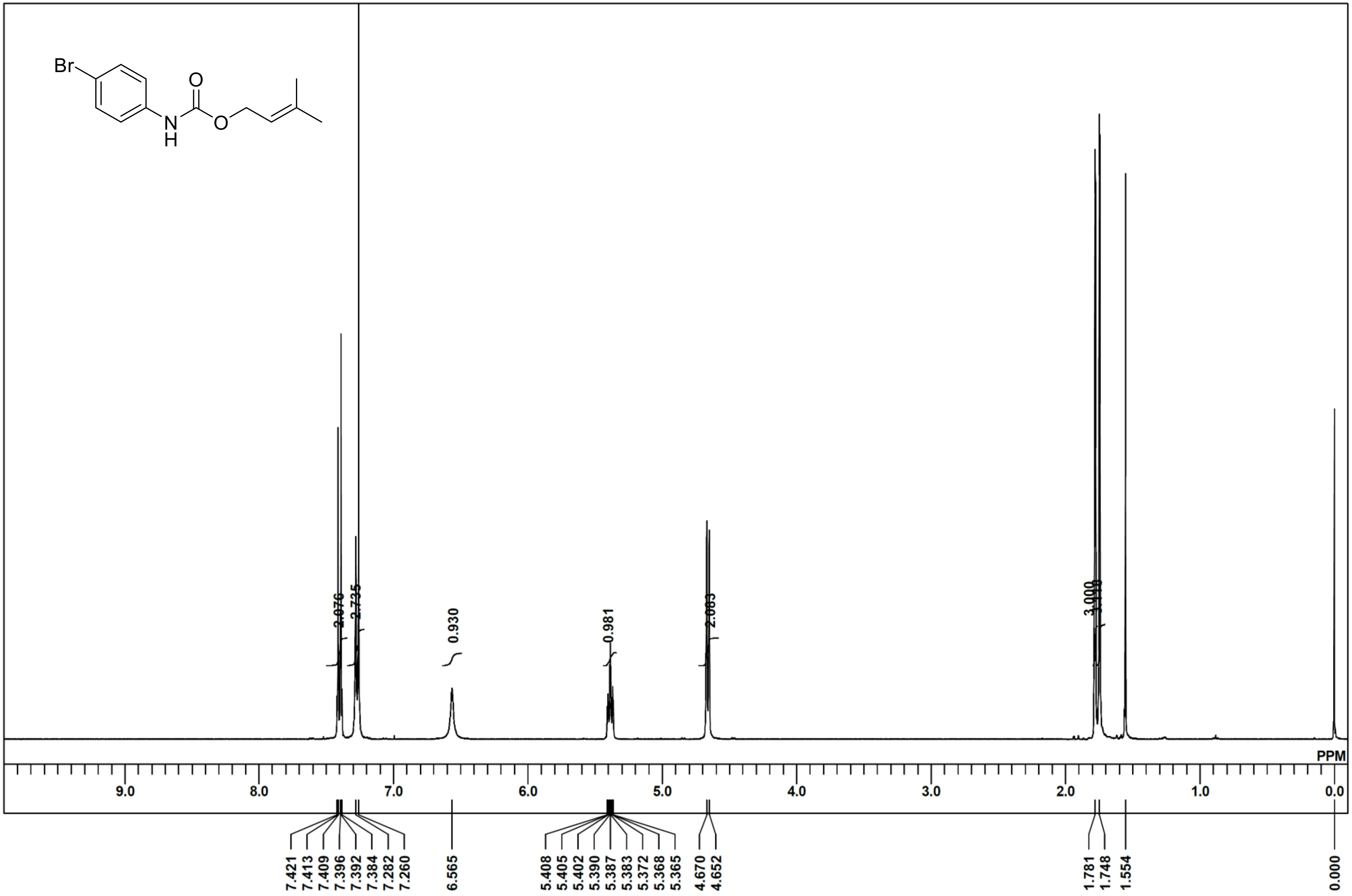

^{13}C NMR spectrum of **1g**

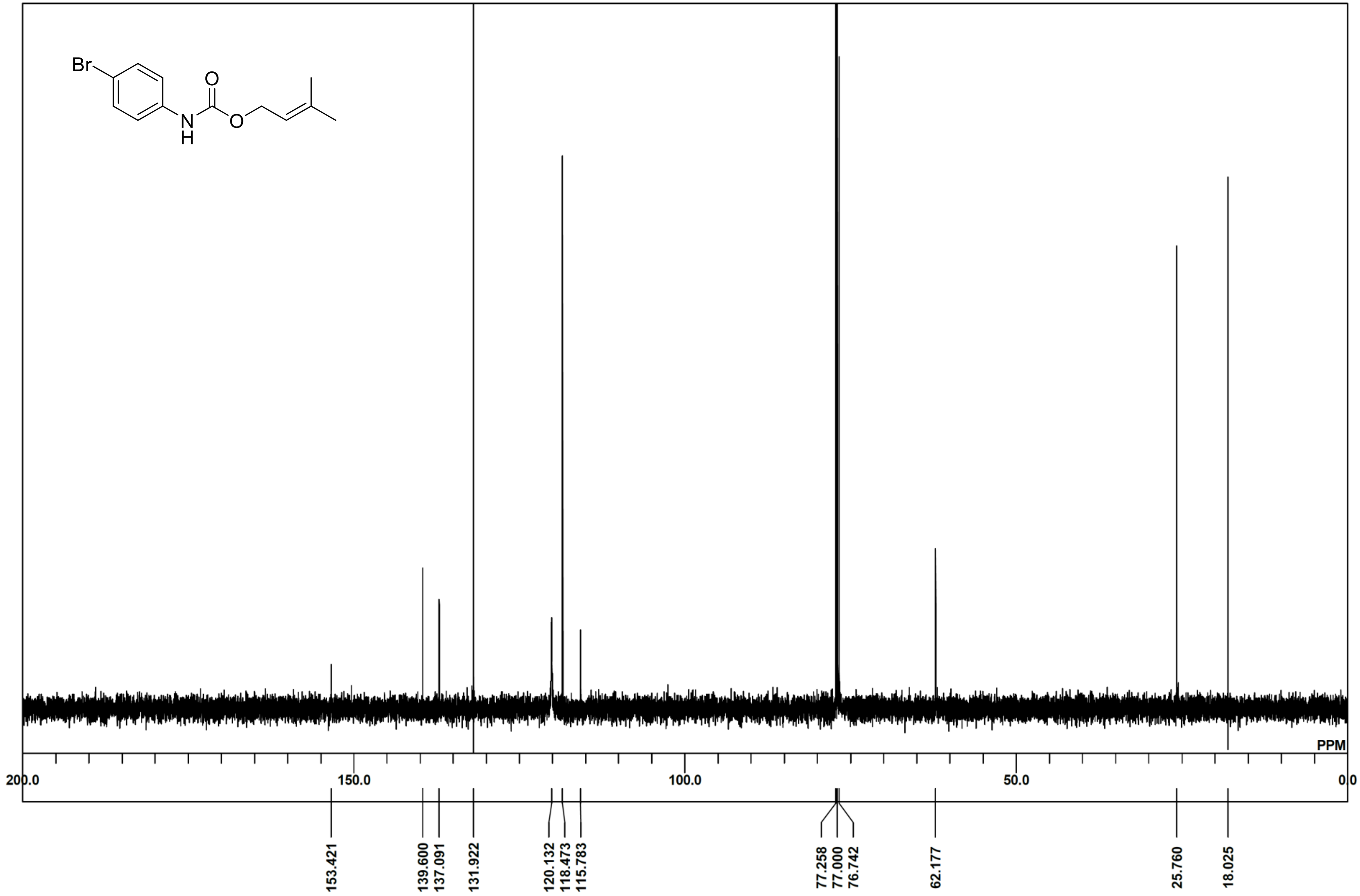

^{1}H NMR spectrum of **1h**

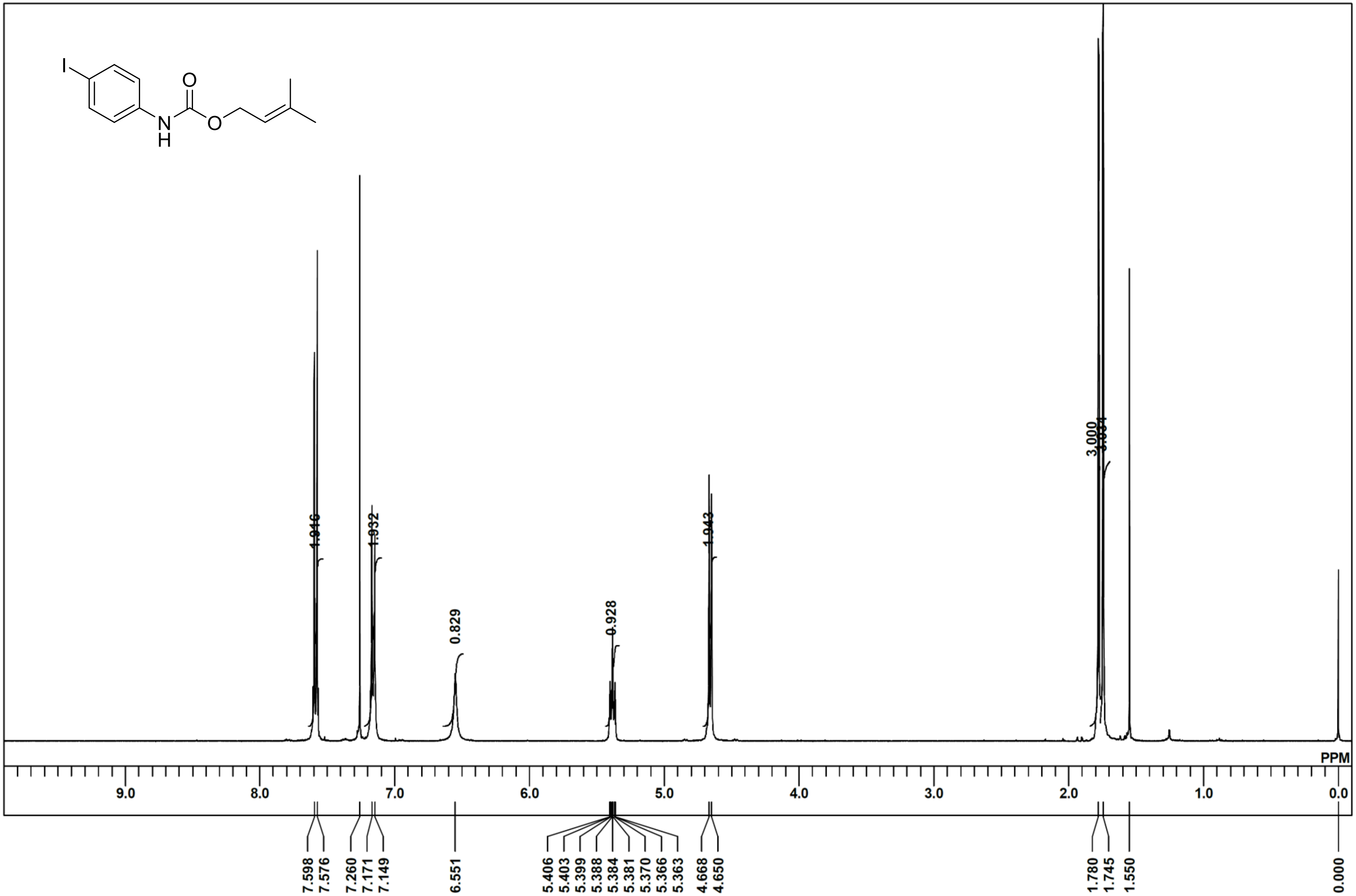

^{13}C NMR spectrum of **1h**

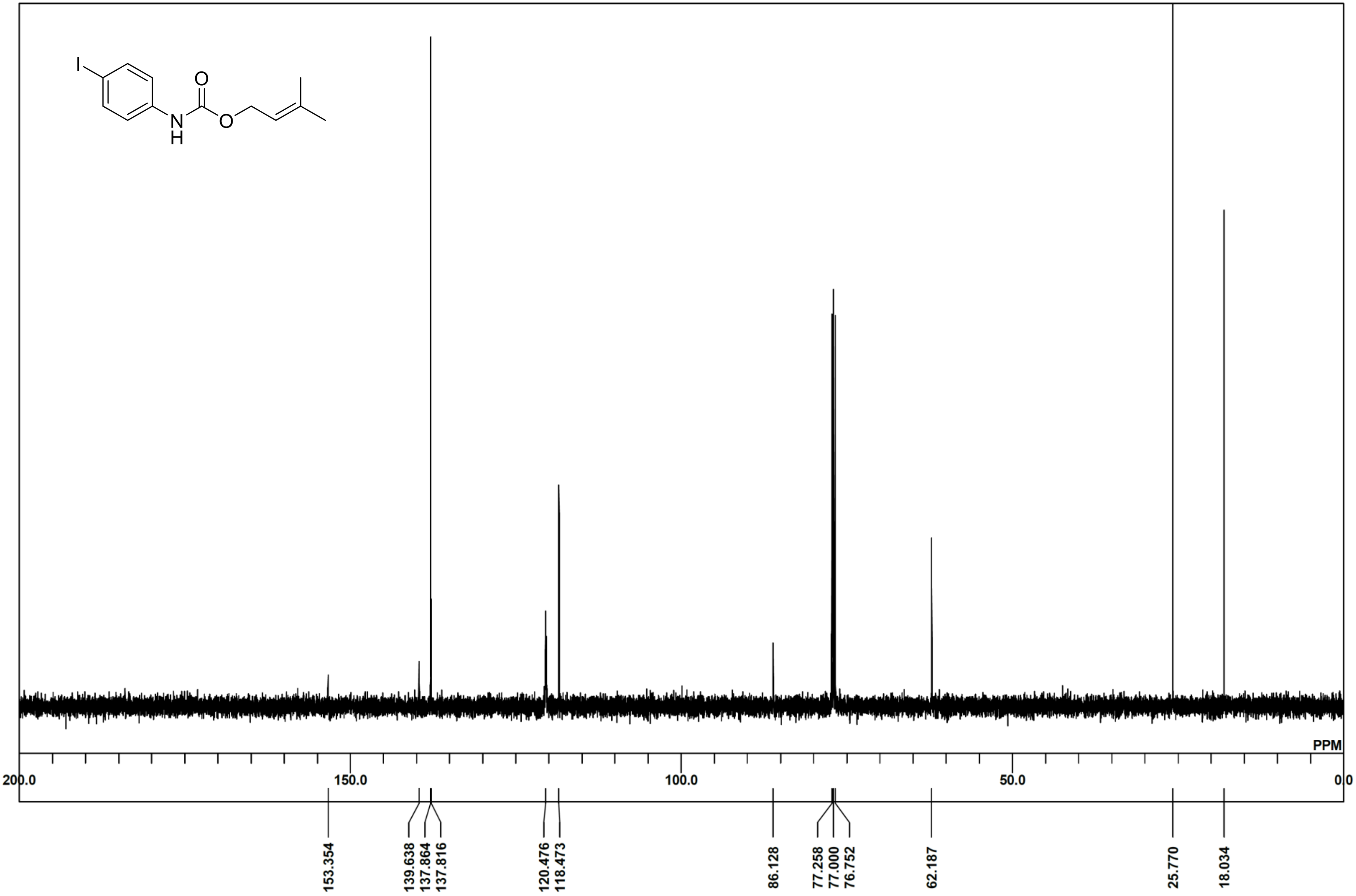

^{1}H NMR spectrum of **1i**

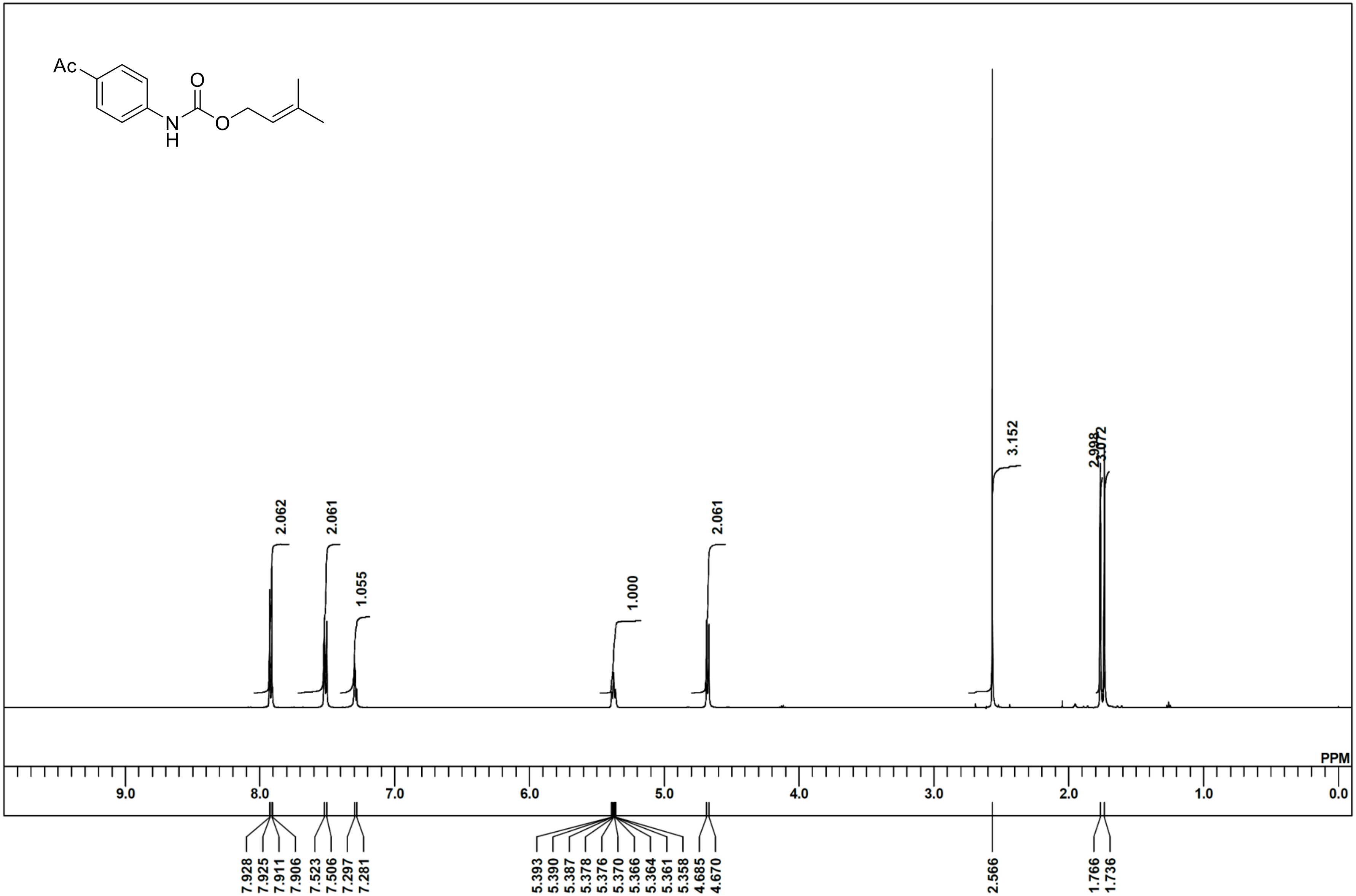

^{13}C NMR spectrum of **1i**

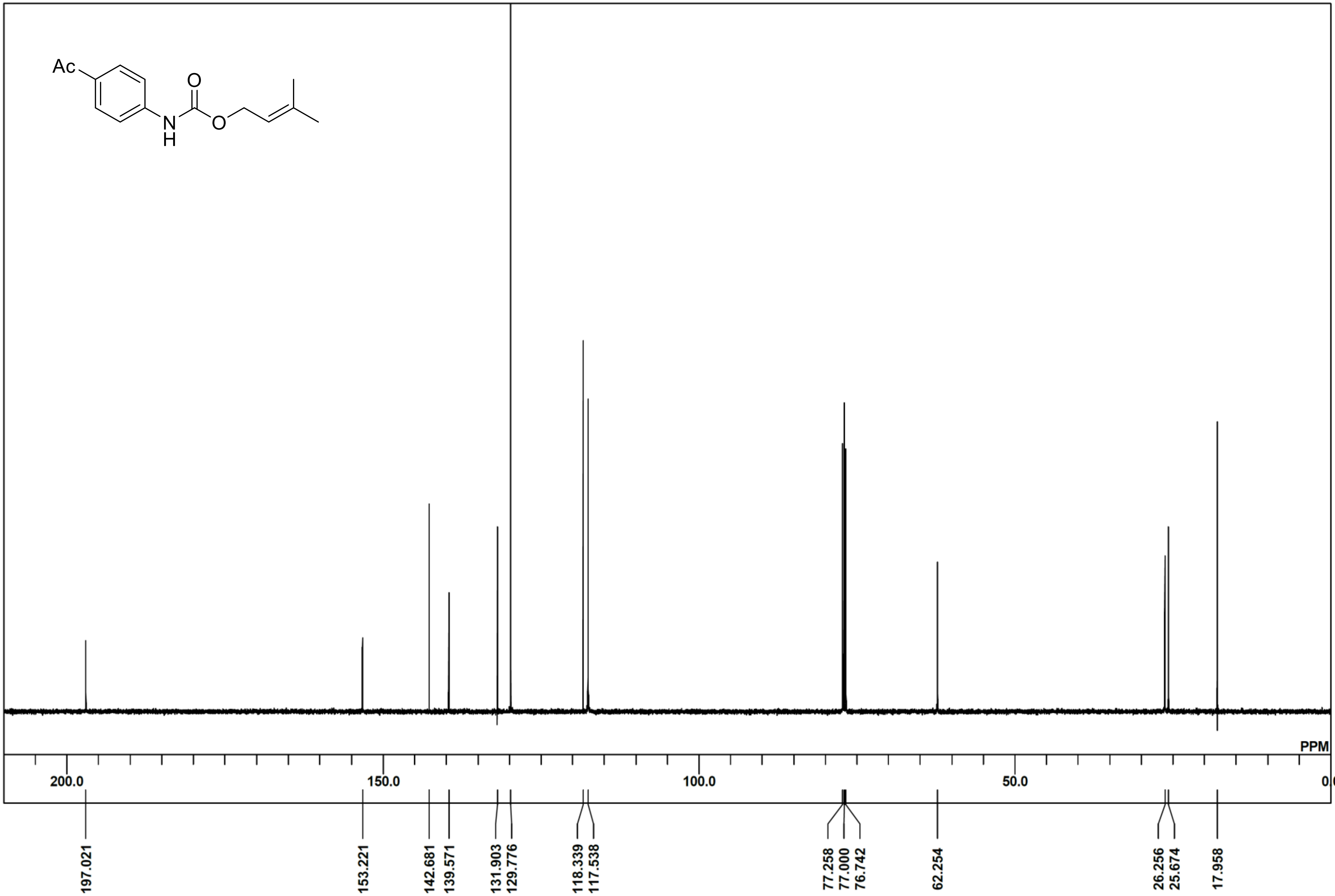

^{1}H NMR spectrum of **1j**

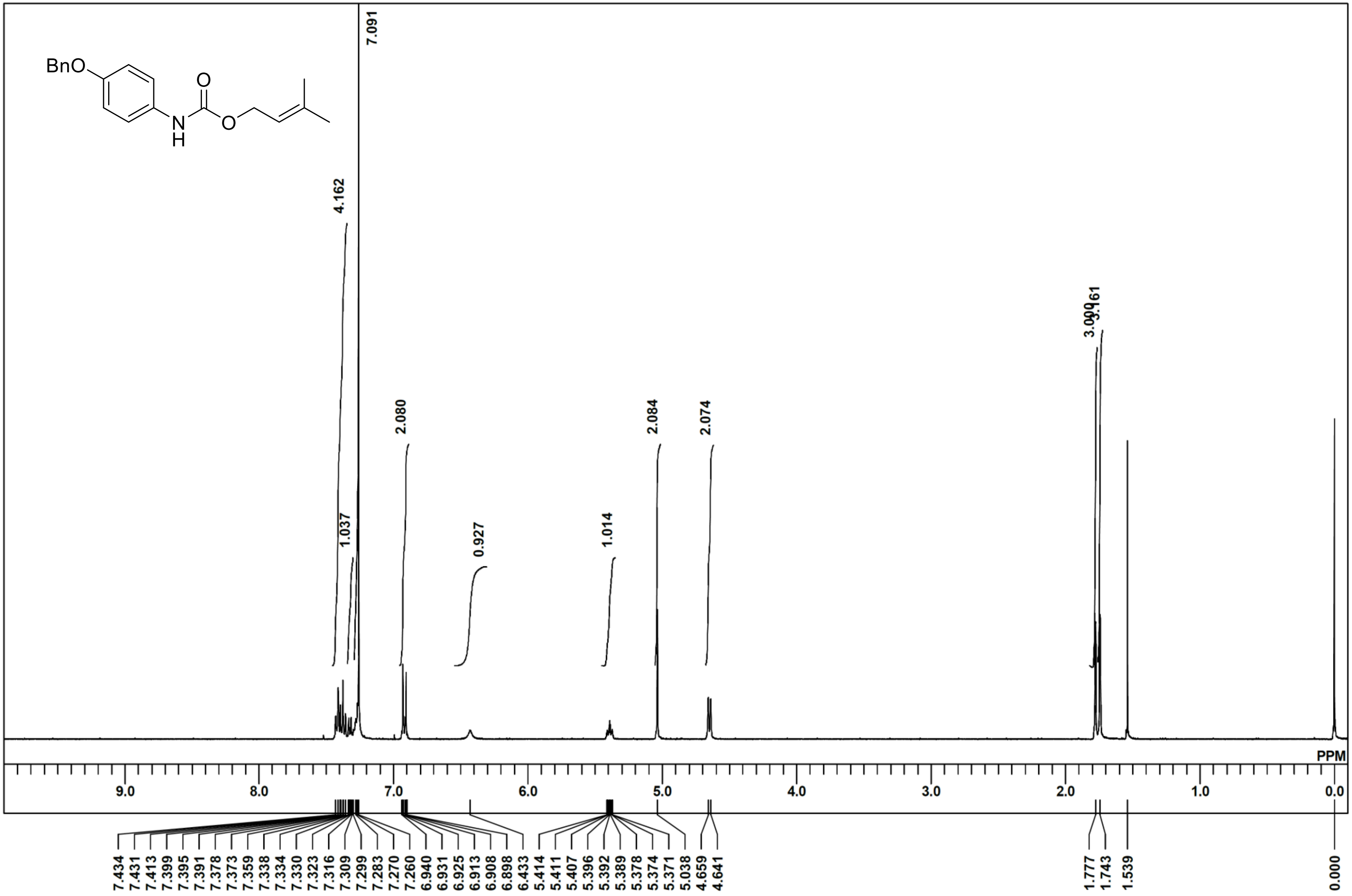

^{13}C NMR spectrum of **1j**

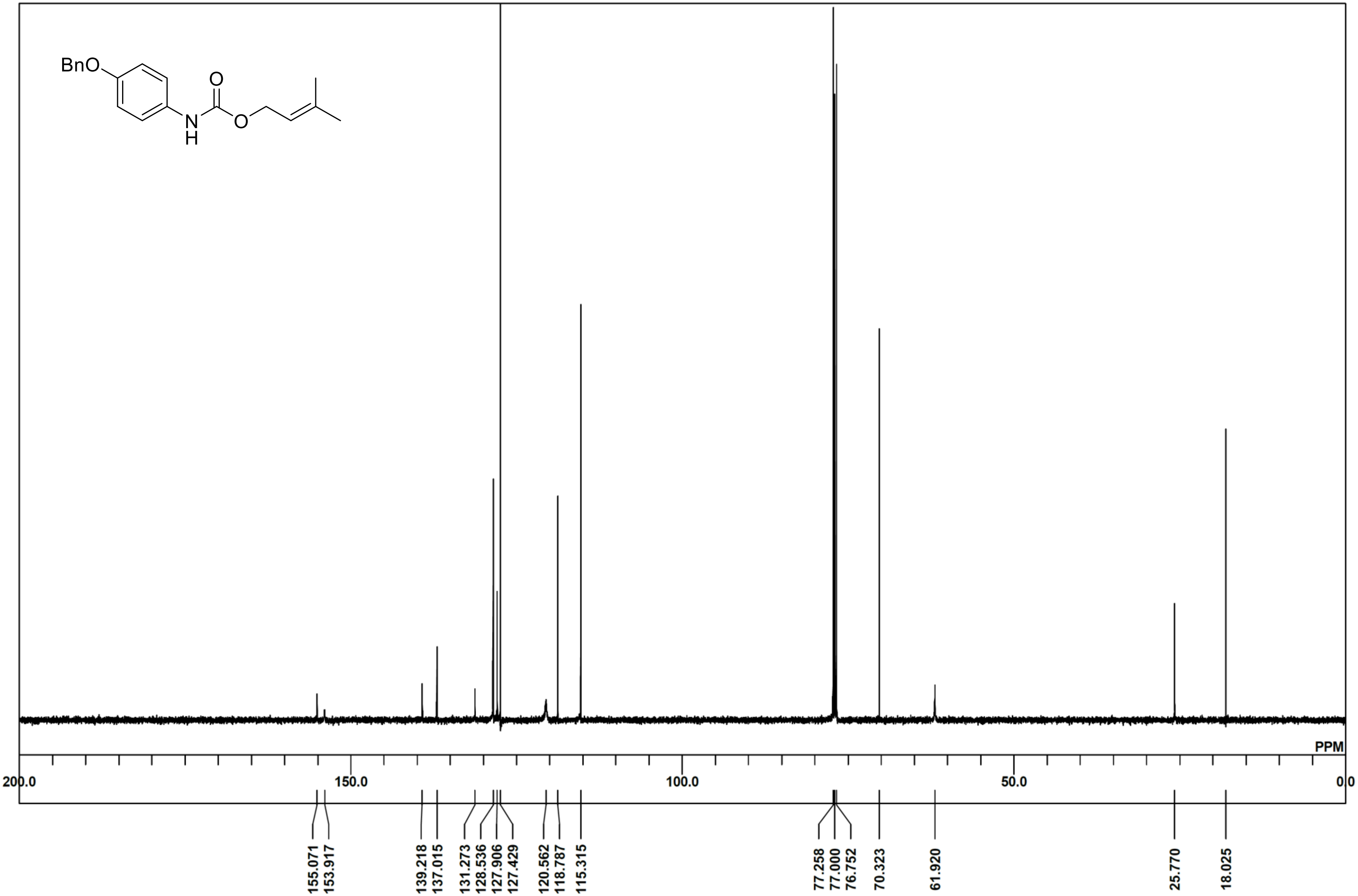

^{1}H NMR spectrum of **1l**

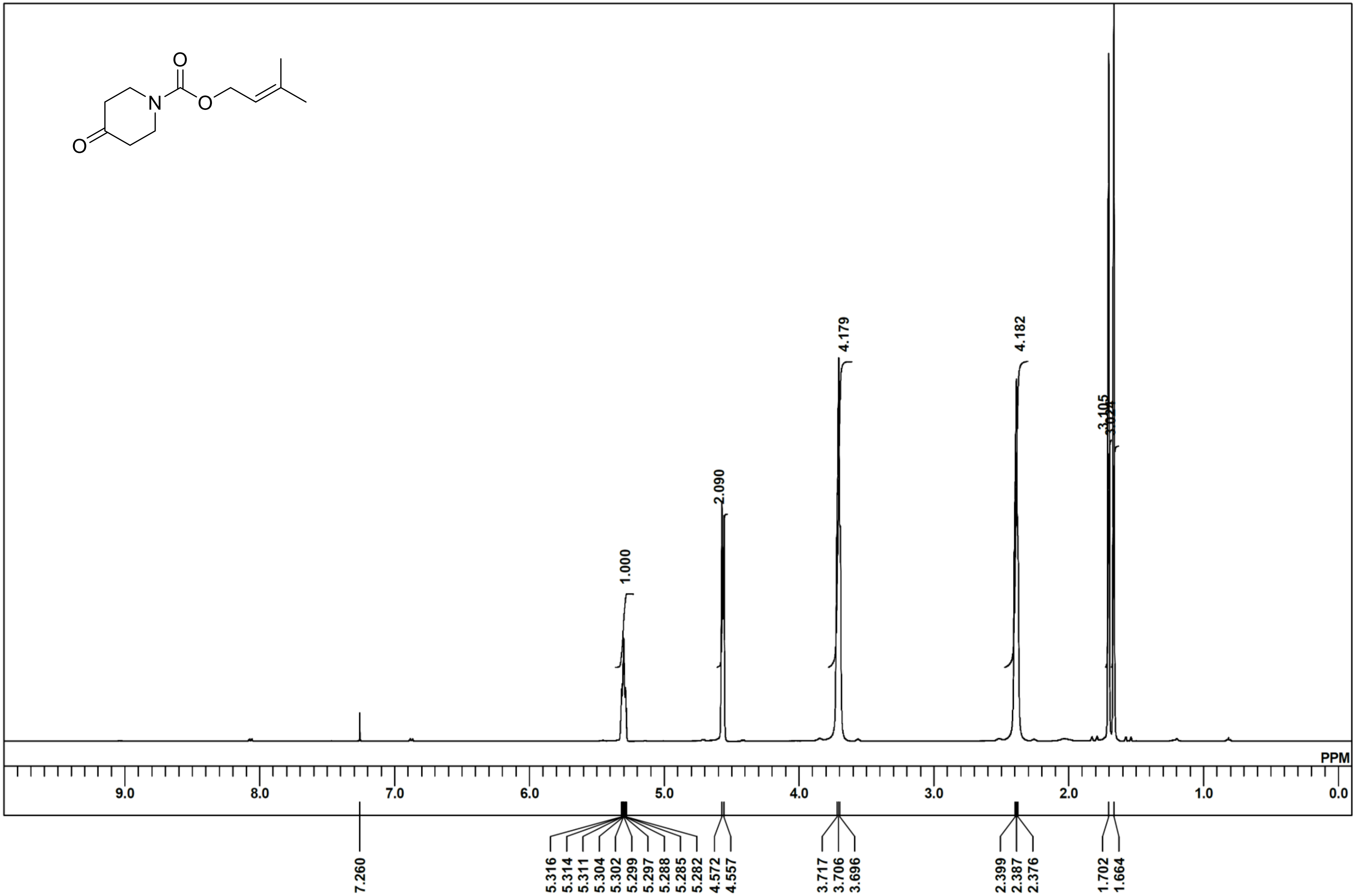

^{13}C NMR spectrum of **1l**

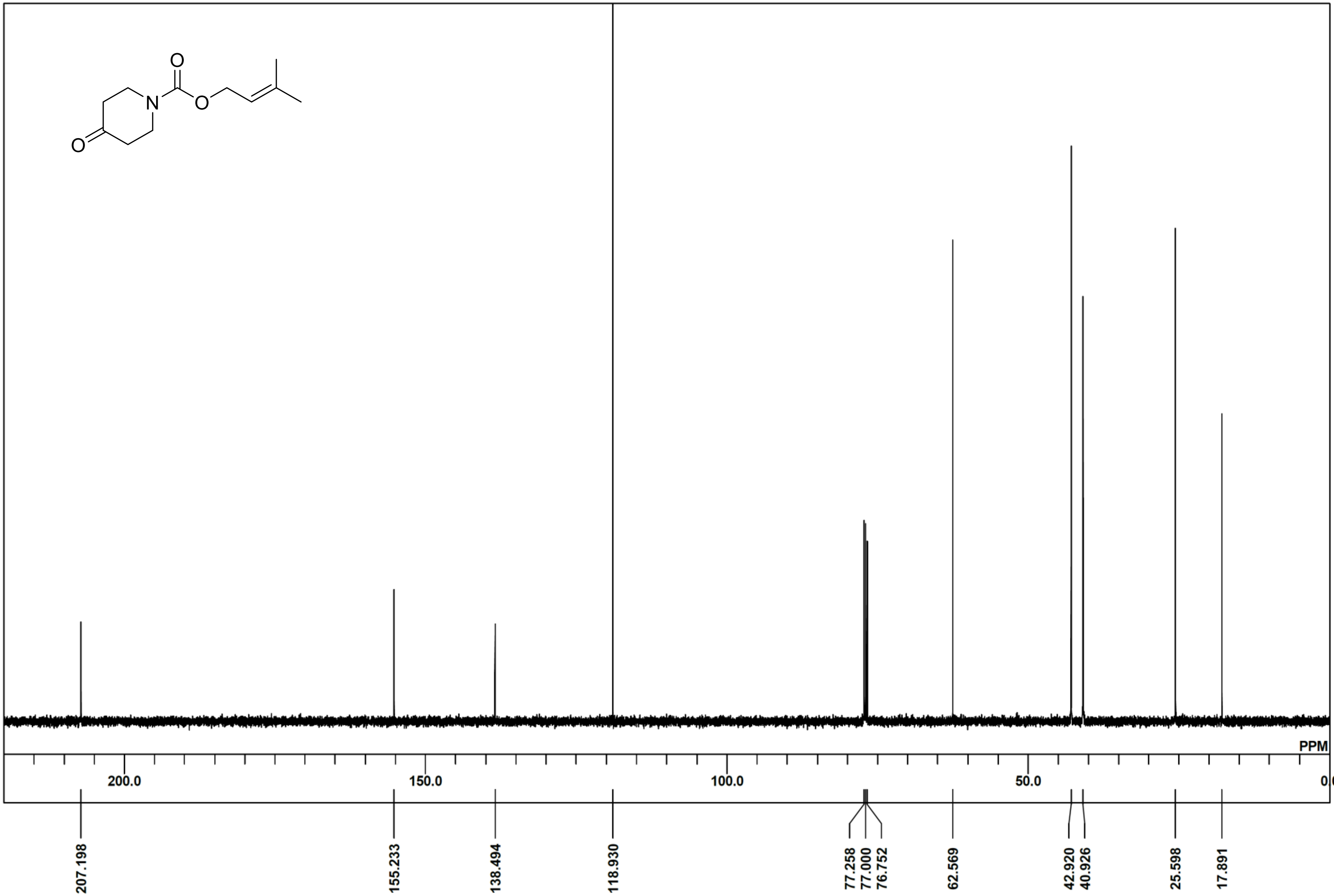

^{1}H NMR spectrum of **1n**

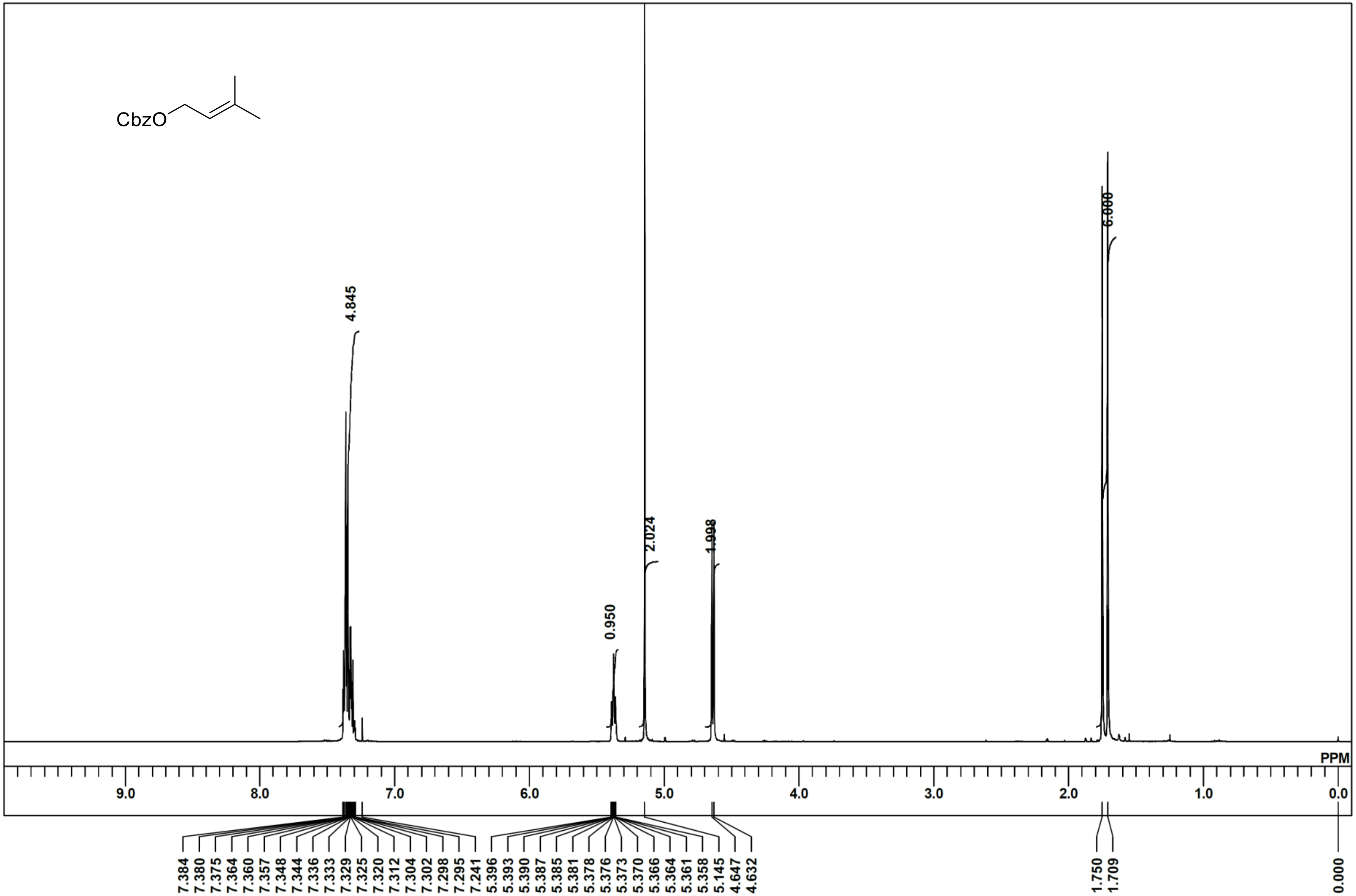

^{13}C NMR spectrum of **1n**

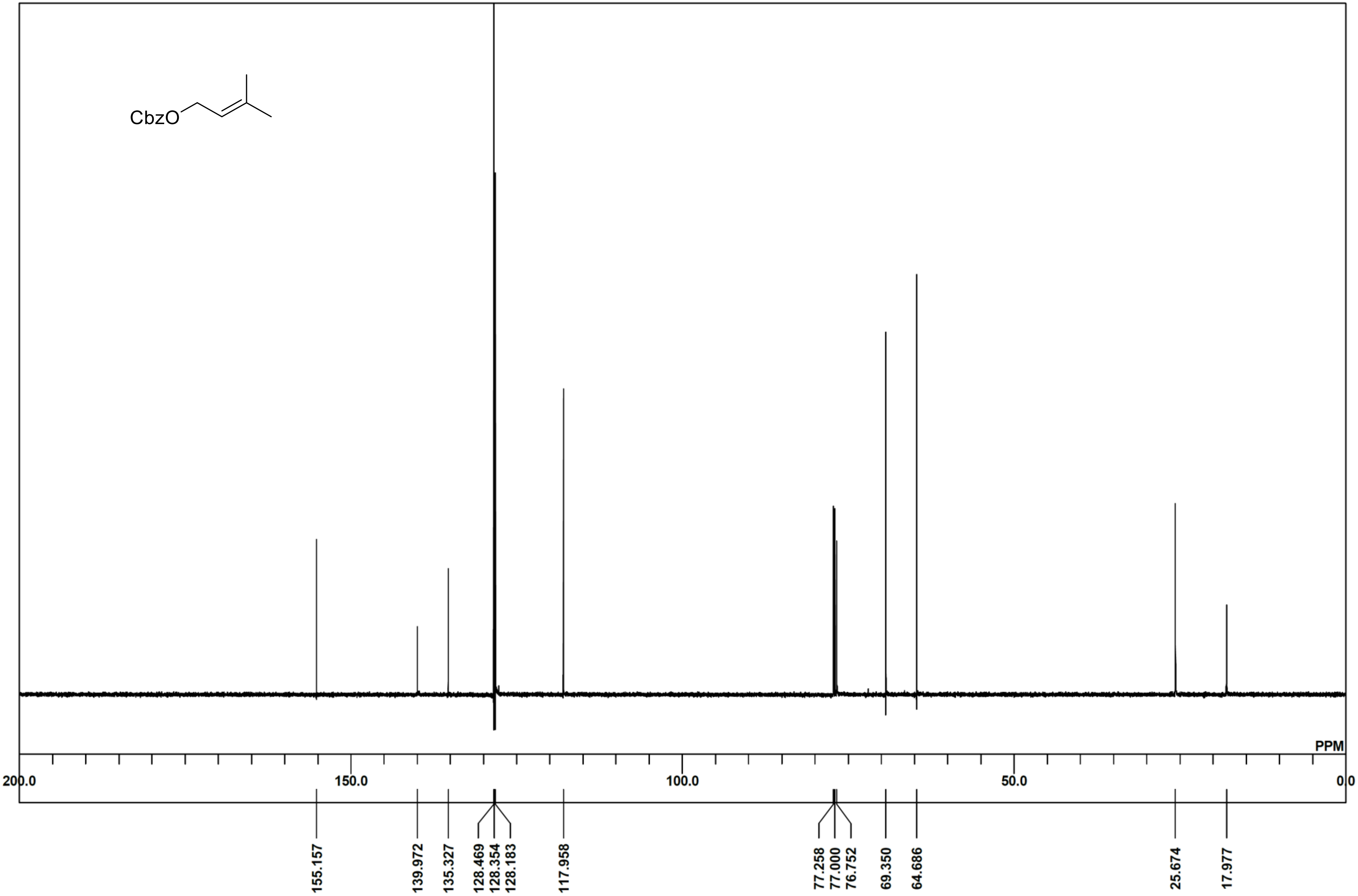

^{1}H NMR spectrum of **1o**

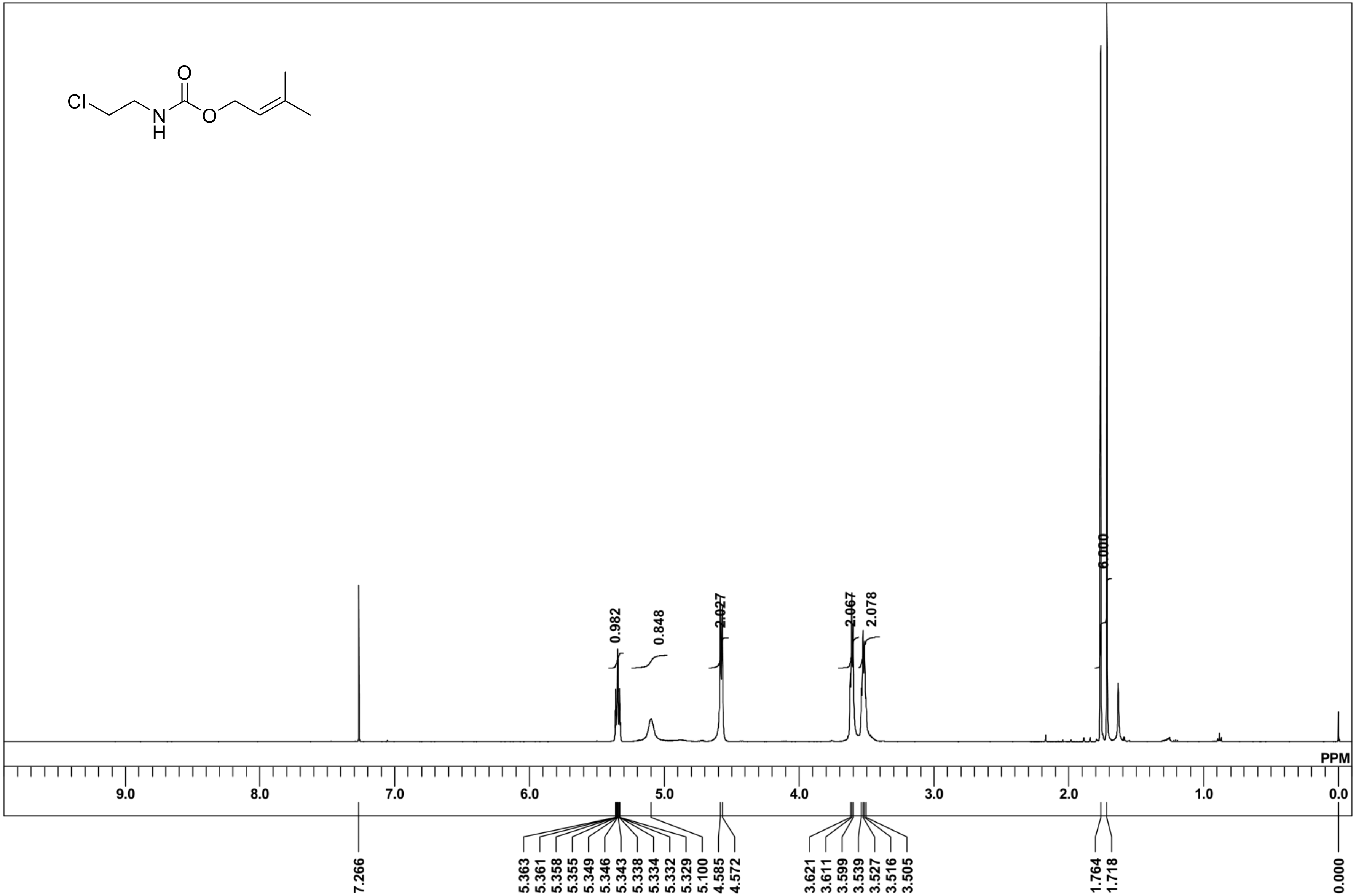

^{13}C NMR spectrum of **1o**

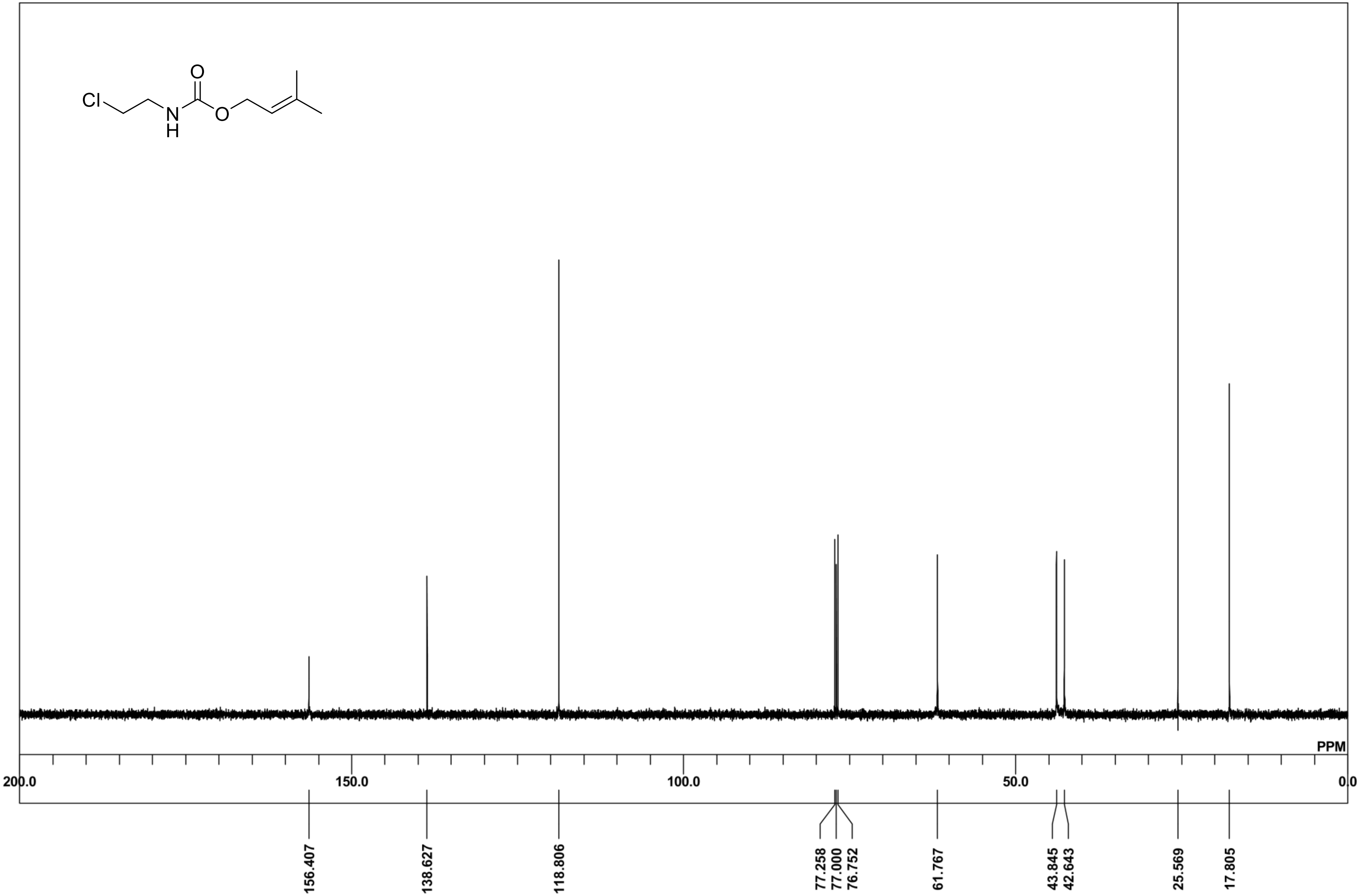

^{1}H NMR spectrum of **1p**

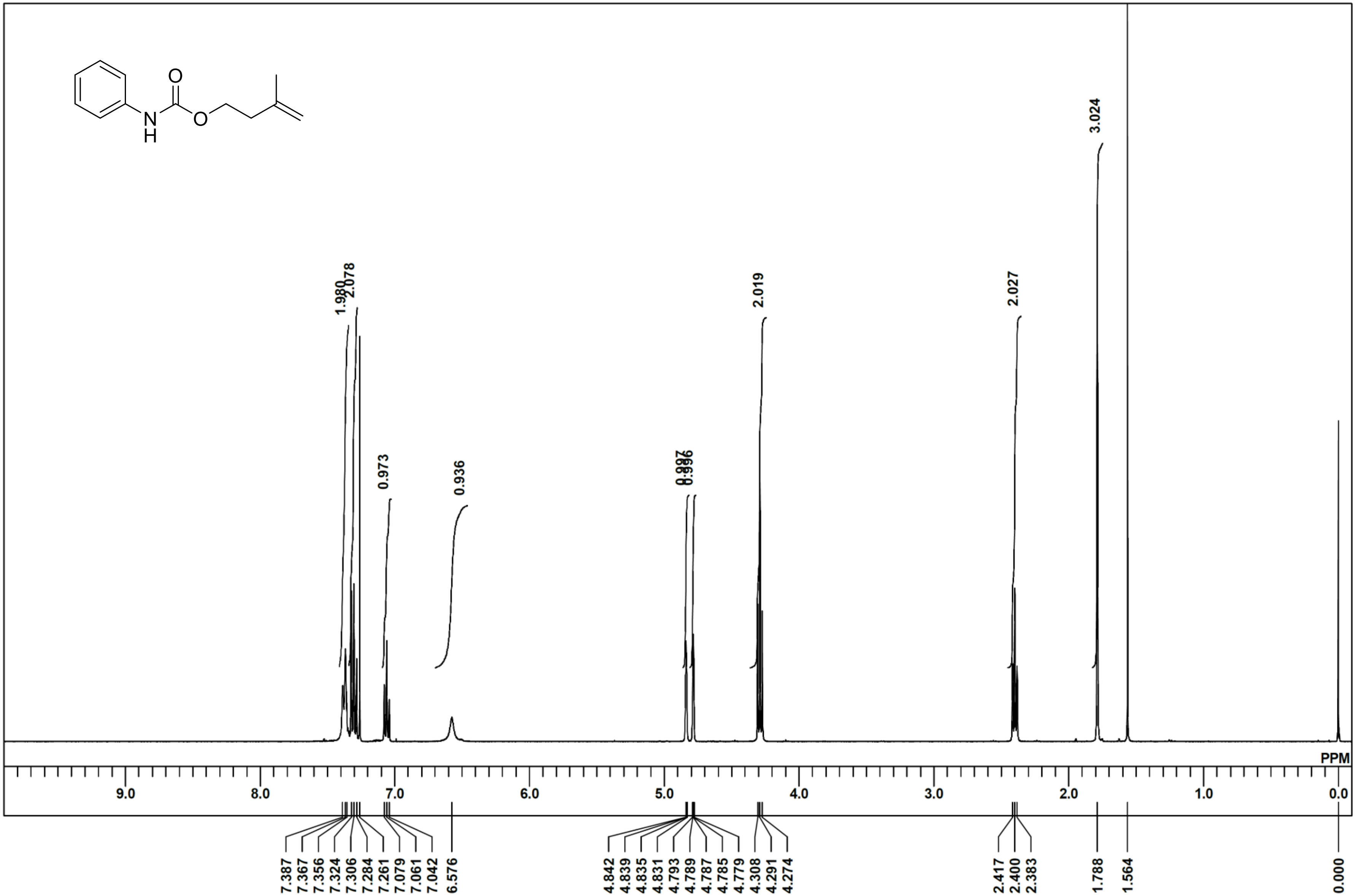

^{13}C NMR spectrum of **1p**

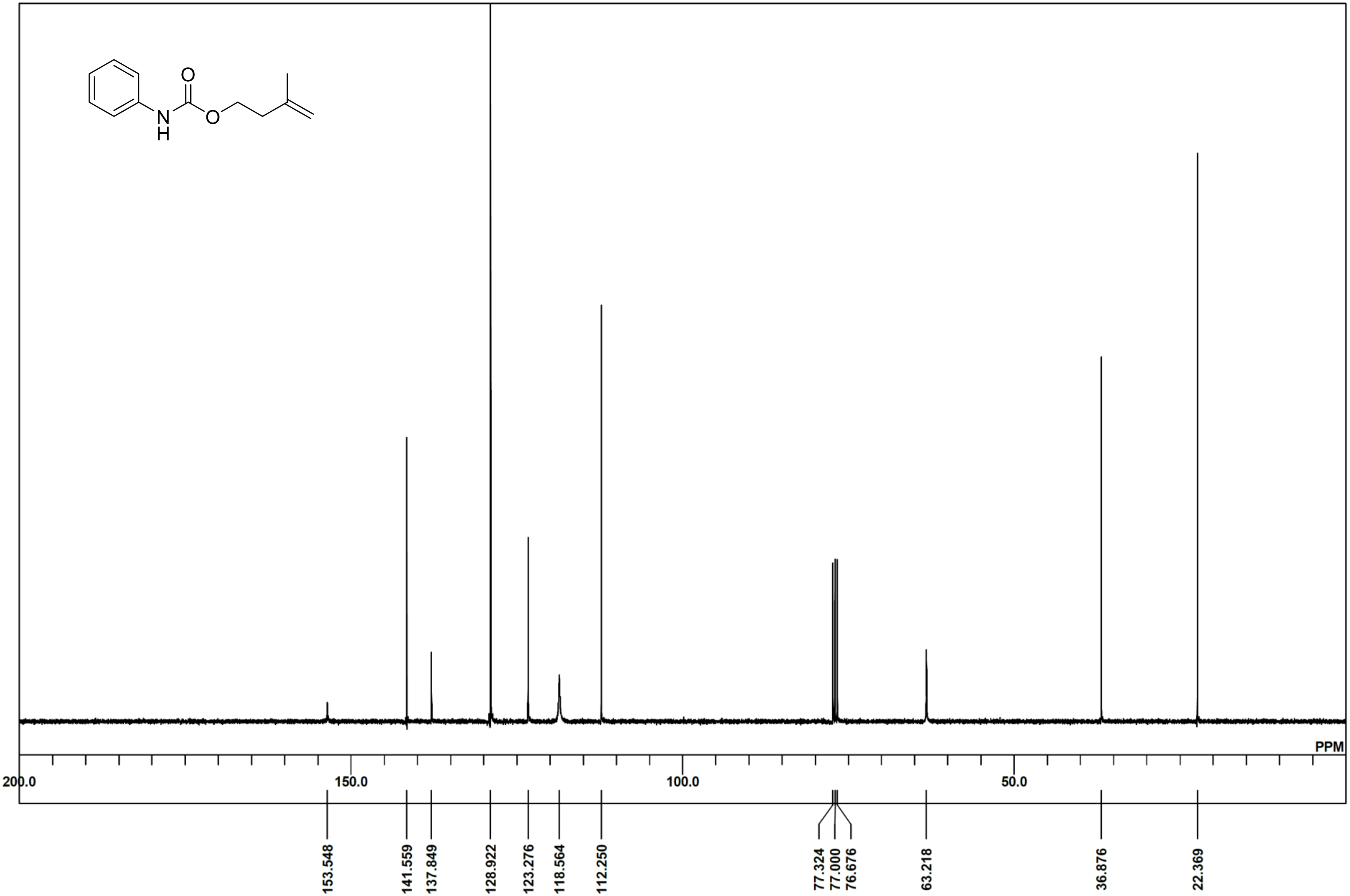

^{1}H NMR spectrum of **1w**

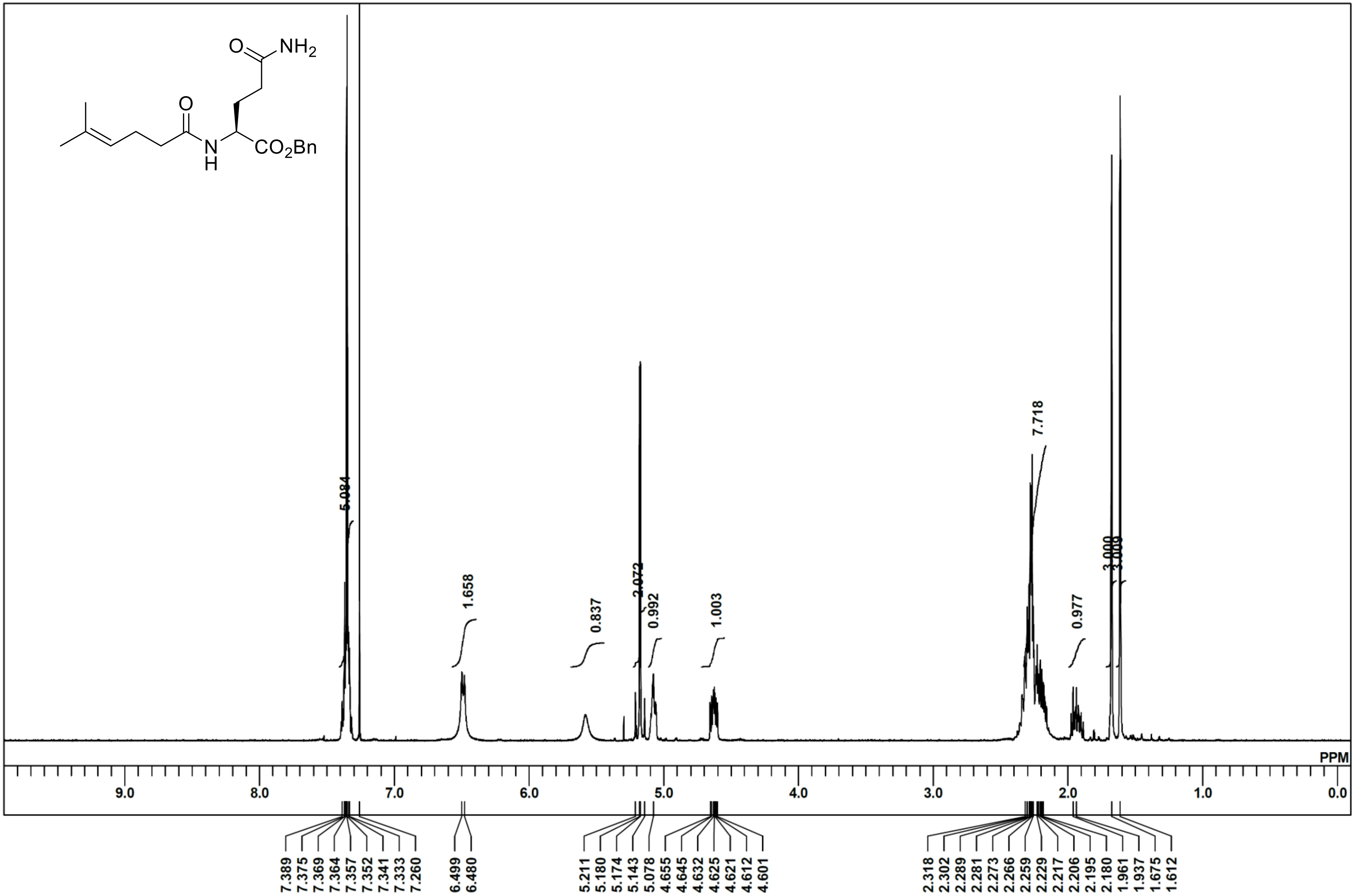

^{13}C NMR spectrum of **1w**

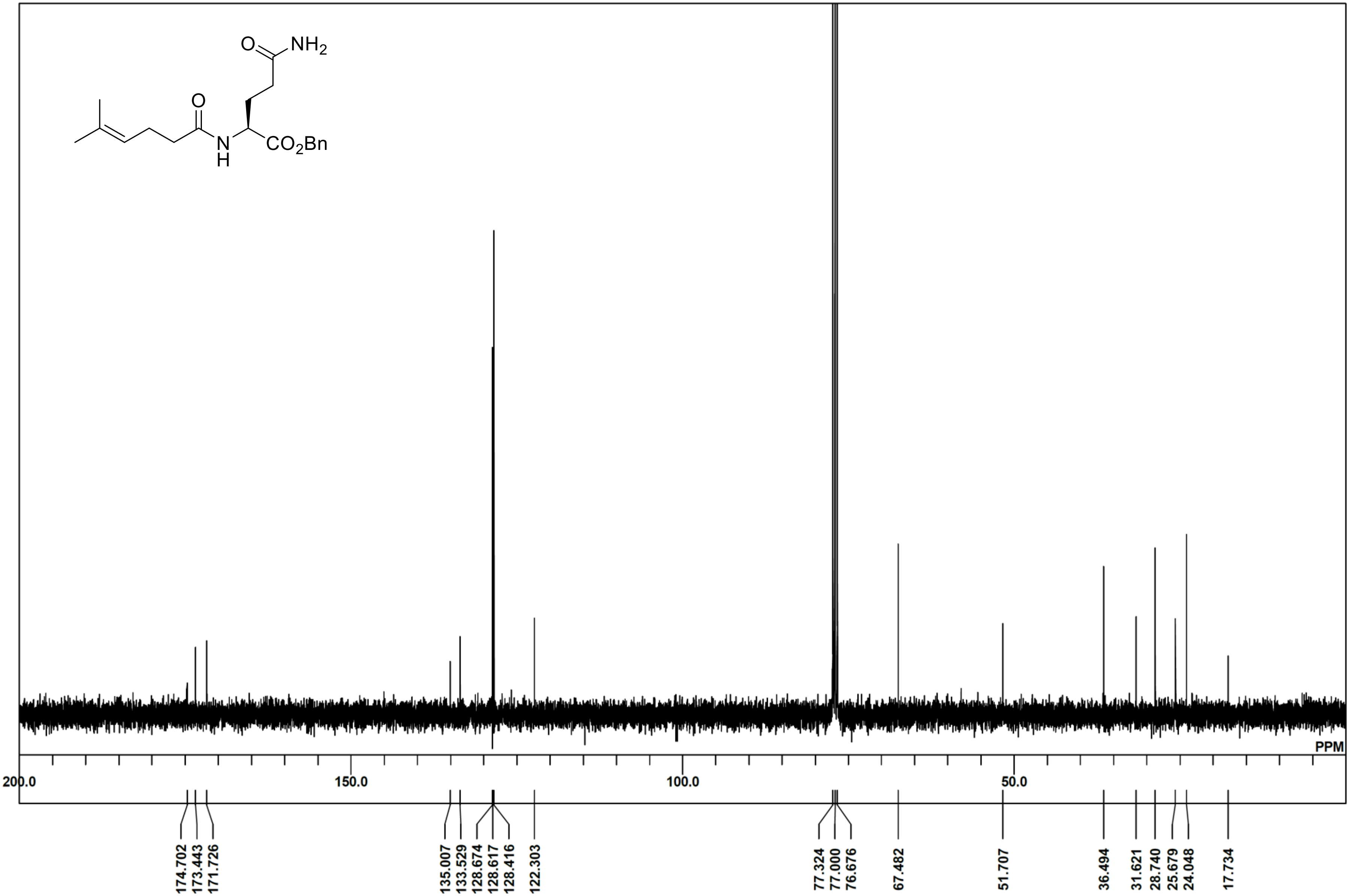

^{1}H NMR spectrum of **1x**

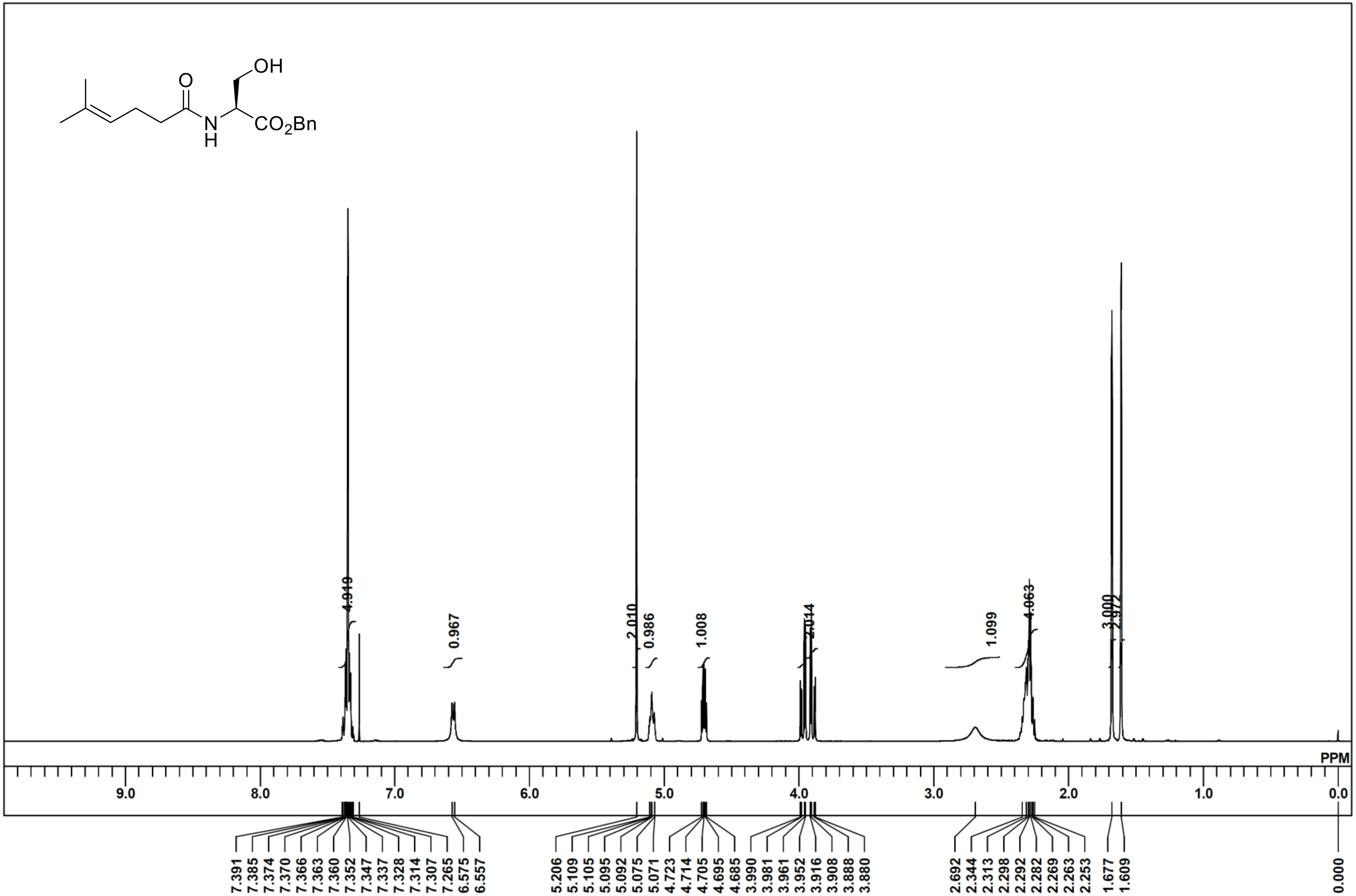

^{13}C NMR spectrum of **1x**

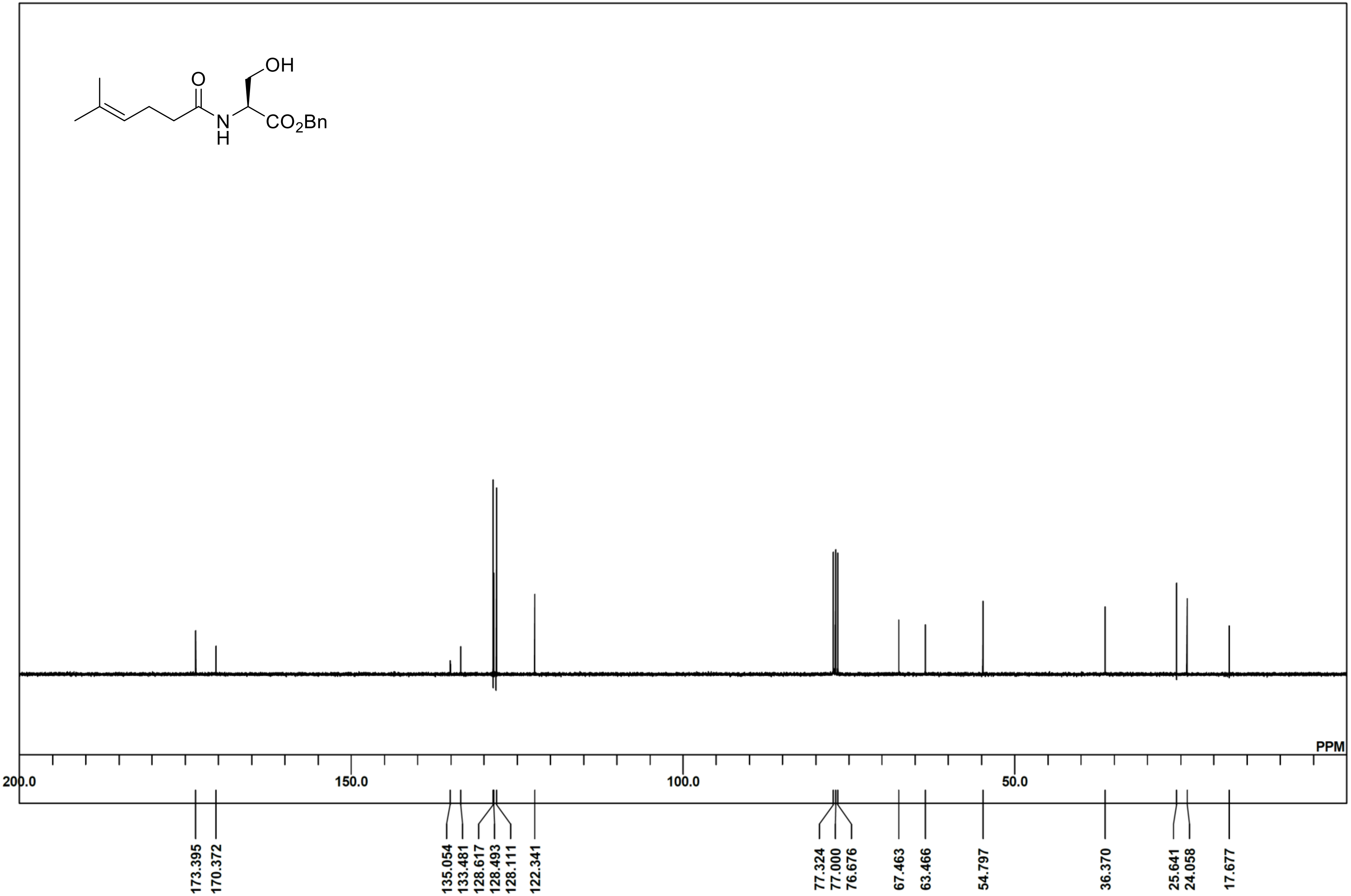

^{1}H NMR spectrum of **1y**

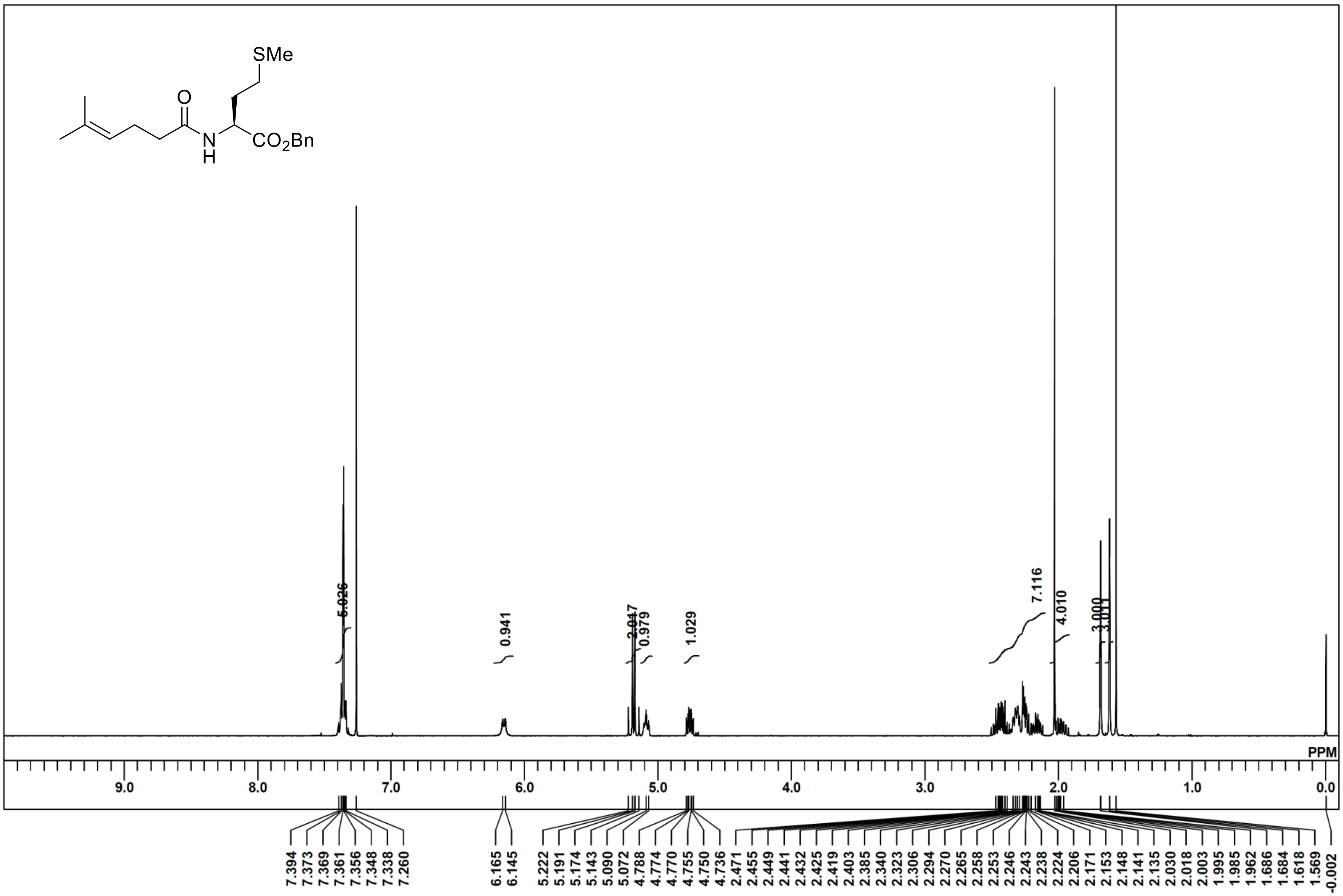

^{13}C NMR spectrum of **1y**

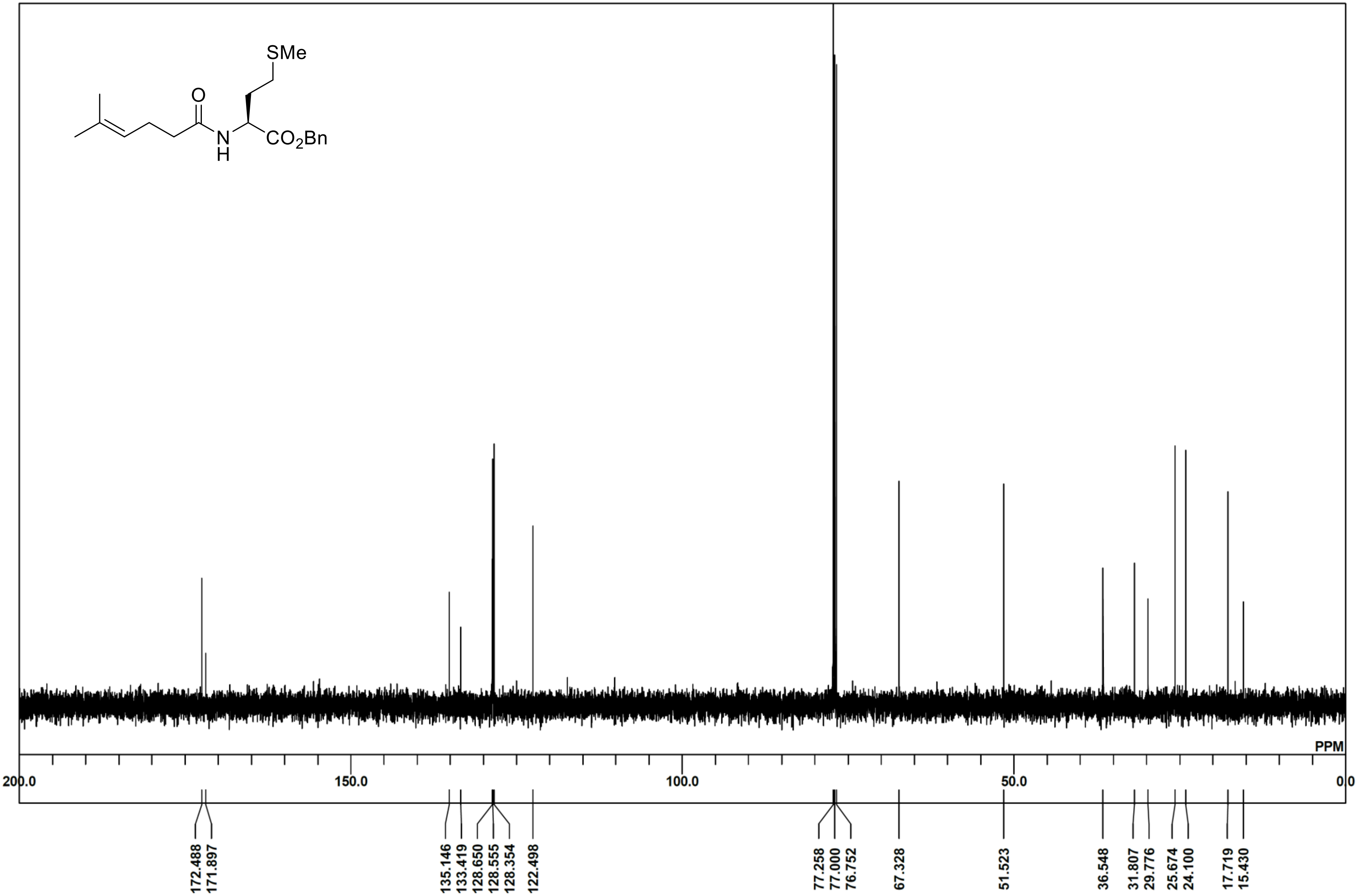

^{1}H NMR spectrum of **1z**

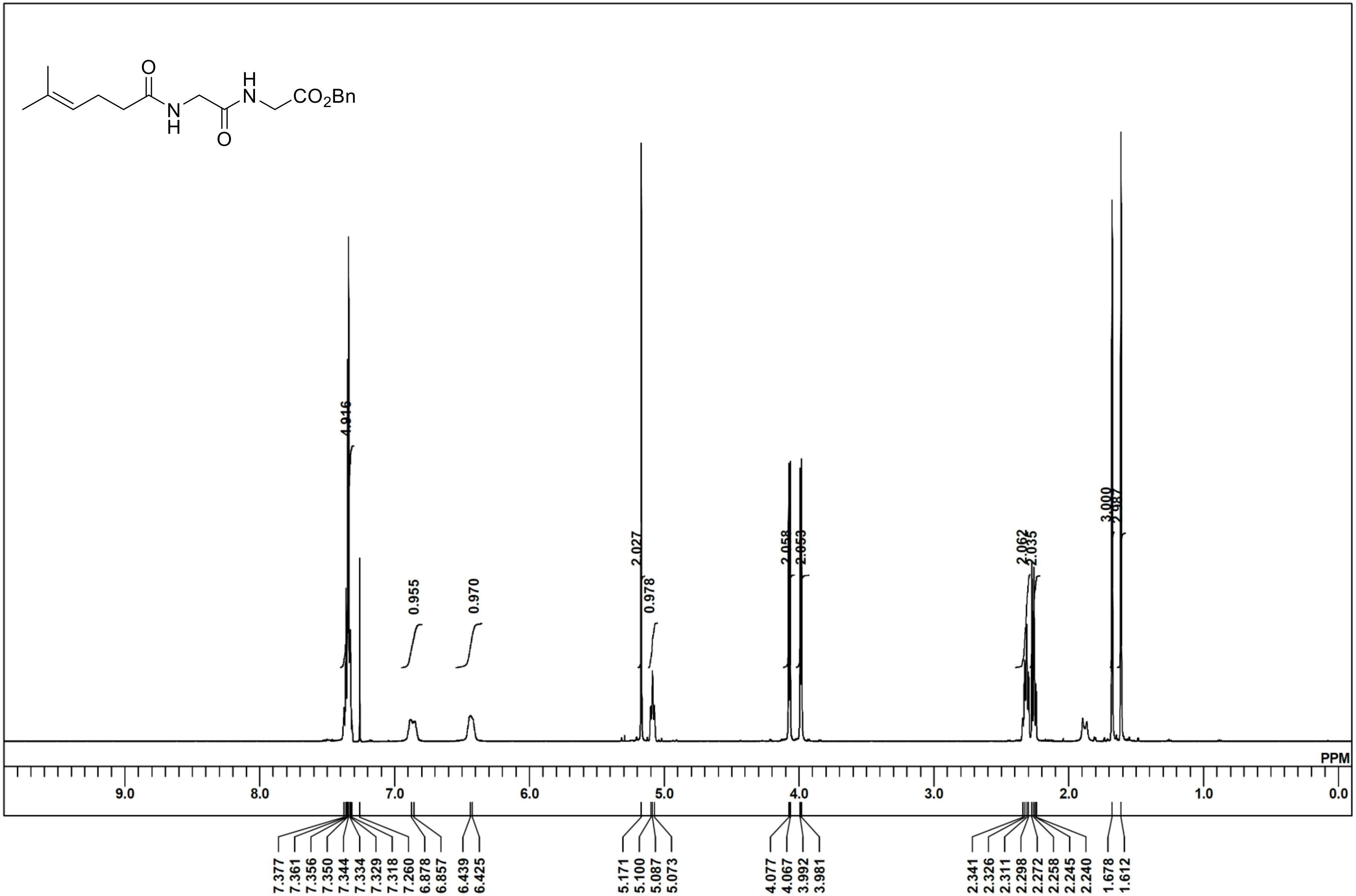

^{13}C NMR spectrum of **1z**

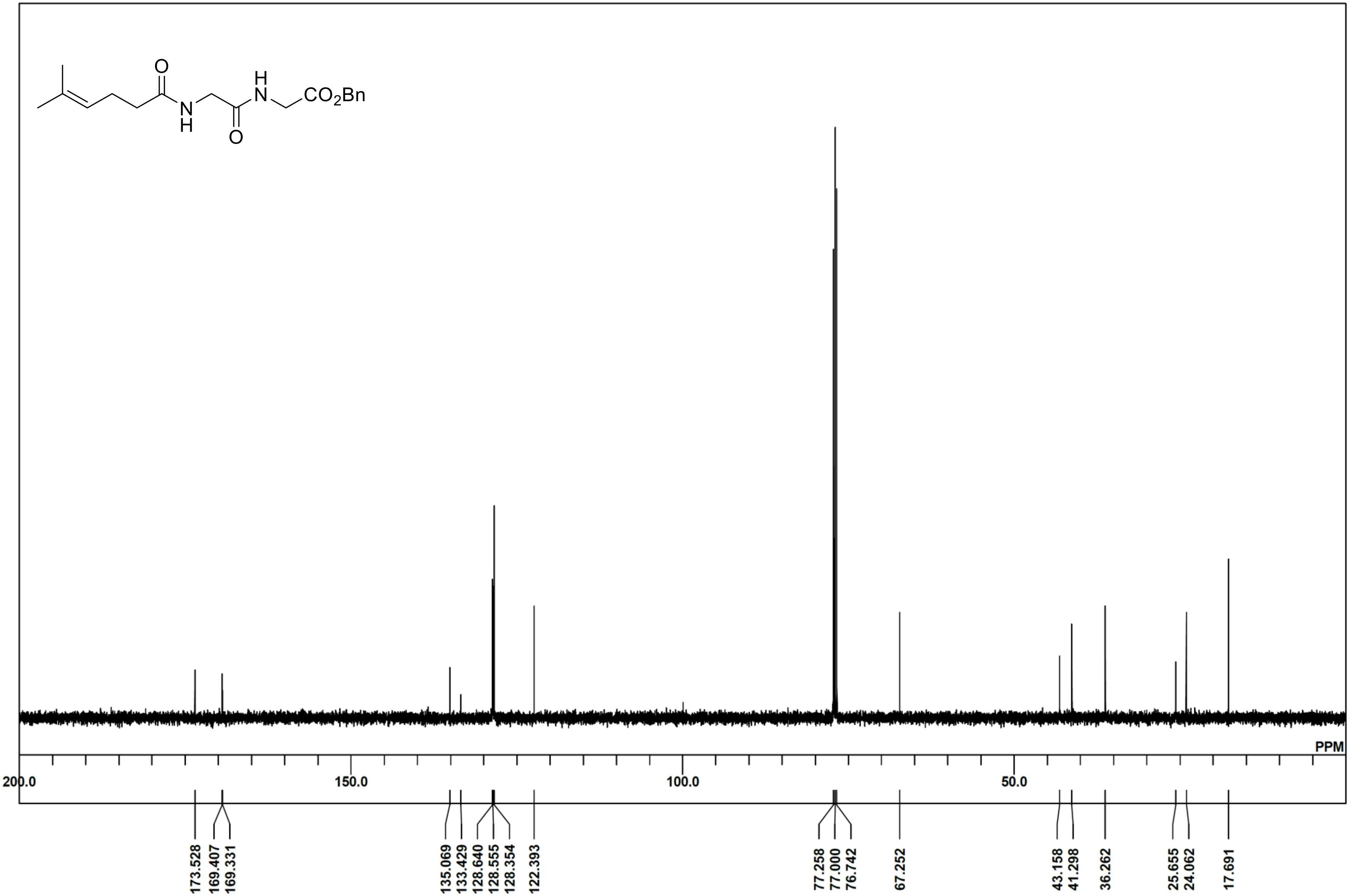

^{1}H NMR spectrum of **2a**

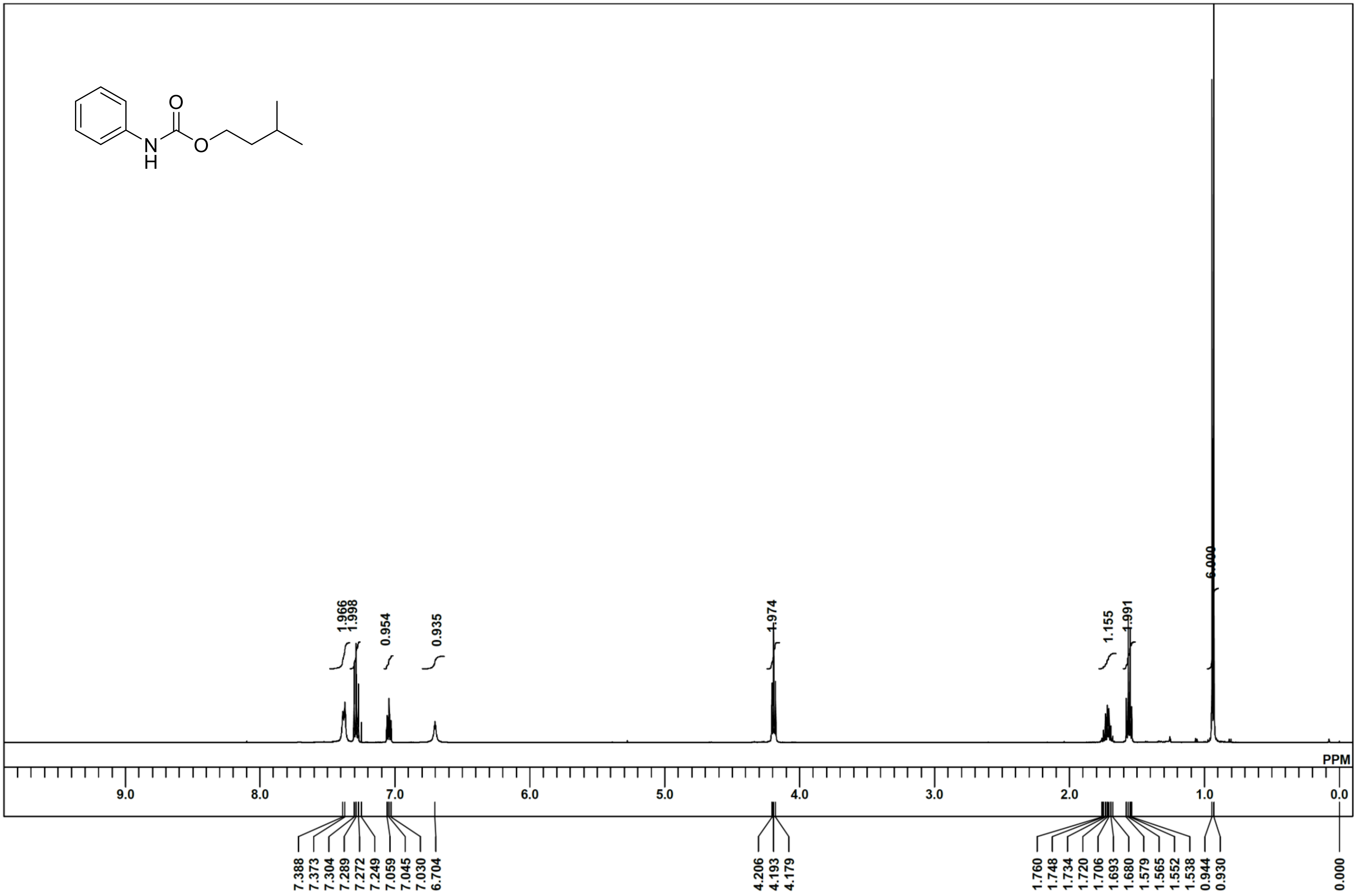

^{13}C NMR spectrum of **2a**

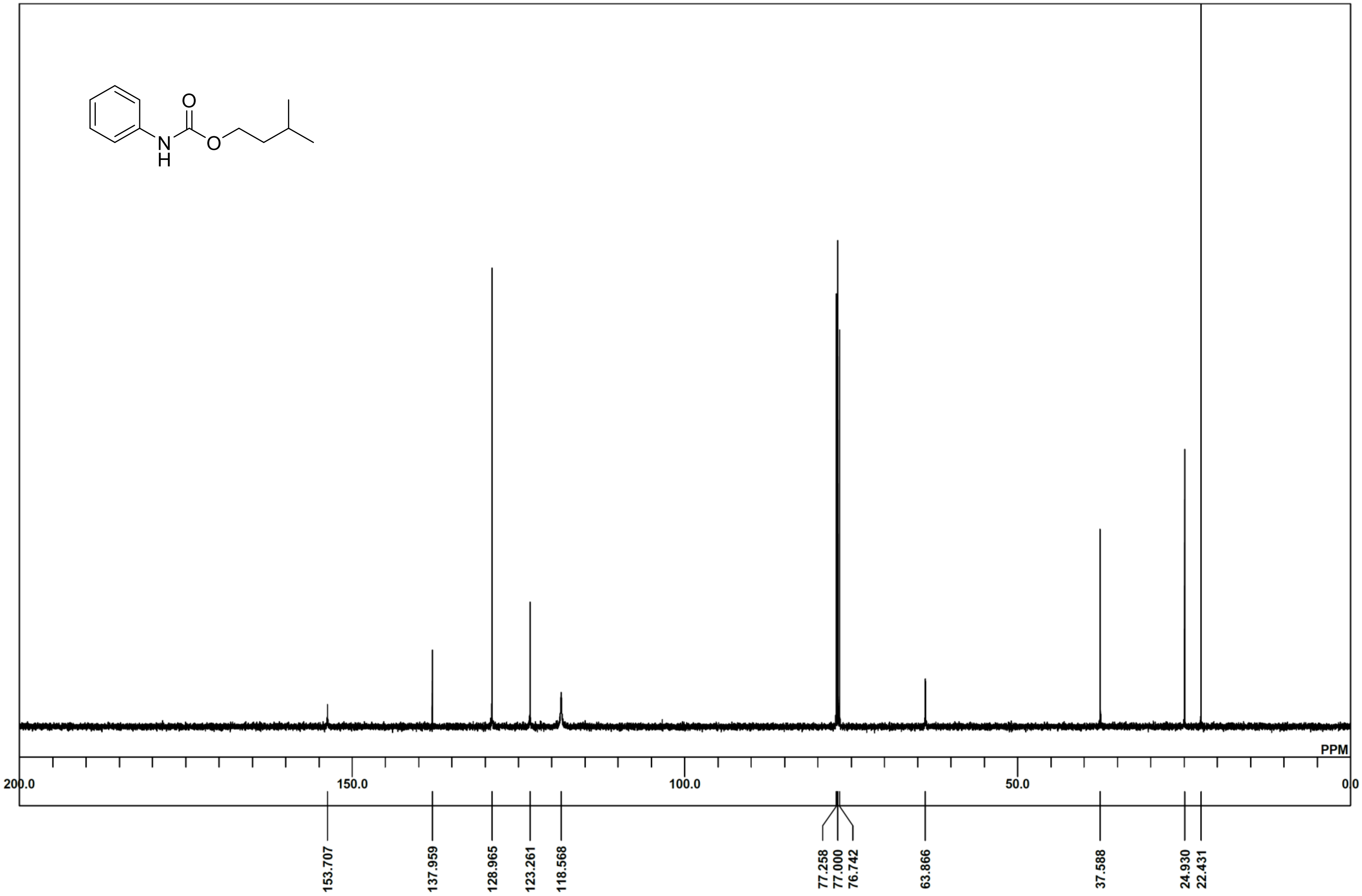

^{1}H NMR spectrum of **2b**

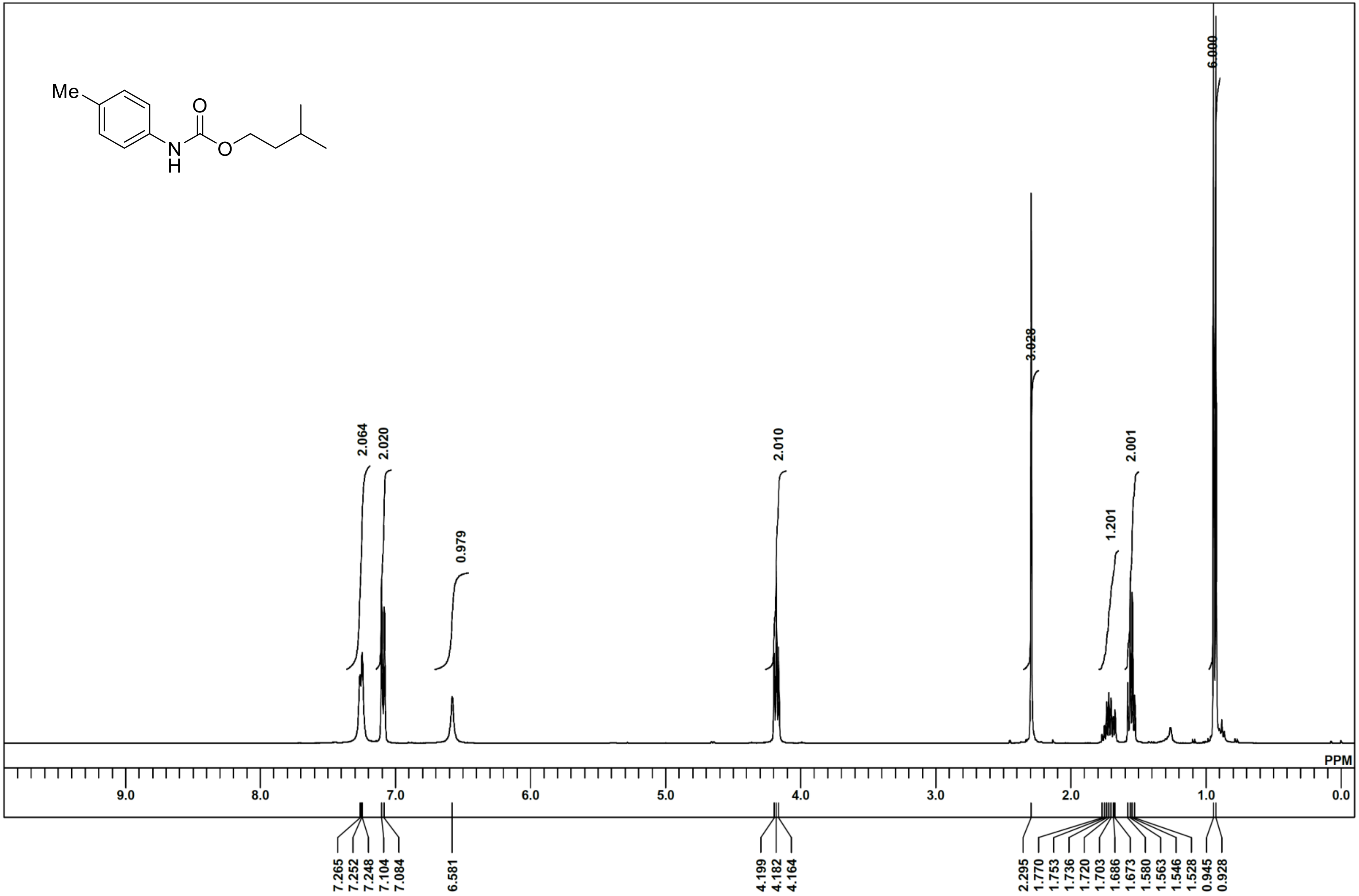

^{13}C NMR spectrum of **2b**

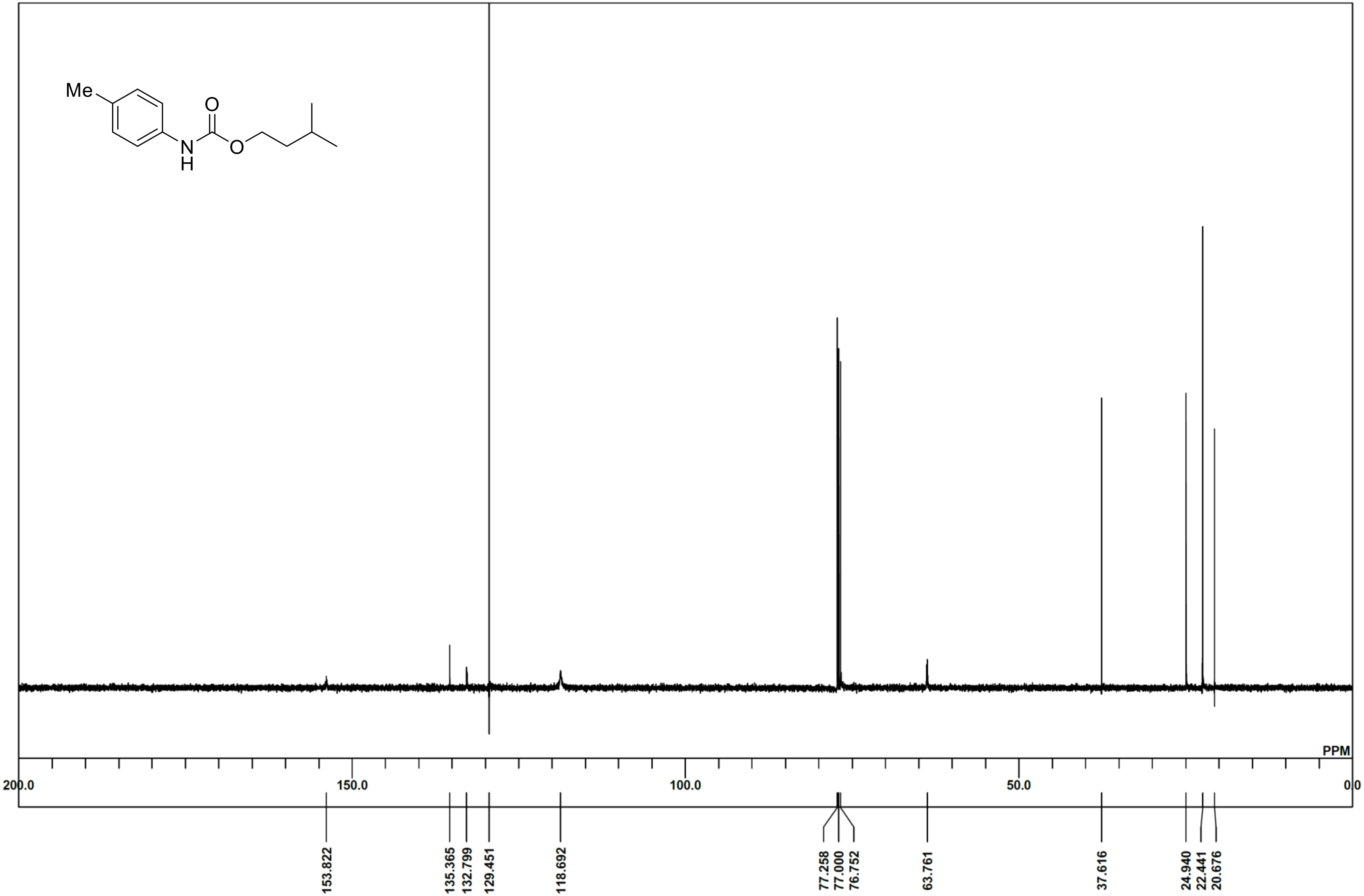

^{1}H NMR spectrum of **2c**

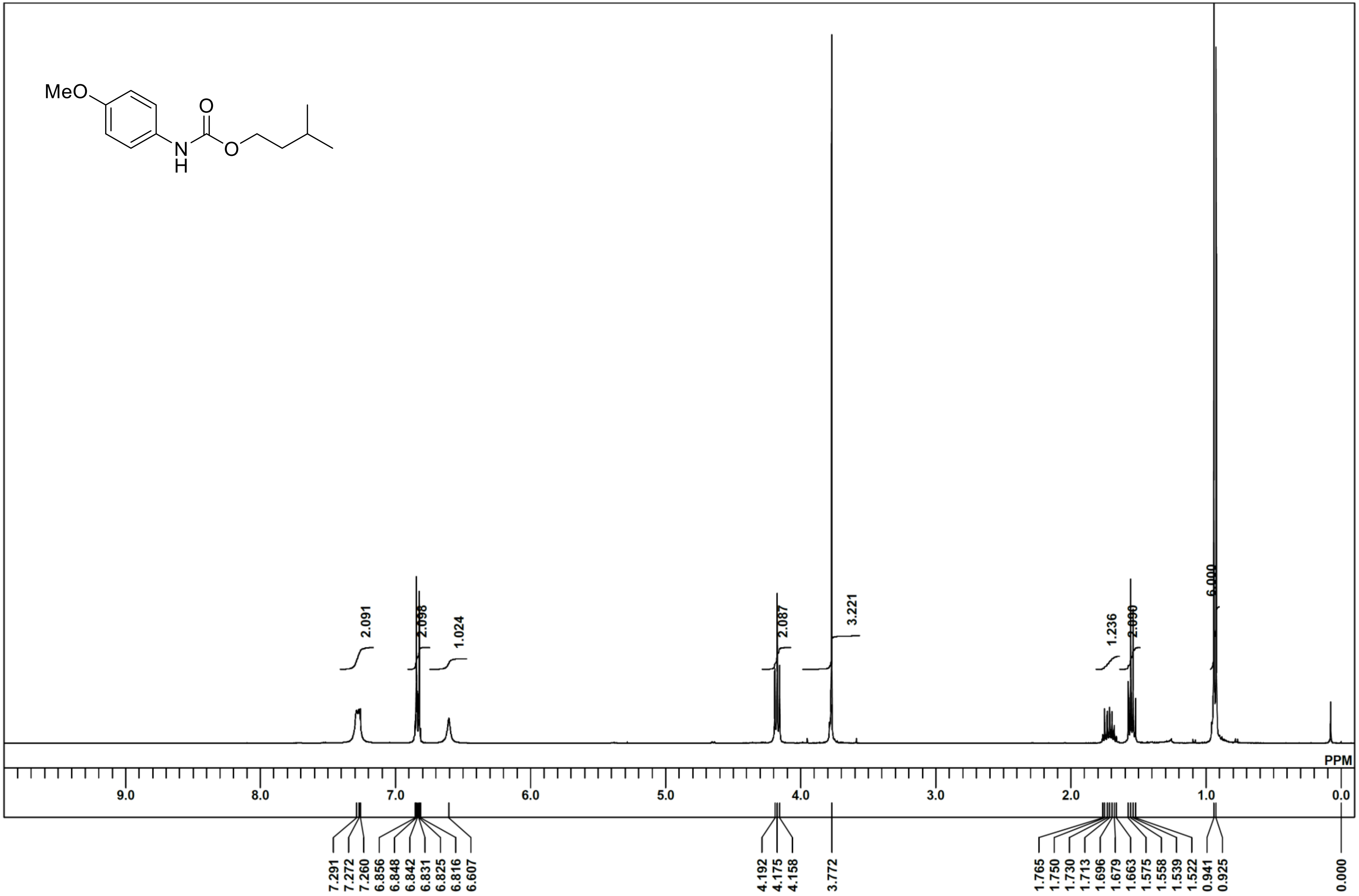

^{13}C NMR spectrum of **2c**

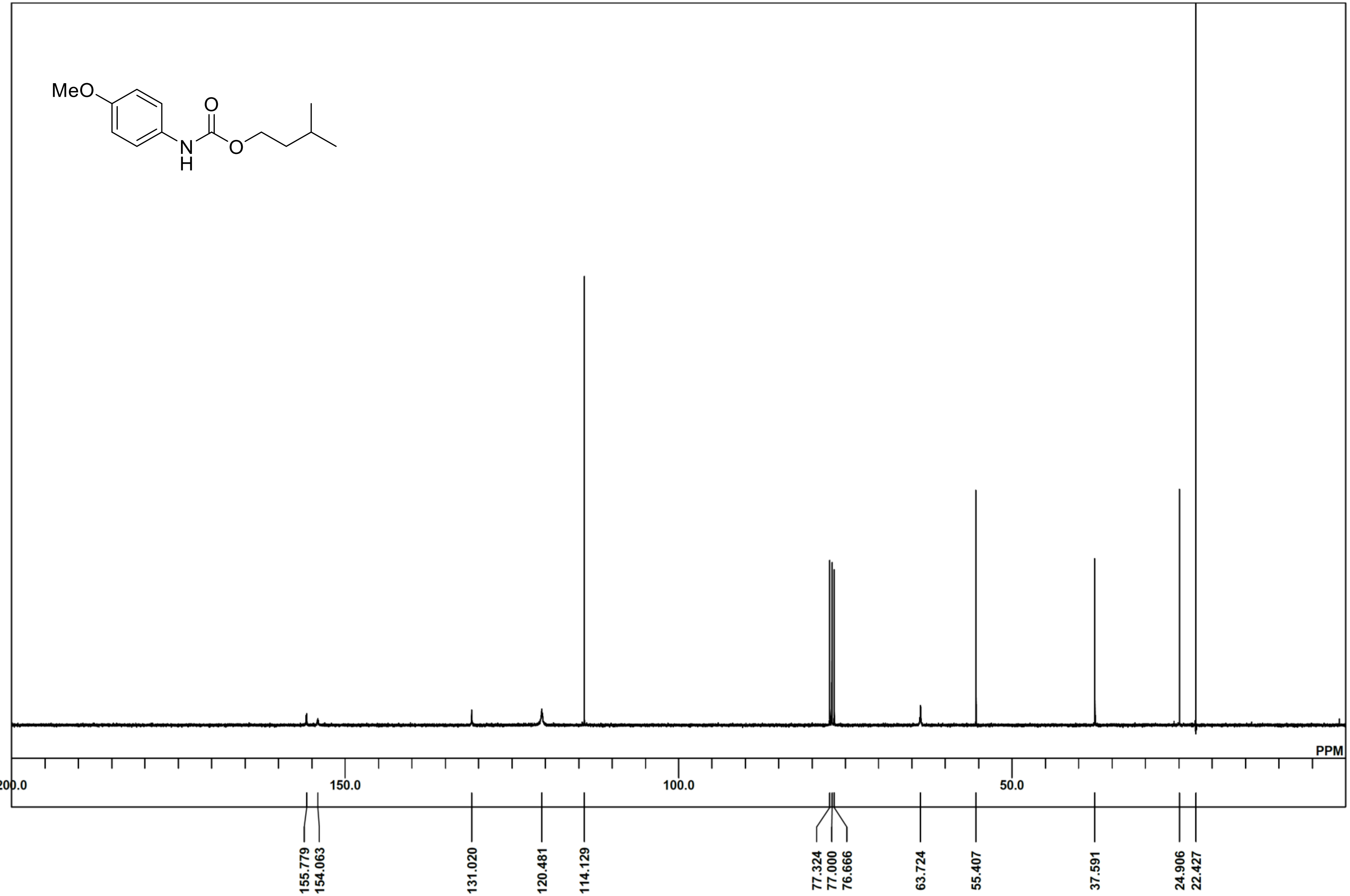

^{1}H NMR spectrum of **2d**

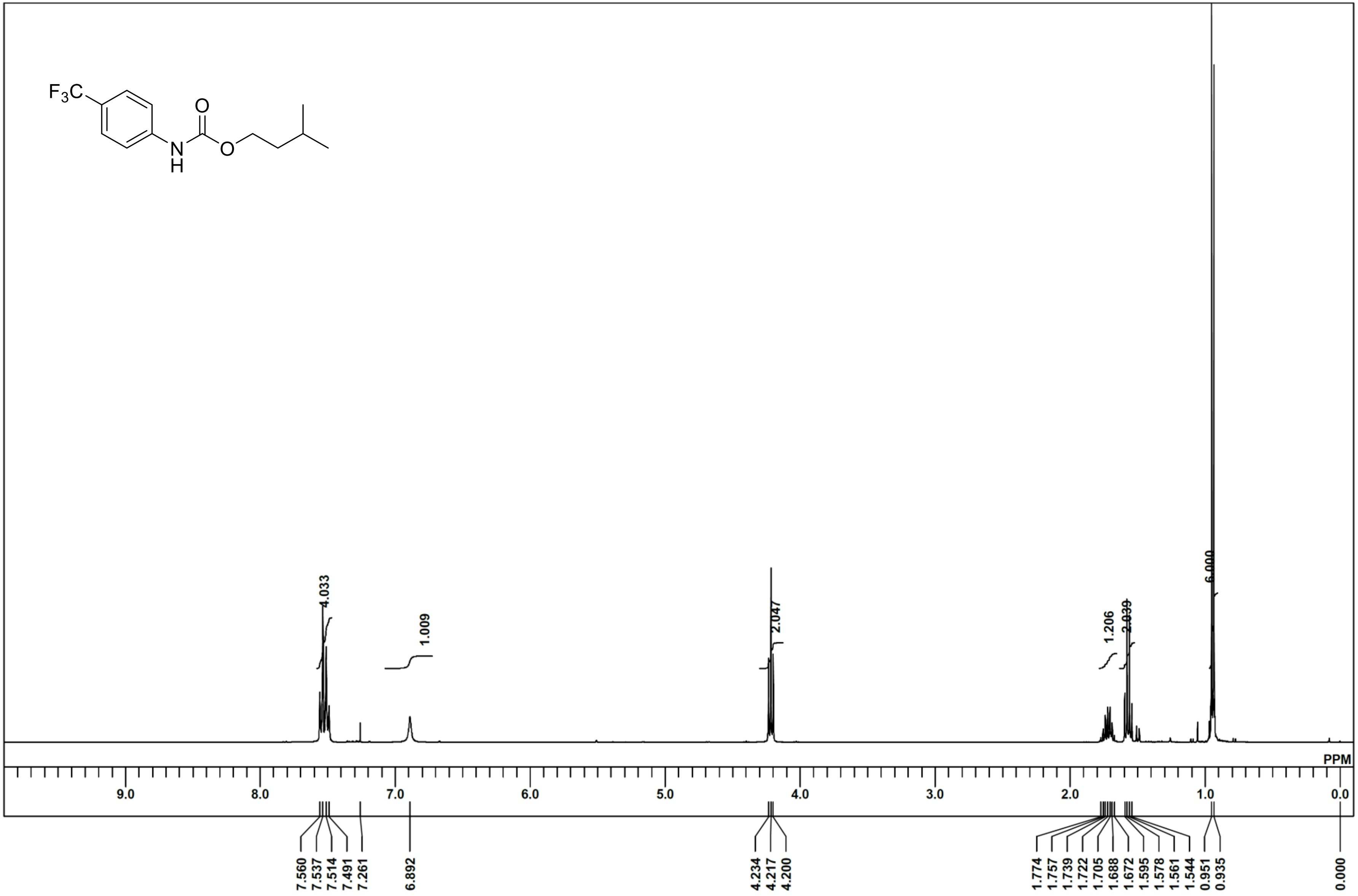

^{13}C NMR spectrum of **2d**

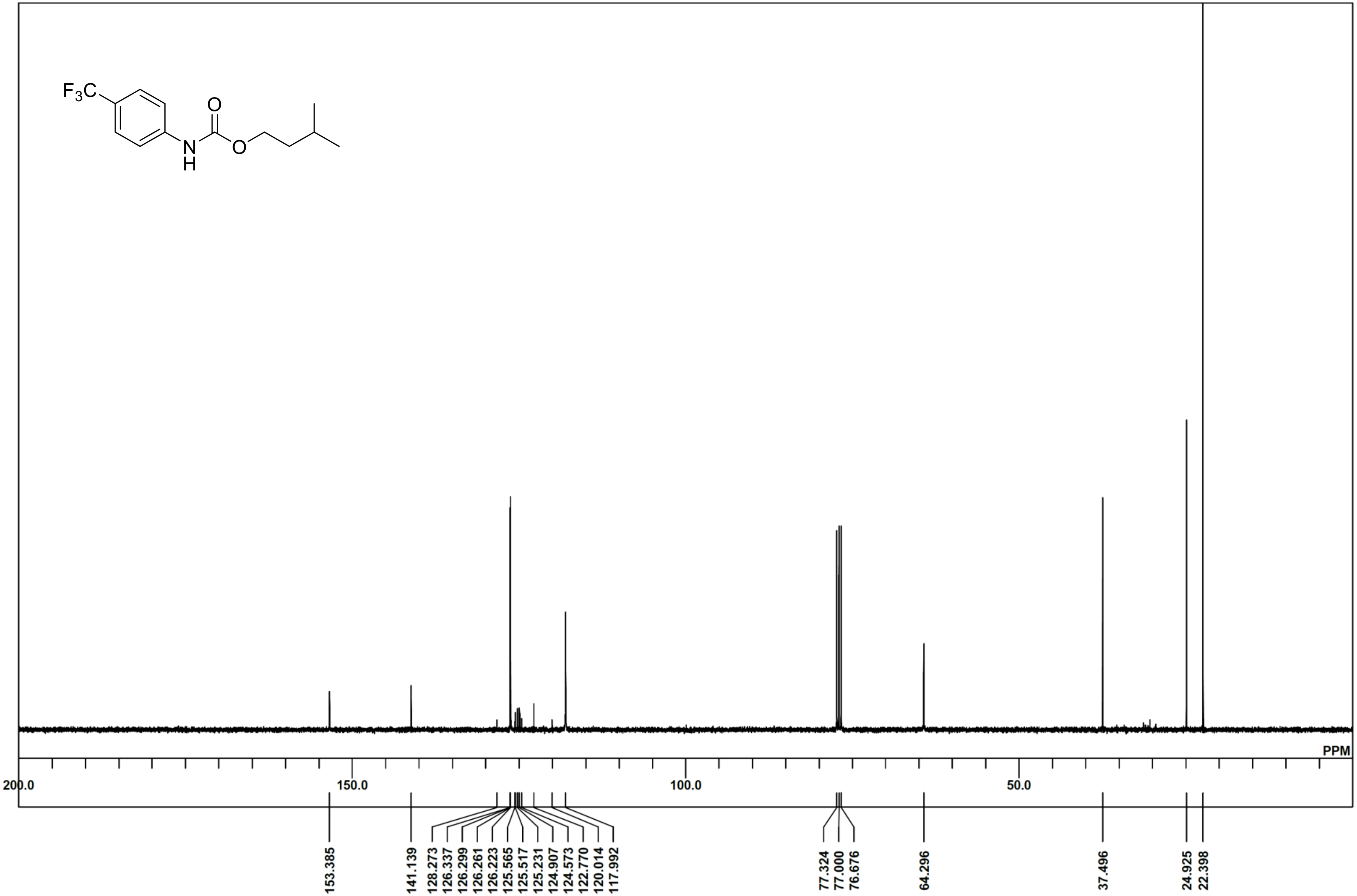

^{19}F NMR spectrum of **2d**

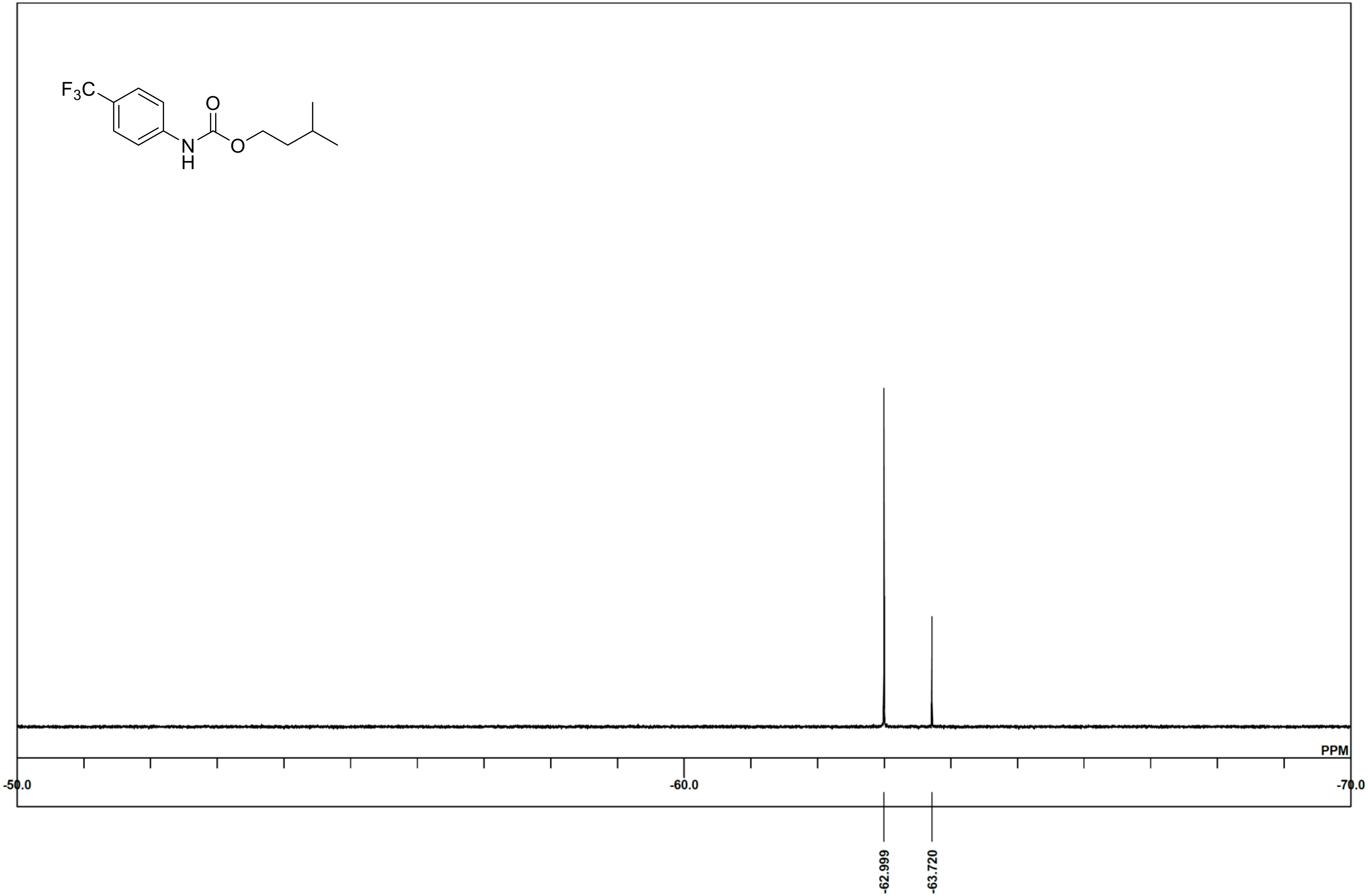

^{1}H NMR spectrum of **2e**

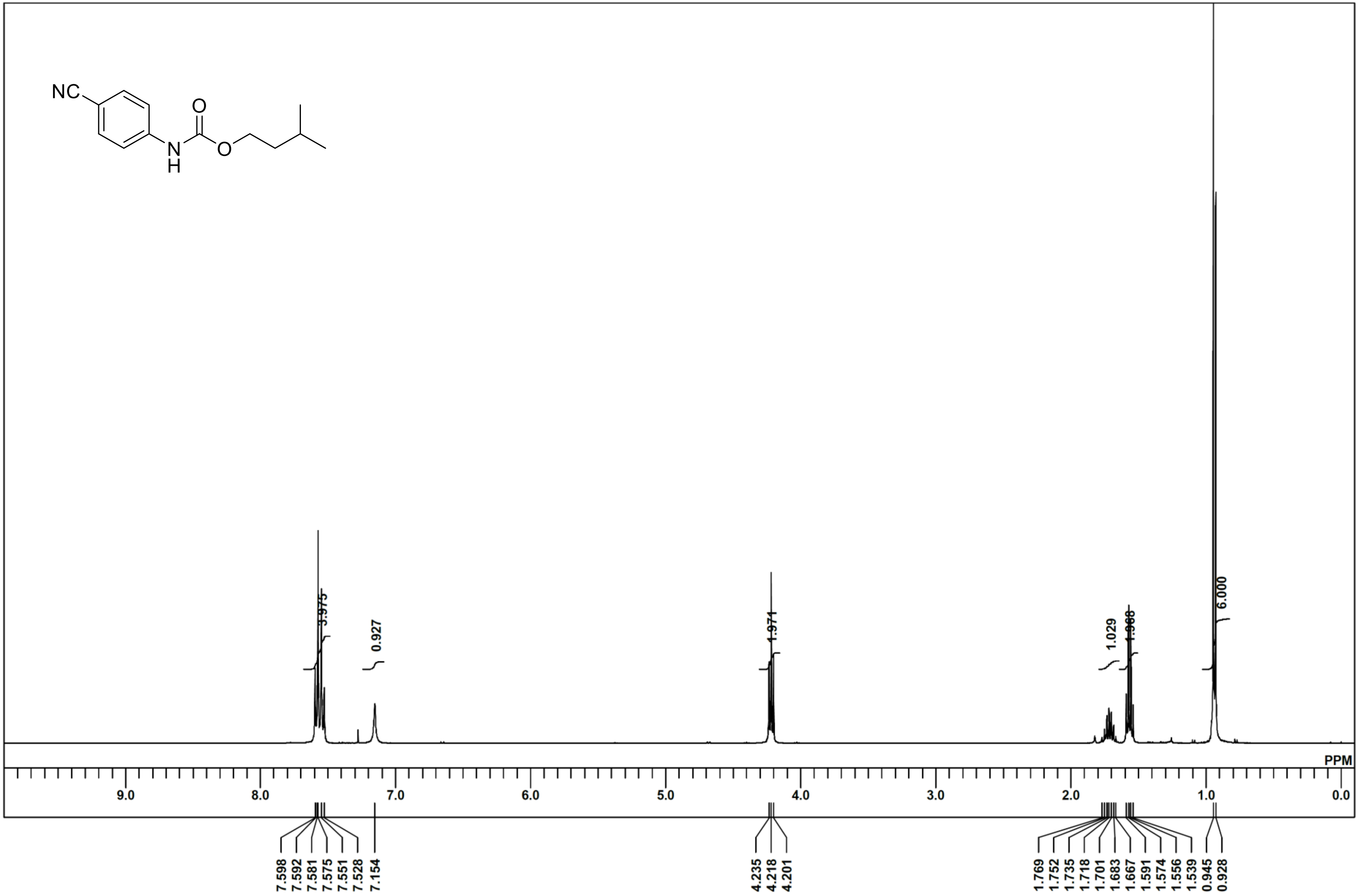

^{13}C NMR spectrum of **2e**

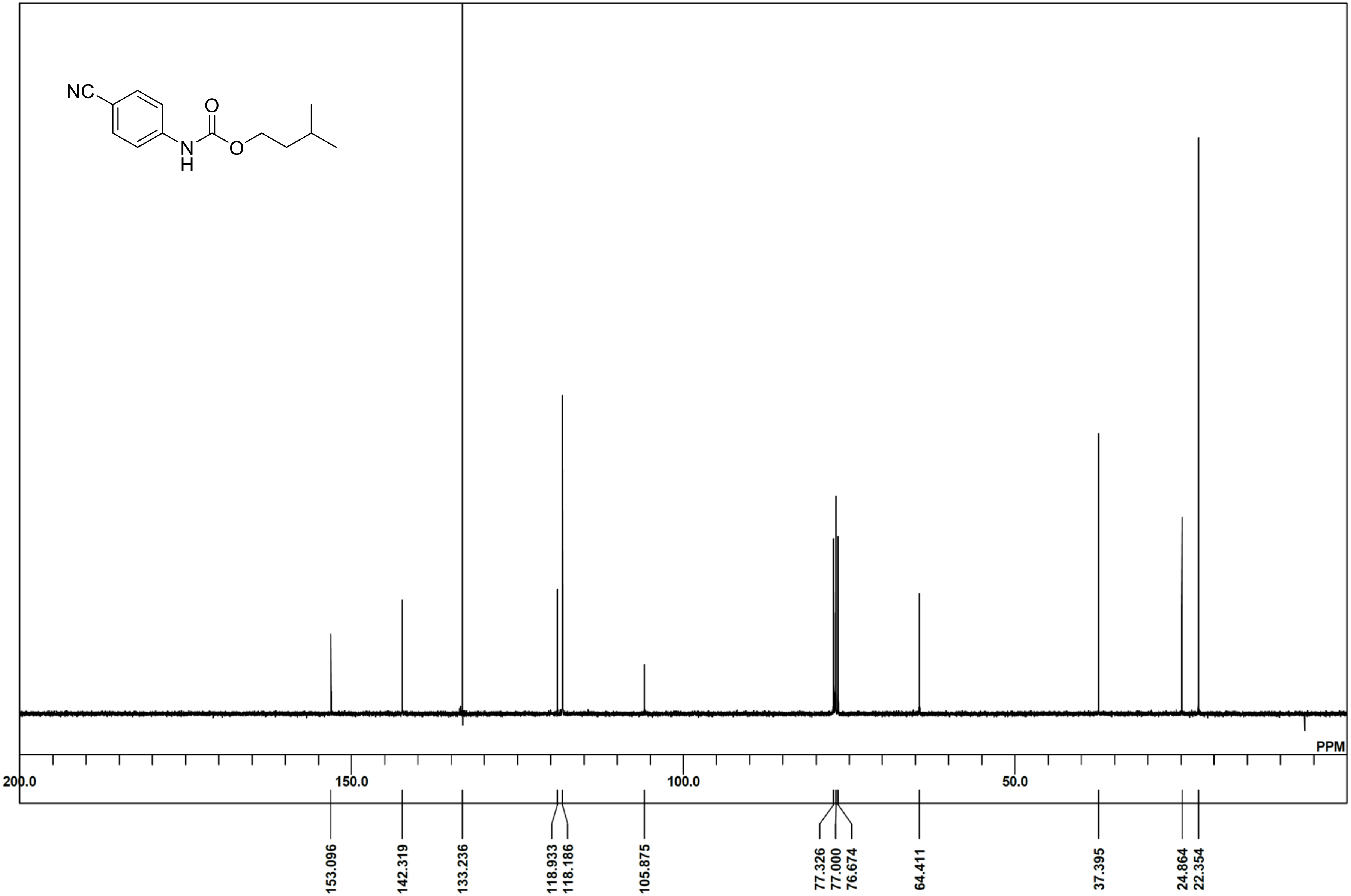

^{1}H NMR spectrum of **2f**

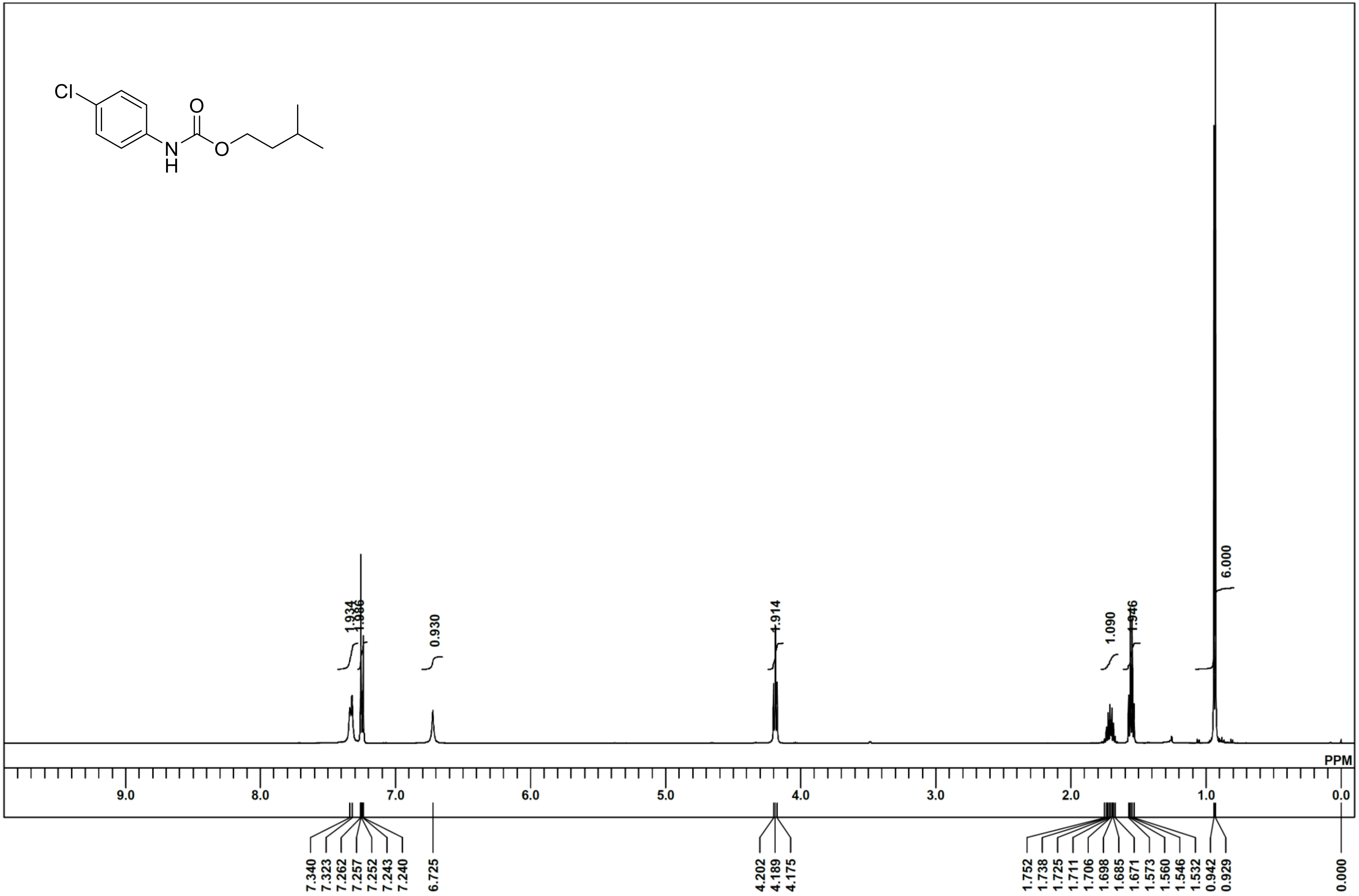

^{13}C NMR spectrum of **2f**

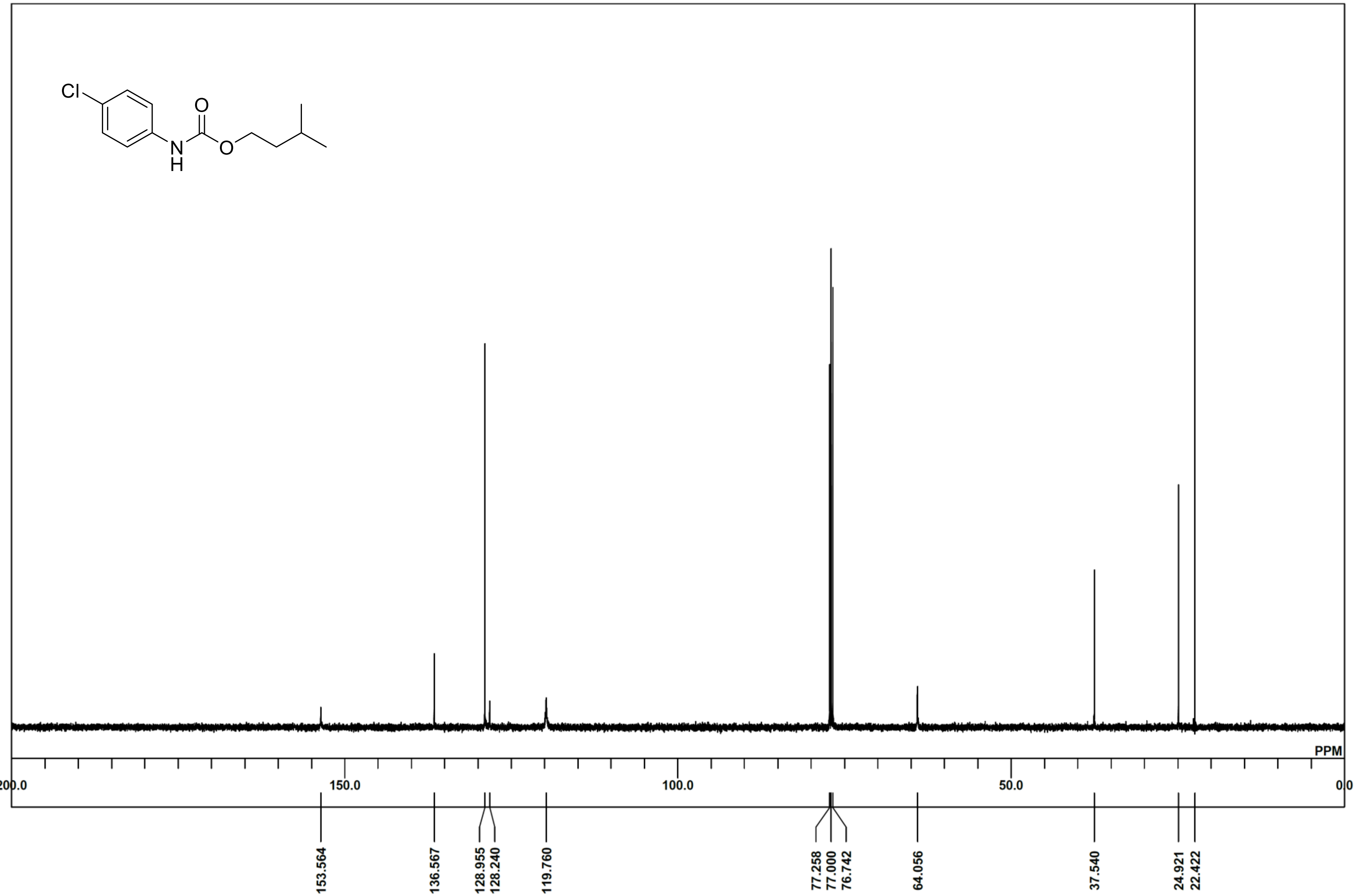

^{1}H NMR spectrum of **2g**

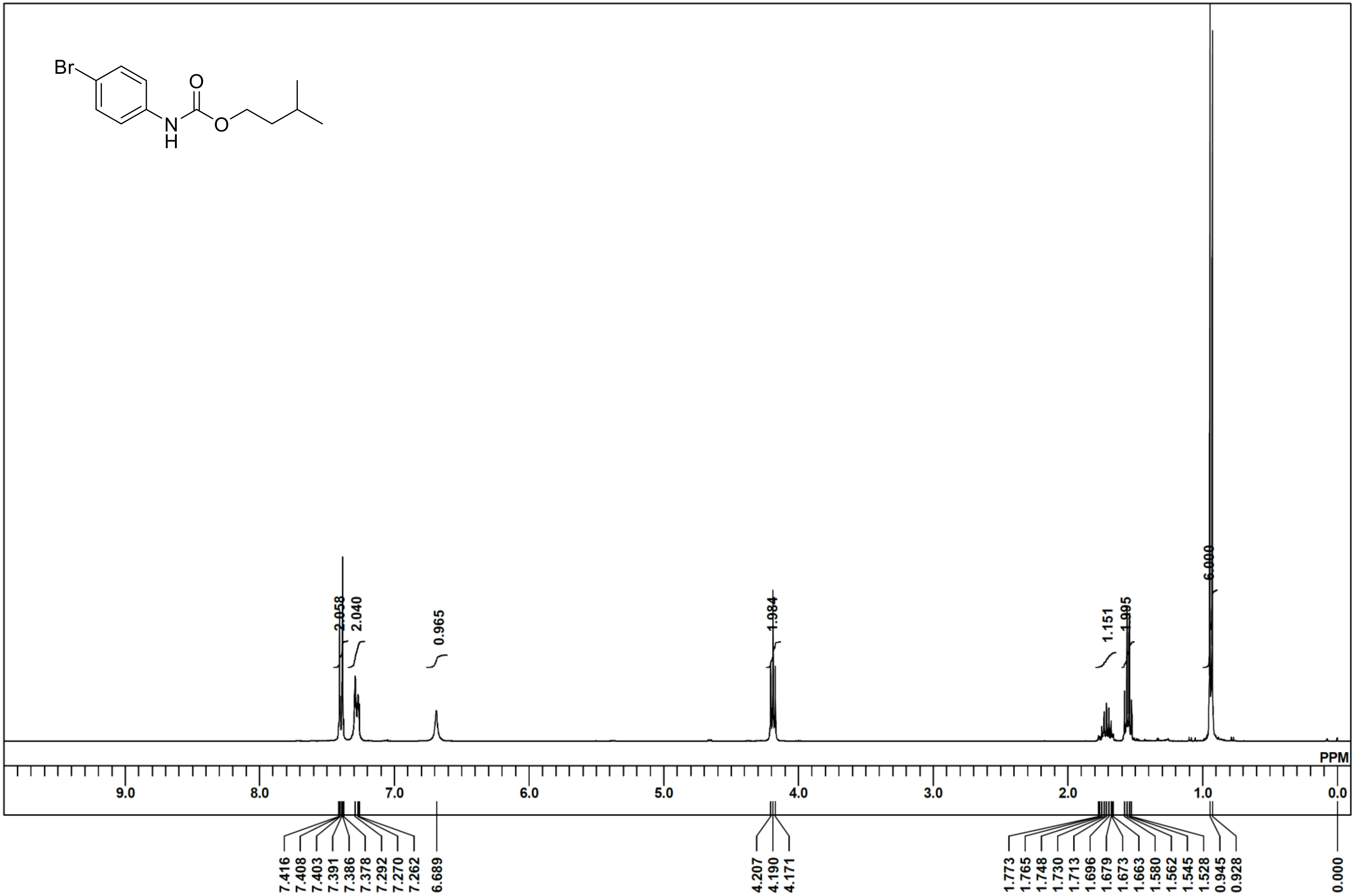

^{13}C NMR spectrum of **2g**

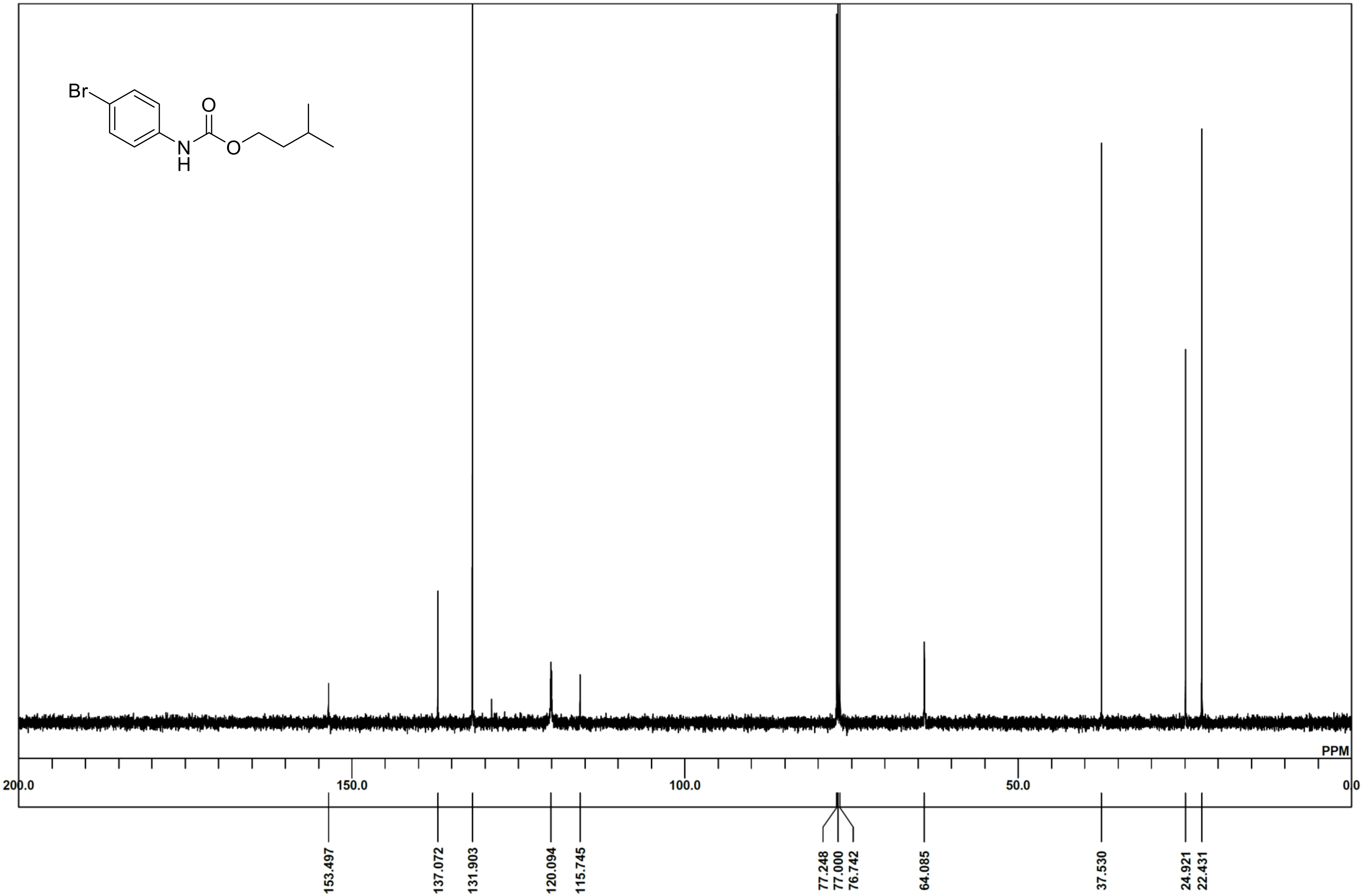

^{1}H NMR spectrum of **2h**

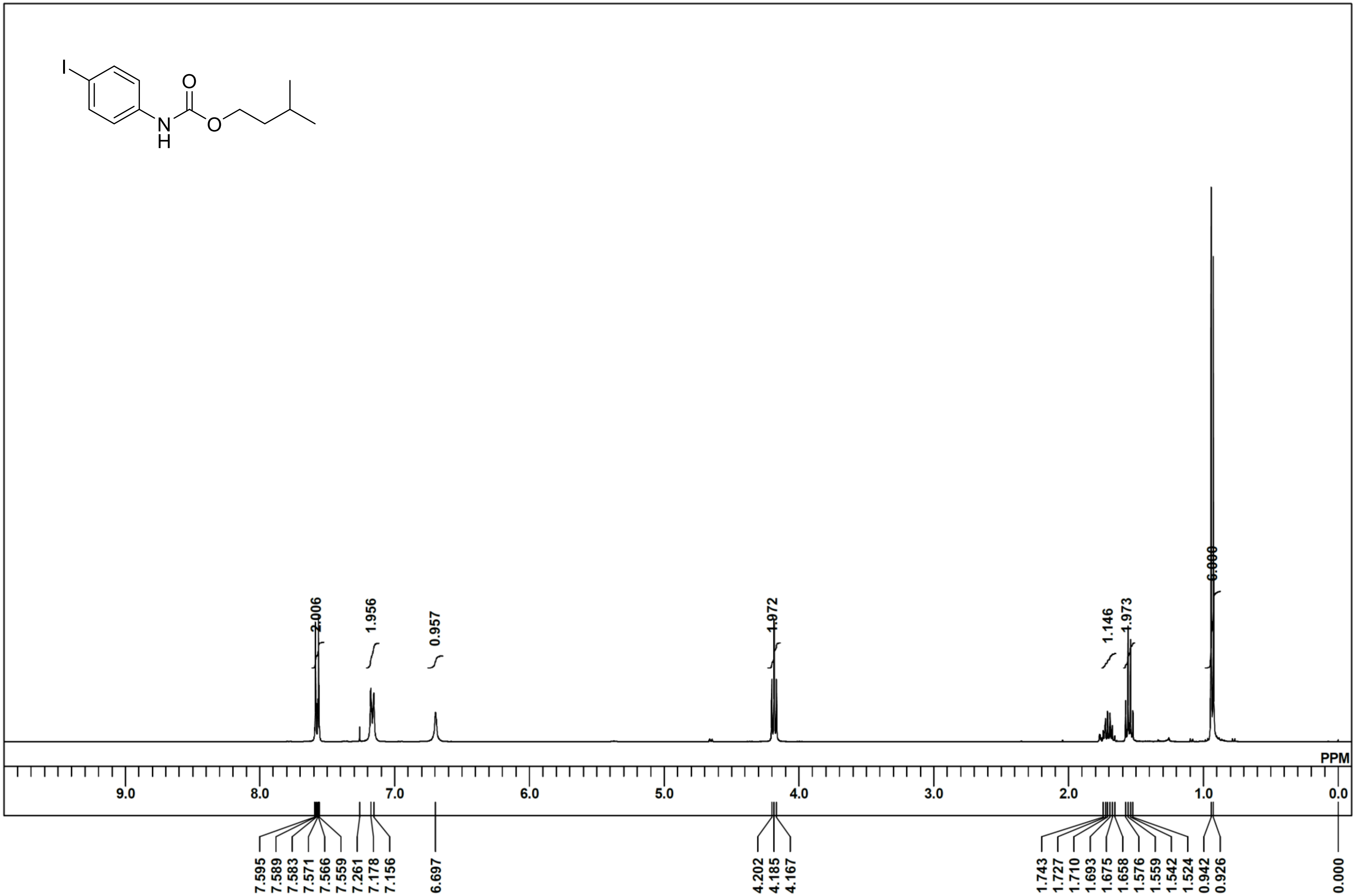

^{13}C NMR spectrum of **2h**

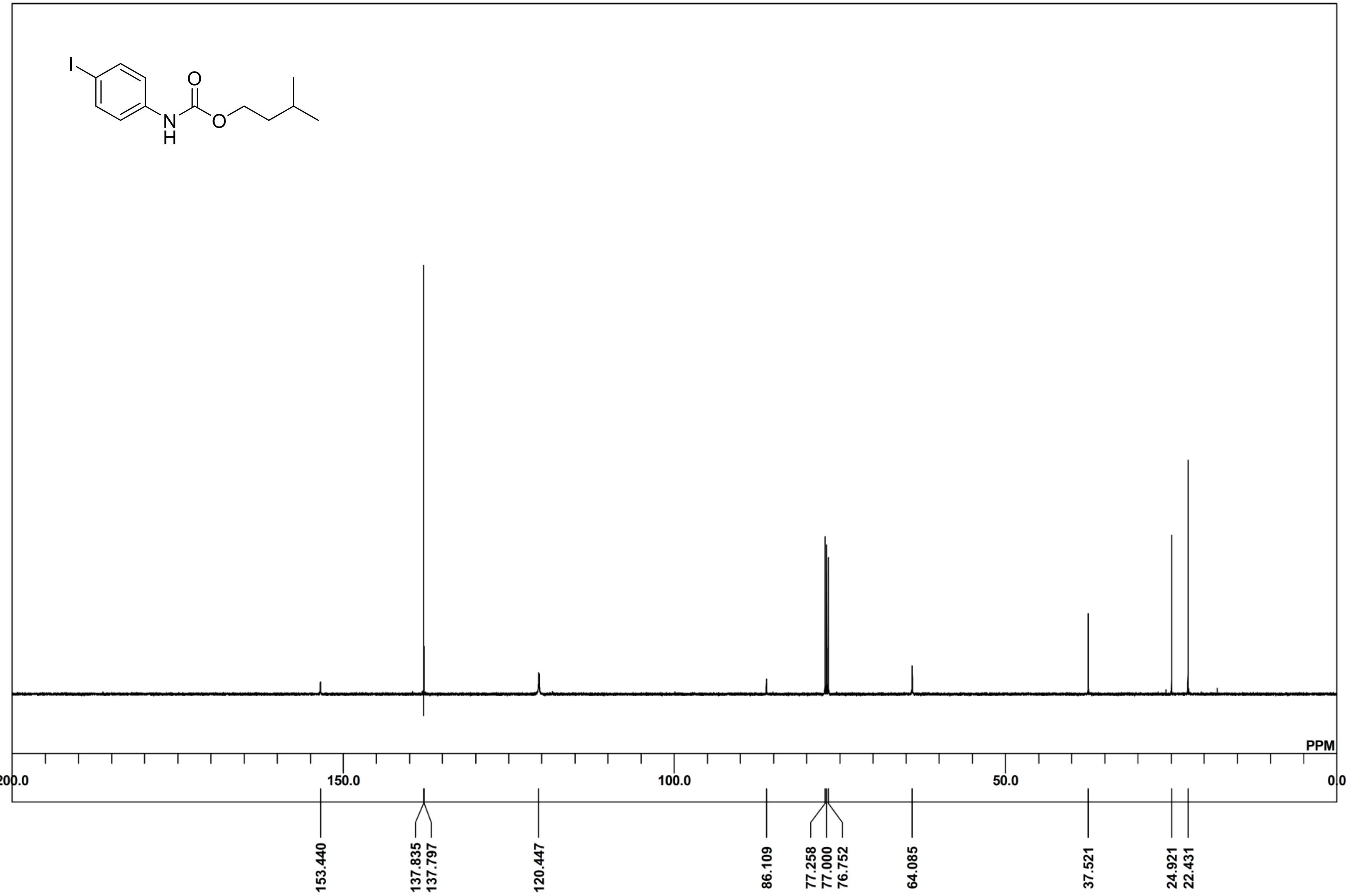

^{1}H NMR spectrum of **2i**

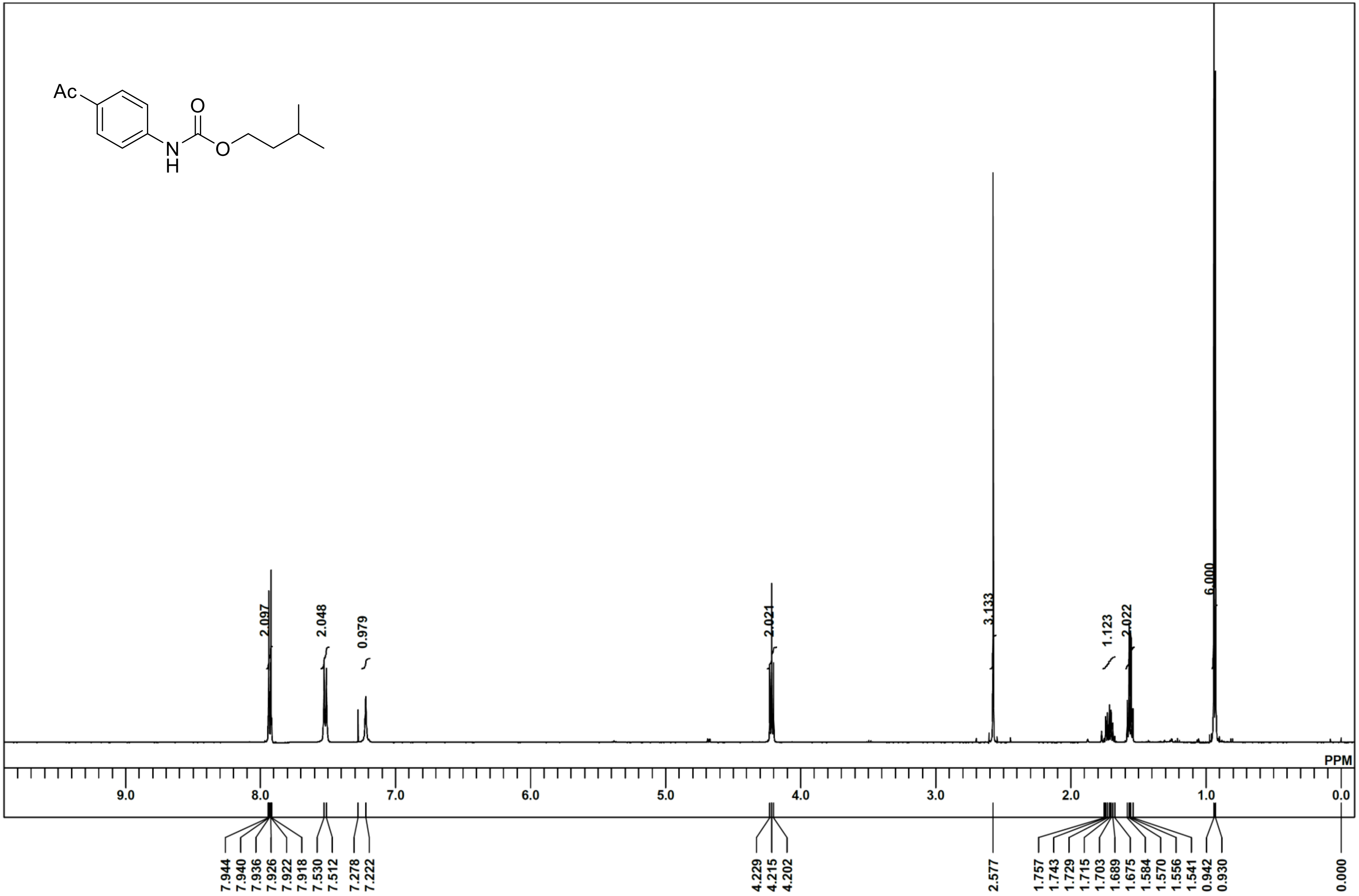

^{13}C NMR spectrum of **2i**

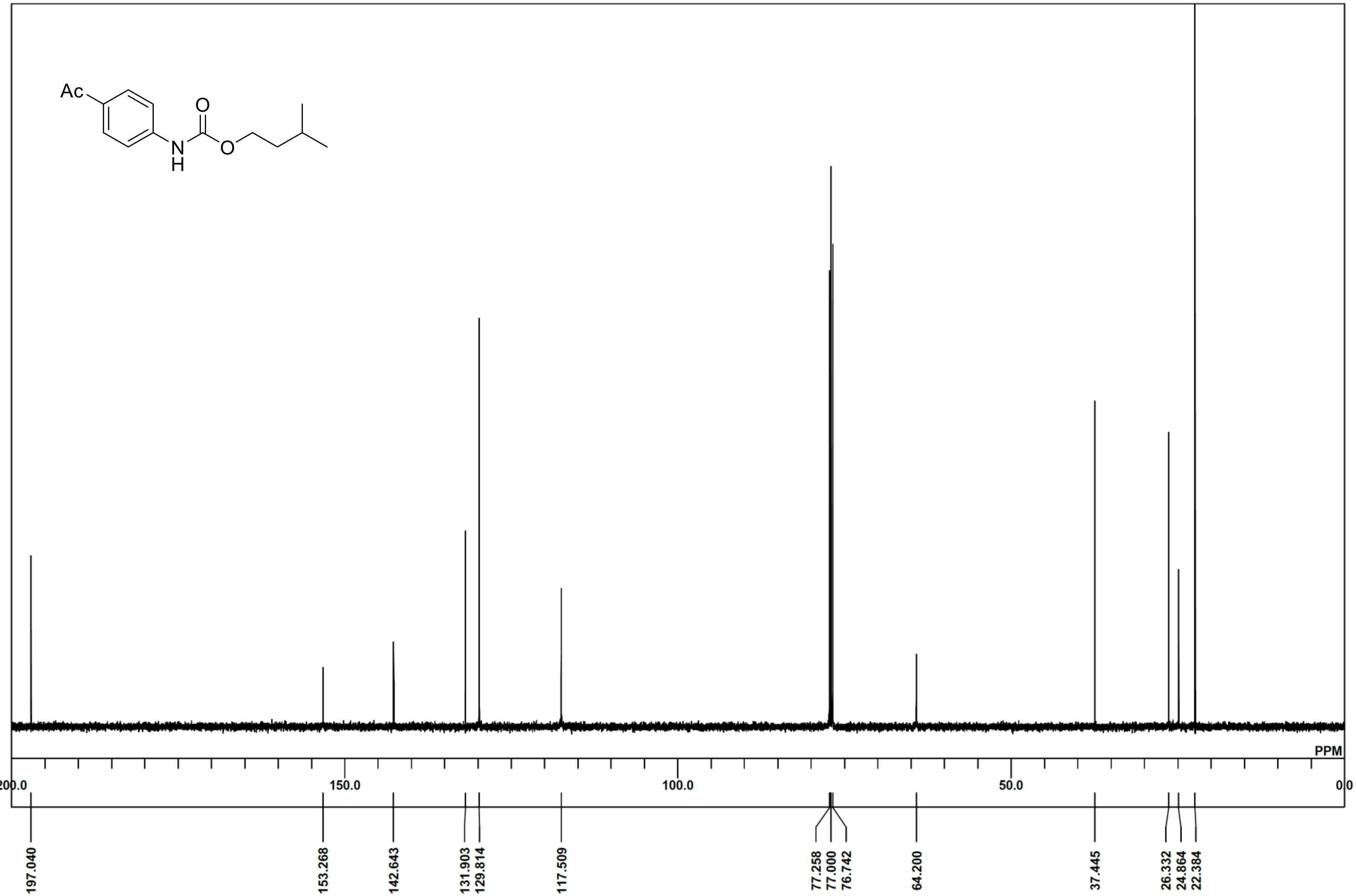

^{1}H NMR spectrum of **2j**

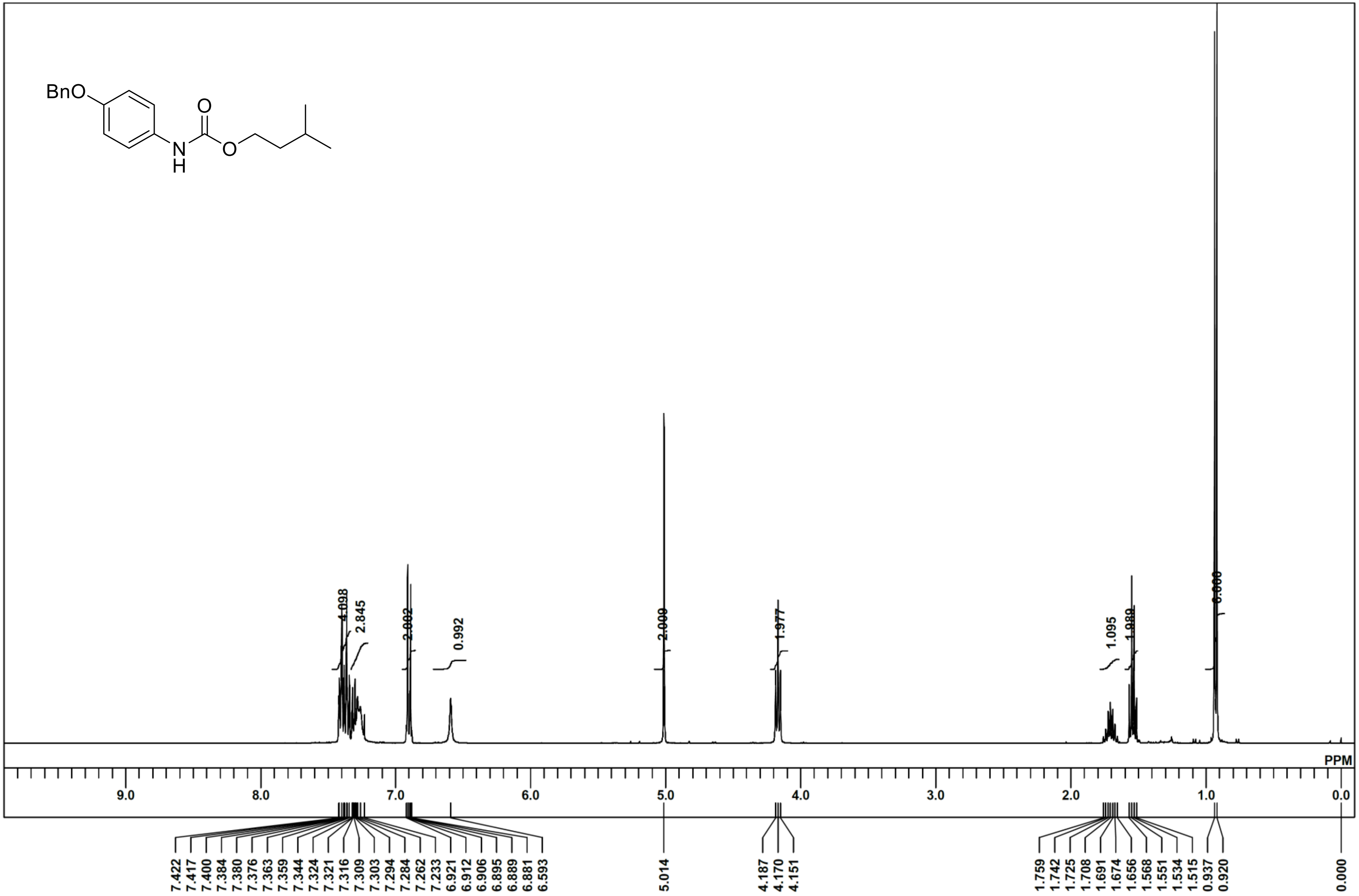

^{13}C NMR spectrum of **2j**

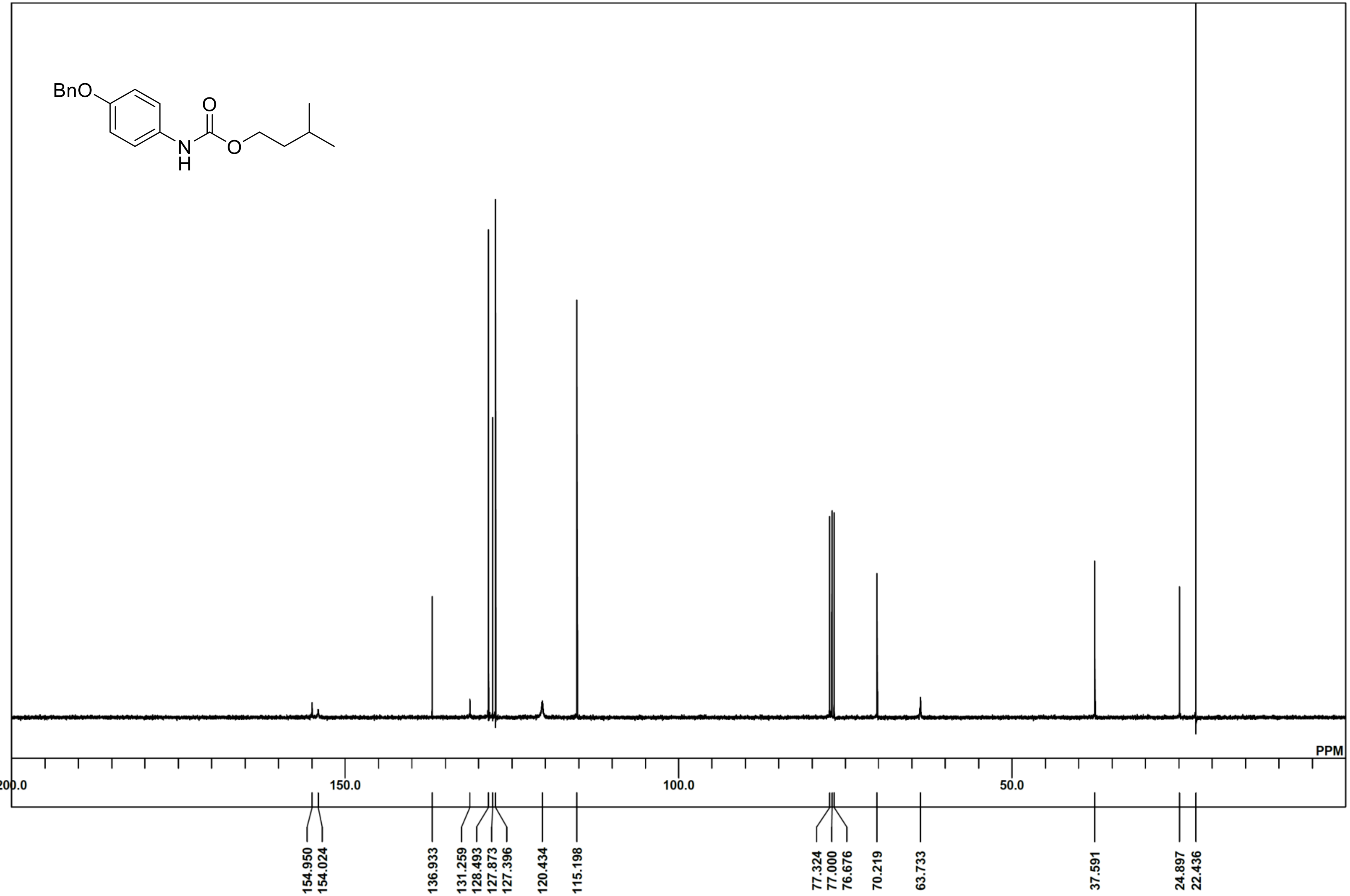

^{1}H NMR spectrum of **2k**

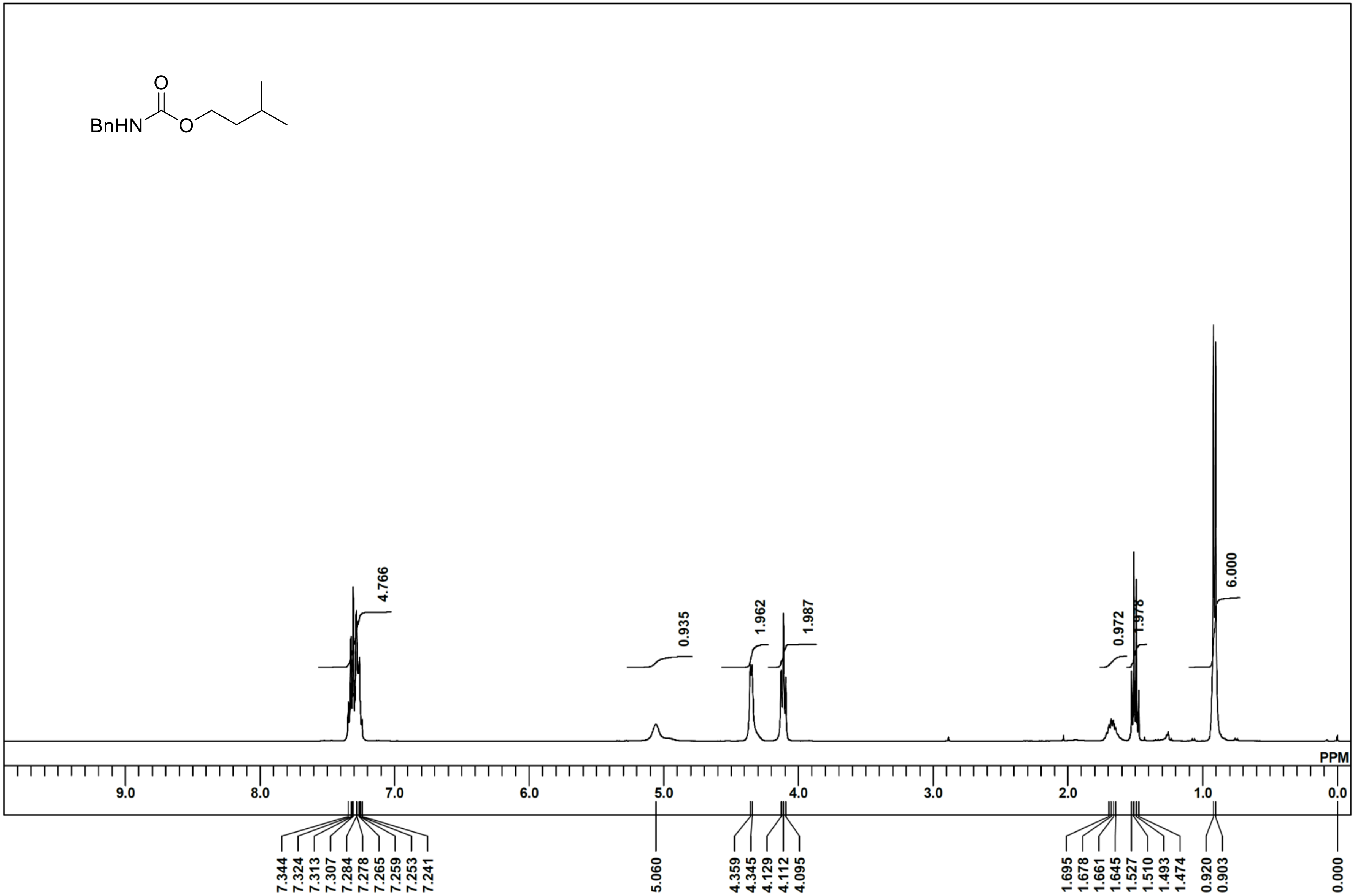

^{13}C NMR spectrum of **2k**

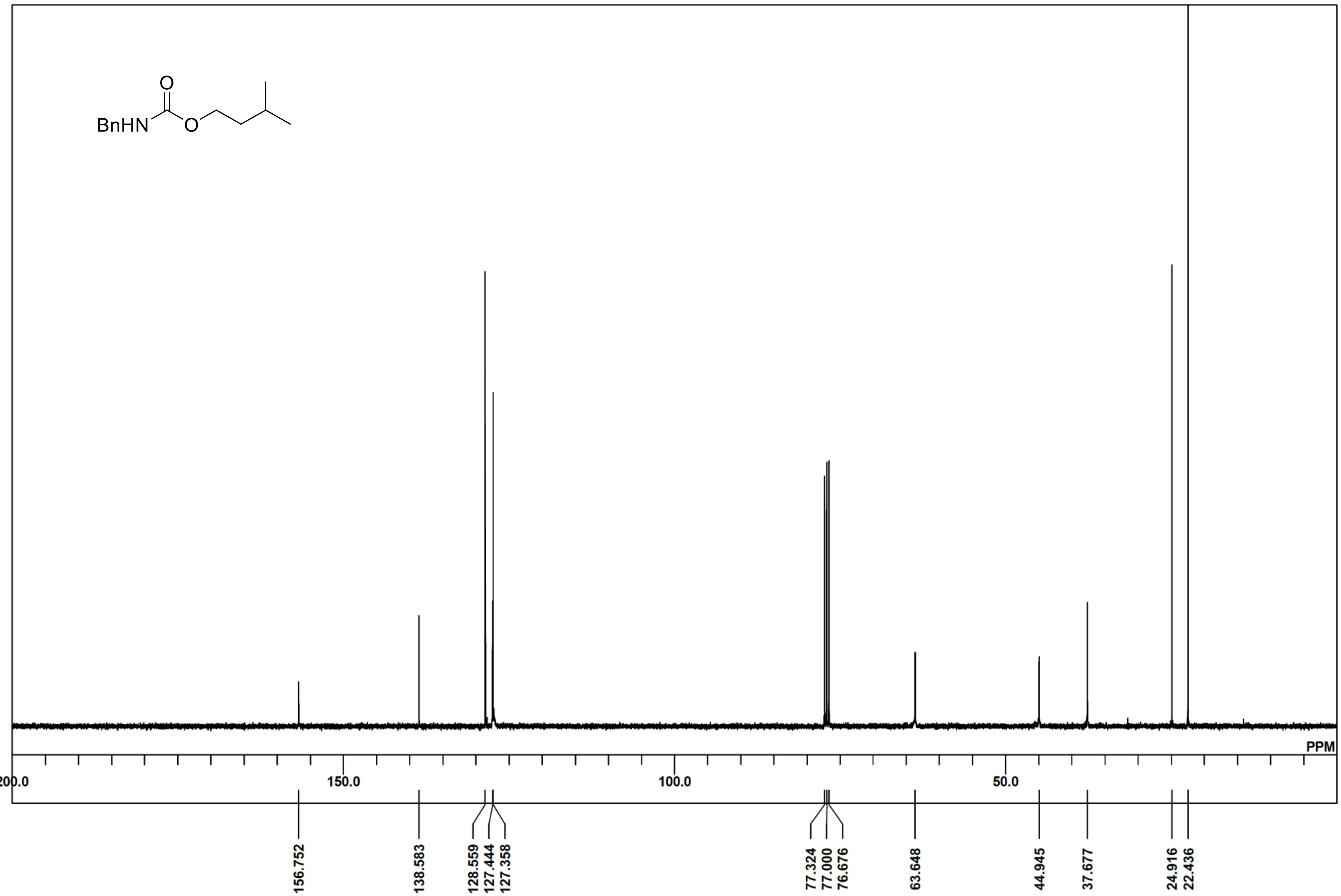

^{1}H NMR spectrum of **2l**

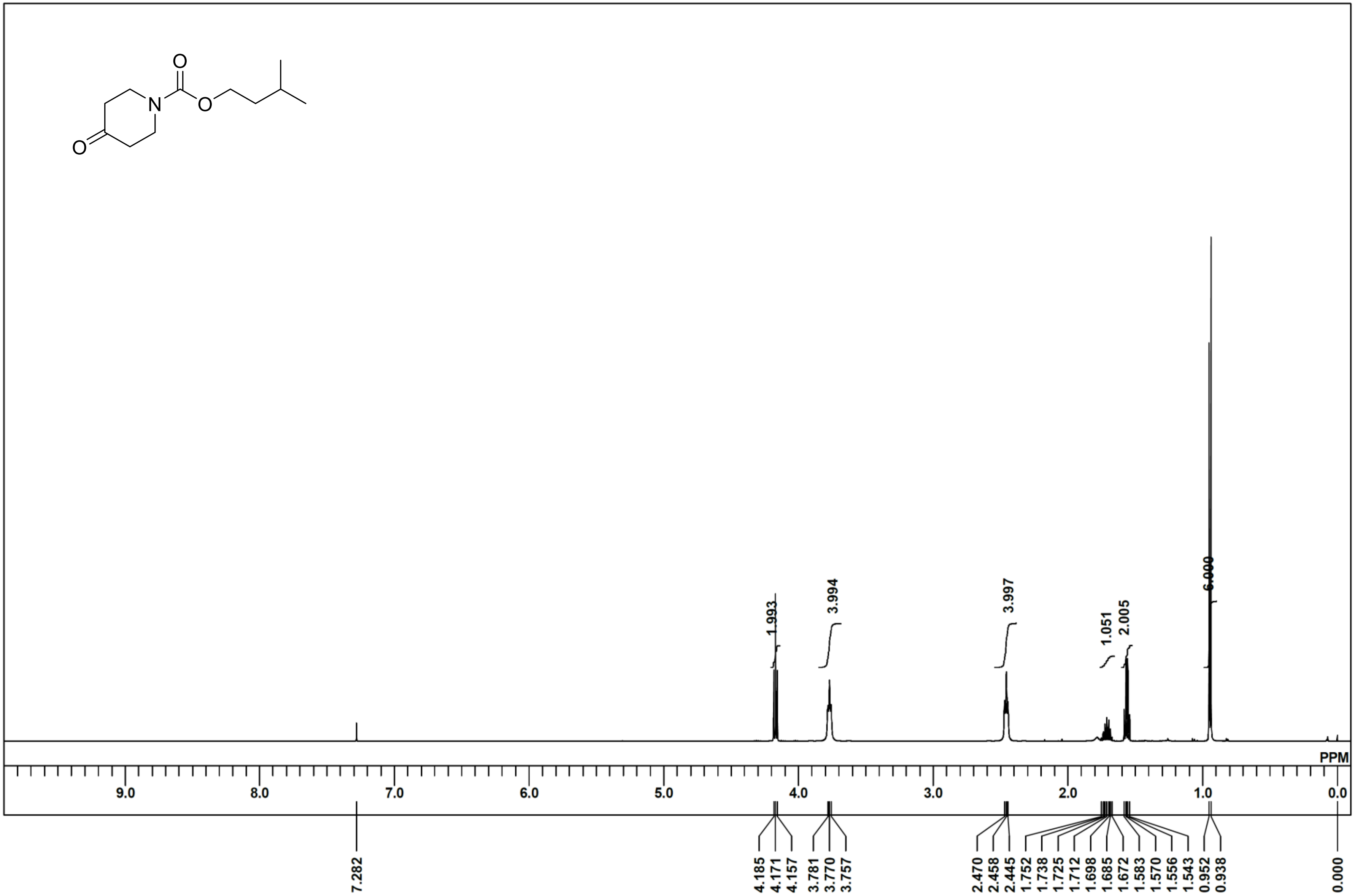

^{13}C NMR spectrum of **2l**

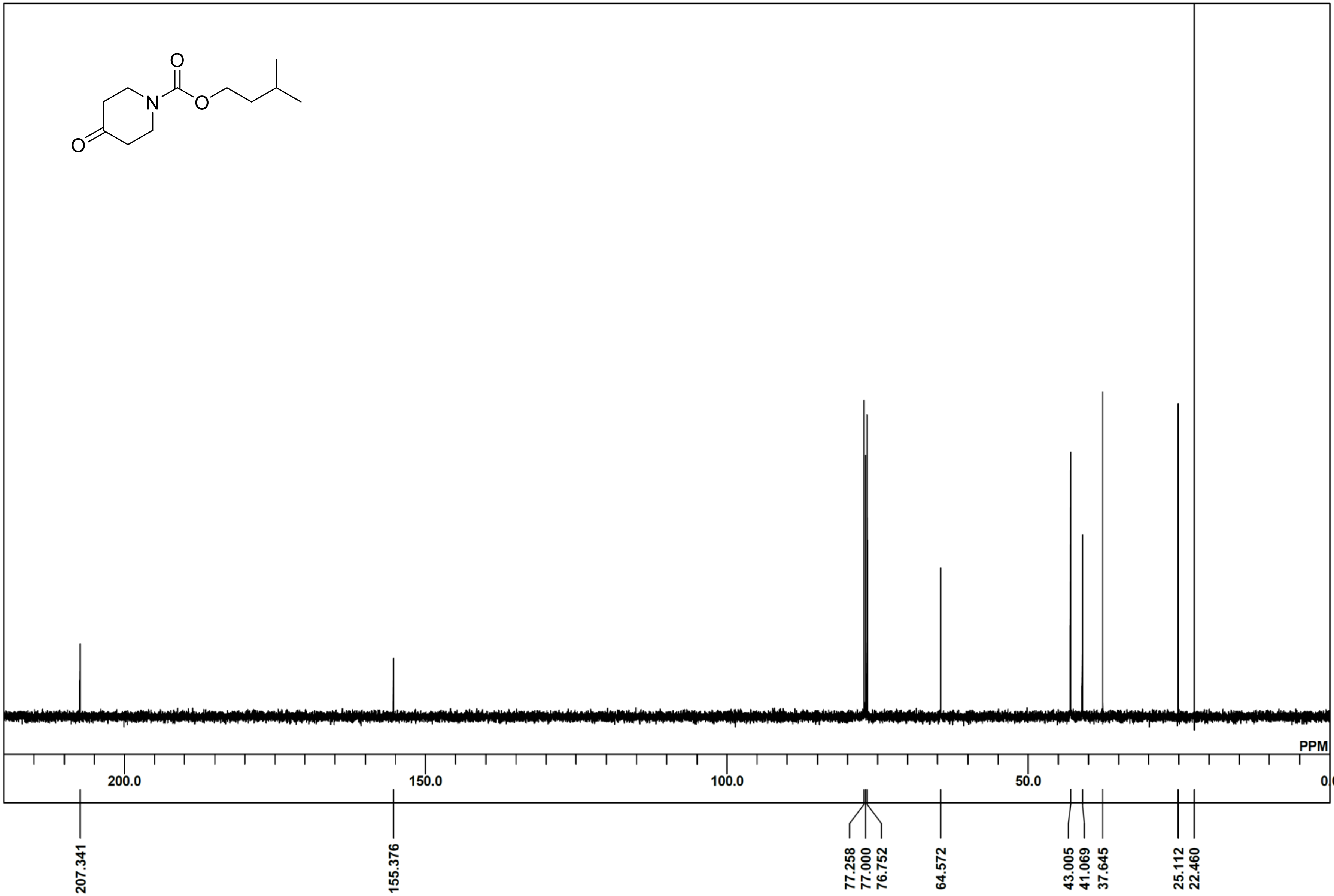

^{1}H NMR spectrum of **2m**

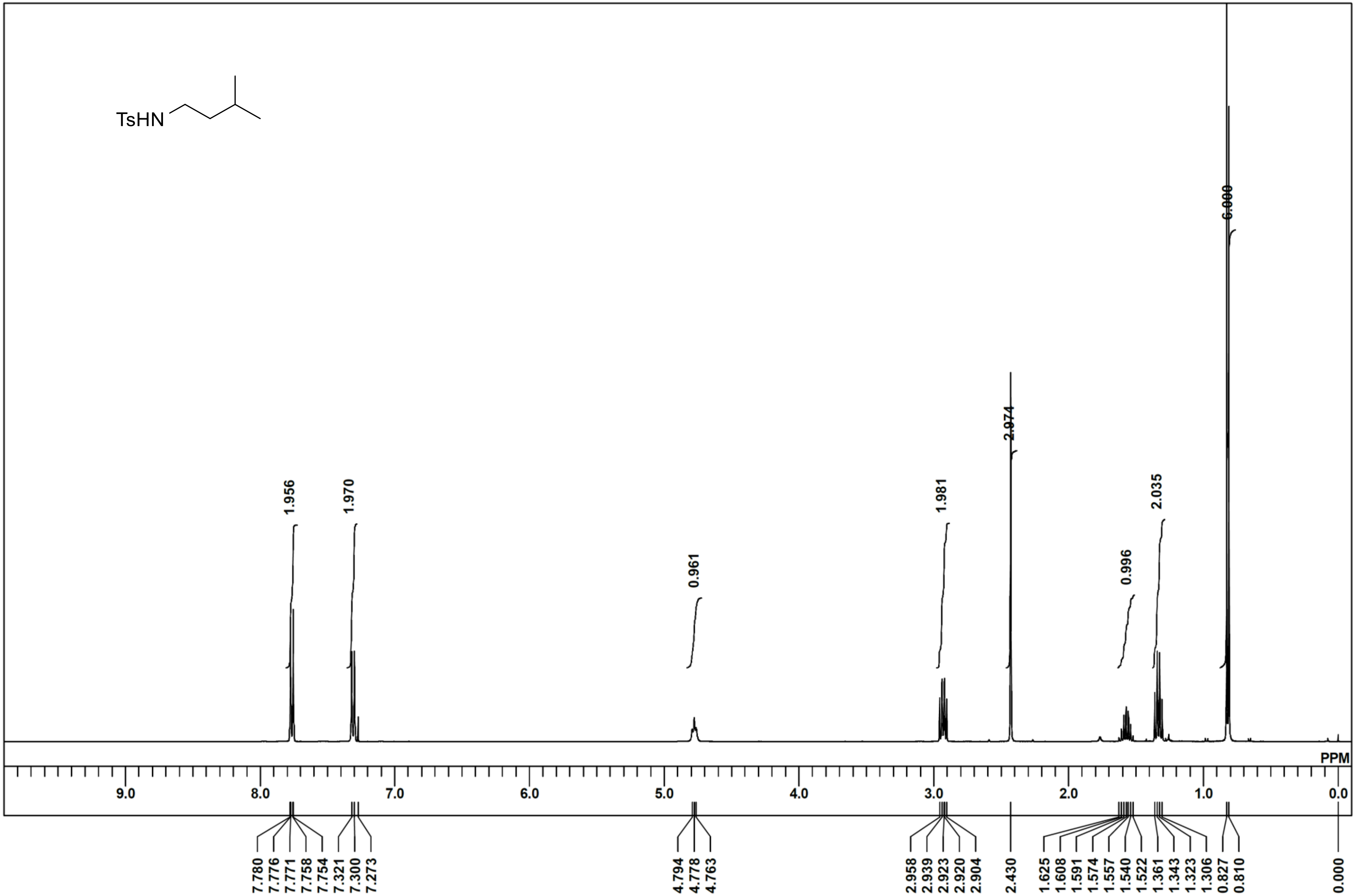

^{13}C NMR spectrum of **2m**

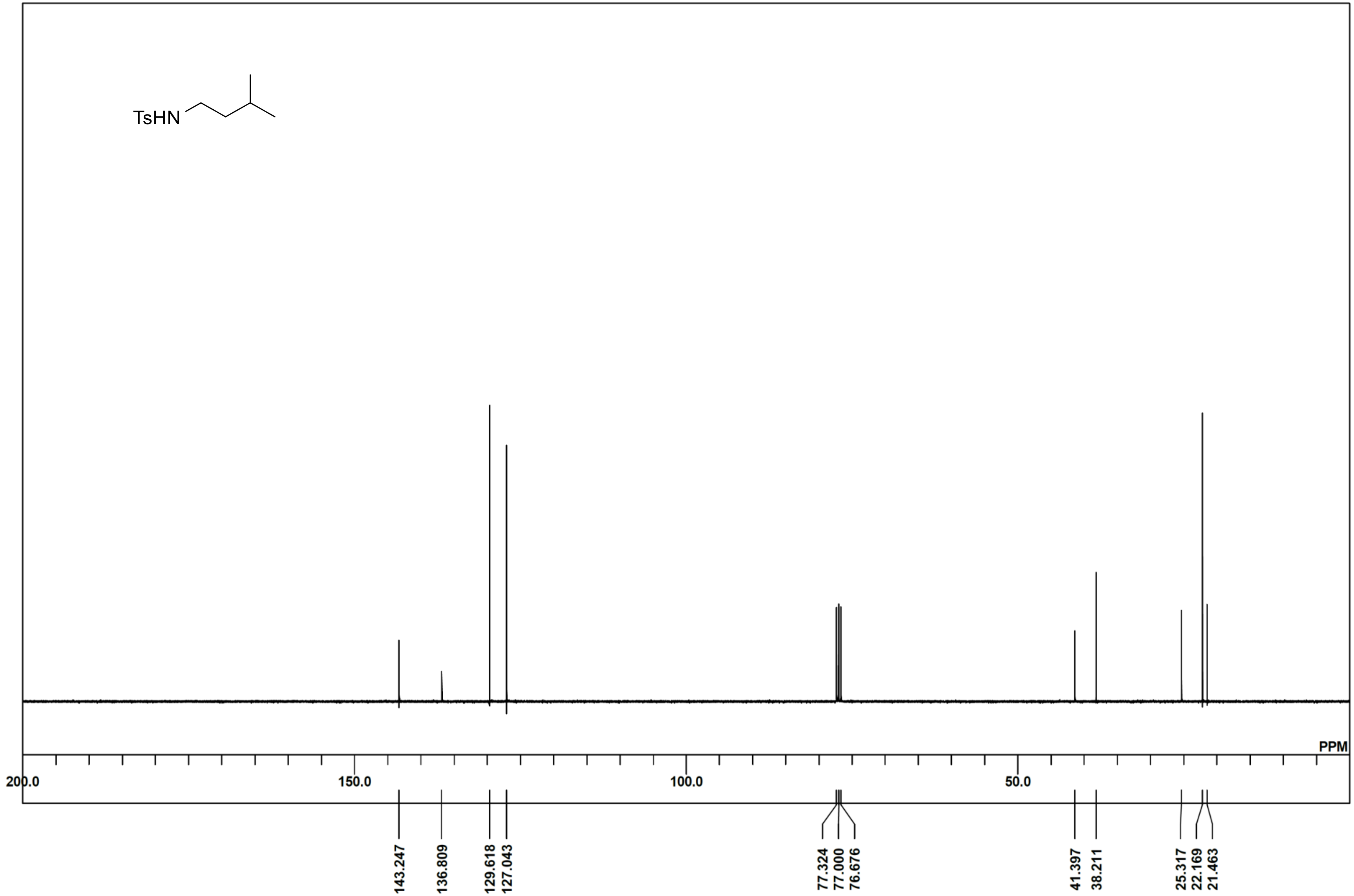

^{1}H NMR spectrum of **2n**

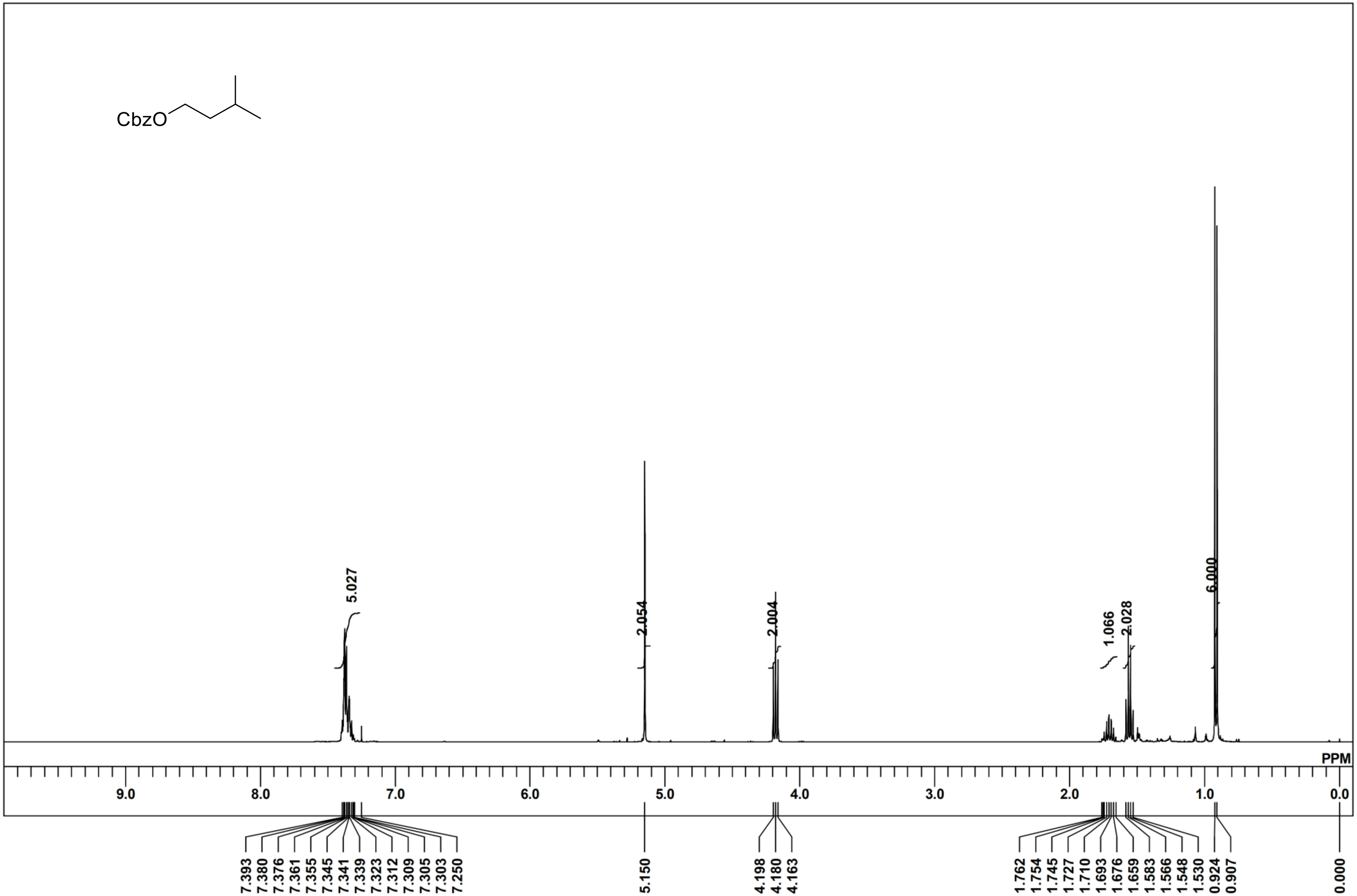

^{13}C NMR spectrum of **2n**

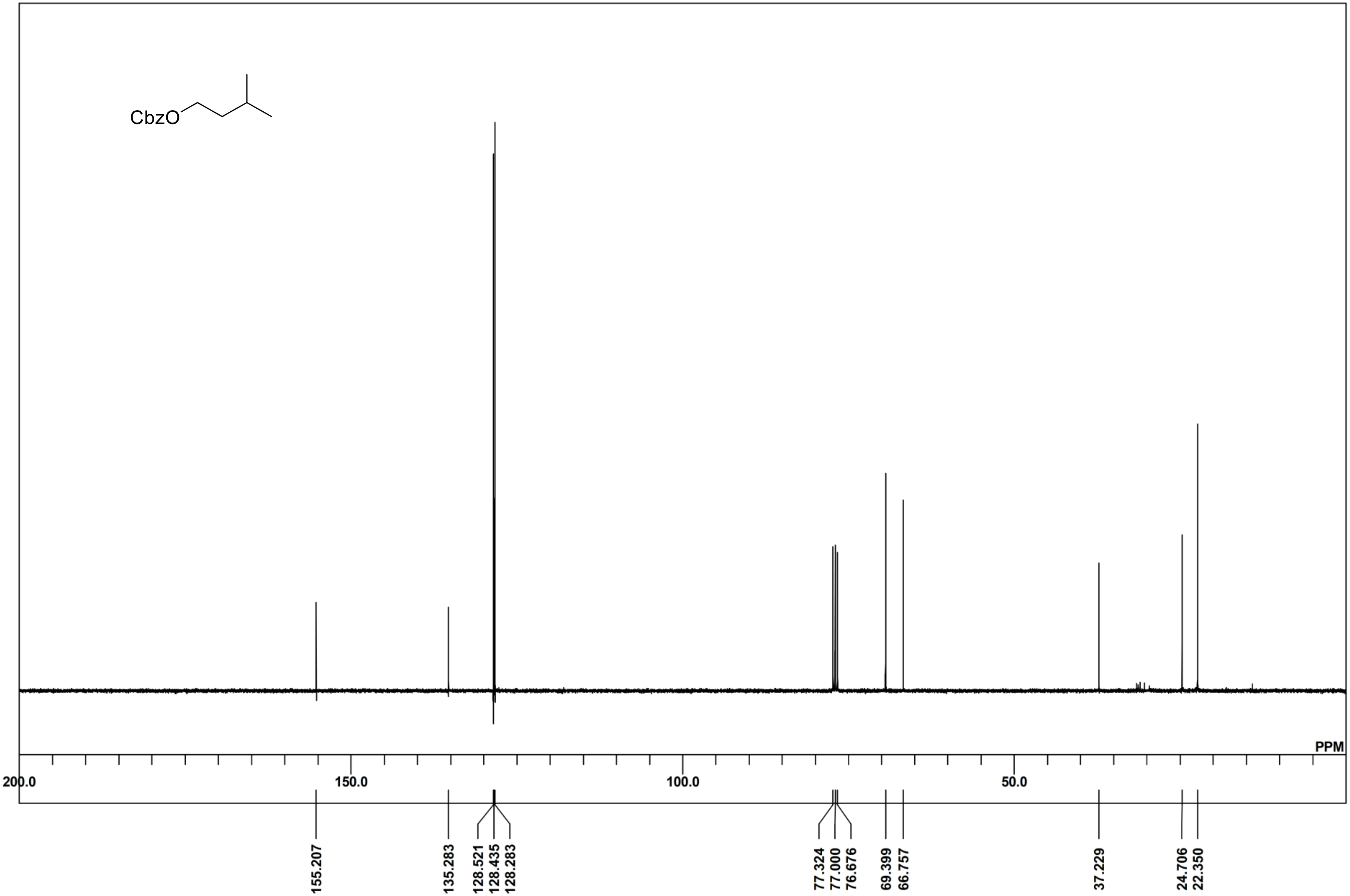

^{1}H NMR spectrum of **2o**

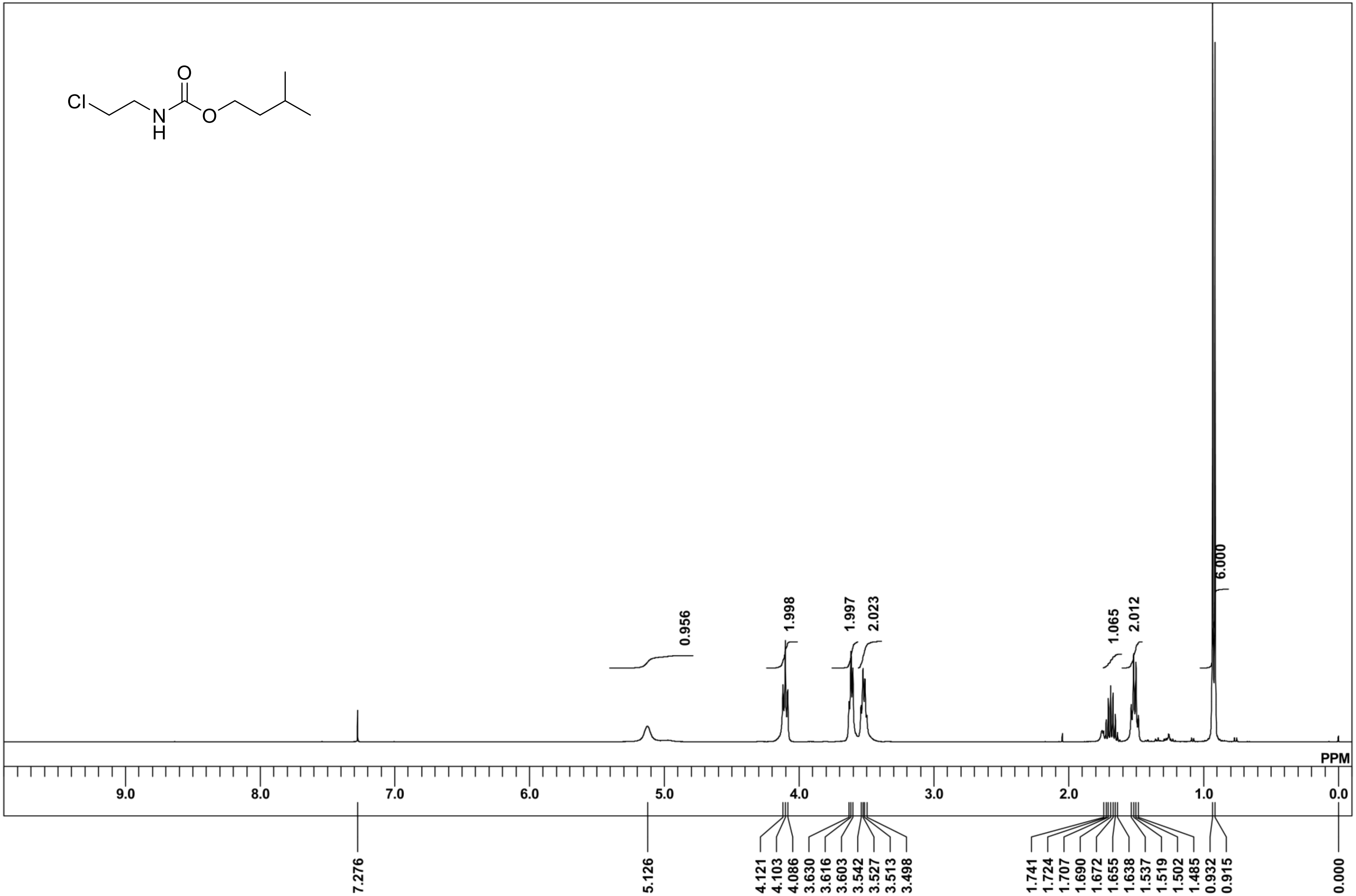

^{13}C NMR spectrum of **2o**

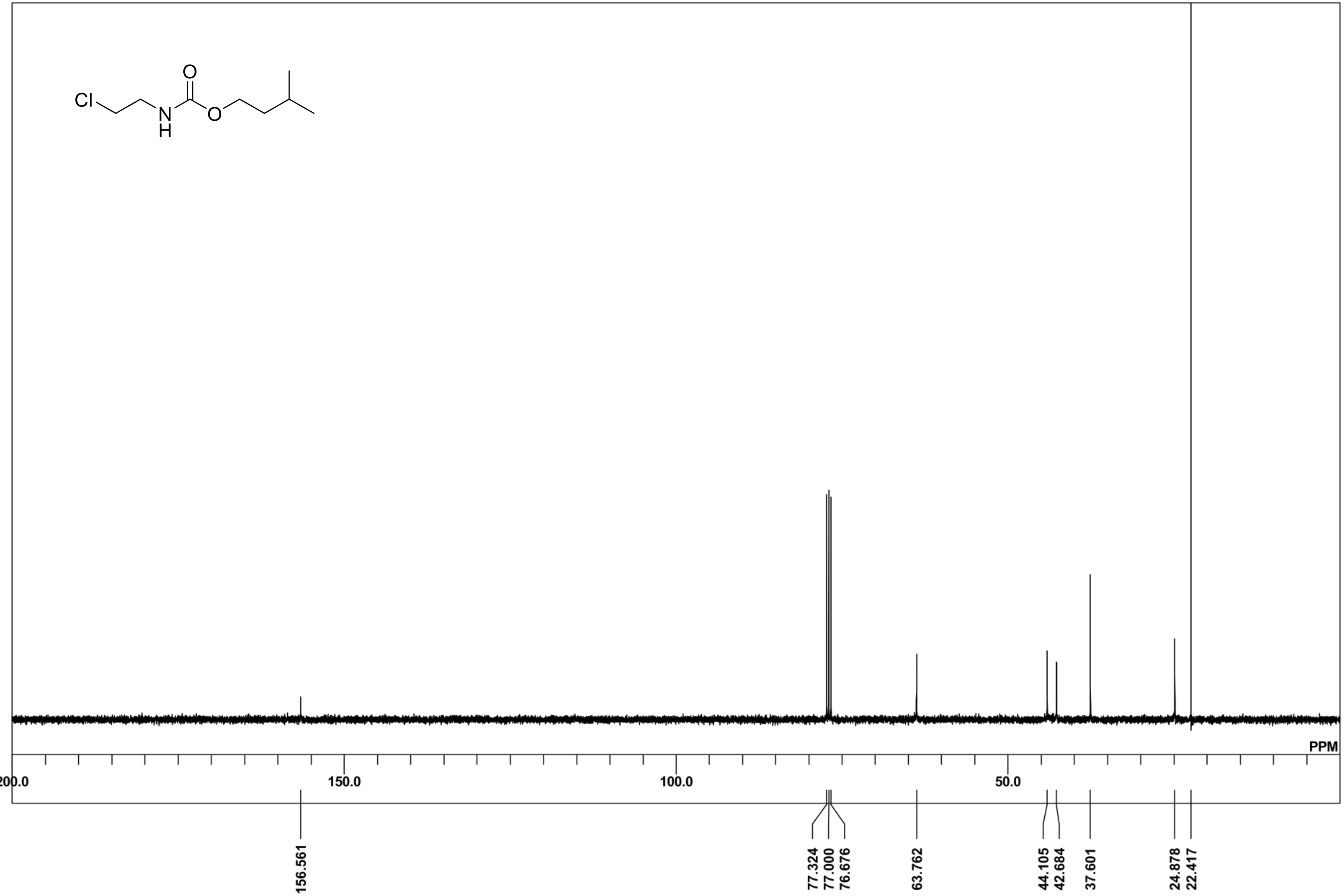

^{1}H NMR spectrum of **2p**

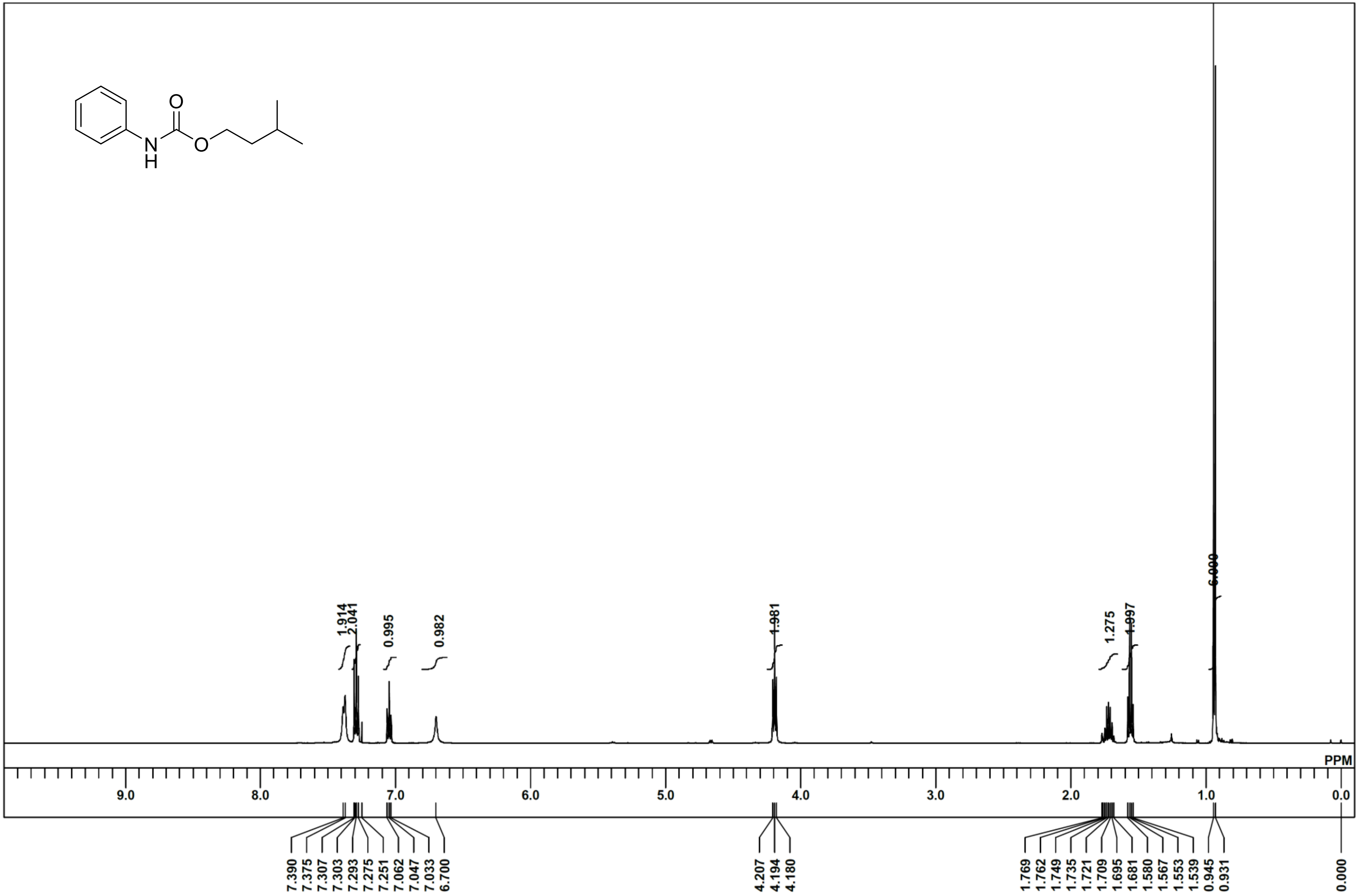

^{13}C NMR spectrum of **2p**

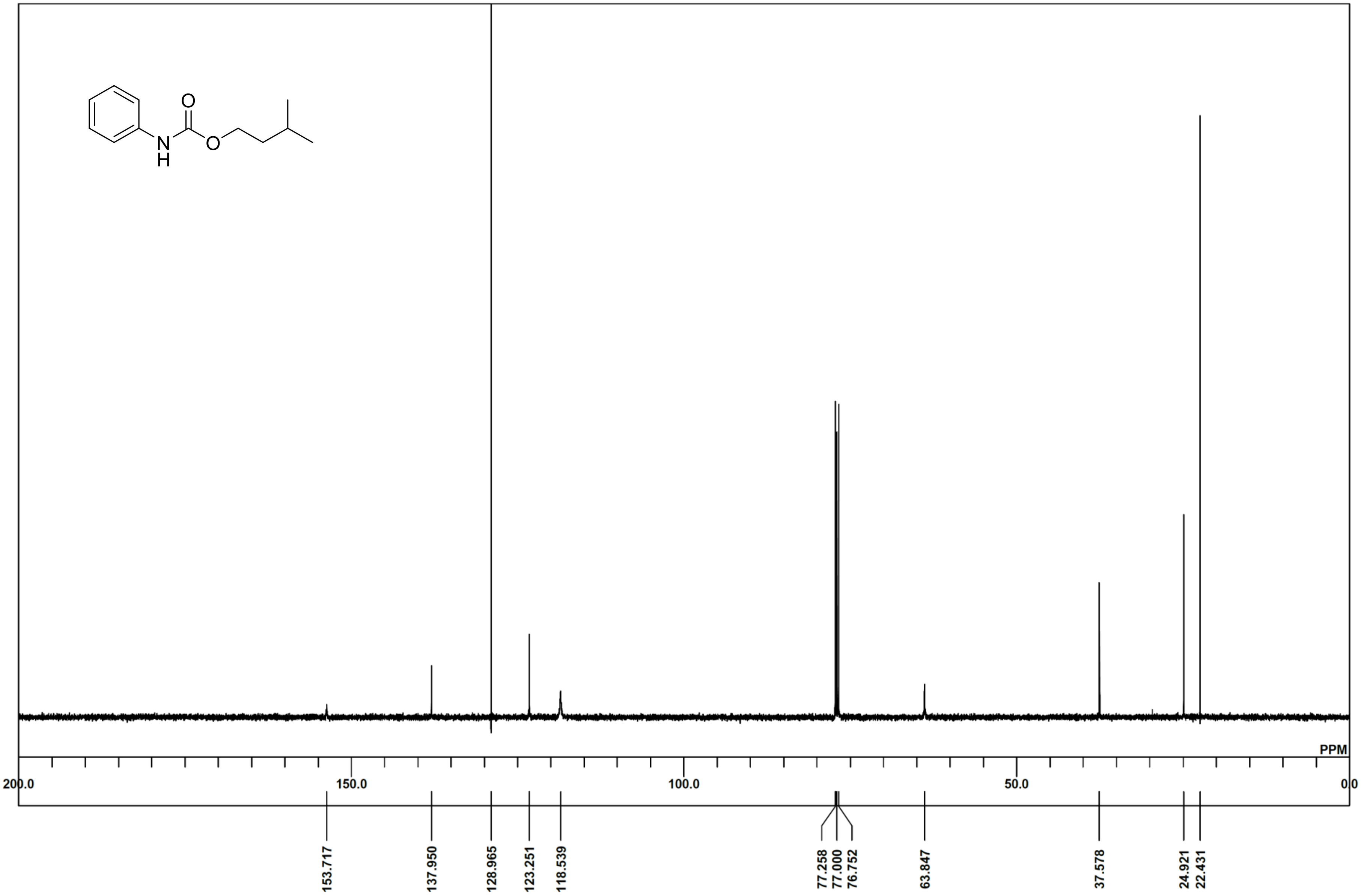

^{1}H NMR spectrum of **2q**

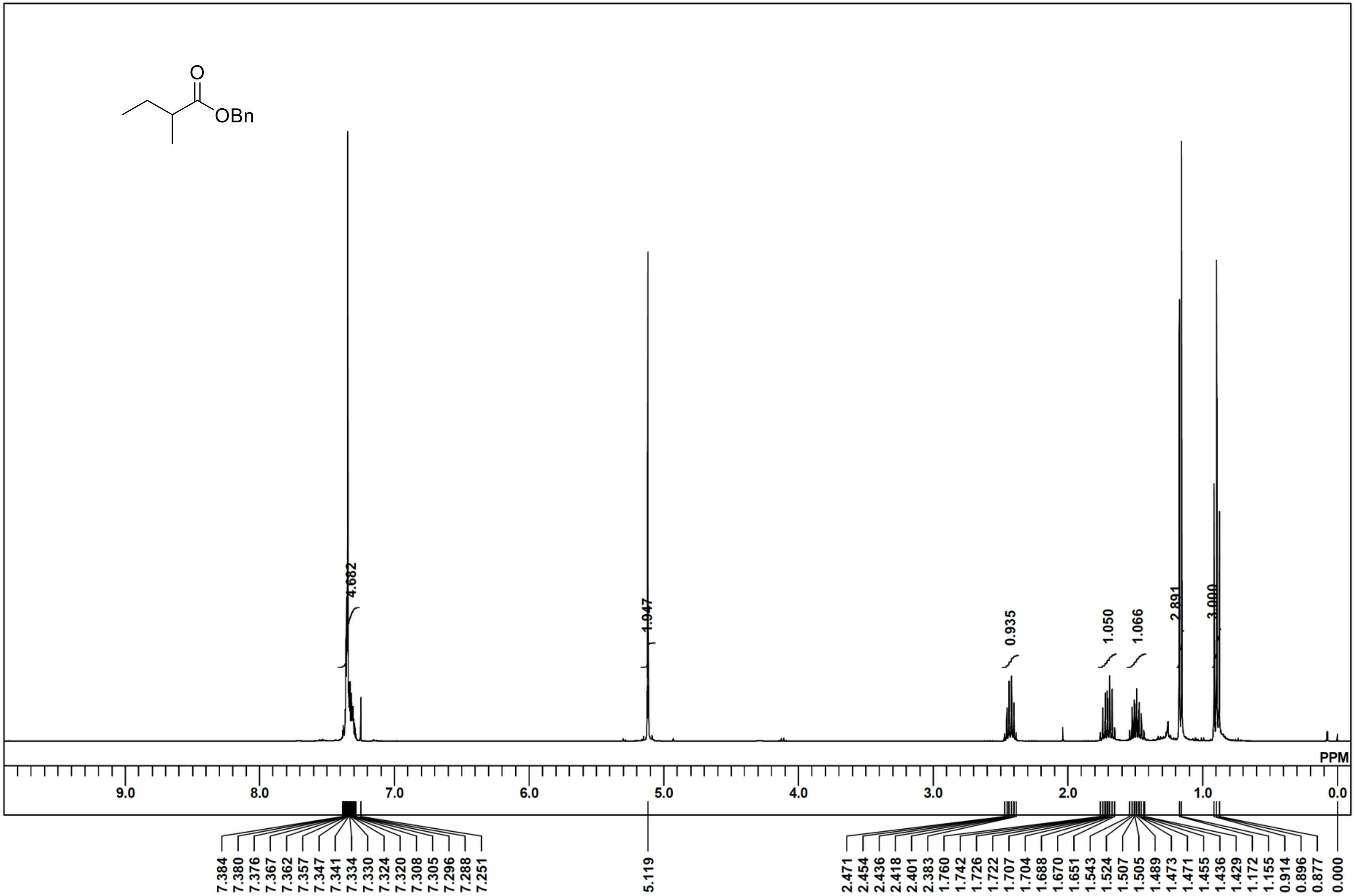

^{13}C NMR spectrum of **2q**

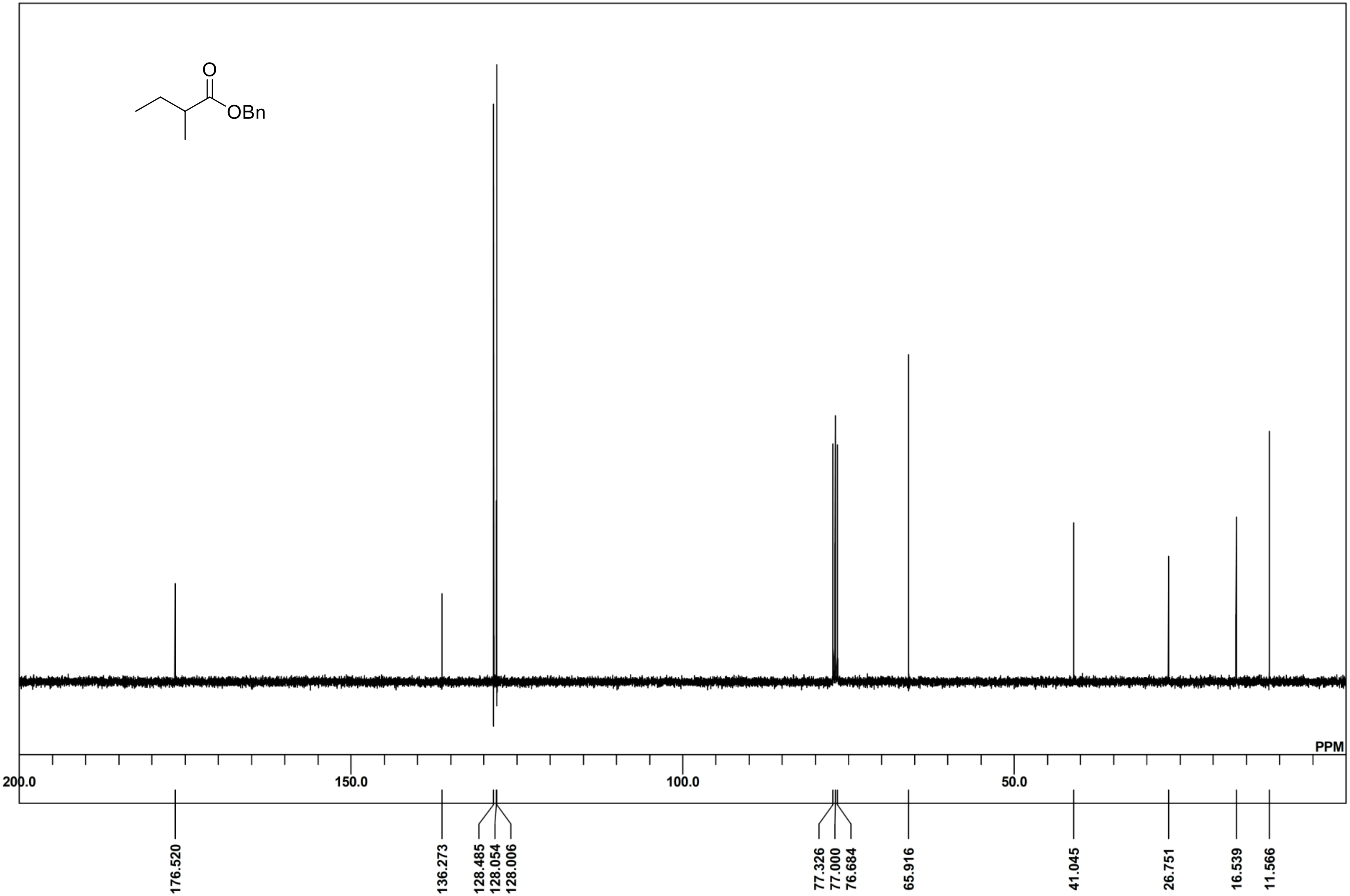

^{1}H NMR spectrum of **2t**

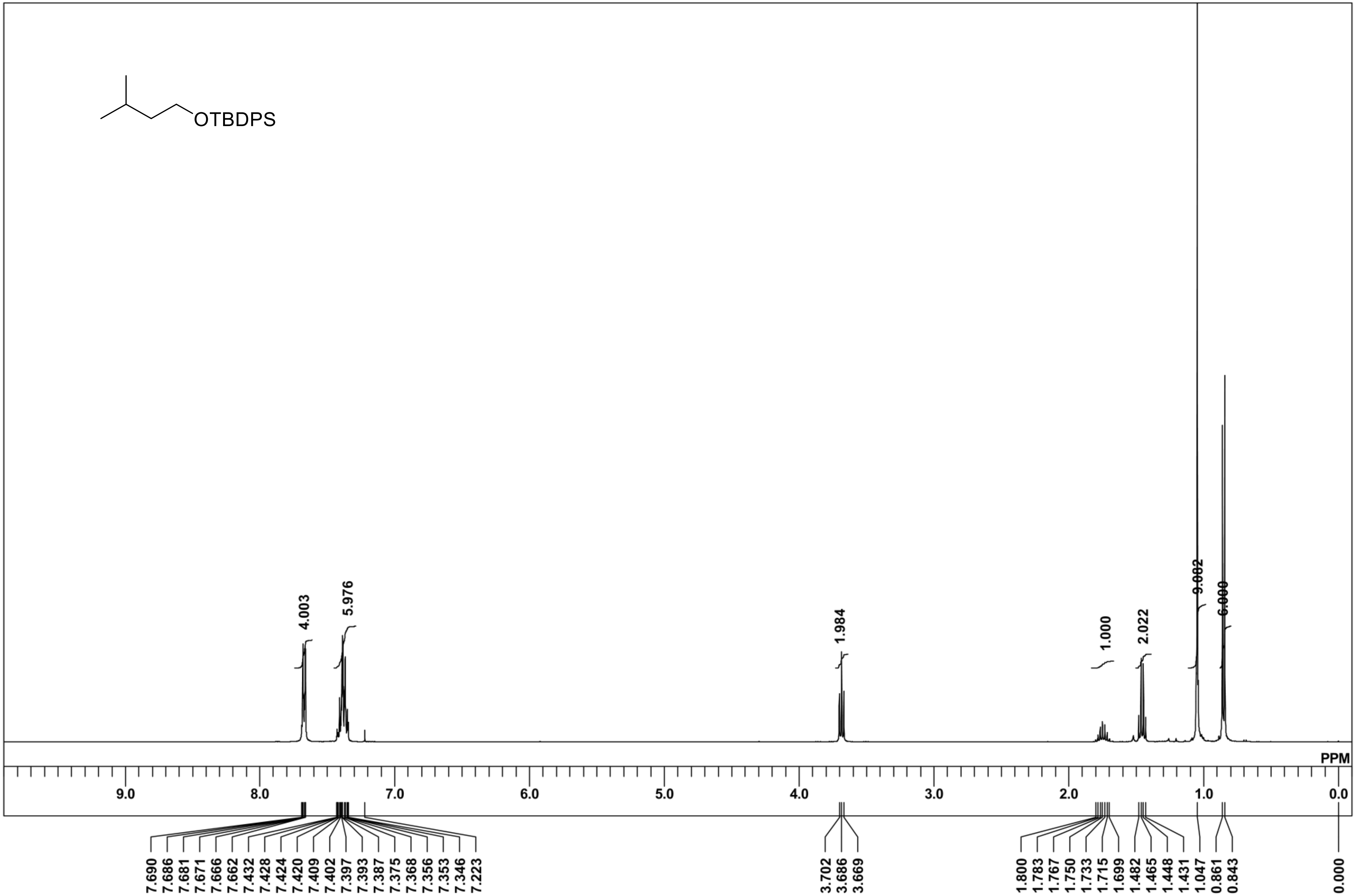

^{13}C NMR spectrum of **2t**

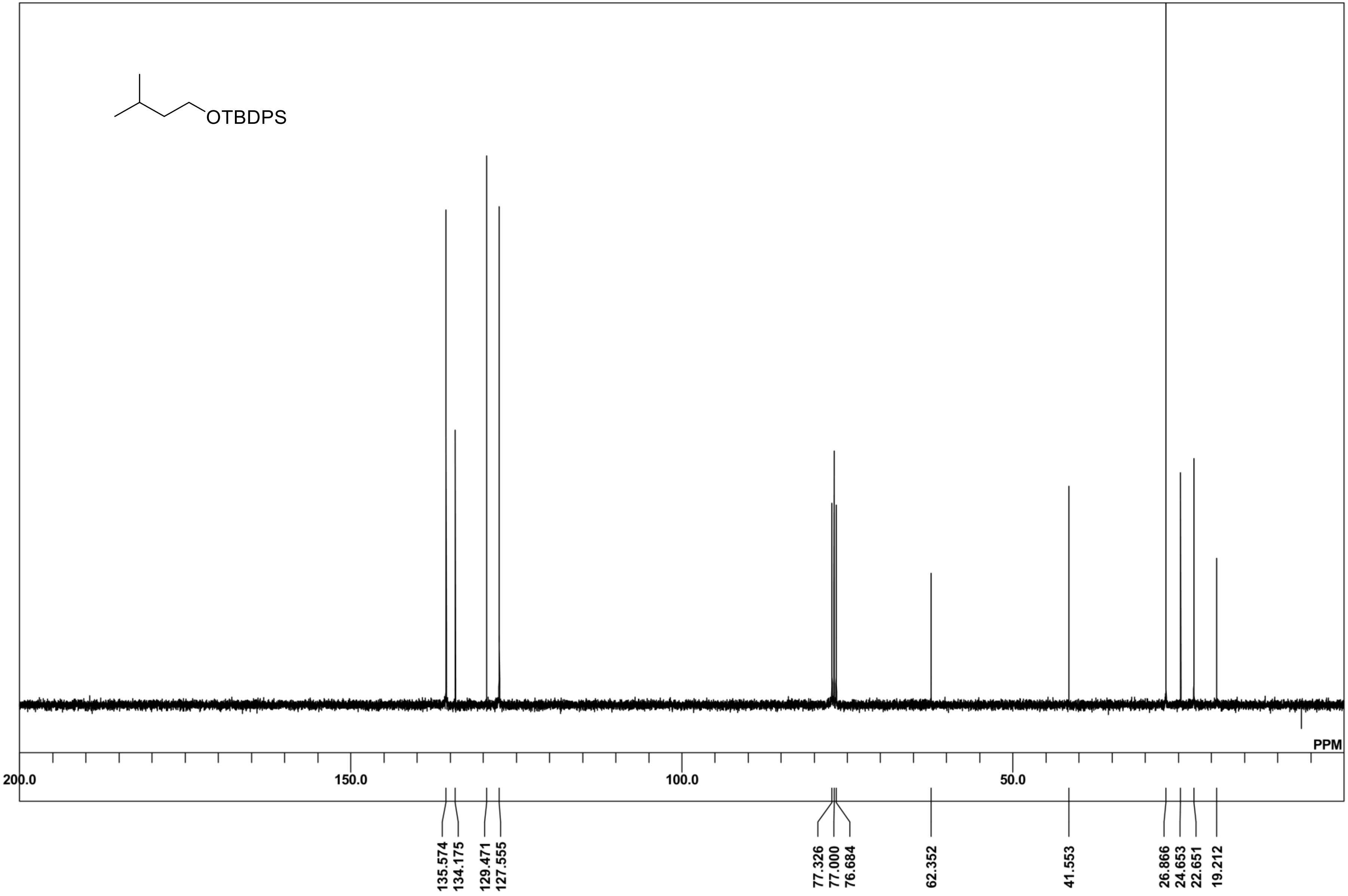

^{1}H NMR spectrum of **2u**

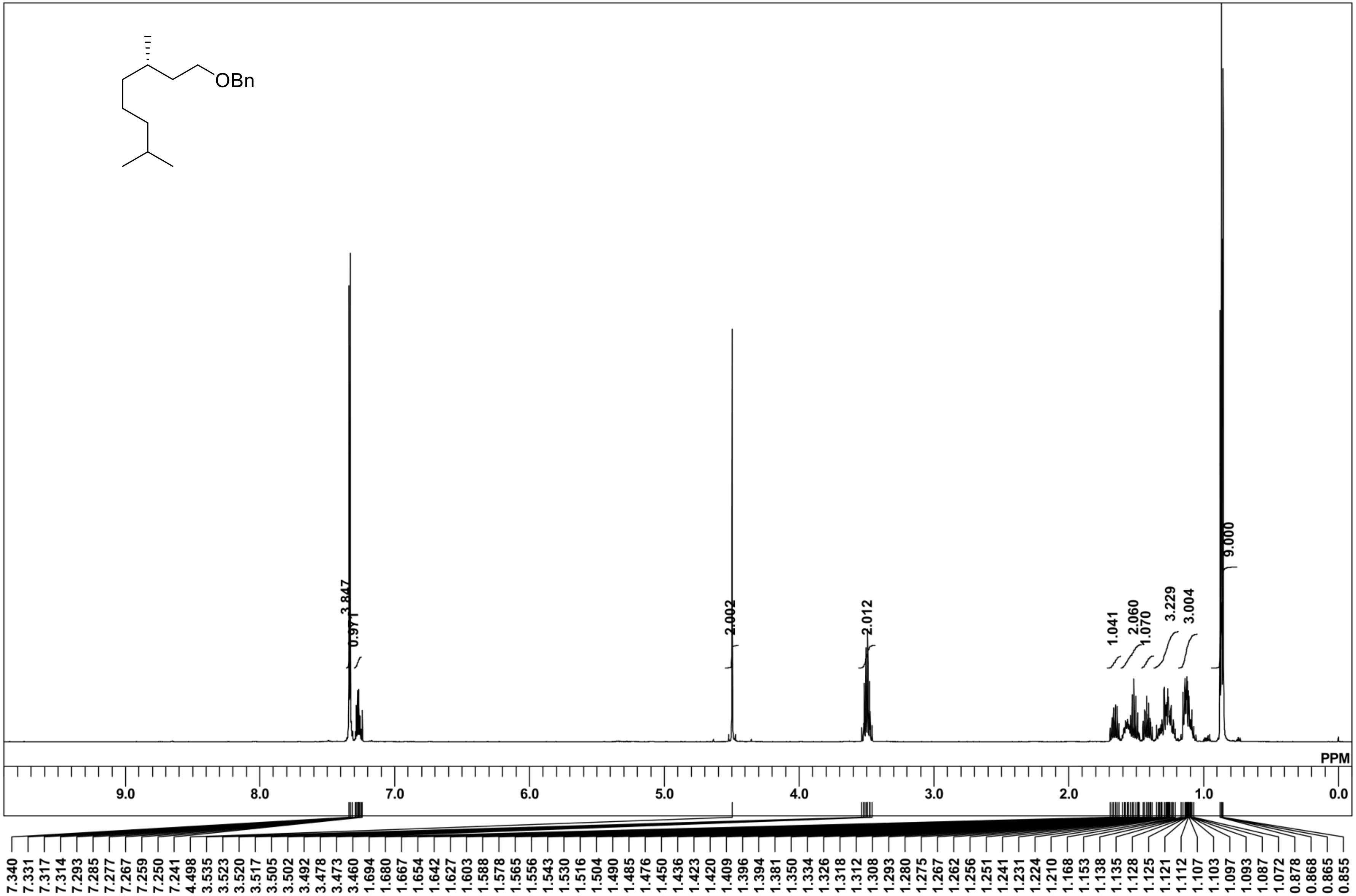

^{13}C NMR spectrum of **2u**

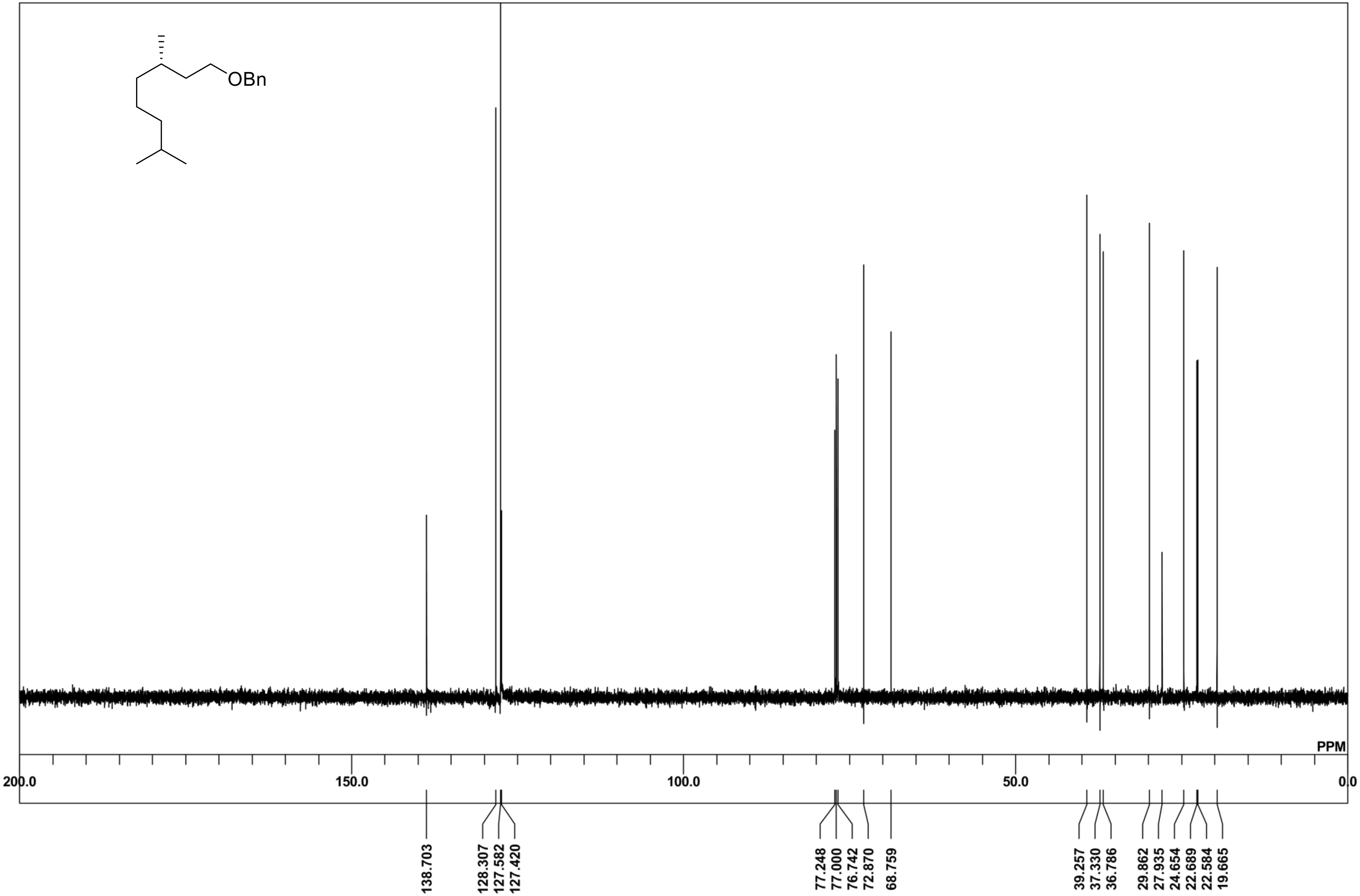

^{1}H NMR spectrum of **2v**

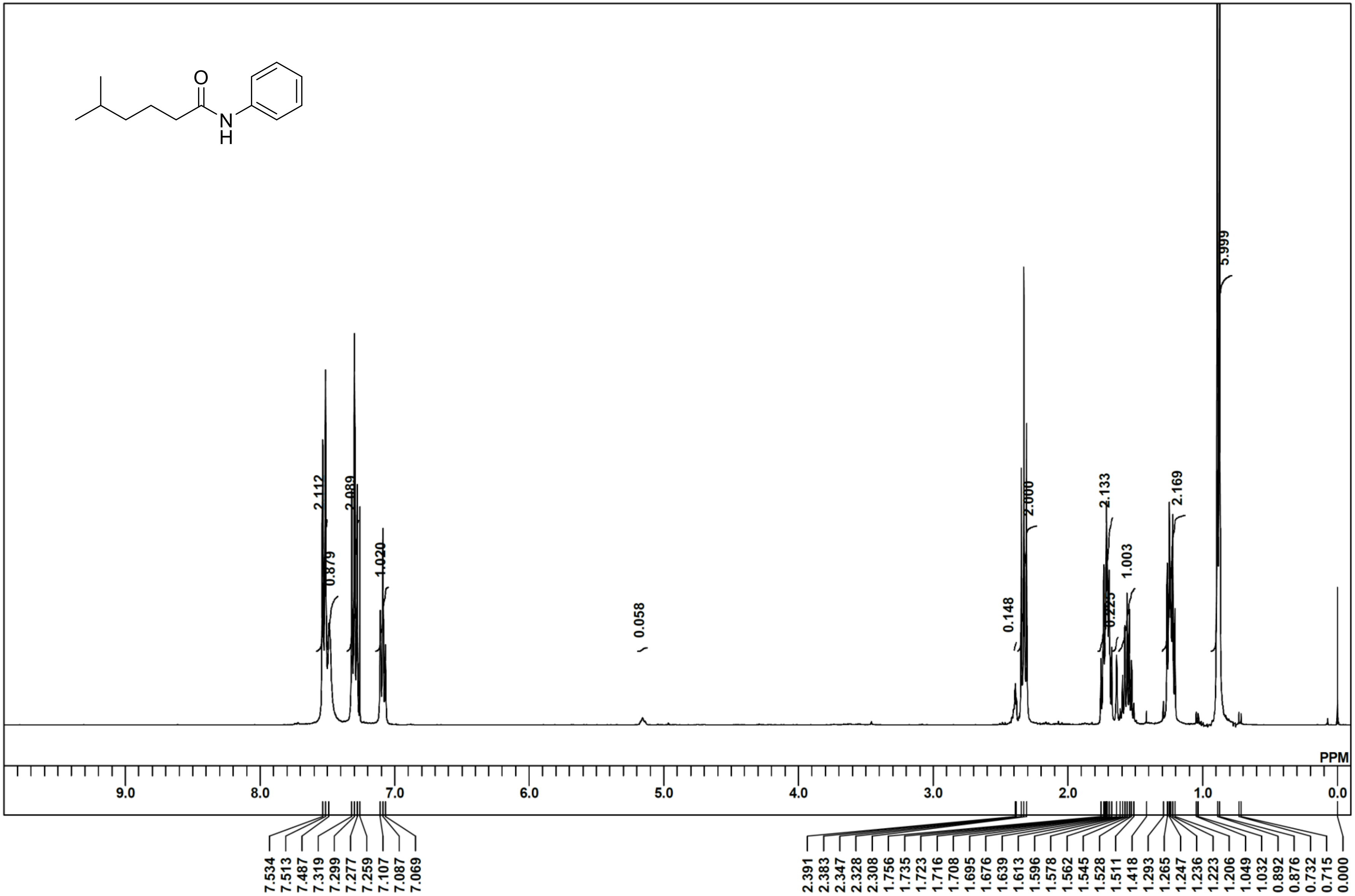

^{13}C NMR spectrum of **2v**

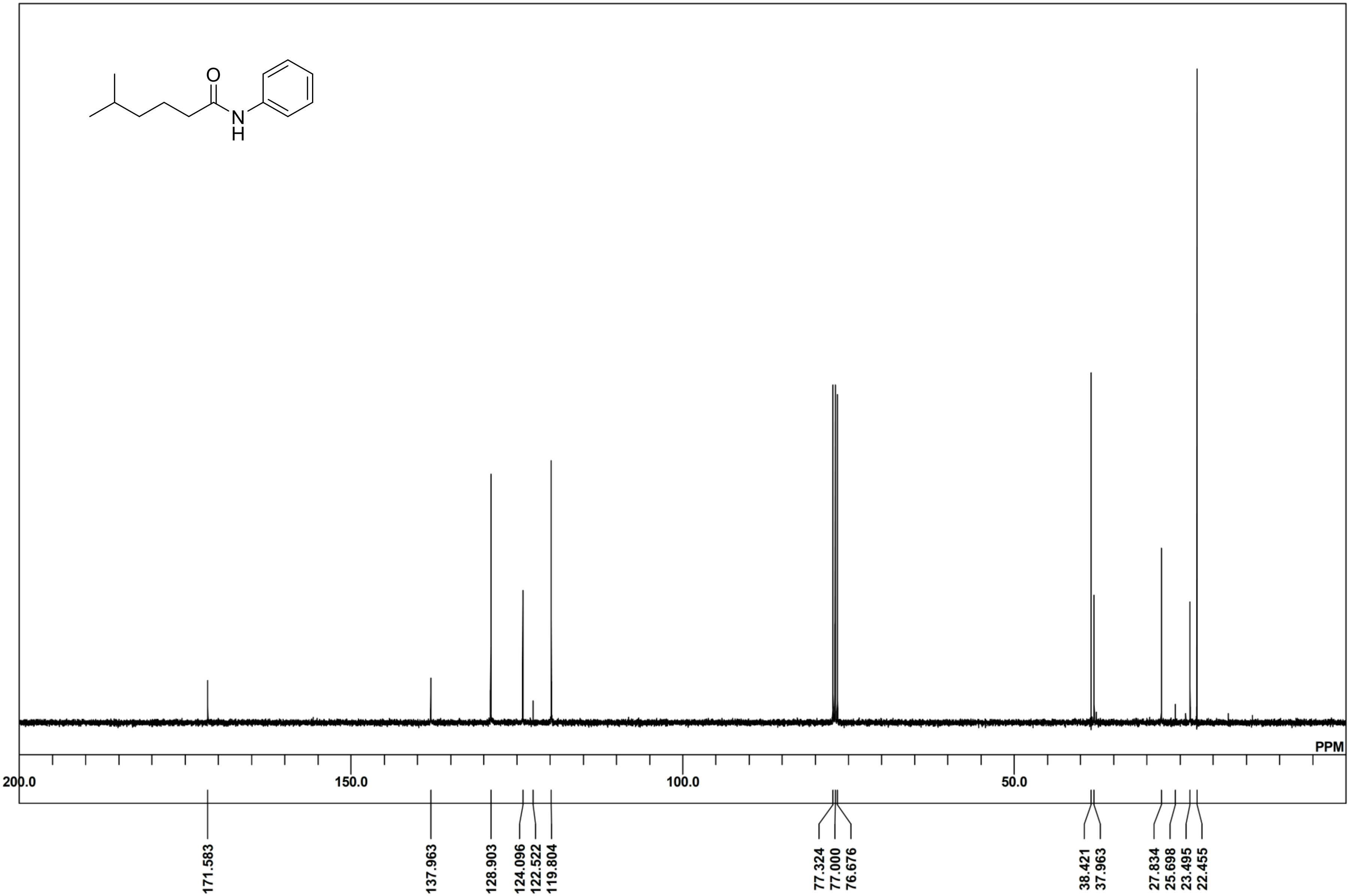

^{1}H NMR spectrum of **2w**

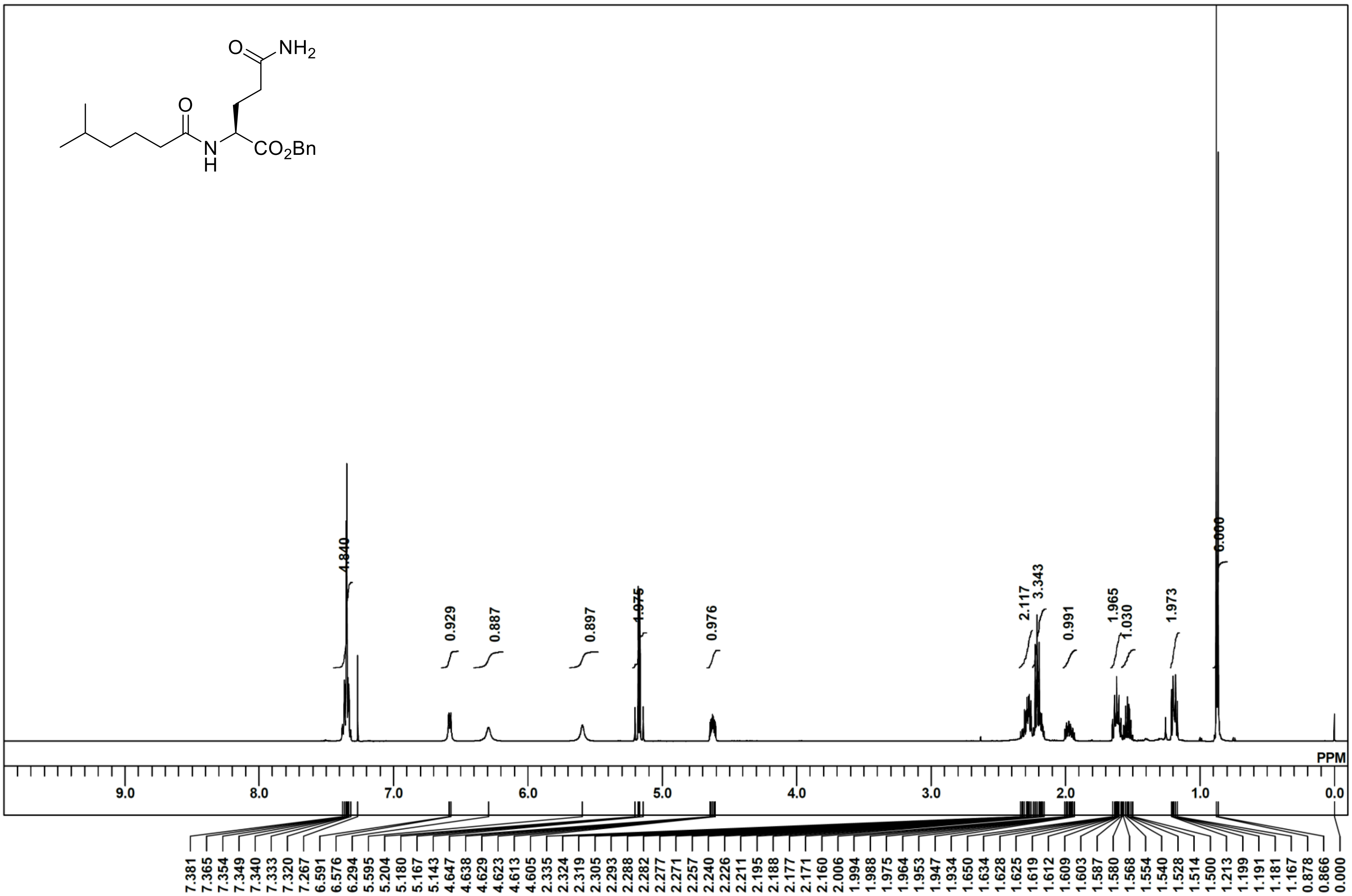

^{13}C NMR spectrum of **2w**

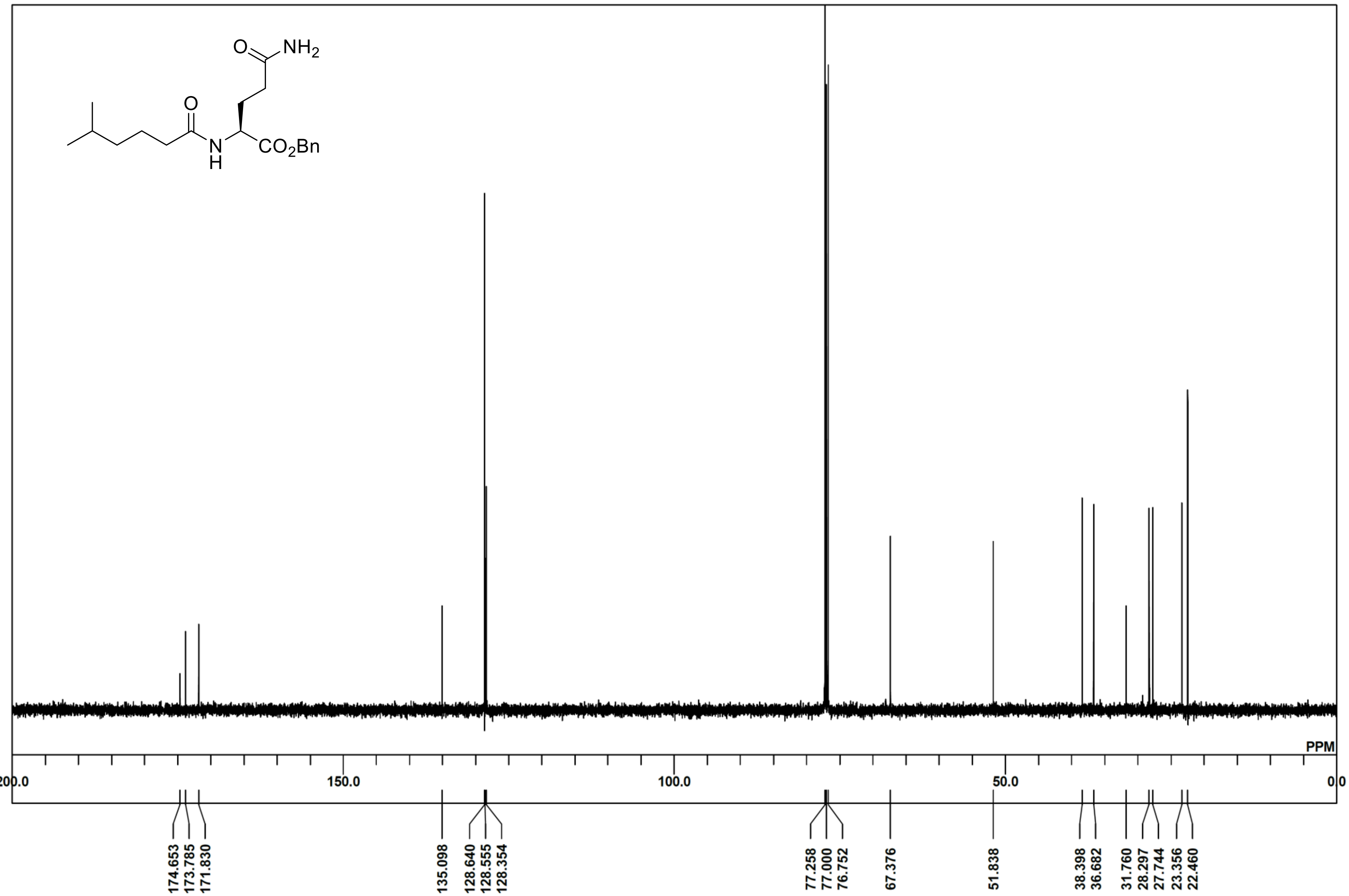

^{1}H NMR spectrum of **2x**

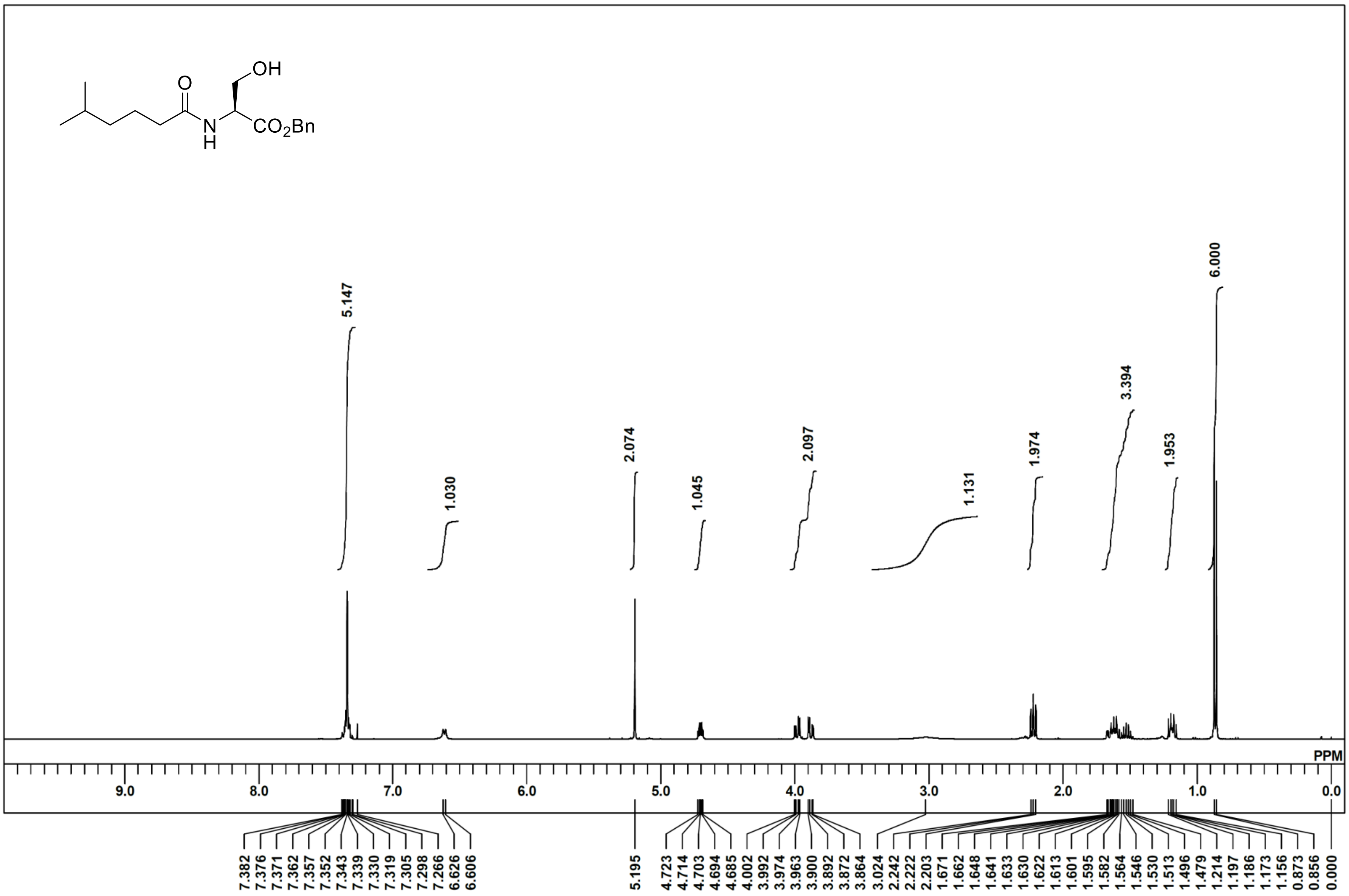

^{13}C NMR spectrum of **2x**

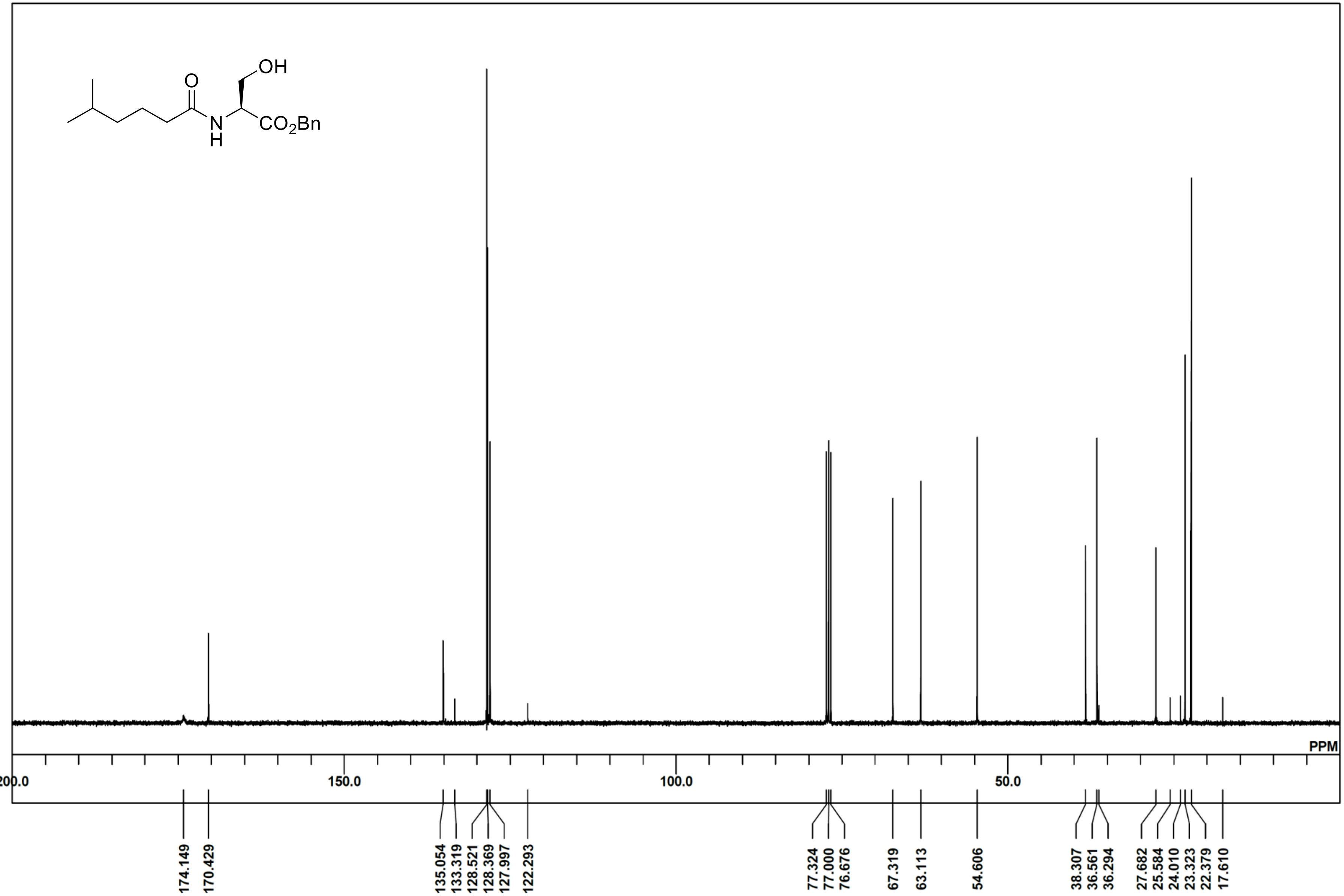

^{1}H NMR spectrum of **2y**

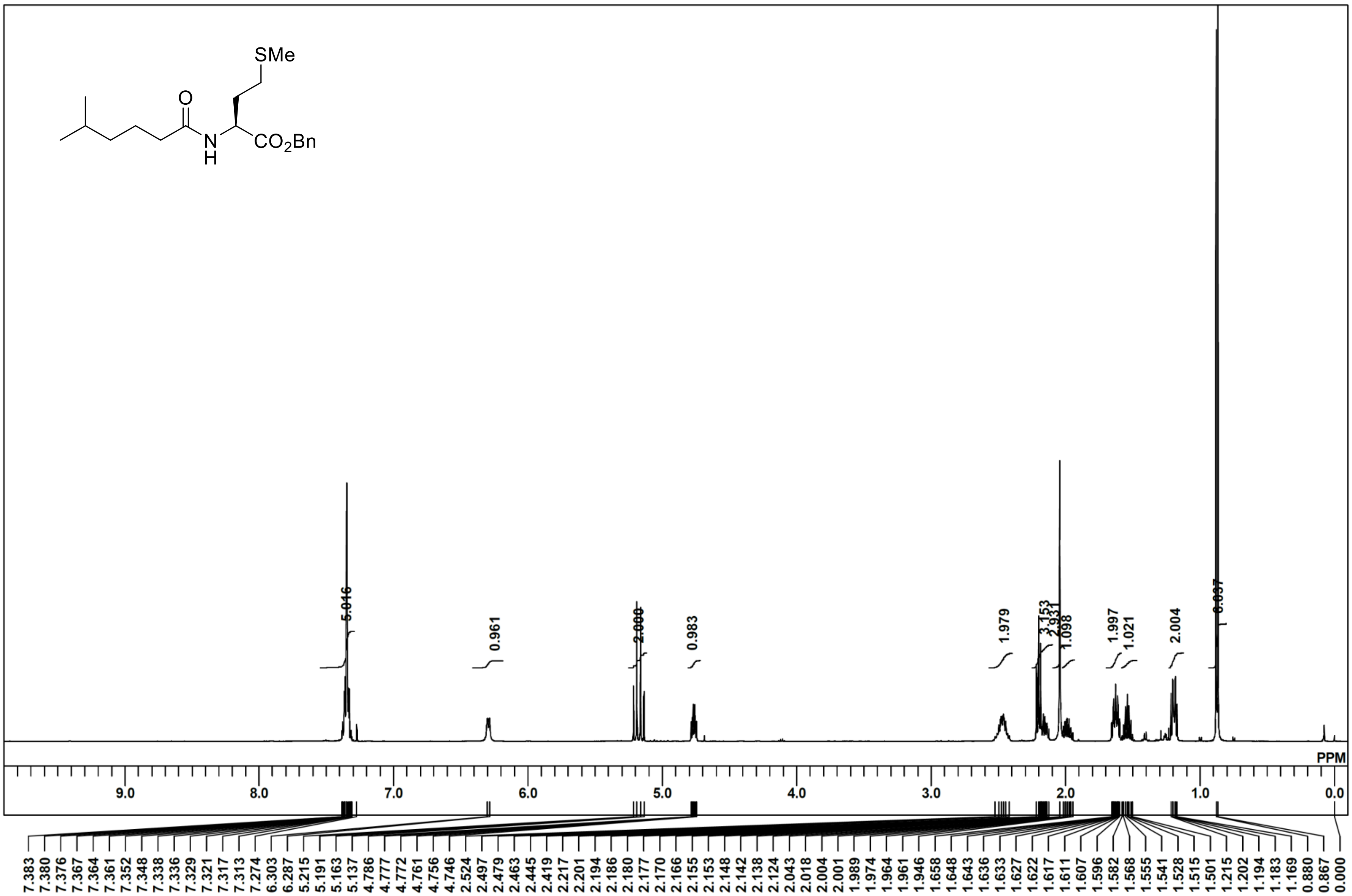

^{13}C NMR spectrum of **2y**

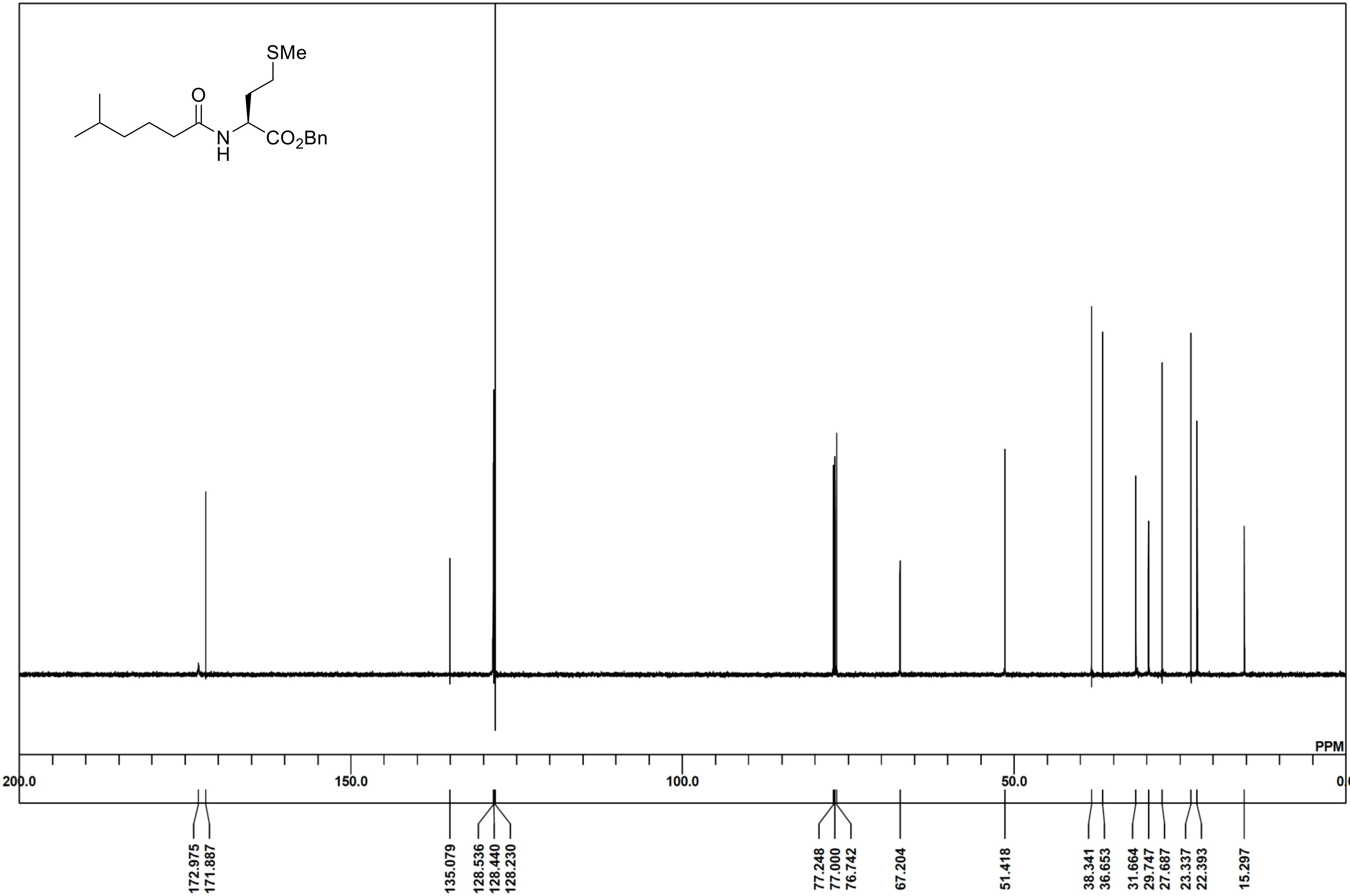

^{1}H NMR spectrum of **2z**

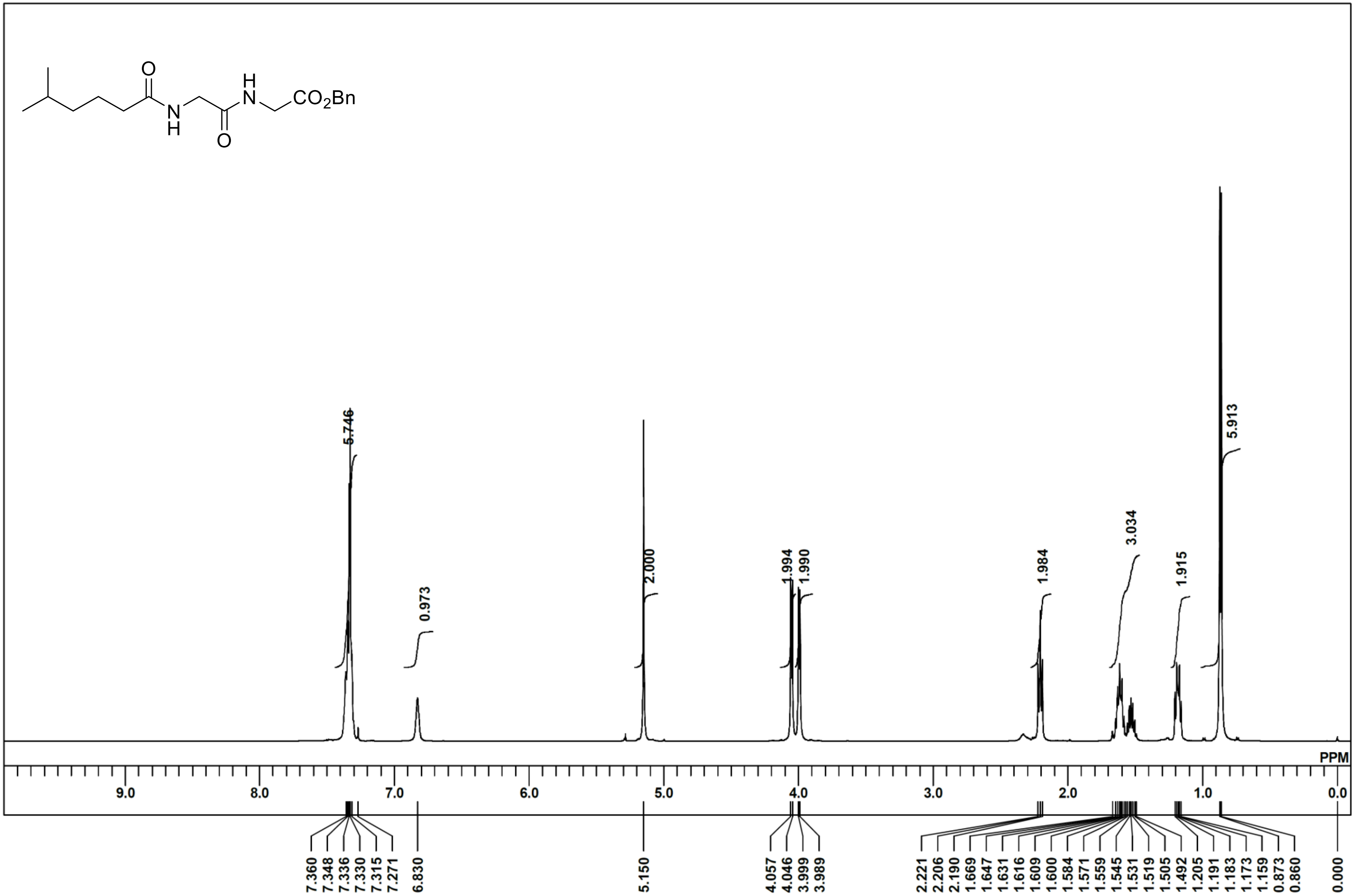

^{13}C NMR spectrum of **2z**

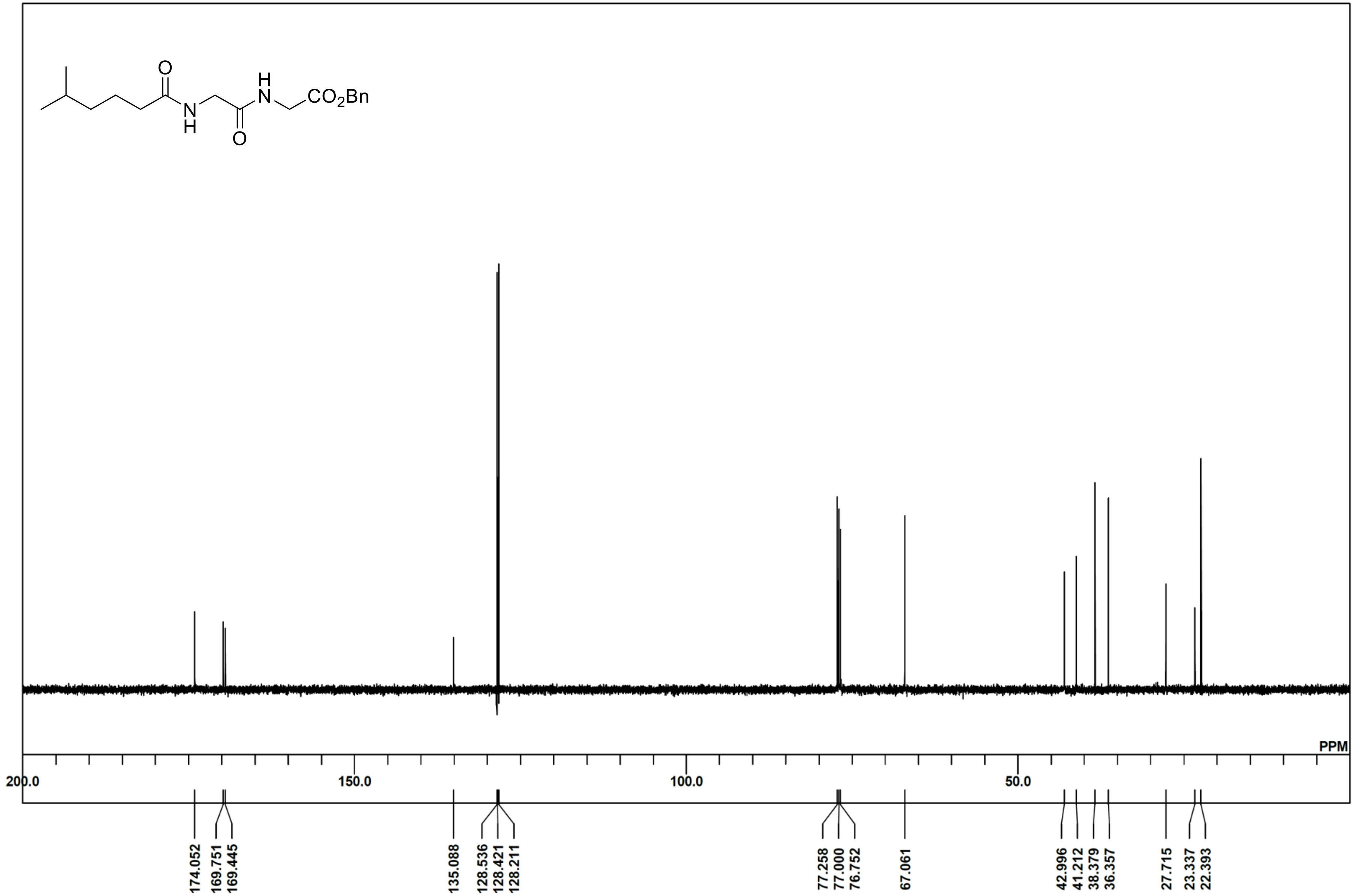

^{1}H NMR spectrum of **2aa**

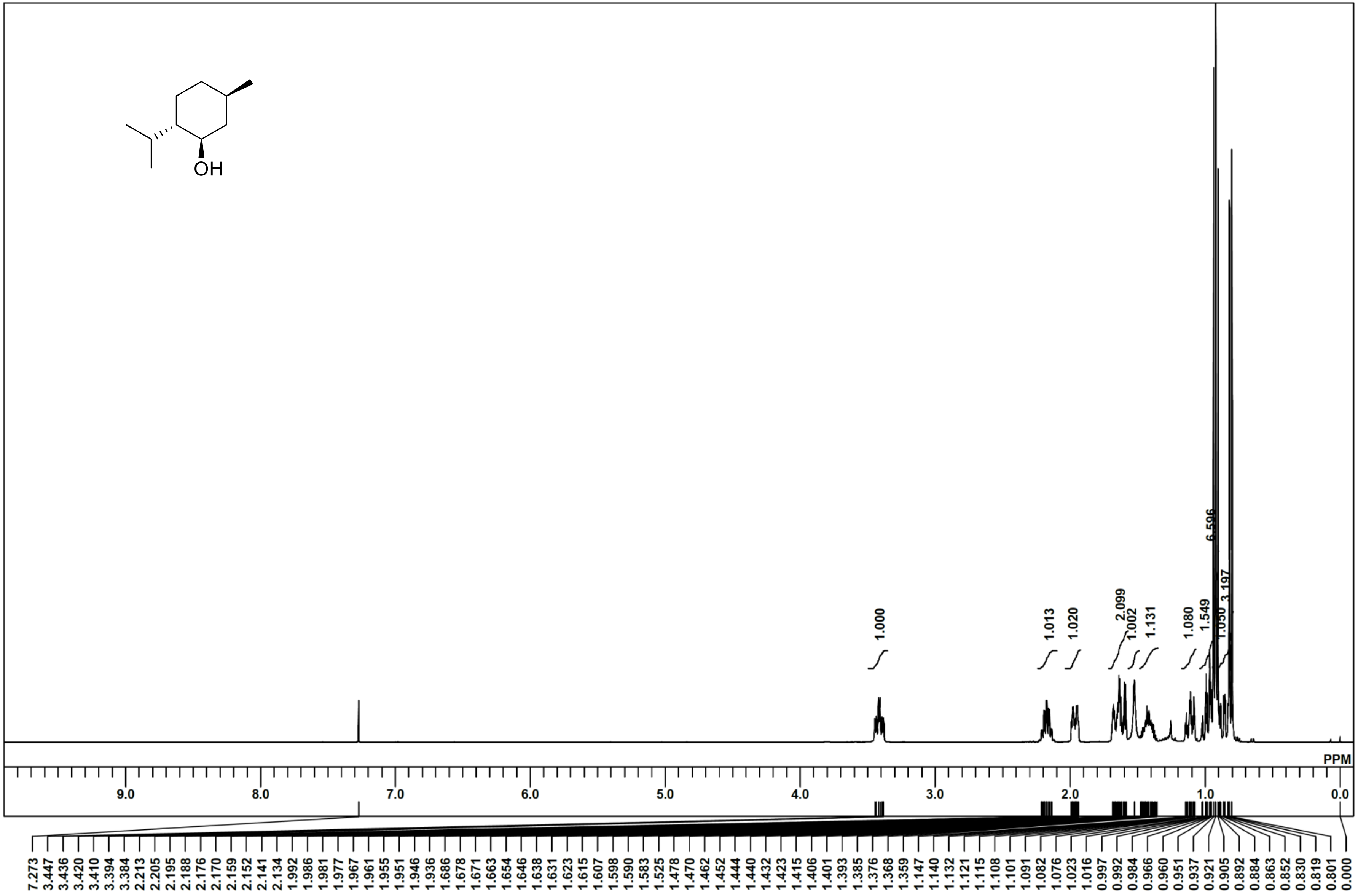

^{13}C NMR spectrum of **2aa**

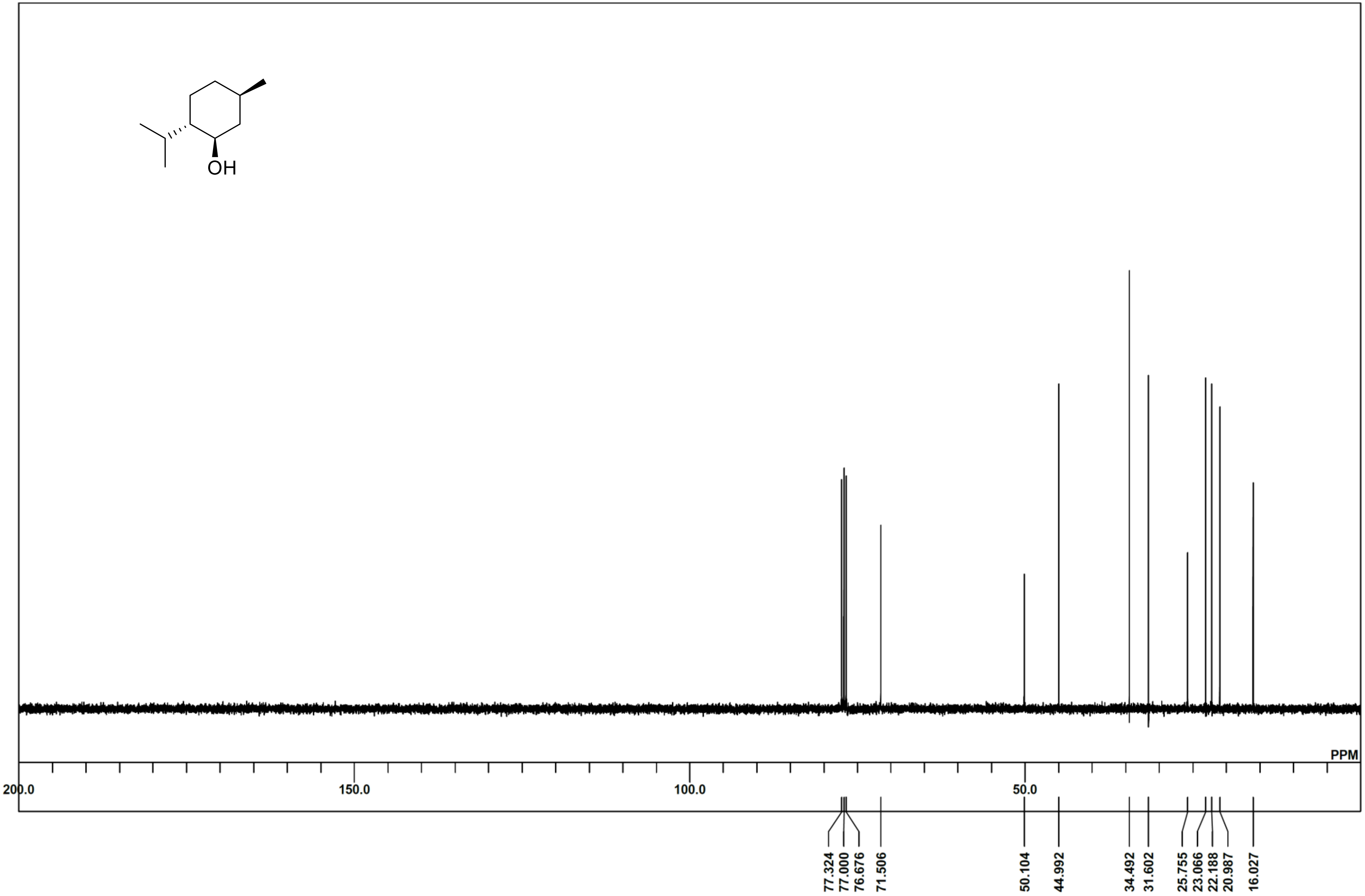

^{1}H NMR spectrum of **2ab**

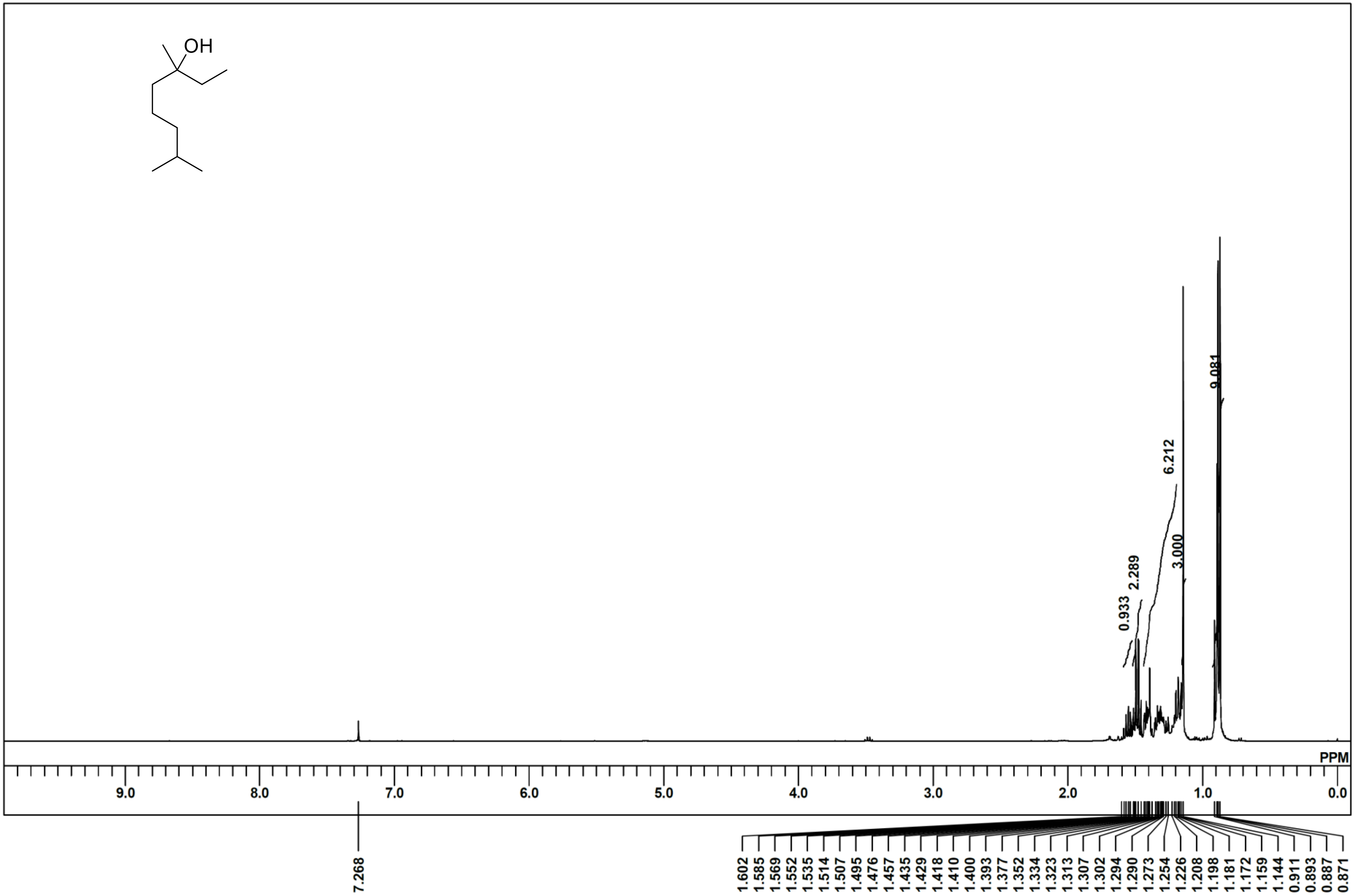

^{13}C NMR spectrum of **2ab**

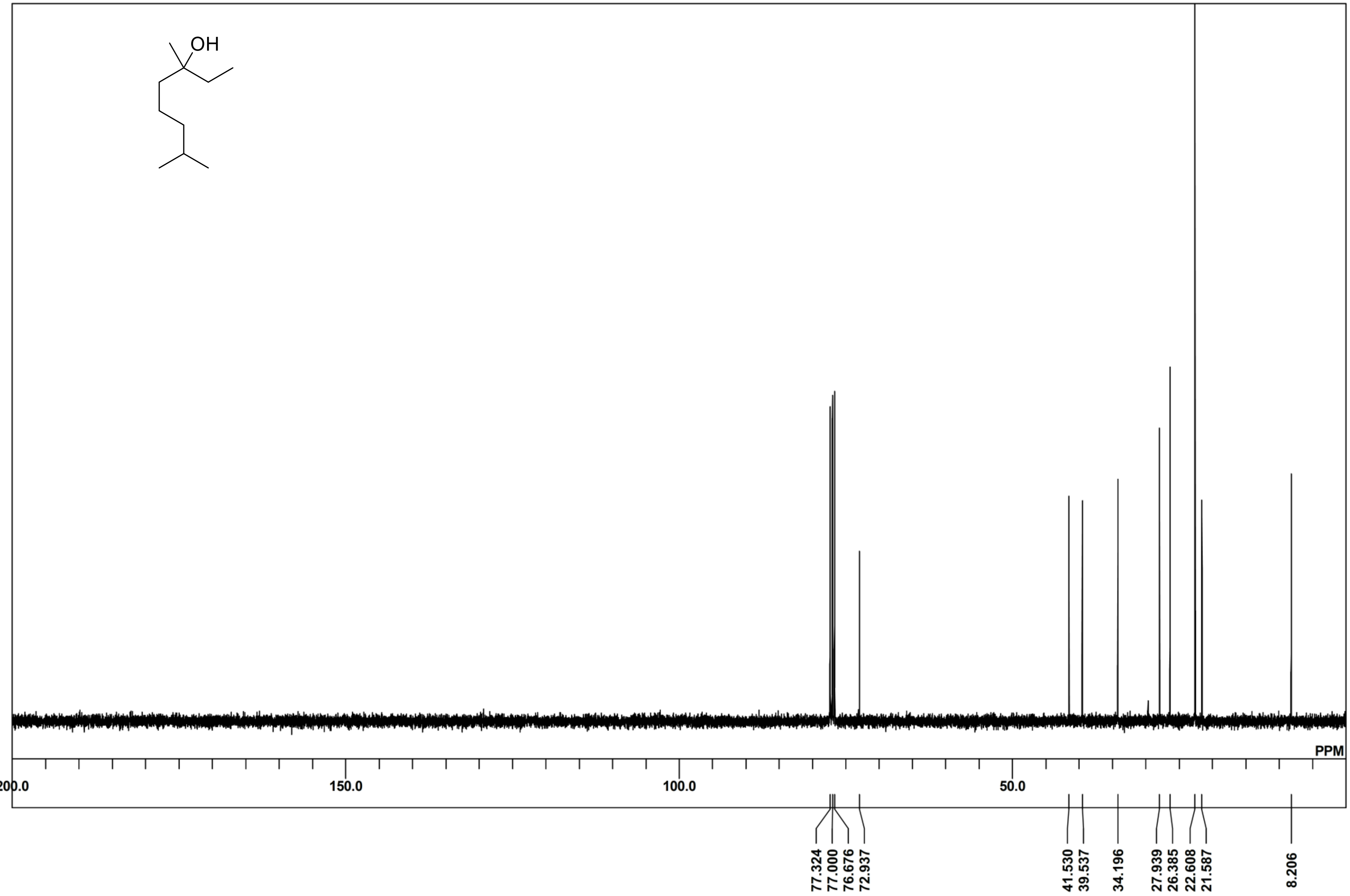

^{1}H NMR spectrum of **2ac**

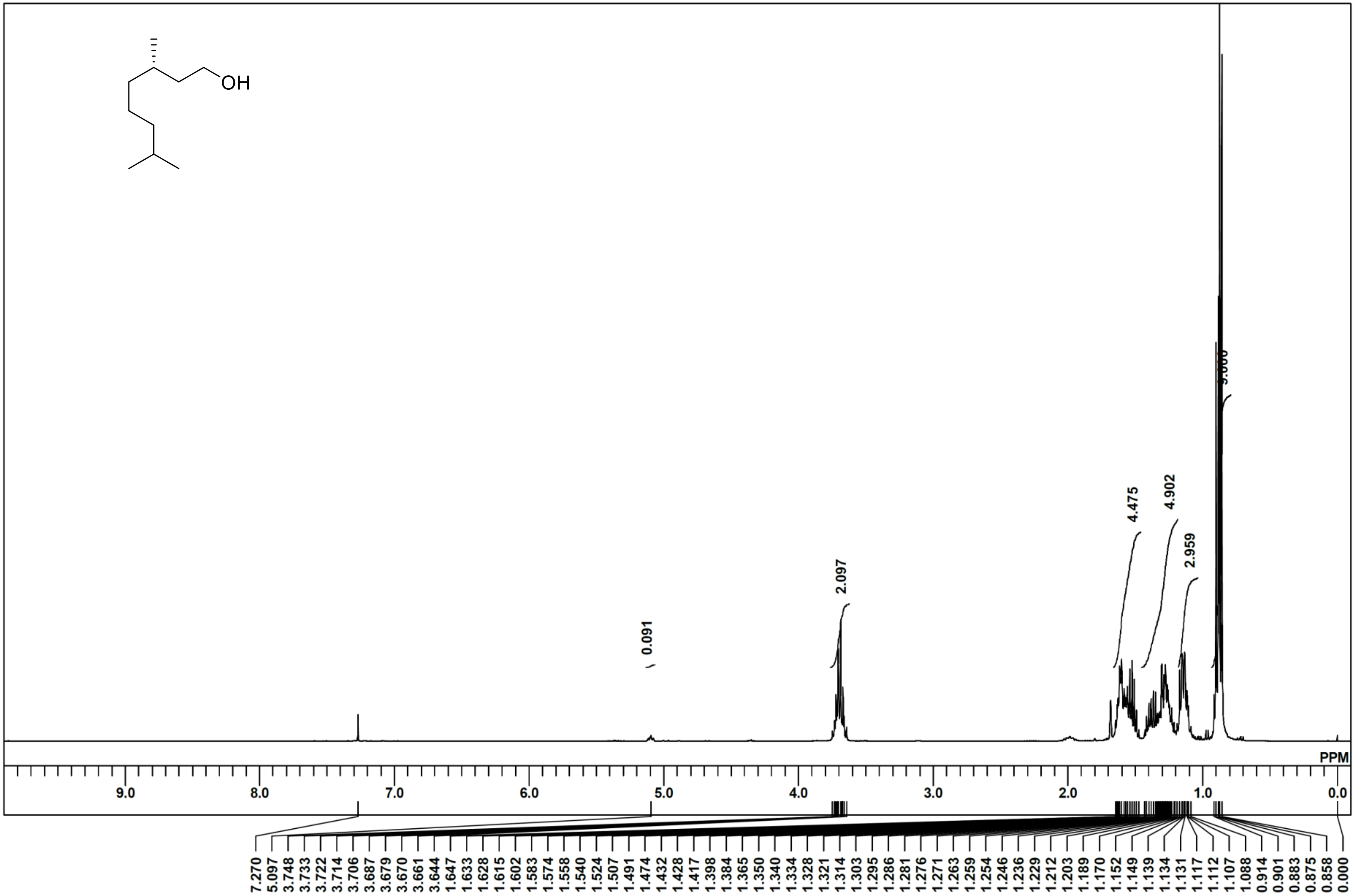

^{13}C NMR spectrum of **2ac**

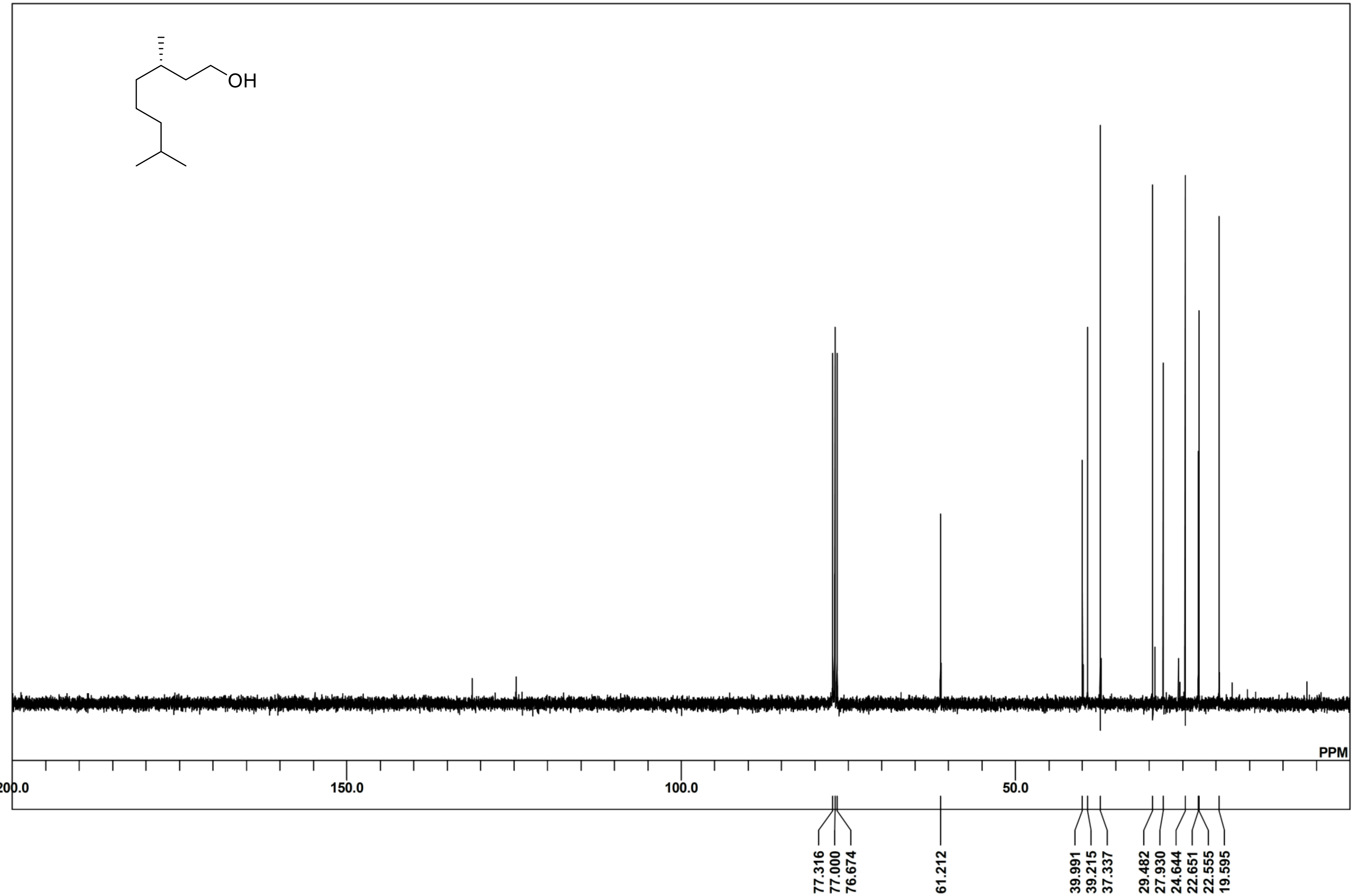

^{1}H NMR spectrum of **2ad’**

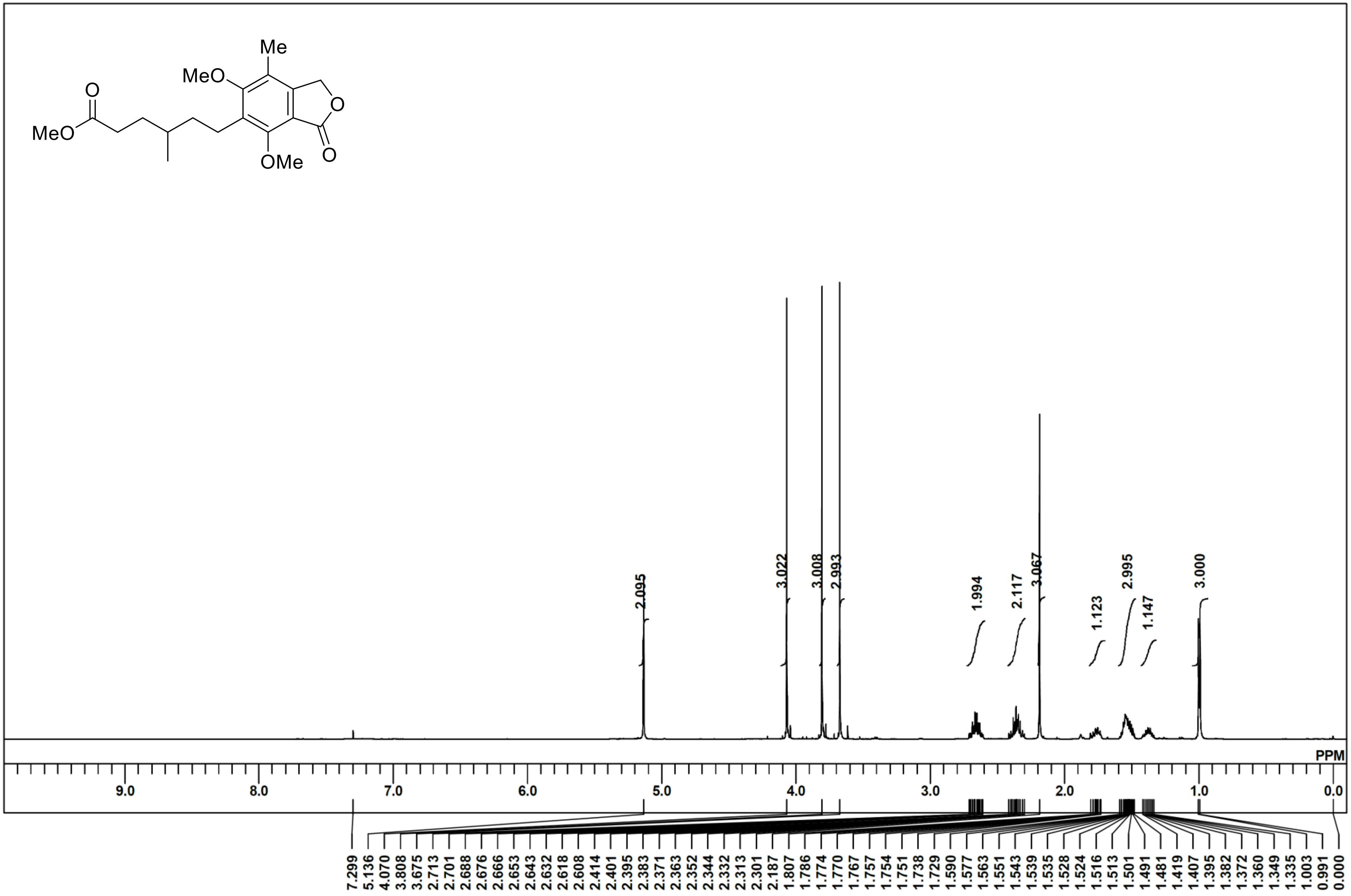

^{13}C NMR spectrum of **2ad’**

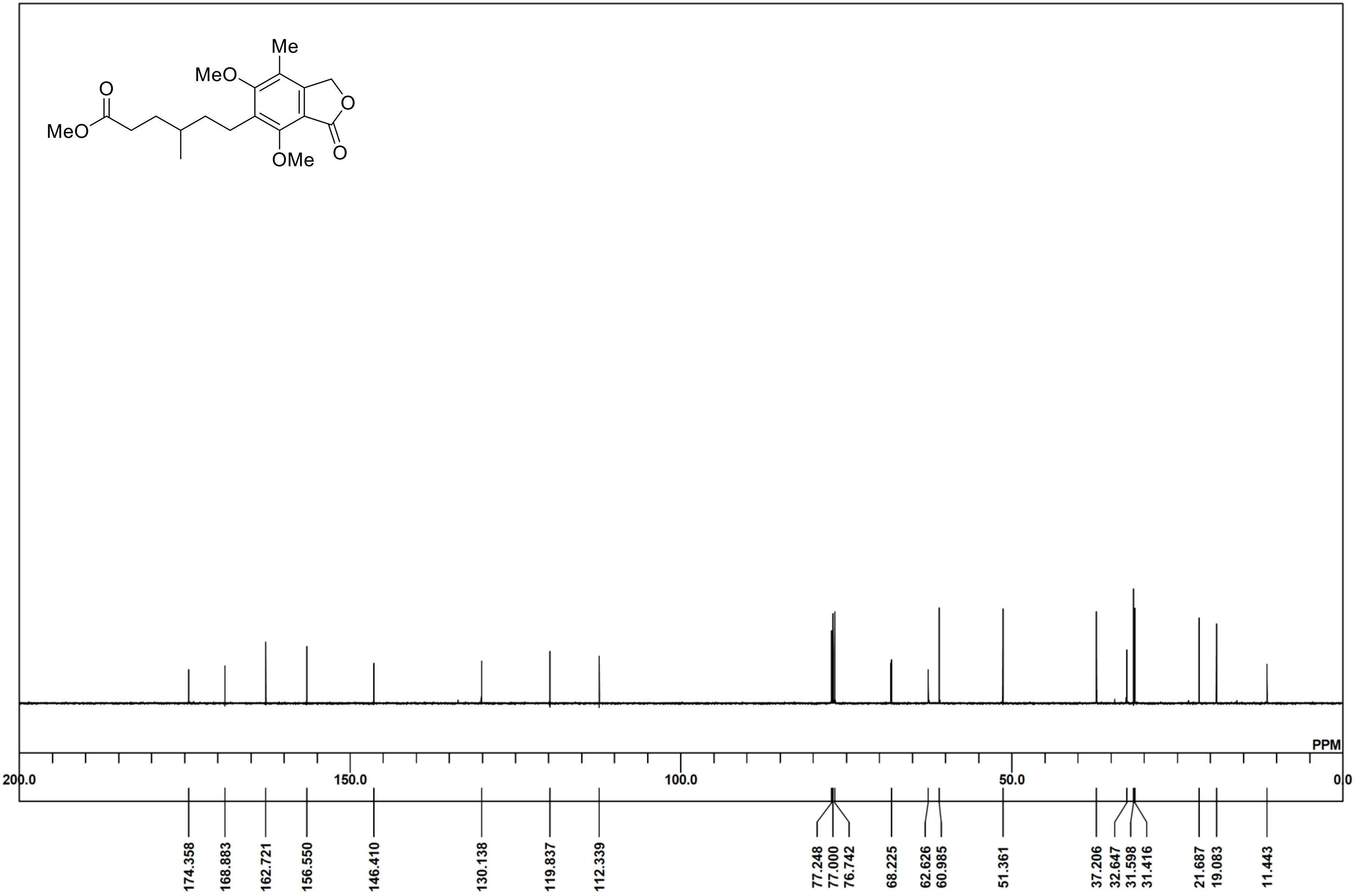

^{1}H NMR spectrum of **2ae'**

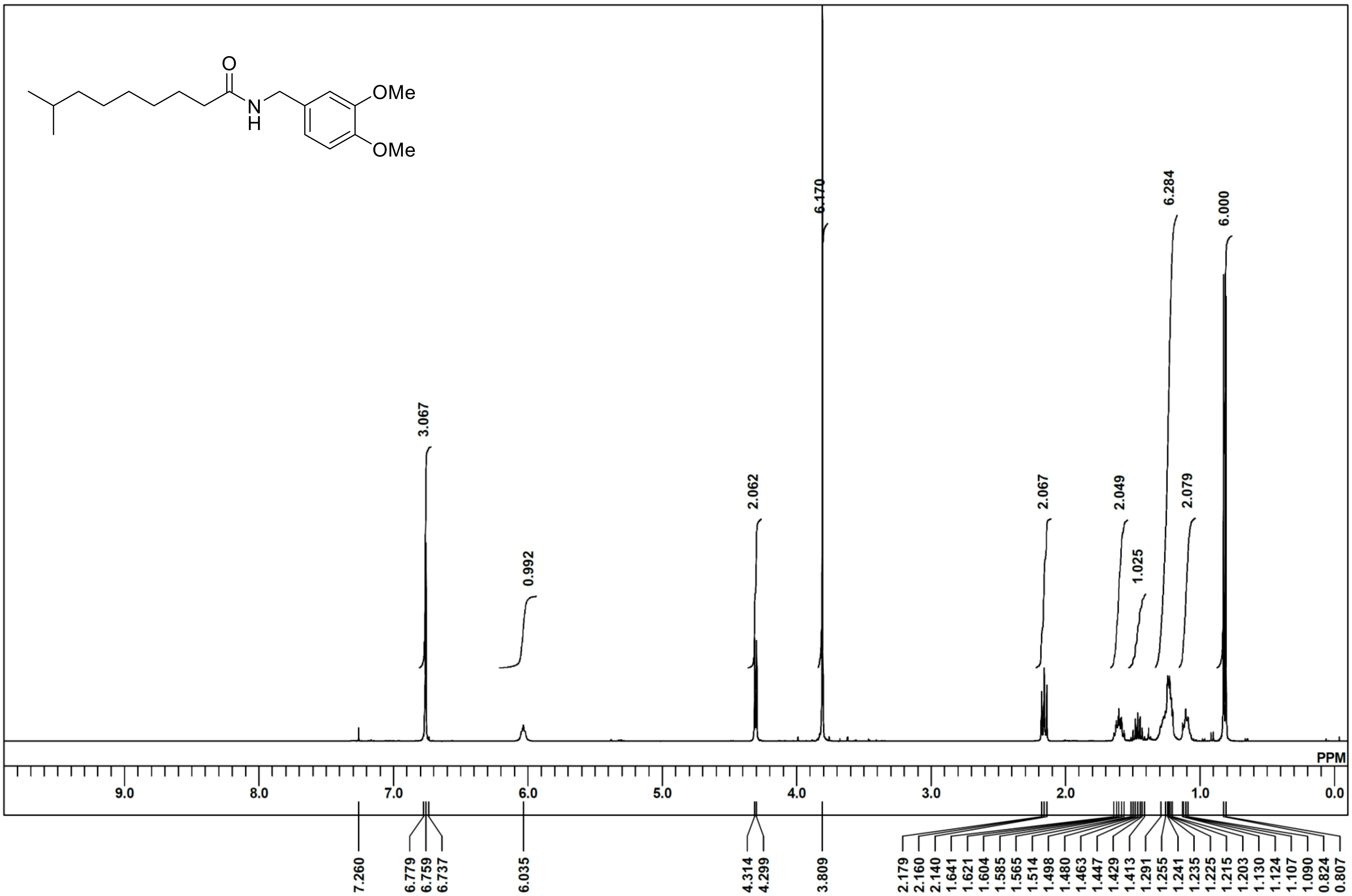

^{13}C NMR spectrum of **2ae’**

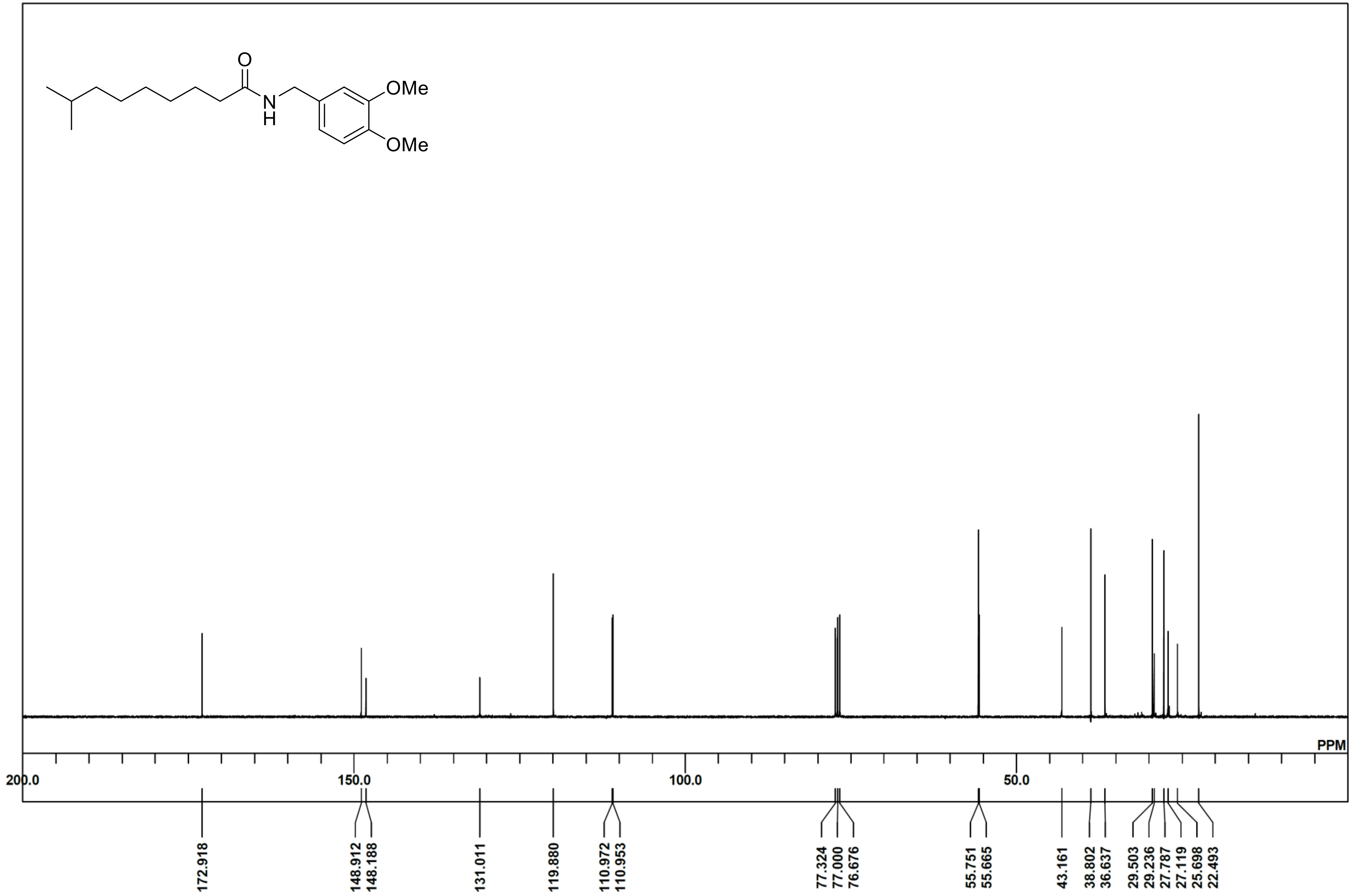

^{1}H NMR spectrum of **2af**

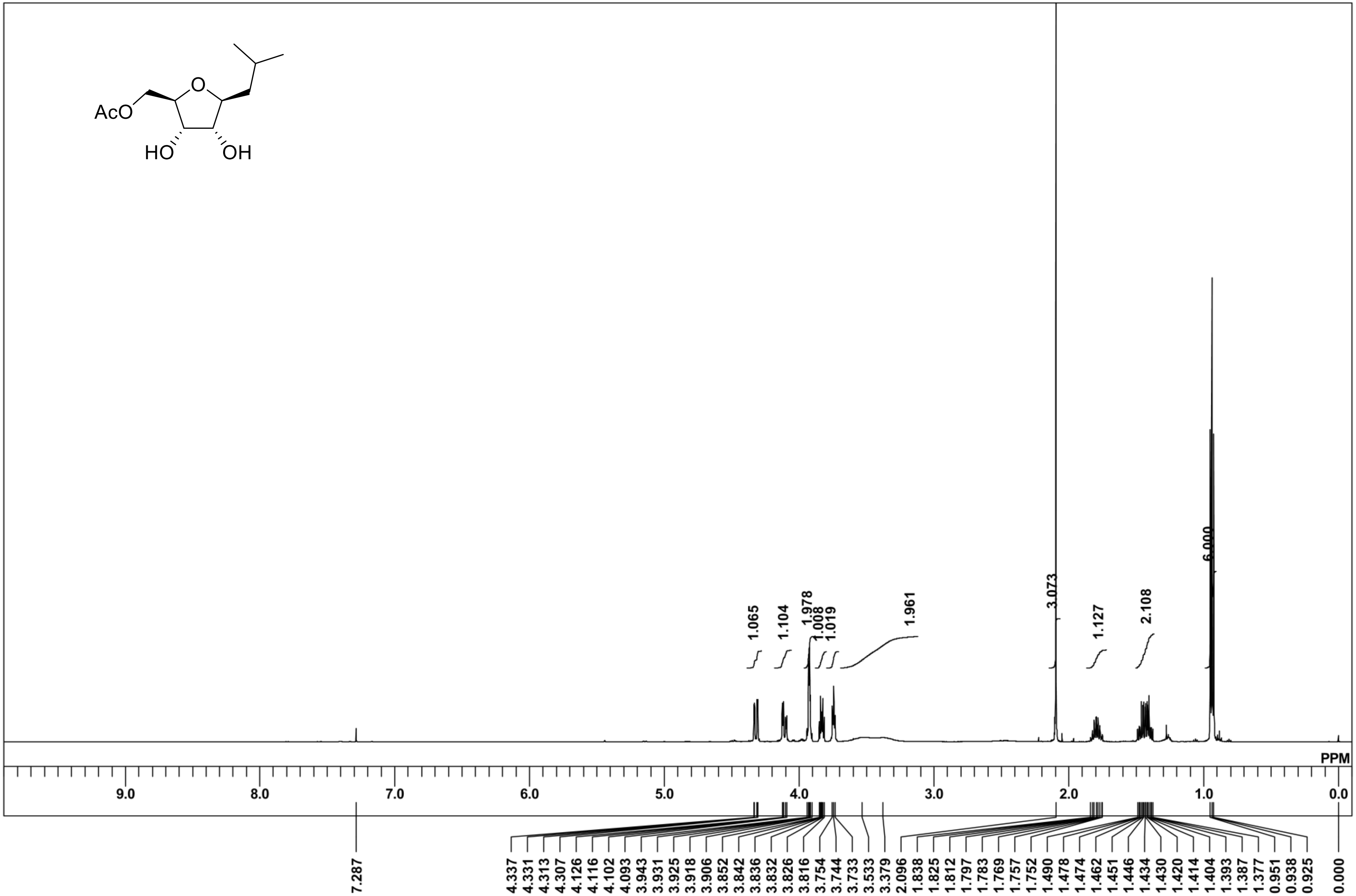

^{13}C NMR spectrum of **2af**

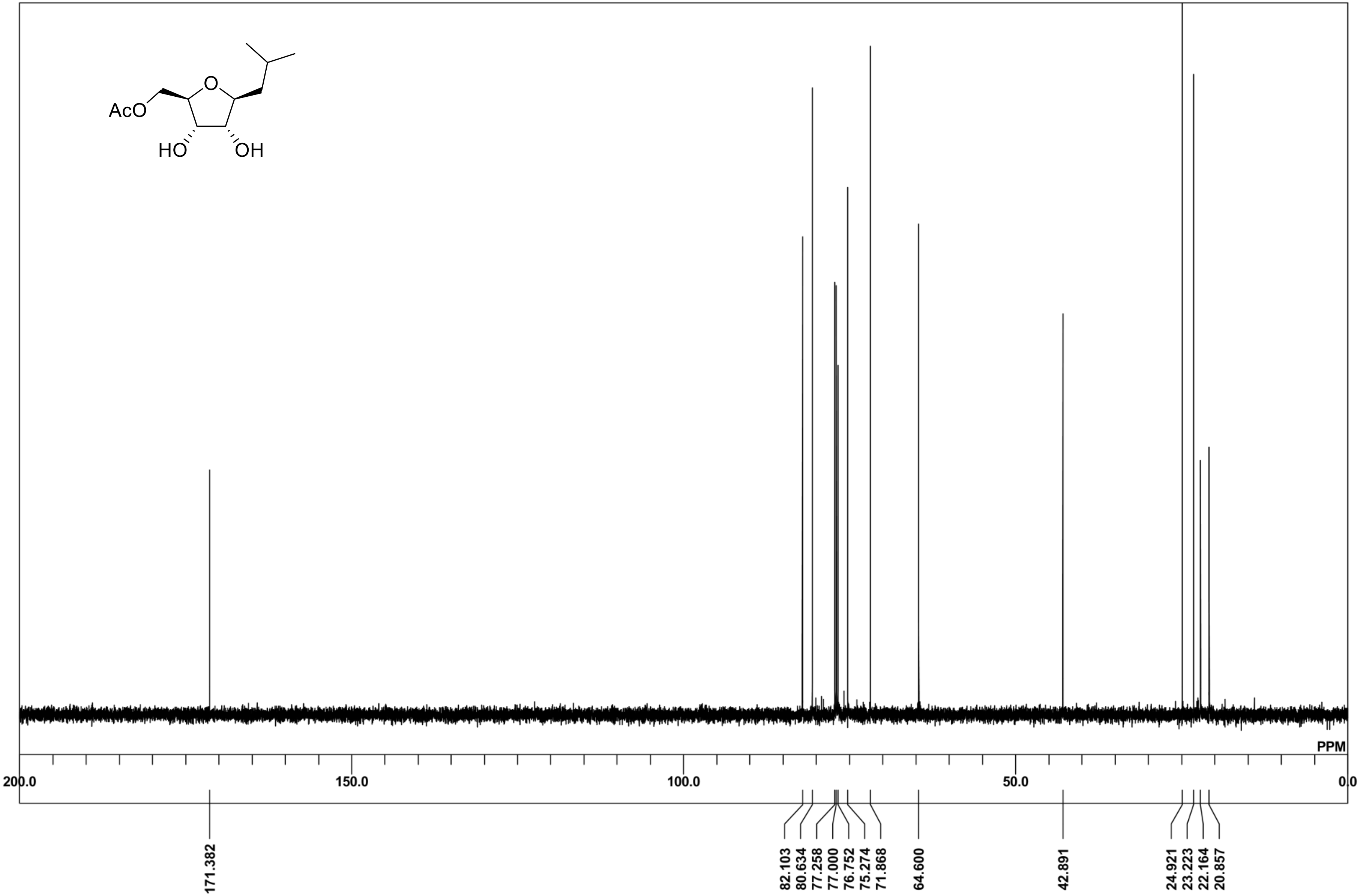

^{1}H NMR spectrum of **2ag**

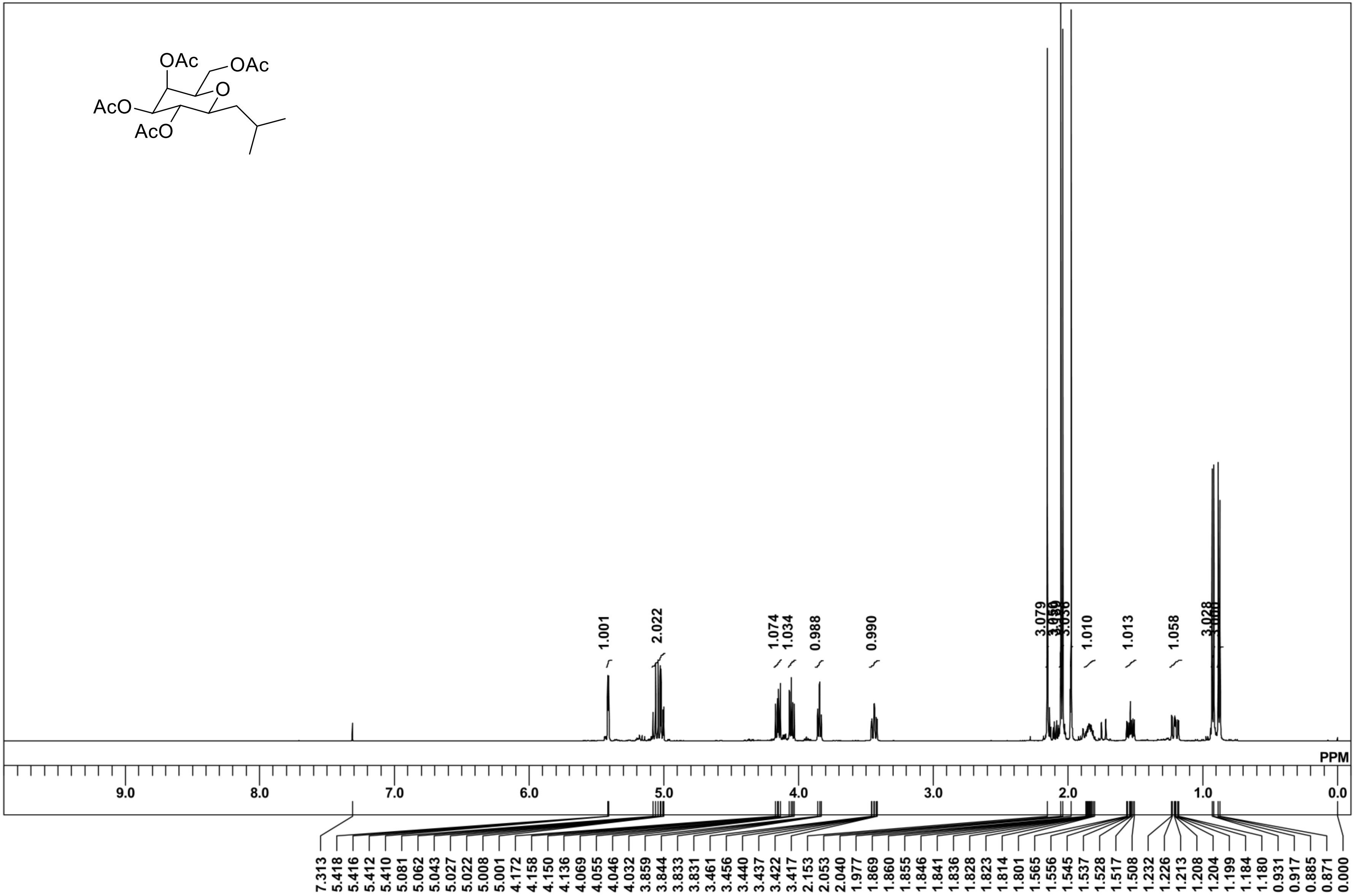

^{13}C NMR spectrum of **2ag**

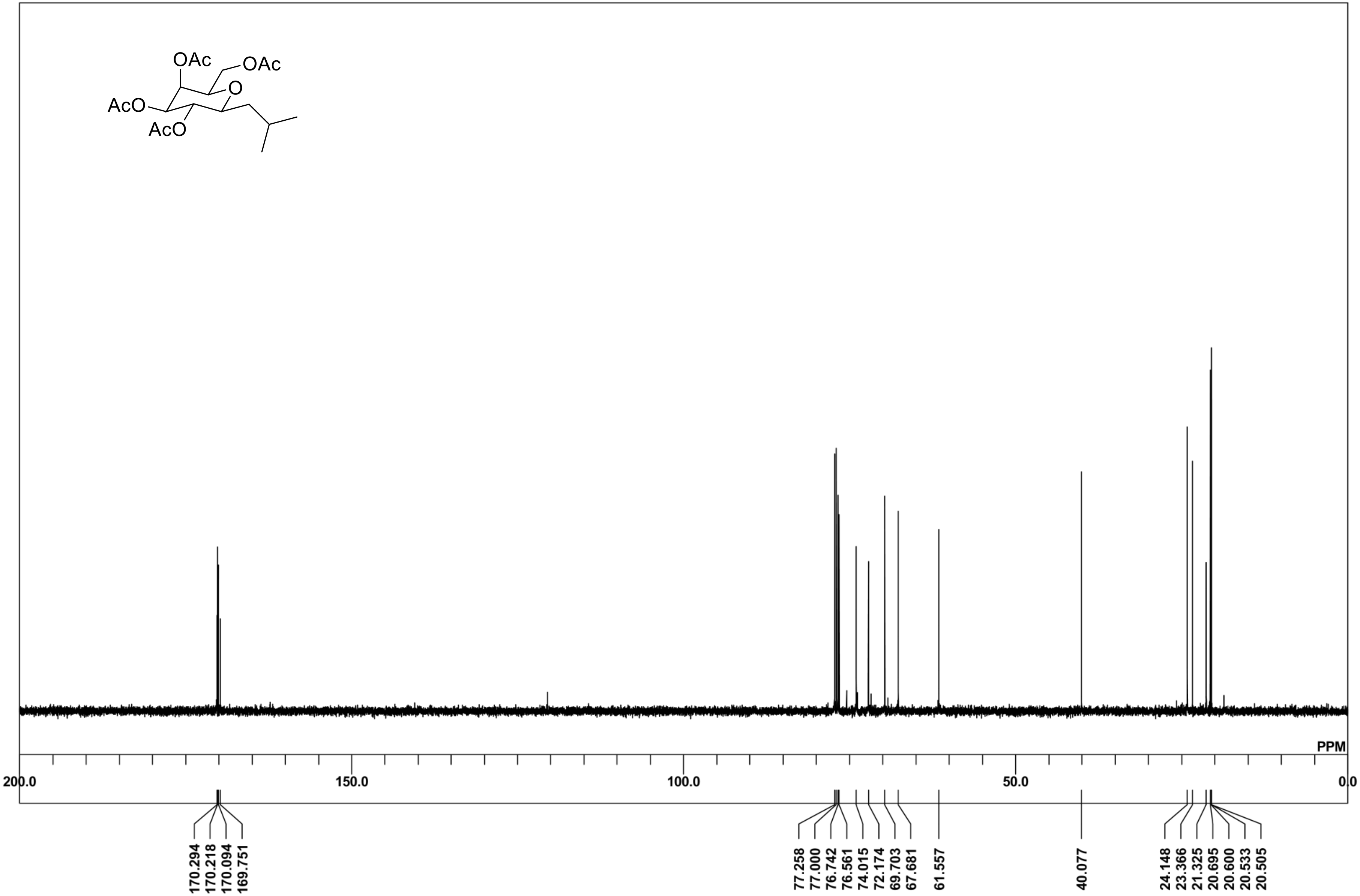

^{1}H NMR spectrum of **2ah**

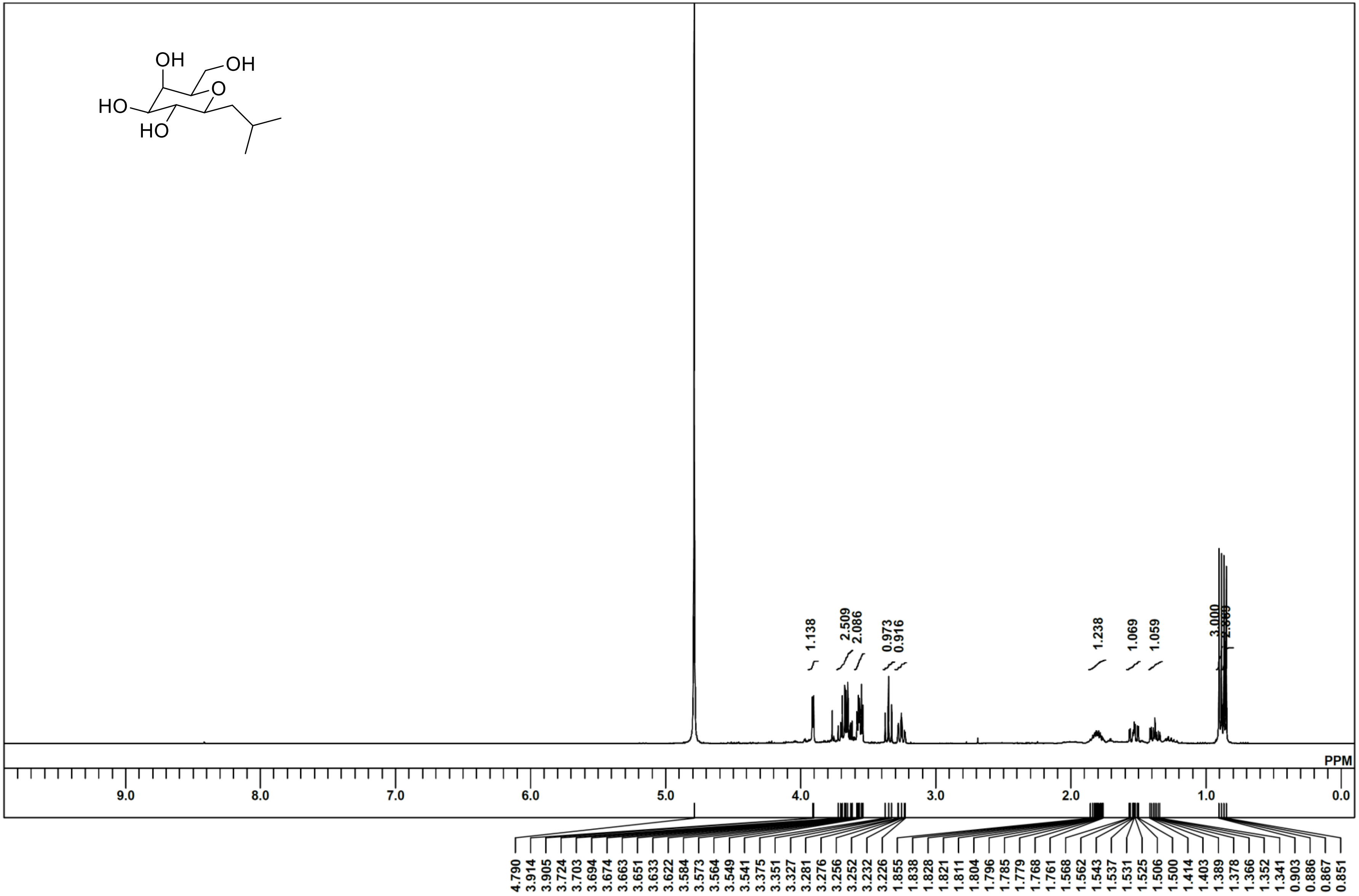

^{13}C NMR spectrum of **2ah**

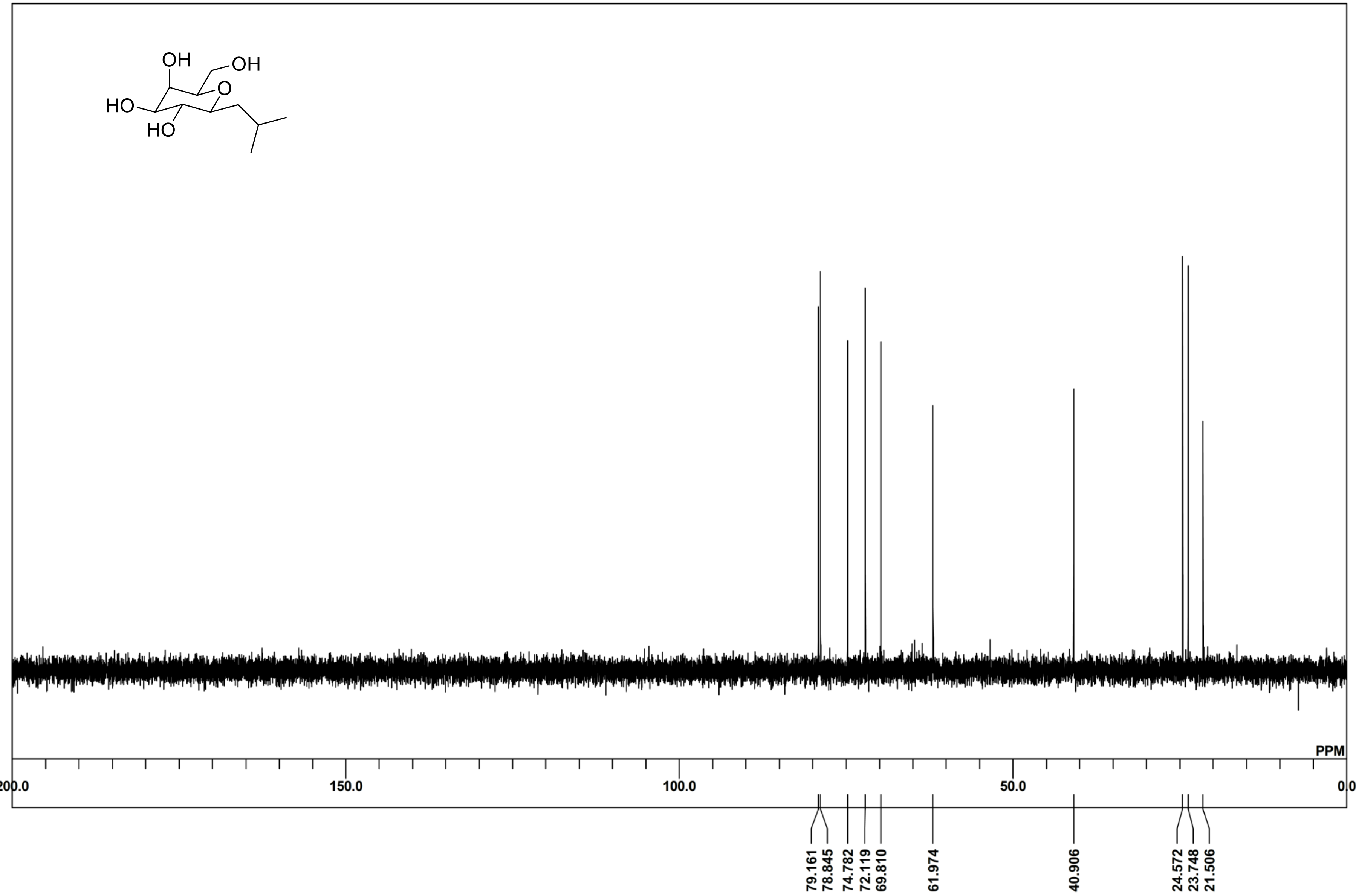

7. Supplementary references

1 Hatano, M., Kamiya, S., Moriyama, K. & Ishihara, K. Lanthanum(III) isopropoxide catalyzed chemoselective transesterification of dimethyl carbonate and methyl carbamates. *Org. Lett.* **13**, 430-433 (2011).

2 Ma, X. & Herzon, S. B. Non-classical selectivities in the reduction of alkenes by cobalt-mediated hydrogen atom transfer. *Chem. Sci.* **6**, 6250-6255 (2015).

3 Choi, G. J. & Knowles, R. R. Catalytic alkene carboaminations enabled by oxidative proton-coupled electron transfer. *J. Am. Chem. Soc.* **137**, 9226-9229 (2015).

4 Vatèle, J.-M. Prenyl carbamates: preparation and deprotection. *Tetrahedron* **60**, 4251-4260 (2004).

5 Xu, H. *et al.* Domino aryne annulation via a nucleophilic–ene process. *J. Am. Chem. Soc.* **140**, 3555-3559 (2018).

6 Dussault, P. H. & Woller, K. R. Approaches to stereoselective dioxygenation of alkenes: Chiral phosphite ozonides. *J. Org. Chem.* **62**, 1556-1559 (1997).

7 Cren, S., Schär, P., Renaud, P. & Schenk, K. Diastereoselectivity control of the radical carboazidation of substituted methylenecyclohexanes. *J. Org. Chem.* **74**, 2942-2946 (2009).

8 Yadav, J. S. & Sengupta, S. The formal total synthesis of FR252921 – An immunosuppressant. *Eur. J. Org. Chem.* 376-388 (2013).

9 Wu, B. *et al.* Synthesis of a comprehensive polyprenol library for the evaluation of bacterial enzyme lipid substrate specificity. *Eur. J. Org. Chem.* 8162-8173 (2013).

10 Braddock, D. C., Cansell, G. & Hermitage, S. A. *Ortho*-Substituted iodobenzenes as novel organocatalysts for bromination of alkenes. *Chem. Commun.* 2483-2485 (2006).

11 Li, Z., Song, L. & Li, C. Silver-catalyzed radical aminofluorination of unactivated alkenes in aqueous media. *J. Am. Chem. Soc.* **135**, 4640-4643 (2013).

12 Okada, Y., Shimada, K., Kitano, Y. & Chiba, K. Short-step anodic access to emissive RNA homonucleosides. *Eur. J. Org. Chem.* 1371-1375 (2014).

13 Liu, L., Abdel Motaal, B., Schmidt-Supprian, M. & Pohl, N. L. B. Multigram synthesis of isobutyl-β-*C*-galactoside as a substitute of isopropylthiogalactoside for exogenous gene induction in mammalian cells. *J. Org. Chem.* **77**, 1539-1546 (2012).

14 Ko, K.-S., Kruse, J. & Pohl, N. L. Synthesis of isobutyl-*C*-galactoside (IBCG) as an isopropylthiogalactoside (IPTG) substitute for increased induction of protein expression. *Org. Lett.* **5**, 1781-1783 (2003).

15 Sonkar, P. K. *et al.* Co(II)-porphyrin-decorated carbon nanotubes as catalysts for oxygen reduction reactions: an approach for fuel cell improvement. *J. Mater. Chem. A* **5**, 6263-6276 (2017).

16 Abel, B. A., Lidston, C. A. L. & Coates, G. W. Mechanism-inspired design of bifunctional catalysts for the alternating ring-opening copolymerization of epoxides and cyclic anhydrides. *J. Am. Chem. Soc.* **141**, 12760-12769 (2019).

17 Peretti, K. L., Ajiro, H., Cohen, C. T., Lobkovsky, E. B. & Coates, G. W. A highly active, isospecific cobalt catalyst for propylene oxide polymerization. *J. Am. Chem. Soc.* **127**, 11566-11567 (2005).

18 Qu, P., Sun, C., Ma, J. & Li, F. The *N*-alkylation of sulfonamides with alcohols in water catalyzed by the water-soluble iridium complex {Cp*Ir[6,6'-$(OH)_2$bpy](H_2O)}[OTf]$_2$. *Adv. Synth. Catal.* **356**, 447-459 (2014).

19 Shi, M. & Shen, Y.-M. Synthesis of mixed carbonates via a three-component coupling of alcohols, CO_2, and alkyl halides in the presence of K_2CO_3 and tetrabutylammonium Iodide. *Molecules* **7**, 386-393 (2002).

20 Ikawa, T., Sajiki, H. & Hirota, K. Highly chemoselective hydrogenation method using novel finely dispersed palladium catalyst on silk-fibroin: its preparation and activity. *Tetrahedron* **61**, 2217-2231 (2005).

21 Iwasaki, K., Wan, K. K., Oppedisano, A., Crossley, S. W. M. & Shenvi, R. A. Simple, chemoselective hydrogenation with thermodynamic stereocontrol. *J. Am. Chem. Soc.* **136**, 1300-1303 (2014).

22 Kleinke, A. S. & Jamison, T. F. Hydrogen-free alkene reduction in continuous flow. *Org. Lett.* **15**, 710-713 (2013).

23 Sajiki, H. & Hirota, K. A novel type of PdMC-catalyzed hydrogenation using a catalyst poison: Chemoselective inhibition of the hydrogenolysis for *O*-benzyl protective group by the addition of a nitrogen-containing base. *Tetrahedron* **54**, 13981-13996 (1998).

24 Dieskau, A. P. & Plietker, B. A mild ligand-free iron-catalyzed liberation of alcohols from allylcarbonates. *Org. Lett.* **13**, 5544-5547 (2011).

25 Alonso, F., Riente, P. & Yus, M. Transfer hydrogenation of olefins catalysed by nickel nanoparticles. *Tetrahedron* **65**, 10637-10643 (2009).

26 Messmore, B. W., Sukerkar, P. A. & Stupp, S. I. Mirror image nanostructures. *J. Am. Chem. Soc.* **127**, 7992-7993 (2005).

27 King, S. M., Ma, X. & Herzon, S. B. A method for the selective hydrogenation of alkenyl halides to alkyl halides. *J. Am. Chem. Soc.* **136**, 6884-6887 (2014).

28 Ishikawa, H. *et al.* Total synthesis of vinblastine, vincristine, related natural products, and key structural analogues. *J. Am. Chem. Soc.* **131**, 4904-4916 (2009).

29 Ma, X., Dang, H., Rose, J. A., Rablen, P. & Herzon, S. B. Hydroheteroarylation of unactivated alkenes using *N*-methoxyheteroarenium salts. *J. Am. Chem. Soc.* **139**, 5998-6007 (2017).

30 Maeda, S., Harabuchi, Y., Takagi, M., Taketsugu, T. & Morokuma, K. Artificial force induced reaction (AFIR) method for exploring quantum chemical potential energy surfaces. *Chem. Rec.* **16**, 2232-2248 (2016).

31 Maeda, S., Harabuchi, Y., Sumiya, Y., Takagi, M., Suzuki, K., Hatanaka, M., Osada, Y., Taketsugu, T., Morokuma, K. & Ohno, K. GRRM-Global reaction route mapping: GRRM17. Available at: http://iqce.jp/GRRM/index_e.shtml. (Accessed: 29th October 2020)

32 Maeda, S., Ohno, K. & Morokuma, K. Systematic exploration of the mechanism of chemical reactions: the global reaction route mapping (GRRM) strategy using the ADDF and AFIR methods. *Phys. Chem. Chem. Phys.* **15**, 3683-3701 (2013).

33 Bannwarth, C., Ehlert, S. & Grimme, S. GFN2-xTB-An accurate and broadly parametrized self-consistent tight-binding quantum chemical method with multipole electrostatics and density-dependent dispersion contributions. *J. Chem. Theory Comput.* **15**, 1652-1671 (2019).

34 Neese, F. Software update: the ORCA program system, version 4.0. *WIREs Comput. Mol. Sci.* **8**, Article number: e1327 (2018).

35 Gaussian 16, Revision A.03, Frisch, M. J., Trucks, G. W., Schlegel, H. B., Scuseria, G. E., Robb, M. A., Cheeseman, J. R., Scalmani, G., Barone, V., Petersson, G. A., Nakatsuji, H., Li, X., Caricato, M., Marenich, A. V., Bloino, J., Janesko, B. G., Gomperts, R., Mennucci, B., Hratchian, H. P., Ortiz, J. V., Izmaylov, A. F., Sonnenberg, J. L., Williams-Young, D., Ding, F., Lipparini, F., Egidi, F., Goings, J., Peng, B., Petrone, A., Henderson, T., Ranasinghe, D., Zakrzewski, V. G., Gao, J., Rega, N., Zheng, G., Liang, W., Hada, M., Ehara, M., Toyota, K., Fukuda, R., Hasegawa, J., Ishida, M., Nakajima, T., Honda, Y., Kitao, O., Nakai, H., Vreven, T., Throssell, K., Montgomery, J. A., Jr., Peralta, J. E., Ogliaro, F., Bearpark, M. J., Heyd, J. J., Brothers, E. N., Kudin, K. N., Staroverov, V. N., Keith, T. A., Kobayashi, R., Normand, J., Raghavachari, K., Rendell, A. P., Burant, J. C., Iyengar, S. S., Tomasi, J., Cossi, M., Millam, J. M., Klene, M., Adamo, C., Cammi, R., Ochterski, J. W., Martin, R. L., Morokuma, K., Farkas, O., Foresman, J. B. & Fox, D. J. Gaussian, Inc., Wallingford CT, 2016.

36 Avogadro: an open-source molecular builder and visualization tool. Version 1.2.0. http://avogadro.cc/ (Accessed: 29th October 2020)

37 Hanwell, M. D. *et al.* Avogadro: An advanced semantic chemical editor, visualization, and analysis platform. *J. Cheminf.* **4**, Article number: 17 (2012).

38 de Bruin, B., Dzik, W. I., Li, S. & Wayland, B. B. Hydrogen-atom transfer in reactions of organic radicals with $[Co^{II}(por)]^{\bullet}$ (por = porphyrinato) and in subsequent addition of [Co(H)(por)] to olefins. *Chem. Eur. J.* **15**, 4312-4320 (2009).

39 Wahidur Rahaman, S. M., Matyjaszewski, K. & Poli, R. Cobalt(III) and copper(II) hydrides at the crossroad of catalysed chain transfer and catalysed radical termination: a DFT study. *Polym. Chem.* **7**, 1079-1087 (2016).
